# Integrated next-generation sequencing of 16S rDNA and metaproteomics differentiate the healthy urine microbiome from asymptomatic bacteriuria in neuropathic bladder associated with spinal cord injury

**DOI:** 10.1186/1479-5876-10-174

**Published:** 2012-08-28

**Authors:** Derrick E Fouts, Rembert Pieper, Sebastian Szpakowski, Hans Pohl, Susan Knoblach, Moo-Jin Suh, Shih-Ting Huang, Inger Ljungberg, Bruce M Sprague, Sarah K Lucas, Manolito Torralba, Karen E Nelson, Suzanne L Groah

**Affiliations:** 1 J Craig Venter Institute, 9704 Medical Center Drive, Rockville, MD, 20850, USA; 2 Childrens National Medical Center, 111 Michigan Avenue NW, Washington, DC, 20010, USA; 3 MedStar National Rehabilitation Hospital, 102 Irving Street NW, Washington, DC, 20010, USA; 4 Georgetown University Hospital, 3800 Reservoir Road, Washington, DC, 20007, USA

**Keywords:** Bacteriuria, Urine, Catheter, Neuropathic, Bladder, Microbiome, Metaproteome, Next-generation, Personalized, rRNA

## Abstract

**Background:**

Clinical dogma is that healthy urine is sterile and the presence of bacteria with an inflammatory response is indicative of urinary tract infection (UTI). Asymptomatic bacteriuria (ABU) represents the state in which bacteria are present but the inflammatory response is negligible. Differentiating ABU from UTI is diagnostically challenging, but critical because overtreatment of ABU can perpetuate antimicrobial resistance while undertreatment of UTI can result in increased morbidity and mortality. In this study, we describe key characteristics of the healthy and ABU urine microbiomes utilizing 16S rRNA gene (16S rDNA) sequencing and metaproteomics, with the future goal of utilizing this information to personalize the treatment of UTI based on key individual characteristics.

**Methods:**

A cross-sectional study of 26 healthy controls and 27 healthy subjects at risk for ABU due to spinal cord injury-related neuropathic bladder (NB) was conducted. Of the 27 subjects with NB, 8 voided normally, 8 utilized intermittent catheterization, and 11 utilized indwelling Foley urethral catheterization for bladder drainage. Urine was obtained by clean catch in voiders, or directly from the catheter in subjects utilizing catheters. Urinalysis, urine culture and 16S rDNA sequencing were performed on all samples, with metaproteomic analysis performed on a subsample.

**Results:**

A total of 589454 quality-filtered 16S rDNA sequence reads were processed through a NextGen 16S rDNA analysis pipeline. Urine microbiomes differ by normal bladder function vs. NB, gender, type of bladder catheter utilized, and duration of NB. The top ten bacterial taxa showing the most relative abundance and change among samples were Lactobacillales, Enterobacteriales, Actinomycetales, Bacillales, Clostridiales, Bacteroidales, Burkholderiales, Pseudomonadales, Bifidobacteriales and Coriobacteriales. Metaproteomics confirmed the 16S rDNA results, and functional human protein-pathogen interactions were noted in subjects where host defenses were initiated.

**Conclusions:**

Counter to clinical belief, healthy urine is not sterile. The healthy urine microbiome is characterized by a preponderance of *Lactobacillales* in women and *Corynebacterium* in men. The presence and duration of NB and method of urinary catheterization alter the healthy urine microbiome. An integrated approach of 16S rDNA sequencing with metaproteomics improves our understanding of healthy urine and facilitates a more personalized approach to prevention and treatment of infection.

## Background

Affecting nearly one half of all Americans over the course of a lifetime [1] and with costs exceeding $1 billion annually [2], urinary tract infection (UTI) is a major public health problem [3,4]. It is the most common urologic disorder in the outpatient setting [3,4] and the most common health care associated infection [3,5,6]. In the health care setting alone, approximately 561777 UTIs occur annually, costing an estimated $1006 per infection, totaling more than $500 million, and being responsible for 8205 deaths [5]. This does not include the personal suffering or time lost from gainful employment.

Often considered an antecedent to UTI, asymptomatic bacteriuria (ABU) represents an asymptomatic carrier state recognized to have little impact on health or quality of life. This is in contrast to healthy urine, considered to be sterile until reaching the urethra, which is colonized by facultative anaerobic Gram-negative rods and cocci. The most common risk factor for the development of ABU and UTI is use of a urinary catheter [5-7], as catheters provide a conduit for bacterial colonization and symptomatic infection. Resolution of ABU typically occurs with removal of the urinary catheter. This is not possible in many cases, however, as the urinary catheter facilitates function and emptying in cases of bladder impairment.

The clinical distinction between symptomatic UTI and ABU is not trivial since symptomatic UTI requires treatment and perhaps further evaluation irrespective of the circumstances in which the UTI occurred, while ABU does not. Exceptions to this rule include the treatment of ABU in select at-risk populations, such as pregnant women, which has been shown to be associated with improved outcomes [8]. Distinction between these states is particularly relevant, as renewed urgency and heightened focus have been placed on UTI occurrence by national policy-makers and payers. The Centers for Medicare & Medicaid Services (CMS) has identified catheter-associated UTI, the most common hospital acquired infection, as a “never event”. Effective in 2008, this has resulted in non-reimbursement for catheter-associated UTIs that were not present on admission to acute care hospitals [9,10]. An unintended consequence of designating catheter-associated UTI as a “never event” is more aggressive screening for ABU and UTI upon admission of patients to hospitals, a strategy that may lead to increased unnecessary antibiotic treatment and emergence of antimicrobial resistance.

To achieve improved outcomes in the care of patients with ABU and UTI, improved information distinguishing states of urine in health and disease is needed. To this end, we sought to first describe states of urinary health utilizing a highly sensitive, culture independent approach to determine whether the urine microbiome of healthy people who are at risk for ABU because they utilize urinary catheters differs from that of healthy controls, and if so, to identify key factors or bacterial signatures that might ultimately lead to UTI requiring antimicrobial treatment. Subjects with neuropathic bladder due to spinal cord injury who are known to be at highest risk for ABU and UTI due to their need for catheter-assisted bladder management [4,11,12] were assessed and compared with healthy controls to achieve our goal.

## Methods

The study was approved by the MedStar Institutional Review Board (IRB). All study personnel were certified in and the study protocol conformed to the ethical guidelines of the 1975 Declaration of Helsinki as reflected in approval by the MedStar IRB.

### Sample acquisition and clinical urinalysis

Patients and healthy controls were recruited into this IRB-approved study (NRH IRB# 2011–019) from the outpatient clinic and inpatient ward at National Rehabilitation Hospital (Washington, DC). Following written consent, urine samples were obtained from 26 healthy, non-SCI controls and 27 with neuropathic bladder (NB) due to spinal cord injury (SCI). Patients provided urine samples by sterile collection using the means by which they customarily empty their bladder (i.e. midstream collection during voiding, or sterile catheterization if unable to void). (Table 1) The samples were coded with an anonymous research identification number and separated into two aliquots: one, for standard urine analysis and culture (Quest Diagnostics) and another for microbiome analysis. Quest Diagnostics performed analysis of urine samples for nitrite formation, leukocyte esterase, and microscopic examination for the presence and quantity of leukocytes and erythrocytes in each sample. Bacterial cultures were performed by inoculation of blood agar plates and incubation at 37°C for 48 hours.

**Table 1:** Patient demographics

**Group**	**Gender**	**Age**	**Race/Ethnicity**	**Months with NB**	**Urinanalysis**		**Urine culture with >50000 cfu**
					**Leukocyte esterase**	**WBC (no./hpf)**	
**Healthy controls**	**Female (57.7%)**	**Mean 35.6**					
S01	Female	40	Asian	n/a	NEG	0	NEG
S02	Male	24	Asian	n/a	NEG	0	NEG
S03	Female	32	Caucasian	n/a	NEG	0	NEG
S04	Female	35	Caucasian	n/a	NEG	0	*Streptococcus* (beta-hemolytic)
S05	Male	32	Caucasian	n/a	NEG	0-1	NEG
S06	Female	57	Caucasian	n/a	NEG	0-1	NEG
S07	Male	35	Caucasian	n/a	NEG	0-1	NEG
S08	Female	43	African American	n/a	NEG	0-1	NEG
S09	Female	25	Caucasian	n/a	NEG	0-1	NEG
S10	Female	34	Caucasian	n/a	NEG	0-1	NEG
S11	Male	33	Caucasian	n/a	NEG	0-1	NEG
S12	Male	29	Asian	n/a	NEG	0-1	NEG
S13	Male	35	Caucasian	n/a	NEG	0-1	NEG
S14	Female	22	Caucasian	n/a	NEG	0-1	*Staphylococcus*
S15	Female	34	Asian	n/a	NEG	0-1	*Lactobacillus*
S16	Female	45	Caucasian	n/a	NEG	0-1	*Lactobacillus*
S17	Female	46	African American	n/a	TRA	0-1	*Escherichia coli*
S18	Female	51	Asian	n/a	NEG	1-2	*Escherichia coli, Staphylococcus aureus*
S19	Female	40	Caucasian	n/a	NEG	1-2	*Lactobacillus*
S20	Male	50	Caucasian	n/a	NEG	1-2	NEG
S21	Male	25	Caucasian	n/a	NEG	5-9	NEG
S22	Male	29	Caucasian	n/a	1+	1-2	NEG
S23	Female	30	Caucasian	n/a	1+	3-4	NEG
S24	Male	39	Caucasian	n/a	2+	5-9	NEG
S25	Female	27	Asian	n/a	2+	100+	*Escherichia coli*
S26	Male	33	Asian	n/a	3+	30-49	NEG
**NB - void**	**Female (37.5%)**	**Mean 37.3**		**Mean 41.5**			
S27	Male	20	African American	9	ND	ND	NEG
S28	Male	31	African American	157	ND	ND	NEG
S29	Male	19	Caucasian	3	NEG	1-2	*Klebsiella pneumoniae*
S30	Female	41	African American	1	NEG	1-2	*Enterococcus faecalis*
S31	Female	54	Caucasian	1	TRA	1-2	NEG
S32	Male	31	African American	158	TRA	3-4	*Enterococcus faecalis*
S33	Male	48	Hispanic	1	2+	5-9	*Escherichia coli, Enterococcus faecalis*
S34	Female	54	Caucasian	2	2+	5-9	*Klebsiella oxytoca*
**NB-IC**	**Female (50.0%)**	**Mean 44.1**		**Mean 140.1**			
S35	Male	40	African American	84	NEG	0	*Escherichia coli*
S36	Female	36	Caucasian	7	NEG	0-1	*Escherichia coli*
S37	Female	55	Caucasian	3	NEG	1-2	NEG
S38	Female	55	Caucasian	442	NEG	1-2	NEG
S39	Male	48	Native American	261	NEG	3-4	*Klebsiella pneumoniae*
S40	Male	48	Native American	260	1+	3-4	NEG
S41	Female	50	Caucasian	2	2+	3-4	NEG
S42	Male	21	African American	62	2+	5-9	*Proteus*
**NB-FC**	**Female (54.5%)**	**Mean 37.6**		**Mean 136**			
S43	Female	47	African American	79	ND	ND	NEG
S44	Female	47	African American	80	NEG	0	*Enterococcus faecalis*, Gram Negative Rods
S45	Male	23	African American	20	NEG	0-1	*Escherichia coli* (ESBL), *Klebsiella pneumoniae, Providencia stuartii, Pseudomonas aeruginosa, Enterococcus faecalis*
S46	Female	40	African American	236	NEG	1-2	*Escherichia coli, Citrobacter koseri* (diversus), *Enterococcus faecalis*
S47	Female	40	African American	235	NEG	3-4	NEG
S48	Female	61	Caucasian	469	TRA	1-2	*Escherichia coli*
S49	Male	27	African American	92	1+	3-4	*Pseudomonas aeruginosa*
S50	Female	40	African American	235	1+	15-19	NEG
S51	Male	48	African American	18	2+	5-9	*Escherichia coli, Escherichia coli *(ESBL), *Pseudamonas aeruginosa, Enterococcus faecalis*
S52	Male	20	African American	25	2+	10-14	NEG
S53	Male	21	African American	7	2+	50+	NEG

No.= number, WBC = white blood cells, hpf = high power field, HC = healthy control, IC = intermittent catheter, FC = Foley catheter, NEG = negative, TRA = trace, n/a = not applicable, ND = not determined, ESBL = Extended-Spectrum-Beta-Lactamases.

### Sample preparation and PCR

Thawed urine samples were clarified by low-speed centrifugation and bacterial genomic DNA was extracted from urine pellets by enzymatic digestion using a final concentration of 20 mg/ml Lysozyme (Invitrogen) followed by physical lysis using Lysing Matrix B tubes (QBiogene). A previous study comparing mechanical and enzymatic methods for extracting microbial genomic DNA showed that mechanical cell disruption by bead beating produced the highest bacterial diversity [13]. The samples were vortexed at maximum speed for 45 seconds using a Fastprep fp120 (MP Biomedicals) then cooled on ice. DNA was extracted from the lysate using phenol chloroform isoamyl alcohol extraction and ethanol precipitation. 16S rDNA sequences were generated by amplifying the V1-V3 region of the bacterial 16S rRNA gene using primers 27F and 534R fused with 454 adaptors and barcodes for multiplexing. Primers targeting V2 and V3 were shown to perform as well as full-length 16S rDNA sequence for community clustering and taxonomic assignments [14].

The amplicons were normalized and pooled prior to emulsion PCR and 454 sequencing (Roche, Inc.) using titanium chemistry.

### DNA sequence processing

The 16S rDNA sequence-processing pipeline used for this study is composed of a selection of bioinformatics tools proven to be accurate, robust and fast. A supplementary archive contains the dot language representation of a graph depicting the entire workflow executed, outlining the specific parameters used for each command.

Initially, the SFF file, output from the sequencer, was converted into fasta and qual files using the sffinfo program included as a part of 454/Roche software package. Subsequently, the trim.seqs function in mothur [15] (version v.1.22.2) was used to de-multiplex sequencer reads. No barcode mismatches, and up to one primer mismatch were allowed past this step. The de-multiplexed reads were processed using LUCY [16-18] to filter out reads with low quality segments. At this point, the sub.sample function of mothur was used to select an equal number of reads per biological sample (n=3671 based on the biological sample with the fewest number of reads). Subsequent, the screen.seqs function of mothur was used to remove sequences shorter than 220 bases [17]. Furthermore CD-HIT-454 [19,20] was used to collapse duplicate reads, while retaining their count for subsequent enrichment statistics, (analogous to the functionality of the unique.seqs function of mothur, but orders of magnitude faster and less demanding on the computer hardware). The sequences were aligned against the SILVA database of 16S rDNA sequences [15,21] to verify 1) the orientation of noise-filtered sequences; and 2) the correct positioning of the reads with respect to the expectation of which variable regions should have been amplified and sequenced. Thereafter, the remaining sequences were subjected to mothur’s implementation of chimera slayer [15,22] to filter out chimeric reads. The processed 16S rDNA data from this study can be obtained at NCBI under BioProject ID 97505.

### Taxonomical classification of OTU representative reads

Taxonomical classification of the final set of 82160 operational taxonomic unit (OTU)-representative reads down to the genus level was performed using mothur’s version of the RDP Bayesian classifier using a normalized RDP training dataset [23]. The final step of the pipeline clustered the sequences based on their similarity to produce OTUs. Customarily, a similarity threshold of 97% has been used to define OTUs at approximately the species level [24]. A module of CD-HIT suite [19] called CD-HIT-EST was employed to perform species-level read-clustering for subsequent analyses.

### Statistical analyses

The orchestration and automation of steps has been achieved using a custom set of in-house utilities written in python and R [25] programming languages. These utilities are available online as a part of the YAP package on github [26]. JCVI grid infrastructure based on the Oracle Grid Engine (OGE) was used for all steps described. Relative abundance and diversity statistics were calculated within mothur [15]. Further statistical analyses were accomplished using R. Heat maps were generated using the *heatmap.2* function of the *gplots* package available on CRAN [25]. OTU counts have been normalized to 100% per individual, to facilitate comparability. Only taxa (order or genus) with a standard deviation greater than 5% across all 52 individuals were used to generate the heat maps. Differences between subject OTU communities were assessed using the Bray-Curtis beta diversity statistic implemented in the *vegdist* function, a part of vegan R package available on CRAN. Clustering was accomplished using average-neighbor-joining method implemented in *hclust* function in the default installation of R. PCA analysis was performed using *ade4*[27]. P-values used to determine statistical significance in relative OTU difference plots were established using the default installation of R and *kruskal.test* functions implementing the Kruskal-Wallis rank-sum statistic test [28].

### Phylogenetic tree building

OTU-representative sequences classified as either Lactobacillales or Enterobacteriales were aligned using tools available from Release 10 of the RDP web site [29]. Specifically, sequences from OTUs composed of reads from more than one individual were aligned to the RDP reference 16S rRNA sequence, taking into account secondary structure. At most one nearest neighbor sequence from RDP was recruited into the alignment per input sequence. The alignment was downloaded and trimmed to remove columns whose gap fractions were greater than 50%, using Belvu [30]. Based on the alignment, a bootstrapped Neighbor-Joining (NJ) tree was subsequently inferred using paupFasta, an in-house wrapper script around the PAUP* program as described [31], and edited using Fig Tree [32]. In combination with nearest neighbor taxonomy, BLASTN was used against the NCBI reference RNA database, which lacks uncultured organisms, to identify certain Lactobacillales OTU-representative branches that lacked classified RDP top matches, to the species level, using a cut-off of 97% identity.

### Proteomics

A urinary pellet specimen equivalent to 5–10 ml voided urine washed two times with ~10 ml ice-cold PBS was re-suspended in 1 ml of 10 mM ammonium bicarbonate containing 0.1% Triton-X100, 0.5% octylglucoside, 5 μg/ml leupeptin, 10 mM EDTA and 2 mM benzamidine. The suspension was heated to 85°C for 5 min followed by sonication (amplitude 4, Misonex 3000 sonicator) in 30s on/15s off cycles 10 times on ice. The suspension was centrifuged for 15 min at 16,100 x g and the supernatant recovered. Following protein quantity estimates based on SDS-PAGE analysis, an aliquot with ~10 μg protein was digested at a 1:50 ratio (trypsin/protein) using Filter-Aided Sample Preparation (FASP) [33]. The digestion mixture was reconstituted in 50 μl 0.1% formic acid. For shotgun proteomic analysis, peptides in a 20 μl sample aliquot were separated on a capillary C_18_ LC column in 122 min binary gradient runs from 97% solvent A (0.1% formic acid) to 80% solvent B (0.1% formic acid, 90% acetonitrile) at a flow rate of 350 nl/min. Nano-electrospray into the source was followed by mass analysis and spectral acquisition in automated MS/MS mode, with the top five parent ions selected for fragmentation in scans of the m/z range 350–2,000 and a dynamic exclusion setting of 90 sec. The LC-MS/MS workflow using the LTQ-XL ion trap system (Thermo Fisher Scientific) was previously described in more detail [34]. The instrument was calibrated prior to performance of LC-MS/MS experiments with 200 nmol human [Glu^1^-fibrinopeptide B (M.W. 1570.57), verifying that peaks representing ion counts had widths at half-height of <0.25 min, signal/noise ratios >200 and peak heights >10^7^. The LTQ search parameters (+1 to +3 ions) included mass error tolerances of ± 1.4 Da for peptide precursor ions and ± 0.5 Da for peptide fragment ions, selecting monoisotopic m/z values. The search engine used to select these parameters and identify peptides and proteins was Mascot v.2.3 (Matrix Science). The protein sequence database was comprised of 19 bacterial genomes (details in Additional file 1) and Uniref90’s human database subset [35]. We limited this database to the human Uniref90 subset and 19 bacterial genomes frequently associated with ABU and UTI, because significant computational challenges for peptide-spectral match (PSM) assignments are encountered when very large protein databases are used [36]. As described in Additional file 1, we used stringent criteria for PSMs (q-value <=0.01; PEP-value <=10^-4^) using the Mascot Percolator algorithm that improves discrimination between correct and incorrect PSMs, particularly when the searched sequence space in the database is large [37].

## Results

Urine samples were obtained from 26 healthy, non-SCI controls and 27 individuals with NB due to SCI at the National Rehabilitation Hospital in Washington, DC. Among those with SCI and NB, 8 voided spontaneously without a catheter, 8 emptied by clean intermittent catheterization (IC), and 11 used indwelling urethral Foley catheterization (FC) (Table 1). Frozen, resuspended pellets from the urine samples were analyzed with culture-independent surveys of bacterial 16S rDNA and urinary proteins.

**Figure 1:**
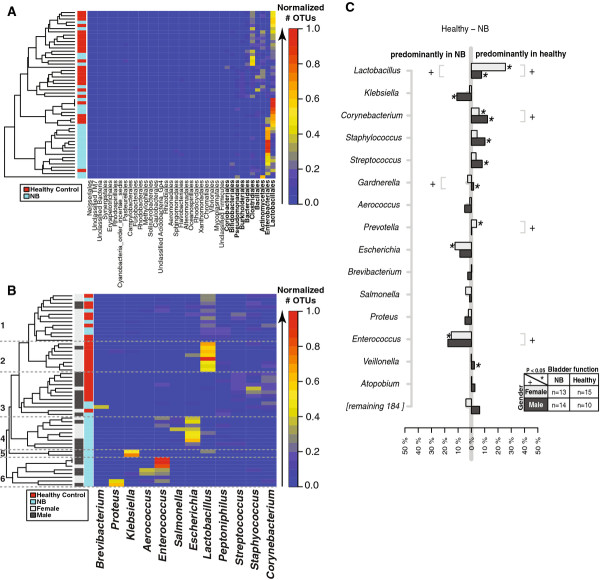
**Differences in relative bacterial OTU counts between neuropathic and healthy bladder in males and females.** For every individual, the OTU counts were normalized to the individual's total OTU count. A heat map of the clustered distribution of OTU taxonomy at the level of bacterial order (**A**) and genus (**B**) was constructed using the Bray-Curtis index. For panels A and B, only the taxa with a standard deviation > 5% across all individuals are shown. Differences in the average OTU count between females and males are plotted in light and dark gray, respectively (C). In panel (**C**), the top 15 (8%) most abundant bacterial OTUs are represented. The X-axis indicates the difference in relative OTU counts per bacterial genus indicated on the Y-axis. Statistical significance was established using Kruskal-Wallis test. Significant differences (P < 0.05) between the relative OTU counts are indicated by an asterisk (*) for bladder function, and a plus sign (+) for gender.

A total of 589454 quality-filtered 454 16S rDNA sequence reads were processed through a NextGen 16S rDNA analysis pipeline [26] (Additional file 2) where taxonomy of species-level operational taxonomic units (OTUs) was determined using the RDP classifier [38]. Analysis of the bacterial community in these urine samples revealed between 5 and 236 species-level (97% identity) OTUs per individual (Additional file 3). Per sample taxonomic profiles were generated, showing considerable sample-to-sample variation (Additional file 4). To better visualize sample-to-sample taxonomic profiles, a heat map was generated, clustering the distribution of OTU taxonomy at the level of bacterial order using the Bray-Curtis index (Figure 1A). The top ten bacterial taxa showing the most relative abundance and change among samples were Lactobacillales, Enterobacteriales, Actinomycetales, Bacillales, Clostridiales, Bacteroidales, Burkholderiales, Pseudomonadales, Bifidobacteriales and Coriobacteriales (Figure 1A). The Lactobacillales and Enterobacteriales were the two most relative abundant and changing taxonomic groups. 

### Urinary microbiome differed by gender and bladder function

To determine whether distinct microbial signatures were associated with gender (male versus female) and/or bladder function (healthy control versus NB), samples were grouped by these variables. Taxonomic counts were then normalized by total number of OTUs per sample, and visualized by a heatmap, clustering the distribution of OTUs at the level of bacterial genus using the Bray-Curtis index (Figure 1B). The samples were colored by relative abundance (red/warm most abundant to blue/cool least abundant). Six main taxonomic profile clusters emerged with distinct patterns when grouped by gender and bladder function. Cluster 1 was composed of almost an equal proportion of healthy and NB samples, clusters 2 and 3 were dominated by healthy controls, whereas clusters 4–6 were entirely composed of patients with NB (Figure 1B). The 2 “healthy” clusters, (2 and 3) were distinguished by gender, with females in cluster 2 and males in cluster 3, and by bacterial genus, *Lactobacillus* grouping with females in cluster 2 and completely absent in the male-dominated cluster 3. Cluster 3 was composed of different Gram-positive organisms. Cluster 1 had the most diverse bacterial genus profile, composed largely of *Lactobacillus*, but not as abundant as cluster 2 with elements of cluster 3 and a few potentially pathogenic genera (*Enterococcus*, *Salmonella*, and *Peptoniphilus*). Closer inspection of clusters 4–6 showed a very different pattern of bacteria, with known UTI pathogens dominating the profiles (Figure 1B). Cluster 4 was primarily composed of *Enterococcus*, *Escherichia* and *Salmonella*. Cluster 5 was dominated by *Klebsiella* sp. and was the only cluster comprised of all males. Cluster 6 had the most *Enterococcus* counts of any cluster and also contained *Aerococcus* and *Proteus* sp.

By taking the difference in normalized relative abundance between controls and SCI groups, the most relative abundant bacterial taxa per group as confirmed (Figure 1C). Statistical significance was established using the Kruskal-Wallis test. Significant differences (P < 0.05) between the top relative OTU counts suggest that *Lactobacillus, Corynebacterium, Gardnerella, Prevotella* and *Enterococcus* define gender differences (“+” in Figure 1C). *Lactobacillus, Klebsiella, Corynebacterium, Staphylococcus, Streptococcus, Gardnerella*, *Prevotella, Escherichia* and *Enterococcus* defined statistically significant differences between healthy bladder controls and NB (“*” in Figure 1C).

### Urinary microbiome differed with duration of NB

**Figure 2:**
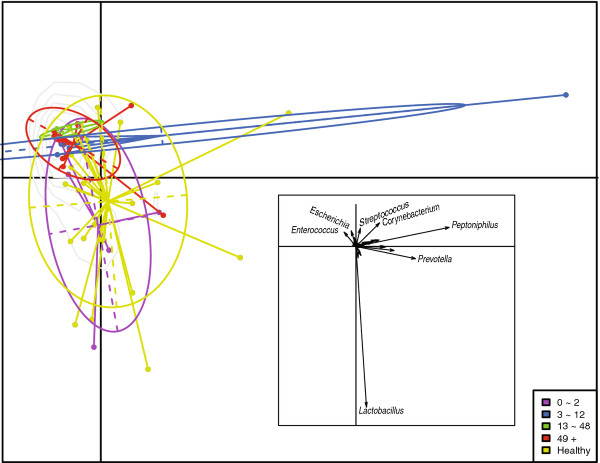
**OTU differences among individuals by duration of neuropathic bladder.** A PCA analysis of the OTU counts of 52 individuals. The points are circled and colored based on the duration (in months) of neuropathic bladder (see key). The inset depicts a vector plot indicating the most influential principal component (bacterial genus).

To further investigate the dynamics of the urinary microbiome in NB, the NB group was divided into bins based on the time post SCI. The bins consisted of 0–2 months (n=5), 3–12 months (n=5), 13–48 months (n=3), and 49+ months (n=14). Principal component analysis (PCA) revealed that the healthy control group and the 0–2 month duration were very similar (Figure 2). Likewise, the 13–48 and 49+ month groups were similar to each other, while the 3–12 month group was too distorted by one outlier to provide meaningful information. *Enterococcus* and *Escherichia* emerged as major contributors to these profiles as illustrated in the vector diagram (inset, Figure 2).

### Urinary microbiome differed with bladder management

**Figure 3:**
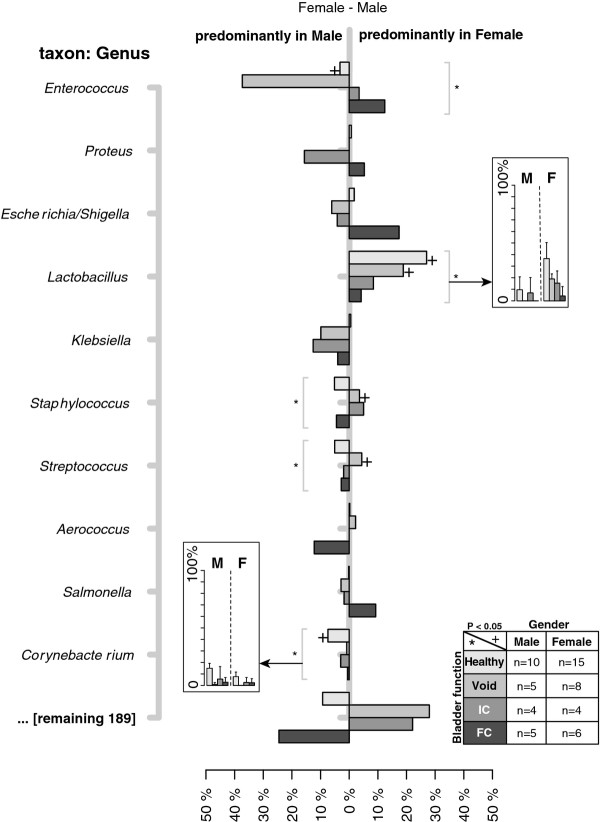
**Differences in the relative OTU counts between males and females stratified by bladder management.** For every individual, the OTU counts were normalized to the individual’s total OTU count. Differences between relative OTU counts were calculated by subtracting the average OTU counts from females and males per bladder management category; healthy control, void (SCI patient with no catheter usage), indwelling catheter (IC), and Foley catheter (FC) (see key for color coding and sample sizes). The X-axis indicates the difference in relative OTU counts per bacterial genus indicated on the Y-axis. Significant differences (P < 0.05) between the relative OTU counts are indicated by plus sign (+) for gender, and an asterisk (*) for bladder management. The inset depicts the mean and standard deviation of OTU counts of the indicated genera for each catheter management group.

To get an overview of bacterial species variation within the NB group, differences in microbiome taxonomic profiles between males and females and among bladder management groups were plotted (Figure 3). *Lactobacillus* species were significant (P < 0.05) contributors to female healthy controls and the void NB groups, while *Corynebacterium* sp. defined the healthy bladder male urinary microbiome (Figure 3). The preponderance of *Enterococcus* sp. in male NB observed in Figure 1C was primarily within the patients with NB who emptied by spontaneous voiding (Figure 3).

### Phylogenetic tree of the urinary Lactobacillales revealed potentially pathogenic species

**Figure 4:**
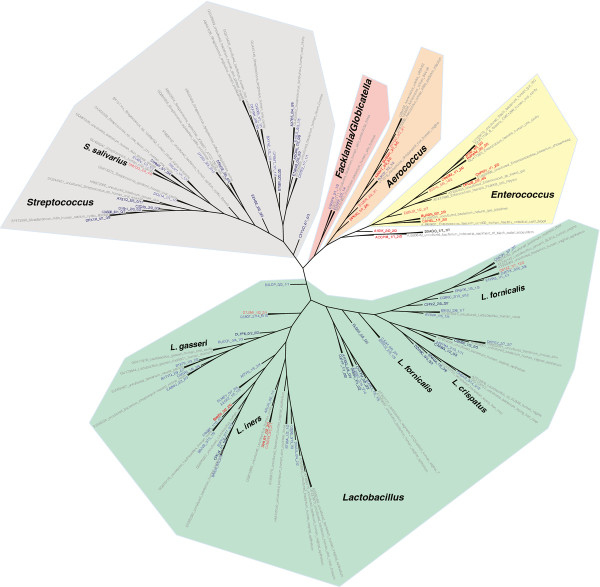
**Phylogenetic diversity of Lactobacillales 16S rDNA sequences in human urine.** NJ tree clustering of Lactobacillales OTU representatives labeled based on similarity to known RDP database sequences (gray), and OTU composition. Leaves are colored as follows: OTUs consisting of only healthy individuals (dark blue), mostly healthy (light blue), only NB (red), mostly NB (pink/salmon). Branches were highlighted and labeled by identifiable bacterial genera. Genus-level classification was based on the OTU representative RDP classification and the classification of nearest neighbors the RDP alignment. The nodes show SequenceID_#male/#female_#SCI/#healthy subjects.

A phylogenetic tree was constructed with OTU representatives of all OTUs with matches to Lactobacillales and nearest neighbors in the RDP database in order to provide an environmental context and determine species-level taxonomy (Figure 4). Labels of the tree were colored based on NB composition. Red indicates OTUs composed only of samples from NB, light red indicates OTUs with a majority composition of NB samples, while dark blue denotes OTUs only solely of healthy controls, and light blue illustrates OTUs with a majority of reads from healthy controls. There is much greater phylogenetic diversity among the *Lactobacillus* and *Streptococcus* branches (green and gray shading, respectively, Figure 4). *Aerococcus* and *Enterococcus* branches are dominated by OTUs (red leaves) from urine of NB patients, suggesting qualitatively that these two genera may be important indicators of bacteriuria, or of catheter usage. In contrast to these two branches, the *Lactobacillus* and *Streptococcus* branches are largely comprised of OTUs from healthy controls with a few exceptions (red leaves). One such exception was *Lactobacillus iners* (Figure 4), which was previously shown to significantly contribute to UTI [39,40]. Another exception was determined to be *Streptococcus salivarius*, a lactic acid-producing Gram-positive organism typically found in the oral cavity, and an opportunistic pathogen implicated in bacteremia [41-43] and septicemia [44]. 

### Phylogenetic diversity of the urinary enterobacteriales

**Figure 5:**
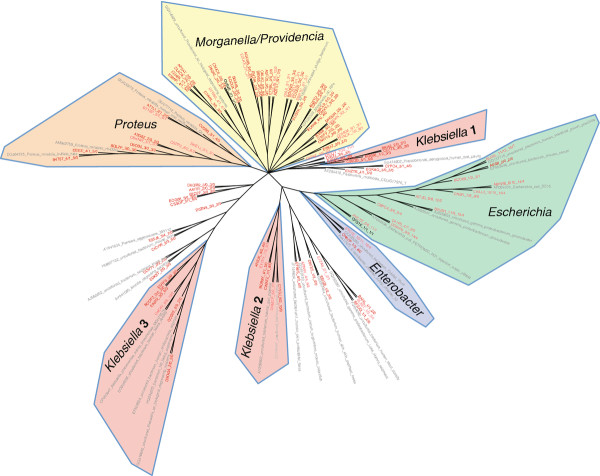
**Phylogenetic diversity of Enterobacteriales 16S rDNA sequences in human urine.** NJ tree clustering of Enterobacteriales OTU representatives labeled based on similarity to known RDP database sequences (gray), and OTU composition. Leaves are colored as follows: OTUs consisting of only healthy individuals (dark blue), mostly healthy (light blue), only NB (red), mostly NB (pink/salmon). Branches were highlighted and labeled by identifiable bacterial genera. Genus-level classification was based on the OTU representative RDP classification and the classification of nearest neighbors the RDP alignment. The nodes show SequenceID_#male/#female_#SCI/#healthy subjects.

In contrast to the Lactobacillales, the Enterobacteriales had no OTUs composed solely of healthy controls; indicating this group of bacteria may be a potential indicator of future UTI (Figure 5). Shaded regions of the tree noted unambiguous genus taxonomy as follows: *Escherichia, Enterobacter, Klebsiella, Proteus, Morganella*, and *Providencia*. All of these genera have been associated with UTI, and dominated the tree. *Klebsiella* stood out from the others, having been divided into three distinct branches. Top matches to N_2_-fixing, plant-associated *Klebsiella pneumoniae* and *K. variicola* isolates rather than known *K. pneumoniae* clinical strains suggests that N_2_-fixing, plant-associated *Klebsiella* may exist in urine. Further work is needed to confirm this result.

### Metaproteomics reveals anti-microbial and pro-inflammatory responses in the absence of diagnosed UTI

Shotgun proteomic data for a subset of nine urinary samples searching a protein sequence databases of 19 bacterial genomes were compared with the OTU analysis derived from 16S rDNA sequencing. In addition to protein identifications targeting the 19 species frequently associated with UTIs or urethra colonization, a non-redundant human protein database was searched to assess the detection of host responses towards bacterial colonization. While proteomic analysis identified more species than those diagnosed by culture methods, it did not identify the fastidious anaerobic or microaerophilic bacteria frequently assigned by the RDP classifier in the 16S rDNA analysis with the exception of *Lactobacillus*. We assume this to be due to species abundance issues. Proteins encoded by *Escherichia*, *Klebsiella* and *Enterobacter* species were identified in half of the examined samples, generally in agreement with 16S rDNA data (Table 2). Metaproteomic profiles allowed preliminary insights into the production of bacterial factors interacting with the urinary tract environment. Identifications of such proteins as limited to those urine donor samples with evidence for the initiation of pro-inflammatory and microbicidal host defenses (high spectral counts for calprotectin subunits; identification of lactotransferrin, myeloperoxidase and eosinophil peroxidase). We emphasize that the sample sizes are too small to predict whether these observations will be reproducible using larger-scale proteomic surveys. *Pseudomonas aeruginosa, Enterobacter hormaechei* and *E. coli* (known opportunistic pathogens in the urinary tract) produced proteins for iron/siderophore acquisition and high mobility (flagellins) in subjects 36 and 51 (Table 3). Flagellins are important for swarming and spread in the urogenital tract during infections [45]. The iron-sequestering protein lactotransferrin may be expressed and released into the urinary tract by neutrophils to sequester iron, which in turn, *E. coli* and *E. hormaechei* may counteract by addition of copious amounts of iron/siderophore receptors to the outer membrane protein repertoire (Table 3). While the S100-A8 and S100-A9 calprotectins appear to have numerous physiological functions, one of them is to sequester zinc in response to sensing the presence of bacterial pathogens. This sequestration can inactivate metalloproteinases that the bacteria produce and secrete to invade the host tissue [46]. Such proteases were not identified in our datasets. 

**Table 2:** Bacterial profiles of urinary samples

**Urine donor**	**Species (proteome)**	**Species (urine culture)**	Species (16S rRNA profile)*	**Leukocyte Esterase**	**WBC (no./hpf)**
1(HC)	-	-	*Lactobacillus*	NEG	0
16(HC)	Lj	*Lactobacillus* spp	*Lactobacillus*	NEG	0-1
31(void)	Ec, Eh, Kp	-	*Enterobacter, Enterococcus, Escherichia, Klebsiella, Lactobacillus, Streptococcus*	TRA	1-2
33(void)	-	Ec, Ef	*Enterobacter, Enterococcus, Escherichia, Klebsiella*	2+	5-9
34(void)	Ec, Eh, Kp	*Klebsiella oxytoca*	*Enterobacter, Escherichia, Lactobacillus, Streptococcus*	2+	5-9
36(IC)	Ec, Eh	Ec	*Enterobacter, Escherichia, Klebsiella, Lactobacillus, Streptococcus*	NEG	0-1
37(IC)	-	-	*Enterobacter, Enterococcus, Escherichia, Klebsiella, Lactobacillus, Pseudomonas, Streptococcus*	NEG	1-2
39(IC)	Ec, Kp	Kp	*Enterobacter, Klebsiella, Lactobacillus, Streptococcus*	NEG	3-4
45(FC)	Eh, Kp, Pa, Pm^#^	Ec, Ef, Kp, Pa, Ps	*Enterobacter, Enterococcus, Escherichia, Klebsiella, Lactobacillus, Proteus, Pseudomonas, Streptococcus*	NEG	0-1
51(FC)	Ec, Eh, Pa, Sp	Ec, Ef, Pa	*Enterobacter, Enterococcus, Escherichia, Klebsiella, Pseudomonas, Streptococcus*	2+	5-9

*Not comprehensive. 16S rDNA data is at the genus level.

^#^The species for this sample were determined from urine cultures, but based on shotgun proteomic analysis after protein extraction from colonies.

Ec *Escherichia coli,* Eh *Enterobacter hormaechei,* Ef *Enterococcus faecalis,* Kp *Klebsiella pneumoniae,* Pa *Pseudomonas aeruginosa,* Pm *Proteus mirabilis,* Ps *Providencia stuartii,* Sp *Streptococcus pneumoniae.*

WBC = white blood cells, HC = healthy control, IC = intermittent catheter, FC = Foley catheter, NEG = negative, TRA = trace.

**Table 3:** Human and bacterial proteins potentially contributing directly or indirectly to host-pathogen interactions in the urinary tract

**Human protein**	**Gene name**	**PSMs**	**Functional role**	**Urine donor**
Protein S100-A9 Calprotectin L1H subunit	S10A9	60	Pro-inflammatory, metal ion-chelating	1,16,33,36,51
Protein S100-A8, Calprotectin L1L subunit	S10A8	10	Pro-inflammatory, metal ion-chelating	1,16,36,51
Protein S100-A12	S10AC	14	Pro-inflammatory	36
Myeloperoxidase	PERM	13	Microbicidal	36,51
Eosinophil peroxidase	PERE	4	Microbicidal	51
Lactotransferrin	TRFL	6	Pro-inflammatory, iron-chelating	36
14-3-3 protein sigma	SFN	5	DNA damage response, cell proliferation	16
SNC66 protein	-	12	Secreted, Ig-like domain	51
Heat shock protein beta-1	HSPB1	20	Anti-inflammatory, cell proliferation	1
Annexin A2	ANXA2	8	Cell proliferation, cell adhesion	16
Uromodulin	UMOD	78	Cell protection, inhibitor of Ca crystallization,	1,16,31,36,37,39,51
Cystatin-B	CYTB	8	Immunomodulatory, cathepsin inhibitor	1,16
14-3-3 protein zeta/delta	YWHAZ	2	Adaptor protein, tyrosine phosphorylation pathways	1,16
Serpin B3	SPB3	7	Immunomodulatory, serine protease inhibitor	16
Small proline-rich protein 3	SPRR3	7	Cell repair and proliferation	16
Annexin A1	ANXA1	28	Anti-apoptotic, T-cell differen-tiation, signaling pathways	1,16,31,34,37,39
Glutathione S-transferase P	GSTP1	3	Anti-apoptotic, tyrosine phosphorylation pathways	1,16,31,34,37
**Bacterial protein**	Species^*^			
Colicin receptor CirA	Ec	2	Iron/colicin-binding	36
OM heme/hemoglobin receptor ChuA	Ec	12	Iron-binding	51
Putative pesticin receptor Psn	Ec	23	Iron-binding	51
Ferrienterobactin receptor FepA	Eh	9	Iron-binding	51
Putative fimbrillin MatB	Ec	2	Adhesion	51
Flagellin protein type B FliC	Pa	13	Mobility and adhesion	51
Flagellin protein FliC	Eh	8	Mobility and adhesion	51
Flagellin	Ec	7	Mobility and adhesion	51
Ferrienterobactin receptor FepA	Ec	22	Iron-binding	36, 51

Section one of the table lists proteins with potential pro/anti-inflammatory, immune-modulatory and microbicidal activities. Section two lists identified bacterial proteins implicated in virulence/survival or serving as a target for host defensive mechanisms.

PSMs: the highest number of peptide-spectral matches (PSMs) is provided for each listed protein * for species abbreviations, see Table 2 (minimal Mascot Percolator PEP value: 10^-4^).

Several other proteins implicated in the innate immune response were detected (Table 3). Calprotectin has acute and chronic pro-inflammatory functions and recruits immune cells to the site of inflammation [47]. Antimicrobial peroxidases, such as eosinophil and myeloperoxidase, produce reactive oxygen species during the respiratory burst of neutrophils and are directly microbicidal. Like calprotectin, annexin A1 is a calcium-binding protein that was detected in high abundance primarily in those donors where respiratory burst of neutrophils did seem to be muted (subjects 1, 16, 31, 34, 37, 39). These proteins are implicated in innate immunity, influence cell apoptosis and function as damage-associated molecular patterns (DAMPs) [48,49]. For a complete list of bacterial and human proteins profiled in this study, see Additional file 1.

## Discussion

In this study we describe the healthy urine microbiome in a heterogeneous population of men and women with and without NB, using both 16S rDNA sequencing and metaproteomic analysis. Based on other reports [50-53] and including our data, this is further confirmation that the commonly held clinical belief that healthy urine should be sterile is false. Specifically, our data indicate that (1) when collected by the routine method of “clean, midstream catch”, healthy urine is not aseptic; (2) the healthy and NB urine microbiomes differs by gender; (3) the asymptomatic bacteriuria urine microbiome of people with NB differs from that of healthy controls; and (4) the asymptomatic bacteriuria urine microbiome of people with NB differs depending on duration of exposure to and type of urinary catheter.

This is the first report comparing the healthy urine microbiome in male and female subjects. Historically, and based on cultivation results, clinicians have assumed urine to be ‘sterile’. However, Wolfe et al. recently used 16S rDNA sequencing to identify uncultivated bacteria in the urine of healthy women [53]. Our data confirm these results in women, and further show that uncultivated bacteria are also present in the urine of healthy men. Moreover, we demonstrate that the healthy urine microbiome of males and females differs, with men having significantly more relative abundance of *Corynebacterium*, a common component of the superficial skin flora, and women having significantly greater relative abundance of *Lactobacillus*. Critical to this discussion is an understanding that because all samples from healthy subjects and those of subjects with NB who voided were collected by midstream clean catch, it is not possible to distinguish whether the microbes identified originated in the bladder, urethra, or both. Therefore, the possibility exists that the identified urine microbiomes are populated by species that exist in the bladder, urethra, or both.

Our finding of predominance of *Corynebacterium* in healthy males suggests that this microbe may contribute to the healthy urine microbiome. Not only was *Corynebacterium* identified to a significantly greater degree in healthy males compared to those with NB, males with NB who voided and were sampled by clean catch had the lowest abundance of this species. Dong et al. compared clean catch urine and distal urethral swabs in healthy volunteers and similarly found a predominance of *Corynebacterium* in both types of sampling but in significantly greater amounts in the clean catch urine samples [51]. Taken together, the data suggest that *Corynebacterium* may reside in the proximal urethra and/or bladder in addition to the distal urethra, and may play a role in the healthy urine microbiome.

While it is well established that in most healthy women of childbearing age the vaginal tract is colonized by *Lactobacillus* species [54-56], this has not been investigated in women with NB. We too found a clear preponderance of *Lactobacillus* in healthy control females; however, in addition to the greatest relative abundance of *Lactobacillus* in healthy control females, there was a progressive reduction in abundance of *Lactobacillus* in females with NB who void (clean catch sample) or who use intermittent catheterization, and females with NB who use indwelling (Foley) catheters (Figure 3 inset). This may suggest that either increasing exposure to a urinary catheter and/or increasing severity of NB can influence the ability of *Lactobacillus* to colonize the urinary tract. Alternatively, *Lactobacillus* may merely be a contaminant of the external urethra that arises from proximity to the vaginal flora. However, this appears less likely since Wolfe et al. showed the presence of *Lactobacillus* in urine collected by transurethral catheters and suprapubic aspirate, which samples the bladder directly [53]. Because lactic acid-producing *Lactobacillus* species contribute to controlling the growth of more virulent bacteria that cannot survive in a more acidic environment, the presence of *Lactobacillus* within the urethra and/or bladder may be protective. This has been shown to be the case in infants [57] and may also be true for males as *Lactobacillus* has been shown to be present in clean catch urine samples of healthy males by Dong et al. [51] and in our study. Our findings suggest that *Lactobacillus* may be a commensal organism present during states of health, more in females than in males, and that the microbiome of at-risk populations may be characterized by a distinct lack of *Lactobacillus*, which perhaps creates a better environment for the growth of pathogenic microorganisms. Together, these findings suggest that the clinical objective of ‘sterile’, microbe-free urine may not be optimal for the patient.

This is the first report of 16S rDNA sequencing of urine in people with NB, providing much more detail about the ABU state than has previously been available through cultivation-based evidence. Standard cultivation diagnostics show populations vulnerable to bacteriuria include nursing home residents utilizing long-term indwelling catheterization [58], institutionalized Veterans utilizing intermittent catheterization [59], and patients with SCI who utilize urinary catheters [60-62]. Our analysis not only confirms the cultivation-based evidence, but also shows that a significantly different microbiome was present in the NB group, and that 16S rDNA sequencing specifically identifies microorganisms, such as *Enterobacteriales,* as potential major contributors to a pathogenic microbiome. These results supplement those from Bank et al., where urine specimens of 142 consecutive patients with varied genitourinary pathology (kidney stones, indwelling catheters, or suspected UTI) were analyzed with standard cultivation and screened for *Actinobaculum schaalii* using PCR [50]. Those authors found that the most heavily colonized patients were those who were older and who utilized indwelling urinary catheters, while the younger patients who typically use intermittent catheterization (and may have utilized urinary catheters for a shorter amount of time) were colonized with bacteria to a lesser degree. The *Enterobacteriaceae* was most commonly isolated in the catheterized specimens in that study. Significant variance in medical comorbidities, underlying genitourinary pathology, and method of urine collection limit any further comparisons to that study. Moreover, because our NB population was reportedly asymptomatic (i.e. infection free), our findings demonstrate that in the catheterized population, the microbiome is intrinsically different than in controls, even in the absence of illness. This distinction is important, since in the clinical setting ABU is often inappropriately treated with antibiotics, which may further disrupt the NB microbiome.

The present data also indicate that the urine microbiome of healthy subjects with NB became increasingly abundant with Enterobacteriales with increasing duration of NB, whereas *Lactobacillus* decreased over time*,* both in men and women. While the urinary microbiome of men and women with NB remained similar to that of healthy controls during the first several months after NB diagnosis, by one year the urine microbiome was nearly devoid of *Lactobacillus* and dominated by *Enterococcus*. This further suggests a change in the microbiome with duration of NB that may place patients at increased risk of UTI.

Fundamental to these discoveries is the diverse sample population and our novel analytic approach of utilizing a combination of 16S rDNA sequencing in all subjects and metaproteomics in a subsample. Clinical gold standard diagnostic testing when a patient presents with signs and/or symptoms of UTI includes (1) urinalysis to confirm urinary tract inflammation and (2) urine culture to identify, quantify and predict antimicrobial resistance to a given pathogen(s). We believe that 16S rDNA sequencing has the potential for translation to the clinic, offering significant clinical advancement over diagnostic urine culture because it provides a greater depth of understanding and sensitivity pertaining to the composition of commensal and potentially pathogenic microbes present in urine. Furthermore, prospective assessments during varying periods of health and disease may allow personalization of care that has not been possible to date with our current diagnostic methods. Urinary metaproteomic profiles in parallel may contribute to the identification of a host inflammatory response utilizing urinary biomarkers with greater sensitivity and specificity for UTI than traditional measures of urinary leukocyte esterase production or white blood cell count detected by urinalysis. We hypothesize that protein profiles with distinct abundance ratios of immunomodulatory versus pro-inflammatory and microbiocidal molecules, are indicative of either UTI or reflect asymptomatic colonization. For instance, the presence of lactotransferrin in urine has been used to support the notion of a “battle for iron” being waged between the host and the pathogen, involving *E. coli*, particularly in the case of UTI by *E. coli* given the abundance of its triad of iron acquisition receptors [63]. While metaproteomics holds promise as a diagnostic method to discriminate between symptomatic UTI and ABU, more in-depth fractionation of samples is needed to evaluate whether this method can reach the sensitivity of 16S rDNA profiling methods. Larger patient cohorts, including those diagnosed with UTI symptoms, are required to assess if such ‘omics methods’ more accurately differentiate UTI from ABU than current diagnostic standards. In addition, they suggest new considerations that may impact preventive and treatment options for people at-risk for UTI.

Several limitations of this study are to be considered when interpreting the results. The major limiting factor is that some subjects (healthy controls and NB subjects who voided spontaneously) had urine collection via midstream clean catch sampling while the subjects with NB who managed their bladder with intermittent catheterization or indwelling Foley (urethral) catheters were sampled directly from the catheter. Therefore, microbes identified in the former groups could be representatives from the bladder, urethra, skin, or a combination of these microbiomes, whereas urine from subjects in the latter groups represents bladder microbiota. This distinction raises other questions, such as to what degree do differing microbiomes in the urethra and bladder influence each other, and does a changing urethral microbiome represent a potential antecedent UTI state. Further, given that clinical urine sampling is unlikely to become more invasive (via direct sampling from the bladder), how do clinicians interpret the presence of bacteria in clean catch midstream urine samples that could potentially originate from multiple anatomic locations? Lastly, future studies will employ a larger sample size as our results indicate that the urine microbiome differs by a number of clinical factors requiring multiple stratification points.

## Conclusions

Contrary to clinical dogma that healthy urine is sterile; these results suggest that the state of healthy urine is, in fact, one of ‘asymptomatic bacteriuria’. Utilizing high throughput sequencing and metaproteomics, we have described the healthy urine microbiome of a number of populations: male and female healthy controls and healthy subjects with NB. Differing urinary microbiomes for males and females were described. We have demonstrated that NB and/or urinary catheterization impacts the healthy urine microbiome in both genders and this varies by type of bladder management and duration of NB. Furthermore, the presence of a variety of urine microbiomes differing on key, clinical characteristics suggests the benefit of a more personalized approach to UTI care. Clearly, DNA sequencing techniques allow for more specific assessment of the contributing microorganisms than do current clinical diagnostic standards, offering the potential for significant clinical advancement of diagnostic methods for UTI, which have otherwise remained relatively unchanged for decades. Longitudinal differentiation of the urine microbiome at the time of, prior to, and after infection also will be necessary to fully describe the course of disease and its antecedents. These findings advance clinical translational science toward improved diagnostics and more targeted use of therapeutics.

## Abbreviations

ABU: Asymptomatic Bacteriuria; CAUTI: Catheter Associated Urinary Tract Infections; CNMC: Children’s National Medical Center; CRAN: Comprehensive R Archive Network; CTSA: Clinical and Translational Science Awards; DAMPs: Damage-Associated Molecular Patterns; DHHS: Department of Health and Human Services; DNA: Deoxyribonucleic Acid; Ec: *Escherichia coli*; EDTA: Ethylenediaminetetraacetic Acid; Ef: *Enterococcus faecalis*; Eh: *Enterobacter hormaechei*; ESBL: Extended-Spectrum-Beta-Lactamases; FASP: Filter-Aided Sample Preparation; FC: Foley Catheter; HC: Healthy Control; Hpf: High power field; IC: Intermittent Catheter; IRB: Institutional Review Board; JCVI: J. Craig Venter Institute; Kp: *Klebsiella pneumoniae*; LCA: Least Common Ancestor; Lj: *Lactobacillus jensenii*; LTQ: Linear Trap Quadrupole; NB: Neuropathic Bladder; NCATS: National Center for Advancing Translational Sciences; NCBI: National Center for Biotechnology Informatio; NCMRR: National Center for Medical Rehabilitation Research; NCRR: National Center for Research Resources; NEG: Negative; NIH: National Institutes of Health; NINDS: National Institute for Neurological Disorders and Stroke; NJ: Neighbor-Joining; No: Number; NRH: National Rehabilitation Hospital; NT: Nucleotide; OGE: Oracle Grid Engine; OTU: Operational Taxonomic Unit; Pa: *Pseudomonas aeruginosa*; PBS: Phosphate Buffered Saline; PCA: Principal Component Analysis; PCR: Polymerase Chain Reaction; Pm: *Proteus mirabilis*; Ps: *Providencia stuartii*; RDP: Ribosomal Database Project; RNA: Ribonucleic Acid; rRNA: Ribosomal Ribonucleic Acid; SCI: Spinal Cord Injury; SDS-PAGE: Sodium Dodecyl Sulfate Polyacrylamide Gel Electrophoresis; Sp: *Streptococcus pneumoniae*; SSU: Small Subunit; TRA: Trace; UTI: Urinary Tract Infection; WBC: White Blood Cells; YAP: Yet Another Pipeline.

## Competing interests

The authors declare that they have no competing interests.

## Authors’ contributions

DEF, RP, SLG conceived and organized the study; MS, BS, SKL, MT performed laboratory experiments; DEF, RP, SS, HP, SK, MS, IL, BS, SLG processed and/or analyzed data; and DEF, RP, SS, HP, SK, MT, KEN, SLG wrote the manuscript. All authors read and approved the final manuscript.

## Supplementary Material

Additional file 1 Proteomic analyses of urinary pellet samples using the LTQ XL ion trap instrument (Thermo-Electron) and the Mascot search engine version 2.3 (Matrix Science) for spectral matches with a 19-species database. Click here for file

Additional file 2 YAP 16S rDNA sequence-processing and analysis pipeline workflow diagram. Click here for file

Additional file 3 A table summarizing the results of bacterial OTU-based microbiome analysis. Click here for file

Additional file 4 Bacterial OTU taxonomic profiles at the genus level per individual. Click here for file
