# Supplementary figures and images for: Integrated next-generation sequencing of 16S rDNA and metaproteomics differentiate the healthy urine microbiome from asymptomatic bacteriuria in neuropathic bladder associated with spinal cord injury
Source: J Transl Med. 2012 Aug 28;10:174. doi: 10.1186/1479-5876-10-174 (PMC3511201; doi:10.1186/1479-5876-10-174)

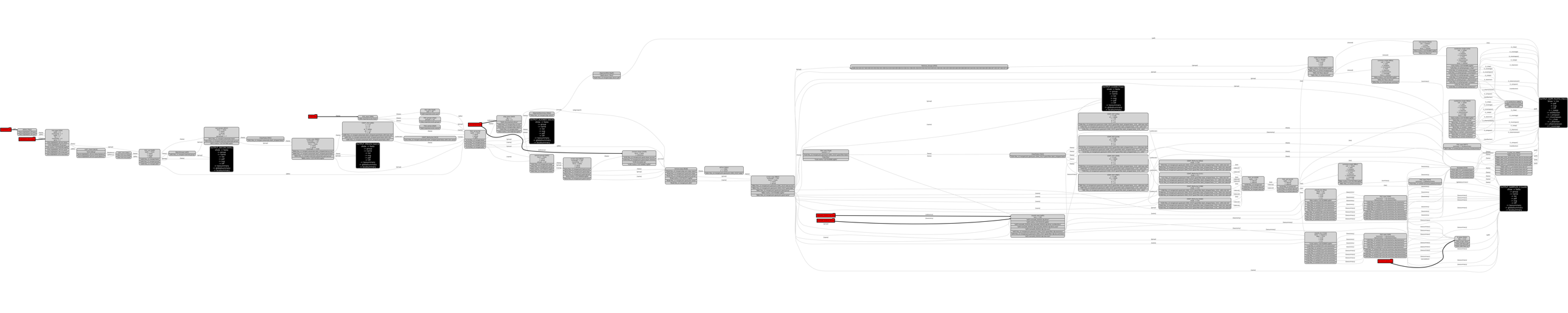

Supplement: Additional file 1 — Proteomic analyses of urinary pellet samples using the LTQ XL ion trap instrument (Thermo-Electron) and the Mascot search engine version 2.3 (Matrix Science) for spectral matches with a 19-species database. [file 1479-5876-10-174-S1.pdf]

# Relative OTU distribution

## Taxon: Genus

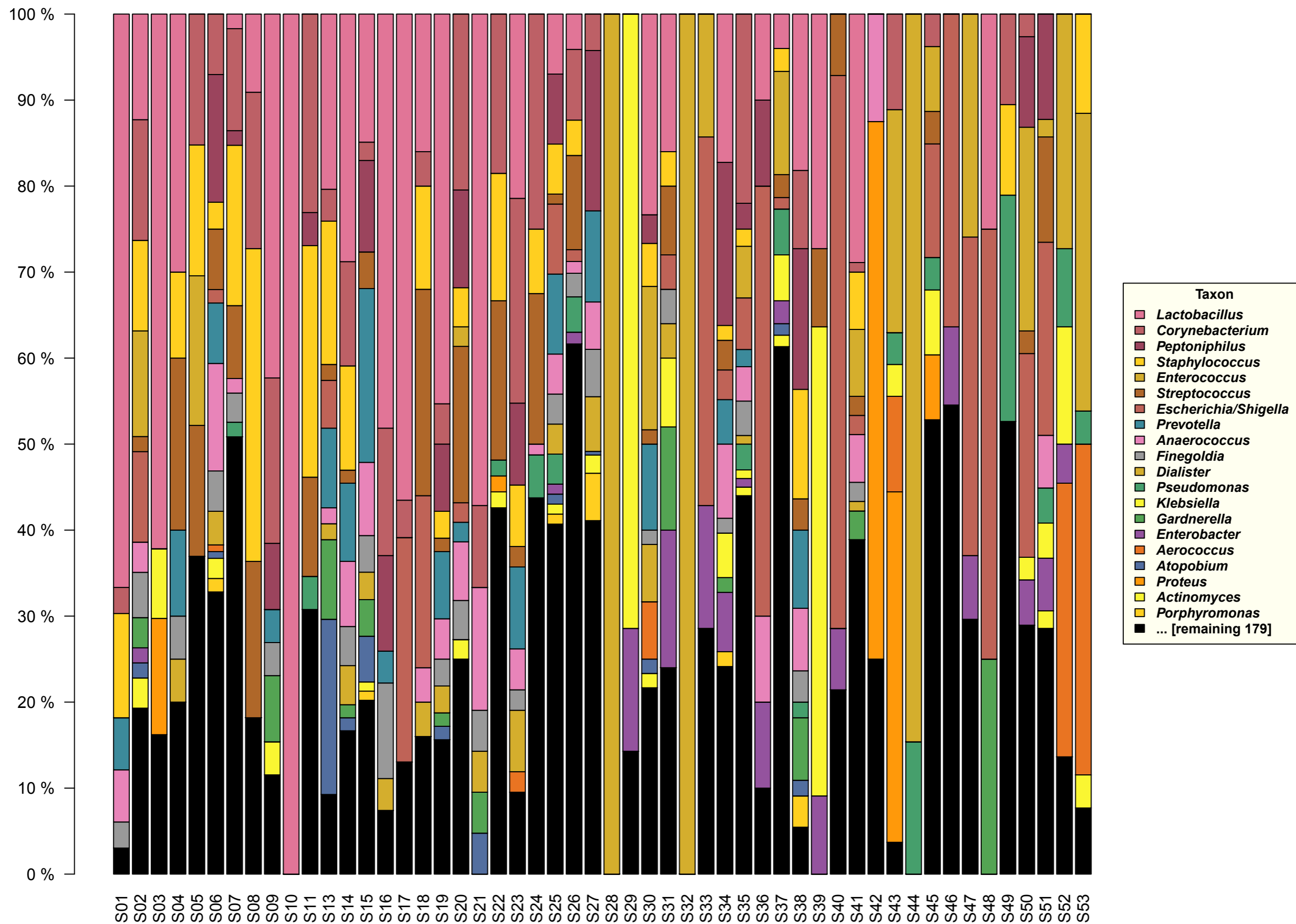

Supplement: Additional file 3 — A table summarizing the results of bacterial OTU-based microbiome analysis. [file 1479-5876-10-174-S3.pdf]
