# Supplementary material for: Integrated next-generation sequencing of 16S rDNA and metaproteomics differentiate the healthy urine microbiome from asymptomatic bacteriuria in neuropathic bladder associated with spinal cord injury
Source: J Transl Med. 2012 Aug 28;10:174. doi: 10.1186/1479-5876-10-174 (PMC3511201; doi:10.1186/1479-5876-10-174)
Supplement: Additional file 2 — YAP 16S rDNA sequence-processing and analysis pipeline workflow diagram. [file 1479-5876-10-174-S2.pdf]

Additional File 1. Summary of the Urinary Microbiome

Subject	Bladder Management	Distance	# of sequences	# of OTUs	Inv Simpson	Chao	Shannon	Shannon Evenness	Coverage
S01	HC	0.03	3360	33	2.07 (1.99, 2.15)	50.5 (38.12, 92.84)	1.2 (1.16, 1.25)	0.34	99.55%
S02	HC	0.03	3331	57	2.96 (2.85, 3.09)	112.2 (75.81, 218.98)	1.68 (1.62, 1.73)	0.41	99.28%
S03	HC	0.03	3310	37	3.21 (3.12, 3.31)	97 (53.65, 253.17)	1.46 (1.42, 1.5)	0.41	99.52%
S04	HC	0.03	3472	20	3.04 (2.97, 3.12)	38.33 (24.48, 95.08)	1.28 (1.25, 1.3)	0.43	99.68%
S05	HC	0.03	1931	46	4.51 (4.31, 4.72)	54.27 (48.35, 75.16)	1.95 (1.89, 2.01)	0.51	99.28%
S06	HC	0.03	3007	128	5.47 (5.14, 5.84)	239.24 (184.07, 348.67)	2.62 (2.56, 2.69)	0.54	97.94%
S07	HC	0.03	3412	59	6.66 (6.37, 6.97)	109.14 (77.47, 195.09)	2.32 (2.27, 2.36)	0.57	99.21%
S08	HC	0.03	3464	22	2.51 (2.43, 2.6)	29 (23.45, 55.75)	1.2 (1.17, 1.24)	0.39	99.77%
S09	HC	0.03	3494	26	1.55 (1.51, 1.6)	45.5 (31.22, 98.91)	0.77 (0.73, 0.81)	0.24	99.63%
S10	HC	0.03	3531	5	1.41 (1.38, 1.45)	5 (5, 5)	0.58 (0.54, 0.61)	0.36	100.00%
S11	HC	0.03	3499	26	1.8 (1.75, 1.85)	33.2 (27.6, 58.37)	0.88 (0.84, 0.92)	0.27	99.74%
S13	HC	0.03	3068	54	3.15 (2.99, 3.32)	101.25 (71.88, 178.88)	1.73 (1.68, 1.78)	0.43	99.09%
S14	HC	0.03	3374	66	2.2 (2.13, 2.27)	104.75 (81.73, 161.45)	1.21 (1.16, 1.26)	0.29	99.08%
S15	HC	0.03	2628	94	6.4 (6.06, 6.78)	176.5 (131.21, 276.92)	2.47 (2.41, 2.53)	0.54	98.29%
S16	HC	0.03	3476	27	2.99 (2.92, 3.07)	67 (38.32, 168.36)	1.29 (1.26, 1.32)	0.39	99.54%
S17	HC	0.03	3490	23	1.75 (1.7, 1.81)	32 (25, 63.45)	0.9 (0.85, 0.94)	0.29	99.74%
S18	HC	0.03	2435	25	2.74 (2.66, 2.82)	36 (27.74, 69.1)	1.25 (1.21, 1.29)	0.39	99.55%
S19	HC	0.03	3176	64	4.28 (4.11, 4.46)	77.57 (68.63, 103.75)	1.91 (1.86, 1.96)	0.46	99.37%
S20	HC	0.03	3461	44	3.63 (3.48, 3.78)	51.8 (46.13, 72.61)	1.79 (1.74, 1.83)	0.47	99.62%
S21	HC	0.03	3488	21	1.72 (1.67, 1.77)	30 (23, 61.45)	0.82 (0.78, 0.86)	0.27	99.74%
S22	HC	0.03	3460	54	1.91 (1.83, 1.99)	65.14 (57.06, 94.63)	1.41 (1.35, 1.48)	0.35	99.62%
S23	HC	0.03	3489	42	2.52 (2.46, 2.58)	63.08 (49.66, 100.02)	1.11 (1.08, 1.15)	0.30	99.34%
S24	HC	0.03	3209	80	7.51 (7.13, 7.93)	105.09 (89.24, 148.12)	2.65 (2.59, 2.7)	0.60	99.25%
S25	HC	0.03	3403	86	3.25 (3.11, 3.4)	113.08 (96.33, 156.98)	1.93 (1.87, 1.99)	0.43	99.24%
S26	HC	0.03	3345	73	6.75 (6.37, 7.18)	78 (74.22, 93.57)	2.61 (2.56, 2.66)	0.61	99.67%
S27	Void	0.03	2757	236	13.9 (12.77, 15.24)	458.68 (369.79, 606.62)	3.73 (3.66, 3.8)	0.68	95.72%
S28	Void	0.03	3505	8	2.02 (1.95, 2.09)	8 (8, 0)	1.01 (0.98, 1.05)	0.49	99.97%
S29	Void	0.03	3493	7	1.1 (1.09, 1.12)	10 (7.36, 31.78)	0.23 (0.2, 0.26)	0.12	99.91%
S30	Void	0.03	3176	60	6.03 (5.8, 6.27)	88.11 (70.06, 138.54)	2.19 (2.14, 2.23)	0.53	99.28%
S31	Void	0.03	3334	25	3.34 (3.24, 3.44)	38.75 (28.4, 80.55)	1.45 (1.42, 1.49)	0.45	99.67%
S32	Void	0.03	3502	8	1.83 (1.78, 1.9)	8 (8, 8)	0.95 (0.91, 0.99)	0.46	100.00%
S33	Void	0.03	3436	7	1.53 (1.49, 1.57)	8 (7.07, 20.79)	0.63 (0.6, 0.66)	0.33	99.94%
S34	Void	0.03	3401	58	5.75 (5.62, 5.88)	109.67 (78.42, 188.73)	1.95 (1.91, 1.98)	0.48	99.09%
S35	IC	0.03	3241	100	10.34 (9.84, 10.9)	117.65 (106.68, 146.64)	2.93 (2.88, 2.98)	0.64	99.23%
S36	IC	0.03	3463	10	1.98 (1.91, 2.05)	11 (10.09, 20.68)	0.97 (0.94, 1.01)	0.42	99.91%
S37	IC	0.03	3297	75	5.03 (4.75, 5.35)	90.3 (79.96, 122.16)	2.39 (2.34, 2.45)	0.55	99.45%
S38	IC	0.03	2589	55	2.43 (2.33, 2.54)	87.5 (67.26, 141.17)	1.42 (1.36, 1.48)	0.36	99.00%
S39	IC	0.03	3496	11	1.15 (1.14, 1.18)	12.5 (11.15, 26.08)	0.34 (0.31, 0.38)	0.14	99.91%
S40	IC	0.03	3412	14	2.5 (2.42, 2.59)	14 (14, 14)	1.23 (1.2, 1.27)	0.47	100.00%
S41	IC	0.03	3105	90	8.23 (7.87, 8.63)	138.46 (110.75, 203.18)	2.63 (2.58, 2.68)	0.58	98.84%
S42	IC	0.03	3464	8	1.03 (1.02, 1.04)	8 (8, 0)	0.09 (0.07, 0.11)	0.04	99.97%
S43	FC	0.03	3368	27	1.26 (1.23, 1.29)	33 (28.33, 54.1)	0.6 (0.55, 0.65)	0.18	99.73%
S44	FC	0.03	3440	13	2.36 (2.27, 2.46)	13.33 (13.02, 18.96)	1.26 (1.23, 1.3)	0.49	99.94%
S45	FC	0.03	3175	53	5.23 (4.99, 5.5)	80.2 (61.26, 142.53)	2.17 (2.12, 2.21)	0.55	99.46%
S46	FC	0.03	3487	11	2.88 (2.79, 2.97)	14 (11.39, 34)	1.25 (1.22, 1.27)	0.52	99.89%
S47	FC	0.03	3201	27	5.65 (5.41, 5.92)	42 (30.25, 96.26)	2.1 (2.07, 2.14)	0.64	99.81%
S48	FC	0.03	3420	8	1.09 (1.07, 1.1)	8.5 (8.03, 16.26)	0.22 (0.19, 0.24)	0.10	99.94%
S49	FC	0.03	3274	19	1.83 (1.77, 1.89)	20.2 (19.14, 29.37)	0.97 (0.93, 1.02)	0.33	99.88%
S50	FC	0.03	3140	38	5.57 (5.35, 5.8)	43.25 (39, 65.61)	2.12 (2.07, 2.16)	0.58	99.78%
S51	FC	0.03	2377	49	3.35 (3.18, 3.53)	199 (95.81, 529.69)	1.74 (1.68, 1.8)	0.45	98.95%
S52	FC	0.03	3422	22	1.65 (1.6, 1.7)	27.6 (23.16, 49.14)	0.9 (0.86, 0.95)	0.29	99.77%
S53	FC	0.03	3212	26	2.29 (2.23, 2.36)	35 (28, 66.45)	1.16 (1.11, 1.2)	0.35	99.72%

HC: Healthy Control, IC: Intermittent Catheter, FC: Foley Catheter
