# Supplementary material for: Integrated next-generation sequencing of 16S rDNA and metaproteomics differentiate the healthy urine microbiome from asymptomatic bacteriuria in neuropathic bladder associated with spinal cord injury
Source: J Transl Med. 2012 Aug 28;10:174. doi: 10.1186/1479-5876-10-174 (PMC3511201; doi:10.1186/1479-5876-10-174)
Supplement: Additional file 4 — Bacterial OTU taxonomic profiles at the genus level per individual. [file 1479-5876-10-174-S4.pdf]

Proteomic Data Tables (Additional File 4)

Metaproteomic analyses of urinary pellets using LC-nESI-ion trap MS/MS instruments (LTQ-XL and Velos Pro, Thermo Fisher Scientific) and the Mascot search engine version 2.3 (Matrix Science) for spectral data interpretation based on a selection of 19 bacterial and a human genome-wide protein sequence database.

There are 11 separate tables (worksheets) each presenting a protein summary report and a peptide summary report. These reports were derived from Mascot v2.3 analysis using the software Proteome Discoverer (Thermo Fisher Scientific). Each of the pairs of reports (11) represents analysis of one urinary sample (from two technical LC-MS/MS replicate analyses). First, we applied the MudPIT scoring approach for our database searches using Mascot v2.3.
A peptide filter with a Mascot significance threshold of $p < 0.05$ was applied. Second, to obtain a more robust false discovery rate on the peptide level, we used the Mascot Percolator option where machine learning is employed to recalculate the false peptide discovery rate. A stringent peptide confidence threshold in the Percolator analysis mode was set with a q-value (the minimal false discovery rate) <= 0.01. On the protein level, we filtered requiring a minimal number of two distinct peptides with the rank 1, or one distinct peptide only, if the peptide was ranked 1 and if the peptide's PEP threshold value (from the Percolator analysis) was 10e-4 or lower. The protein summary report designates the identified proteins (or protein groups based on Mascot's algorithm to define protein groups with high amino acid sequence similarity. The peptide summary report provides the sequences of all identified peptides (above the PEP threshold of 10e-4 if a single distinct peptide was identified for a given protein.

The UniRef90 database (http://www.uniprot.org/) was comprised of a selection of non-redundant human protein sequences.
Nineteen sequenced bacterial genomes representing species occasionally or frequently associated with bacteriuria UTIs, were also selected for database (downloaded from NCBI).
The total number of protein sequences searched was 283115. Of these, 61395 sequences were from the human UniRef90 dataset.
The bacterial databases are listed below:
1) Lactobacillus delbrueckii subsp. bulgaricus PB2003/044-T3-4 AEAT01000000 (http://www.ncbi.nlm.nih.gov/Taxonomy/Browser/wwwtax.cgi?id=784613)
2) Lactobacillus jensenii JV-V16 ACGQ02000000 (http://www.ncbi.nlm.nih.gov/Taxonomy/Browser/wwwtax.cgi?id=525329)
3) Lactobacillus gasseri JV-V03 ACGO02000000 (http://www.ncbi.nlm.nih.gov/Taxonomy/Browser/wwwtax.cgi?id=525326)
4) Corynebacterium urealyticum (http://www.ncbi.nlm.nih.gov/Taxonomy/Browser/wwwtax.cgi?mode=Info&id=504474&lvl=3&lin=f&keep=1&srchmode=1&unlock)
5) Escherichia coli UPEC (http://www.ncbi.nlm.nih.gov/Taxonomy/Browser/wwwtax.cgi?mode=Info&id=199310&lvl=3&lin=f&keep=1&srchmode=1&unlock)
6) Peptoniphilus asaccharolyticus (http://www.ncbi.nlm.nih.gov/Taxonomy/Browser/wwwtax.cgi?mode=Info&id=1258&lvl=3&lin=f&keep=1&srchmode=1&unlock)
7) Klebsiella pneumonia (http://www.ncbi.nlm.nih.gov/Taxonomy/Browser/wwwtax.cgi?mode=Info&id=507522&lvl=3&lin=f&keep=1&srchmode=1&unlock)
8) Streptococcus penumoniae (http://www.ncbi.nlm.nih.gov/Taxonomy/Browser/wwwtax.cgi?mode=Info&id=1313&lvl=3&lin=f&keep=1&srchmode=1&unlock)
9) Prevotella intermedia (http://www.ncbi.nlm.nih.gov/Taxonomy/Browser/wwwtax.cgi?mode=Info&id=28131&lvl=3&lin=f&keep=1&srchmode=1&unlock)
10) Anaerococcus vaginalis (http://www.ncbi.nlm.nih.gov/Taxonomy/Browser/wwwtax.cgi?mode=Info&id=33037&lvl=3&lin=f&keep=1&srchmode=1&unlock)
11) Staphylococcus epidermidis (http://www.ncbi.nlm.nih.gov/Taxonomy/Browser/wwwtax.cgi?mode=Info&id=1282&lvl=3&lin=f&keep=1&srchmode=1&unlock)
12) Proteus mirabilis (http://www.ncbi.nlm.nih.gov/Taxonomy/Browser/wwwtax.cgi?mode=Info&id=529507&lvl=3&lin=f&keep=1&srchmode=1&unlock)
13) Pseudomonas aeruginosa (http://www.ncbi.nlm.nih.gov/Taxonomy/Browser/wwwtax.cgi?mode=Info&id=208964&lvl=3&lin=f&keep=1&srchmode=1&unlock)
14) Finegoldia magna (http://www.ncbi.nlm.nih.gov/Taxonomy/Browser/wwwtax.cgi?mode=Info&id=1260&lvl=3&lin=f&keep=1&srchmode=1&unlock)
15) Enterococcus faecalis (http://www.ncbi.nlm.nih.gov/Taxonomy/Browser/wwwtax.cgi?mode=Info&id=1351&lvl=3&lin=f&keep=1&srchmode=1&unlock)
16) Enterococcus faecium (http://www.ncbi.nlm.nih.gov/Taxonomy/Browser/wwwtax.cgi?mode=Info&id=1352&lvl=3&lin=f&keep=1&srchmode=1&unlock)
17) Morganella morganii (http://www.ncbi.nlm.nih.gov/Taxonomy/Browser/wwwtax.cgi?mode=Info&id=582&lvl=3&lin=f&keep=1&srchmode=1&unlock)
18) Enterobacter hormaechei (http://www.ncbi.nlm.nih.gov/Taxonomy/Browser/wwwtax.cgi?mode=Info&id=158836&lvl=3&lin=f&keep=1&srchmode=1&unlock)
19) Ureaplasma urealyticum (http://www.ncbi.nlm.nih.gov/Taxonomy/Browser/wwwtax.cgi?mode=Info&id=2130&lvl=3&lin=f&keep=1&srchmode=1&unlock)

On the following pages, the number part of the sample identifier (line 1) represents the human subject number (with a peptide summary section following the protein summary section)

Protein Summary Report

Index	indicates the order of the list in which the table was exported from Proteome Discoverer
Prot_hit	indicates protein family grouping: a family member will represent multiple same-set proteins. All proteins in a same-set group are indistinguishable on the basis of the peptide match evidence.
Accession	gi| and Ref Seq ID
Description	the name of the protein that appears in the Accession column
Mascot score	Mascot incorporates a probability-based implementation of the Mowse algorithm (see http://www.matrixscience.com/search_form_select.html).
Coverage	the percentage of the protein sequence covered by identified peptides
# peptide	the number of significant distinct peptide sequences in the protein group
# PSM	the total number of identified peptide sequences (peptide spectrum matches) for the protein, including those redundantly identified
# AA	the sequence length of the protein
MW [kDa]	the calculated molecular weight of the protein (without considering PTMs) and ioselectric point of protein
calc. pI	the theoretically calculated isoelectric point, which is the pH at which a particular molecule carries no net electrical charge

Peptide Summary Report

Sequence	the sequence of amino acids that compose the peptide
Protein Descriptions	the proteins associated with the peptide. This description is taken from the FASTA file
# proteins	the number of identified proteins in the protein group of a master protein
# protein group	the number of protein groups in which this peptide is found
Protein Group Accessi	the master protein accession that contain the peptide
Modifications	type of modification
q-value	A q-value is the minimal false discovery rate at which the identification is considered correct (a Mascot Percolator analysis value)
PEP	the probability that the observed PSM is incorrect (a Mascot Percolator analysis value)
Charge	the charge state of the peptide
m/z [Da]	the mass-to-charge ratio of the precursor ion, in daltons
MH+ [Da]	the protonated monoisotopic mass of the peptides, in daltons
Delta Mass [Da]	the difference between the theoretical mass of the peptide and the experimental mass of the precursor ion, in daltons

2DLCSCI_37 Protein Summary

index	Prot_hit	Accession	Description	Mascot Score	Coverage	# Peptides	# PSMs	# AAs	MW [kDa]	calc. pI
1	1	P63261	Actin, cytoplasmic 2 n=1334 RepID=ACTG_HUMAN	430.28	32.53%	7	10	375	41.8	5.48
2	2	P35579	Myosin-9 n=67 Tax=Tetrapoda RepID=MYH9_HUMAN	129.54	1.63%	2	2	1960	226.4	5.6
3	3	P15508	Spectrin beta chain, erythrocyte n=14 Tax=Euarchontoglires RepID=SPTB1_MOUSE	119.09	1.27%	2	2	2128	245.1	5.33
4	4	P04264	Keratin, type II cytoskeletal 1 n=7 Tax=Eutheria RepID=K2C1_HUMAN	324.61	10.87%	2	3	644	66	8.12
5	5	P13645	Keratin, type I cytoskeletal 10 n=12 Tax=Eutheria RepID=K1C10_HUMAN	111.28	3.25%	1	1	584	58.8	5.21
6	6.1	P08727	Keratin, type I cytoskeletal 19 n=9 Tax=Eutheria RepID=K1C19_HUMAN	248.48	7.25%	2	4	400	44.1	5.14
7	6.2	B4DE59	cDNA FLJ60424, highly similar to Junction plakoglobin n=1 Tax=Homo sapiens RepID=B4DE59_HUMAN	248.48	5.15%	2	4	563	62.6	5.17
8	7	P62937	Peptidyl-prolyl cis-trans isomerase A n=98 Tax=Theria RepID=PPIA_HUMAN	191.2	16.97%	2	4	165	18	7.81
9	8	P11277	Spectrin beta chain, erythrocyte n=18 Tax=Eutheria RepID=SPTB1_HUMAN	207.73	2.99%	4	4	2137	246.3	5.27
10	9.1	E9PFJ3	Uncharacterized protein n=3 Tax=Simiiformes RepID=E9PFJ3_HUMAN	600.81	9.17%	6	15	578	63.3	5.31
11	9.2	P07911	Uromodulin n=2 Tax=Catarrhini RepID=UROM_HUMAN	600.81	8.28%	6	15	640	69.7	5.24
12	10	P68871	Hemoglobin subunit beta n=102 Tax=Primates RepID=HBB_HUMAN	2997.24	61.90%	10	66	147	16	7.28
13	11	E9PFT6	Uncharacterized protein n=2 Tax=Homo sapiens RepID=E9PFT6_HUMAN	657.72	27.66%	3	18	141	15.4	7.91
14	12	P00918	Carbonic anhydrase 2 n=8 Tax=Primates RepID=CAH2_HUMAN	185.59	15.77%	3	6	260	29.2	7.4
15	13	P69905	Hemoglobin subunit alpha n=29 Tax=Primates RepID=HBA_HUMAN	1236.61	39.44%	5	30	142	15.2	8.68
16	14	P02081	Hemoglobin fetal subunit beta n=8 Tax=Eutheria RepID=HBBF_BOVIN	247.92	14.48%	2	13	145	15.8	7.03
17	15	A4FUA1	GUCA1B protein (Fragment) n=1 Tax=Homo sapiens RepID=A4FUA1_HUMAN	336.59	58.67%	3	9	75	8.6	5.41
18	16.1	P62741	Hemoglobin subunit gamma-1 n=6 Tax=Hominidae RepID=HBG1_GORGO	161.29	15.65%	2	11	147	16.1	7.64
19	16.2	P69891	Hemoglobin subunit gamma-1 n=20 Tax=Catarrhini RepID=HBG1_HUMAN	161.29	15.65%	2	11	147	16.1	7.2
20	16.3	Q9GJS7	Hemoglobin subunit gamma n=39 Tax=Simiiformes RepID=HBG_CALJA	161.29	15.65%	2	11	147	16	6.93
21	17	P13646	Keratin, type I cytoskeletal 13 n=13 Tax=Simiiformes RepID=K1C13_HUMAN	606.2	22.27%	7	16	458	49.6	4.96
22	18.1	B4DRW1	cDNA FLJ55805, highly similar to Keratin, type II cytoskeletal 4 n=2 Tax=Hominidae RepID=B4DRW1_HUMAN	396.82	8.02%	3	11	474	51.7	6.81
23	18.2	P19013	Keratin, type II cytoskeletal 4 n=14 Tax=Simiiformes RepID=K2C4_HUMAN	396.82	7.12%	3	11	534	57.2	6.61
24	19	E7EUE8	Uncharacterized protein n=14 Tax=Eutheria RepID=E7EUE8_HUMAN	93.79	4.62%	2	3	520	55.8	6.27
25	19.1	P02538	Keratin, type II cytoskeletal 6A n=25 Tax=Euarchontoglires RepID=K2C6A_HUMAN	93.79	4.26%	2	3	564	60	8
26	19.2	F6RMN8	Uncharacterized protein n=4 Tax=Simiiformes RepID=F6RMN8_CALJA	93.79	4.49%	2	3	535	57.8	8.22
27	20.1	E9PF79	Pyruvate kinase n=4 Tax=Simiiformes RepID=E9PF79_HUMAN	82.19	6.33%	2	2	458	50	7.64
28	20.2	P14618	Pyruvate kinase isozymes M1/M2 n=16 Tax=Simiiformes RepID=KPYM_HUMAN	82.19	5.46%	2	2	531	57.9	7.84
29	20.3	B4DRT3	Pyruvate kinase n=2 Tax=Homo sapiens RepID=B4DRT3_HUMAN	82.19	5.68%	2	2	511	55.9	7.5
30	20.4	B4DUU6	Pyruvate kinase n=4 Tax=Simiiformes RepID=B4DUU6_HUMAN	82.19	5.62%	2	2	516	56.2	8.44
31	20.5	P52480	Pyruvate kinase isozymes M1/M2 n=40 Tax=Tetrapoda RepID=KPYM_MOUSE	82.19	5.46%	2	2	531	57.8	7.47
32	21	P05062	Fructose-bisphosphate aldolase B n=20 Tax=Eutheria RepID=ALDOB_HUMAN	90.46	4.12%	1	1	364	39.4	7.87
33	22	P02730	Band 3 anion transport protein n=4 Tax=Simiiformes RepID=B3AT_HUMAN	416.26	8.78%	6	10	911	101.7	5.19
34	23.1	P16157-15	Isoform Er14 of Ankyrin-1 n=9 Tax=Simiiformes RepID=P16157-15	122.97	1.59%	2	4	1821	200.1	6.92
35	23.2	P16157	Ankyrin-1 n=50 Tax=Mammalia RepID=ANK1_HUMAN	122.97	1.54%	2	4	1881	206.1	6.01
36	24	P02549	Spectrin alpha chain, erythrocyte n=16 Tax=Simiiformes RepID=SPTA1_HUMAN	567.72	3.97%	6	11	2419	279.8	5.05
37	25.1	E7ENH1	Uncharacterized protein n=6 Tax=Coelomata RepID=E7ENH1_HUMAN	103.15	10.70%	2	3	271	30.7	4.96
38	25.2	E7EWR1	Uncharacterized protein n=5 Tax=Simiiformes RepID=E7EWR1_HUMAN	103.15	8.22%	2	3	353	39.7	4.93
39	25.3	F7FYG3	Uncharacterized protein n=3 Tax=Simiiformes RepID=F7FYG3_CALJA	103.15	9.15%	2	3	317	35.9	4.88
40	25.4	Q5ST81	Tubulin beta polypeptide n=8 Tax=Eukaryota RepID=Q5ST81_HUMAN	103.15	7.80%	2	3	372	41.7	4.91
41	26.1	P06899	Histone H2B type 1-J n=102 Tax=Euteleostomi RepID=H2B1J_HUMAN	172.33	11.90%	1	2	126	13.9	10.32
42	26.2	P62807	Histone H2B type 1-C/E/F/G/I n=44 Tax=Euteleostomi RepID=H2B1C_HUMAN	172.33	11.90%	1	2	126	13.9	10.32
43	26.3	B4DR52	Histone H2B n=5 Tax=Euarchontoglires RepID=B4DR52_HUMAN	172.33	9.04%	1	2	166	18	10.32
44	26.4	P58876	Histone H2B type 1-D n=136 Tax=Coelomata RepID=H2B1D_HUMAN	172.33	11.90%	1	2	126	13.9	10.32
45	27.1	E9PEX0	Uncharacterized protein n=2 Tax=Homo sapiens RepID=E9PEX0_HUMAN	84.53	2.27%	1	1	704	78.4	5.59
46	27.2	E9PEW9	Uncharacterized protein n=3 Tax=Eutheria RepID=E9PEW9_HUMAN	84.53	2.09%	1	1	767	86	5.43
47	27.3	P11171	Protein 4.1 n=17 Tax=Eutheria RepID=41_HUMAN	84.53	1.85%	1	1	864	97	5.58
48	27.4	Q1WWM3	EPB41 protein (Fragment) n=6 Tax=Eutheria RepID=Q1WWM3_HUMAN	84.53	2.16%	1	1	742	83.2	5.72
49	27.5	Q6Q7P4	Protein 4.1 n=15 Tax=Eutheria RepID=41_CANFA	84.53	1.98%	1	1	810	90.6	5.55
50	28.1	UPI0000D48568	UPI0000D48568 related cluster n=1 Tax=Homo sapiens RepID=UPI0000D48568	149.43	8.60%	1	3	186	20.4	9.06
51	28.2	P04792	Heat shock protein beta-1 n=6 Tax=Simiiformes RepID=HSPB1_HUMAN	149.43	7.80%	1	3	205	22.8	6.4
52	29.1	A8MX94	Uncharacterized protein n=1 Tax=Homo sapiens RepID=A8MX94_HUMAN	202.44	9.20%	1	2	174	19.5	5.97
53	29.2	P09211	Glutathione S-transferase P n=4 Tax=Simiiformes RepID=GSTP1_HUMAN	202.44	7.62%	1	2	210	23.3	5.64
54	30.1	B4DUK1	cDNA FLJ51310, moderately similar to Peroxiredoxin-6 (EC 1.11.1.15) n=1 Tax=Homo sapiens RepID=B4DUK1_HUMAN	264.19	20.79%	1	4	101	11.2	5.39
55	30.2	P30041	Peroxiredoxin-6 n=17 Tax=Eutheria RepID=PRDX6_HUMAN	264.19	9.38%	1	4	224	25	6.38
56	31	P00915	Carbonic anhydrase 1 n=8 Tax=Catarrhini RepID=CAH1_HUMAN	353.05	27.97%	5	9	261	28.9	7.12
57	32.1	B4DY56	ATP synthase subunit alpha n=3 Tax=Eutheria RepID=B4DY56_HUMAN	74.41	4.71%	1	1	531	57.5	9.22
58	32.2	P25705	ATP synthase subunit alpha, mitochondrial n=53 Tax=Euteleostomi RepID=ATPA_HUMAN	74.41	4.52%	1	1	553	59.7	9.13
59	33.1	Q5T3N1	Annexin A1 (Fragment) n=3 Tax=Eutheria RepID=Q5T3N1_HUMAN	182.48	19.12%	2	3	204	22.7	5.53
60	33.2	P04083	Annexin A1 n=14 Tax=Eutheria RepID=ANXA1_HUMAN	182.48	11.27%	2	3	346	38.7	7.02
61	34.1	F7GZR9	Uncharacterized protein n=2 Tax=Simiiformes RepID=F7GZR9_CALJA	81.2	16.07%	1	1	112	12.5	6.6
62	34.2	P23528	Cofilin-1 n=29 Tax=Eutheria RepID=COF1_HUMAN	81.2	10.84%	1	1	166	18.5	8.09
63	35	P45974	Ubiquitin carboxyl-terminal hydrolase 5 n=20 Tax=Amniota RepID=UBP5_HUMAN	67.96	3.03%	1	1	858	95.7	5.03

2DLCSCI_37 Peptide Summary

Sequence	Protein Descriptions	# Proteins	# Protein Groups	Protein Group Accessions	Modifications	q-Value	PEP	Charge	m/z [Da]	MH+ [Da]	Delta Mass [Da]
FSVQMFR	Uncharacterized protein n=3 Tax=Simiiformes Rep	2	1	E9PFJ3		0.00774	0.215	2	458.12418	915.24108	0.39
FSVQMFR	Uncharacterized protein n=3 Tax=Simiiformes Rep	2	1	E9PFJ3		0.00774	0.0986	2	458.31796	915.62865	0.59
SAVTALWGK	Hemoglobin subunit beta n=102 Tax=Primates Rep	1	1	P68871		0	0.0117	2	467.02332	933.03935	0.26
SAVTALWGK	Hemoglobin subunit beta n=102 Tax=Primates Rep	1	1	P68871		0	0.00381	2	467.0636	933.11992	0.3
SAVTALWGK	Hemoglobin subunit beta n=102 Tax=Primates Rep	1	1	P68871		0	0.025	2	467.11771	933.22814	0.35
SAVTALWGK	Hemoglobin subunit beta n=102 Tax=Primates Rep	1	1	P68871		0	0.00112	2	467.11871	933.23015	0.36
VLDALQAIK	Carbonic anhydrase 1 n=8 Tax=Catarrhini RepID=C	1	1	P00915		0	0.00275	2	486.06573	971.12419	0.27
VLDALQAIK	Carbonic anhydrase 1 n=8 Tax=Catarrhini RepID=C	1	1	P00915		0	0.00563	2	486.49664	971.98601	0.7
AGFAGDDAPR	Actin, cytoplasmic 2 n=1334 RepID=ACTG_HUMAN	1	1	P63261		0	0.00163	2	488.9704	976.93352	0.24
TLDEYWR	Uncharacterized protein n=3 Tax=Simiiformes Rep	2	1	E9PFJ3		0.00774	0.108	2	491.944	982.88072	0.21
TLDEYWR	Uncharacterized protein n=3 Tax=Simiiformes Rep	2	1	E9PFJ3		0.00774	0.202	2	492.01202	983.01677	0.28
VVDVLDSIK	Carbonic anhydrase 2 n=8 Tax=Primates RepID=CA	1	1	P00918		0	0.0121	2	494.85309	988.6989	0.56
VGGTGMFTVR	Uncharacterized protein n=3 Tax=Simiiformes Rep	2	1	E9PFJ3		0	0.000223	2	513.03125	1025.05522	0.27
VGGTGMFTVR	Uncharacterized protein n=3 Tax=Simiiformes Rep	2	1	E9PFJ3		0	6.53E-05	2	513.09467	1025.18205	0.33
VGGTGMFTVR	Uncharacterized protein n=3 Tax=Simiiformes Rep	2	1	E9PFJ3		0	2.72E-05	2	513.30182	1025.59636	0.54
MFLSFPTTK	Hemoglobin subunit alpha n=29 Tax=Primates Rep	1	1	P69905		0.00295	0.0782	2	536.57184	1072.1364	0.29
MFLSFPTTK	Hemoglobin subunit alpha n=29 Tax=Primates Rep	1	1	P69905		0	0.0333	2	536.58313	1072.15898	0.3
MFLSFPTTK	Hemoglobin subunit alpha n=29 Tax=Primates Rep	1	1	P69905		0.00295	0.0367	2	536.58563	1072.16399	0.31
MFLSFPTTK	Hemoglobin subunit alpha n=29 Tax=Primates Rep	1	1	P69905		0.00295	0.073	2	536.78162	1072.55596	0.5
VDEVGGEALGR	Hemoglobin fetal subunit beta n=8 Tax=Eutheria F	1	1	P02081		0.00774	0.101	1	1101.78271	1101.78271	0.23
VDEVGGEALGR	Hemoglobin fetal subunit beta n=8 Tax=Eutheria F	1	1	P02081		0	0.000129	2	551.77966	1102.55205	0.5
VDEVGGEALGR	Hemoglobin fetal subunit beta n=8 Tax=Eutheria F	1	1	P02081		0	3.57E-05	2	551.82239	1102.6375	0.54
DAEEWFHAK	GUCA1B protein (Fragment) n=1 Tax=Homo sapien	1	1	A4FUA1		0	0.0318	2	566.99603	1132.98479	0.24
DAEEWFHAK	GUCA1B protein (Fragment) n=1 Tax=Homo sapien	1	1	A4FUA1		0.00295	0.0481	2	567.24713	1133.48699	0.49
DAEEWFHAK	GUCA1B protein (Fragment) n=1 Tax=Homo sapien	1	1	A4FUA1		0.00774	0.106	2	567.25293	1133.49858	0.5
VVAGVANALAHK	Hemoglobin subunit beta n=102 Tax=Primates Rep	1	1	P68871		0	0.00117	2	575.5379	1150.06853	0.2
VVAGVANALAHK	Hemoglobin subunit beta n=102 Tax=Primates Rep	1	1	P68871		0	0.00124	2	575.67053	1150.33379	0.33
VVAGVANALAHK	Hemoglobin subunit beta n=102 Tax=Primates Rep	1	1	P68871		0	0.00134	2	575.80438	1150.60149	0.46
SADFTNFDPR	Carbonic anhydrase 2 n=8 Tax=Primates RepID=CA	1	1	P00918		0	0.00259	2	585.51819	1170.0291	0.25
SADFTNFDPR	Carbonic anhydrase 2 n=8 Tax=Primates RepID=CA	1	1	P00918		0	0.0121	2	585.73602	1170.46477	0.47
VLSPADKTNVK	Hemoglobin subunit alpha n=29 Tax=Primates Rep	1	1	P69905		0	0.011	2	586.71106	1172.41484	0.37
VLSPADKTNVK	Hemoglobin subunit alpha n=29 Tax=Primates Rep	1	1	P69905		0	0.0197	2	586.91602	1172.82475	0.58
AVFPSIVGRPR	Actin, cytoplasmic 2 n=1334 RepID=ACTG_HUMAN	1	1	P63261		0	0.0127	2	600.00098	1198.99468	0.14
vLSPADKTNVK	Hemoglobin subunit alpha n=29 Tax=Primates Rep	1	1	P69905	N-Term(Acetyl)	0.00774	0.106	2	607.65448	1214.30168	0.31
VNVDAVGGEALGR	Uncharacterized protein n=2 Tax=Homo sapiens R	1	1	E9PFT6		0	1.70E-06	2	629.04871	1257.09014	0.22
VNVDAVGGEALGR	Uncharacterized protein n=2 Tax=Homo sapiens R	1	1	E9PFT6		0	4.78E-06	2	629.07703	1257.14678	0.24
VNVDAVGGEALGR	Uncharacterized protein n=2 Tax=Homo sapiens R	1	1	E9PFT6		0	1.06E-05	2	629.0791	1257.15093	0.25
VNVDAVGGEALGR	Uncharacterized protein n=2 Tax=Homo sapiens R	1	1	E9PFT6		0	3.57E-06	2	629.3208	1257.63432	0.49
DRLVPGPVFGSK	Uncharacterized protein n=1 Tax=Homo sapiens R	4	1	D6RIU4		0.00295	0.0415	2	636.69714	1272.38701	0.34
DRLVPGPVFGSK	Uncharacterized protein n=1 Tax=Homo sapiens R	4	1	D6RIU4		0.00774	0.156	2	636.8949	1272.78252	0.54
DRLVPGPVFGSK	Uncharacterized protein n=1 Tax=Homo sapiens R	4	1	D6RIU4		0.00774	0.116	2	637.01367	1273.02007	0.66
LLVVYPWTQR	Hemoglobin subunit beta n=102 Tax=Primates Rep	6	4	P68871;E9PFT6;P02081;P62741		0	0.0197	2	638.10645	1275.20561	0.24
LLVVYPWTQR	Hemoglobin subunit beta n=102 Tax=Primates Rep	6	4	P68871;E9PFT6;P02081;P62741		0	0.00643	2	638.14453	1275.28179	0.28
LLVVYPWTQR	Hemoglobin subunit beta n=102 Tax=Primates Rep	6	4	P68871;E9PFT6;P02081;P62741		0	0.0194	2	638.14917	1275.29106	0.28
LLVVYPWTQR	Hemoglobin subunit beta n=102 Tax=Primates Rep	6	4	P68871;E9PFT6;P02081;P62741		0	0.0163	2	638.16498	1275.32268	0.3
LLVVYPWTQR	Hemoglobin subunit beta n=102 Tax=Primates Rep	6	4	P68871;E9PFT6;P02081;P62741		0.00295	0.0345	2	638.16693	1275.32659	0.3
LLVVYPWTQR	Hemoglobin subunit beta n=102 Tax=Primates Rep	6	4	P68871;E9PFT6;P02081;P62741		0	0.0275	2	638.17517	1275.34307	0.31
LLVVYPWTQR	Hemoglobin subunit beta n=102 Tax=Primates Rep	6	4	P68871;E9PFT6;P02081;P62741		0	0.0178	2	638.18622	1275.36516	0.32
LLVVYPWTQR	Hemoglobin subunit beta n=102 Tax=Primates Rep	6	4	P68871;E9PFT6;P02081;P62741		0.00295	0.0385	2	638.20227	1275.39726	0.34
LLVVYPWTQR	Hemoglobin subunit beta n=102 Tax=Primates Rep	6	4	P68871;E9PFT6;P02081;P62741		0	0.00864	2	638.229	1275.45073	0.36
LLVVYPWTQR	Hemoglobin subunit beta n=102 Tax=Primates Rep	6	4	P68871;E9PFT6;P02081;P62741		0	0.0107	2	638.37225	1275.73723	0.51
ALEEANADLEVK	Keratin, type I cytoskeletal 13 n=13 Tax=Simiiform	1	1	P13646		0	0.0117	2	651.5575	1302.10771	0.22
VNVDEVGGEALGR	Hemoglobin subunit beta n=102 Tax=Primates Rep	1	1	P68871		0	0.0128	1	1314.70239	1314.70239	0.04
VNVDEVGGEALGR	Hemoglobin subunit beta n=102 Tax=Primates Rep	1	1	P68871		0.00295	0.0486	1	1314.7406	1314.7406	0.08
VNVDEVGGEALGR	Hemoglobin subunit beta n=102 Tax=Primates Rep	1	1	P68871		0	0.0065	2	657.88684	1314.76641	0.05
VNVDEVGGEALGR	Hemoglobin subunit beta n=102 Tax=Primates Rep	1	1	P68871		0	0.0237	1	1314.78088	1314.78088	0.12
VNVDEVGGEALGR	Hemoglobin subunit beta n=102 Tax=Primates Rep	1	1	P68871		0.00295	0.0343	1	1314.81848	1314.81848	0.15
VNVDEVGGEALGR	Hemoglobin subunit beta n=102 Tax=Primates Rep	1	1	P68871		0	0.000629	2	658.02026	1315.03325	0.18
VNVDEVGGEALGR	Hemoglobin subunit beta n=102 Tax=Primates Rep	1	1	P68871		0	0.00175	2	658.02246	1315.03765	0.19
VNVDEVGGEALGR	Hemoglobin subunit beta n=102 Tax=Primates Rep	1	1	P68871		0	0.00274	2	658.02917	1315.05107	0.19
VNVDEVGGEALGR	Hemoglobin subunit beta n=102 Tax=Primates Rep	1	1	P68871		0	0.00236	2	658.04175	1315.07622	0.21
VNVDEVGGEALGR	Hemoglobin subunit beta n=102 Tax=Primates Rep	1	1	P68871		0	0.000314	2	658.25146	1315.49565	0.42
VNVDEVGGEALGR	Hemoglobin subunit beta n=102 Tax=Primates Rep	1	1	P68871		0	0.0116	2	658.26868	1315.53008	0.43
VNVDEVGGEALGR	Hemoglobin subunit beta n=102 Tax=Primates Rep	1	1	P68871		0	0.017	2	658.27344	1315.5396	0.44
VNVDEVGGEALGR	Hemoglobin subunit beta n=102 Tax=Primates Rep	1	1	P68871		0	0.000536	2	658.32819	1315.6491	0.49
VNVDEVGGEALGR	Hemoglobin subunit beta n=102 Tax=Primates Rep	1	1	P68871		0	0.00197	2	658.33167	1315.65605	0.5
VNVDEVGGEALGR	Hemoglobin subunit beta n=102 Tax=Primates Rep	1	1	P68871		0	0.00413	2	658.36609	1315.7249	0.53
VNVDEVGGEALGR	Hemoglobin subunit beta n=102 Tax=Primates Rep	1	1	P68871		0	0.00205	2	658.40228	1315.79729	0.57
VNVDEVGGEALGR	Hemoglobin subunit beta n=102 Tax=Primates Rep	1	1	P68871		0	0.00958	2	658.40387	1315.80046	0.57
VNVDEVGGEALGR	Hemoglobin subunit beta n=102 Tax=Primates Rep	1	1	P68871		0	0.0244	2	658.42102	1315.83476	0.59
VNVDEVGGEALGR	Hemoglobin subunit beta n=102 Tax=Primates Rep	1	1	P68871		0	0.0339	2	658.44904	1315.89079	0.61
VNVDEVGGEALGR	Hemoglobin subunit beta n=102 Tax=Primates Rep	1	1	P68871		0.00295	0.0447	2	658.4585	1315.90972	0.62
VNVDEVGGEALGR	Hemoglobin subunit beta n=102 Tax=Primates Rep	1	1	P68871		0	0.000545	2	658.46265	1315.91802	0.63
VNVEDAGGETLGR	Hemoglobin subunit gamma-1 n=6 Tax=Hominidae	3	1	P62741		0	0.00409	2	659.35291	1317.69853	0.53
NLDLDSIIAEVK	Uncharacterized protein n=14 Tax=Eutheria RepID	3	1	E7EUE8		0.00295	0.0391	1	1329.74609	1329.74609	0.02
NLDLDSIIAEVK	Uncharacterized protein n=14 Tax=Eutheria RepID	3	1	E7EUE8		0.00295	0.0646	1	1329.79199	1329.79199	0.07
VVDLMAHMASKE	Glyceraldehyde-3-phosphate dehydrogenase n=3	2	1	E7EUT5		0	0.00031	2	665.91785	1330.82842	0.09
VVDLMAHMASKE	Glyceraldehyde-3-phosphate dehydrogenase n=3	2	1	E7EUT5		0	0.00047	2	666.27887	1331.55046	0.45
IITEGFEAAKEK	Uncharacterized protein n=5 Tax=Eutheria RepID=	2	1	A6NCD2		0.00774	0.132	2	668.75665	1336.50603	0.4
SKAEAEALYQTK	cDNA FLJ55805, highly similar to Keratin, type II cy	4	1	B4DRW1		0	0.000385	2	670.07642	1339.14556	0.23
SKAEAEALYQTK	cDNA FLJ55805, highly similar to Keratin, type II cy	4	1	B4DRW1		0	0.00159	2	670.07806	1339.14885	0.23
SKAEAEALYQTK	cDNA FLJ55805, highly similar to Keratin, type II cy	4	1	B4DRW1		0	0.000563	2	670.32971	1339.65215	0.48
SKAEAEALYQTK	cDNA FLJ55805, highly similar to Keratin, type II cy	4	1	B4DRW1		0	0.000468	2	670.35474	1339.7022	0.51
NLDLDSIIAEVR	cDNA FLJ55805, highly similar to Keratin, type II cy	2	1	B4DRW1		0	0.00107	1	1357.84387	1357.84387	0.11
NLDLDSIIAEVR	cDNA FLJ55805, highly similar to Keratin, type II cy	2	1	B4DRW1		0.00295	0.0356	1	1357.8584	1357.8584	0.13
NLDLDSIIAEVR	cDNA FLJ55805, highly similar to Keratin, type II cy	2	1	B4DRW1		0	0.0115	1	1357.86096	1357.86096	0.13
NLDLDSIIAEVR	cDNA FLJ55805, highly similar to Keratin, type II cy	2	1	B4DRW1		0	0.000893	1	1357.88586	1357.88586	0.15
NLDLDSIIAEVR	cDNA FLJ55805, highly similar to Keratin, type II cy	2	1	B4DRW1		0	0.000125	2	679.71692	1358.42656	0.35
TRLEQEIATYR	Keratin, type I cytoskeletal 13 n=13 Tax=Simiiform	1	1	P13646		0	0.00576	2	690.92004	1380.83281	0.55
AALEDTLAETEAR	Keratin, type I cytoskeletal 19 n=9 Tax=Eutheria Re	2	1	P08727		0	9.33E-05	2	695.82874	1390.65019	0.48
MIGFPSSAGSVSPR	Keratin, type I cytoskeletal 13 n=13 Tax=Simiiform	1	1	P13646		0	2.45E-06	2	696.96912	1392.93096	0.12
MIGFPSSAGSVSPR	Keratin, type I cytoskeletal 13 n=13 Tax=Simiiform	1	1	P13646		0	4.71E-06	2	697.06317	1393.11907	0.21
MIGFPSSAGSVSPR	Keratin, type I cytoskeletal 13 n=13 Tax=Simiiform	1	1	P13646		0	1.11E-05	2	697.2467	1393.48613	0.4
LEGLDTGWNALGR	Spectrin beta chain, erythrocyte n=18 Tax=Eutheri	1	1	P11277		0	7.16E-05	2	701.84119	1402.6751	0.48
ADTLTDEINFLR	Uncharacterized protein n=14 Tax=Eutheria RepID	3	1	E7EUE8		0	0.00995	2	704.90393	1408.80058	0.54
mIGFPSSAGSVSPR	Keratin, type I cytoskeletal 13 n=13 Tax=Simiiform	1	1	P13646	M1(Oxidation)	0	2.82E-06	2	705.08142	1409.15557	0.23
TALQPMVSALNIR	Uncharacterized protein n=3 Tax=Simiiformes Rep	2	1	E9PFJ3		0	0.00135	2	707.63013	1414.25298	0.23
TALQPMVSALNIR	Uncharacterized protein n=3 Tax=Simiiformes Rep	2	1	E9PFJ3		0	0.000894	2	707.66345	1414.31963	0.27
TALQPmVSALNIR	Uncharacterized protein n=3 Tax=Simiiformes Rep	2	1	E9PFJ3	M6(Oxidation)	0	0.00156	2	715.55286	1430.09844	0.16
TALQPmVSALNIR	Uncharacterized protein n=3 Tax=Simiiformes Rep	2	1	E9PFJ3	M6(Oxidation)	0	0.00455	2	715.82019	1430.6331	0.43
mDIAIHHPWIR	Uncharacterized protein n=18 Tax=Eutheria RepID	3	1	E9PJL7	N-Term(Acetyl)	0	0.00377	2	716.41718	1431.82707	0.55
HNLEHAFDVAER	Spectrin beta chain, erythrocyte n=14 Tax=Euarch	1	1	P15508		0	0.026	3	480.20398	1438.59739	0.3
VVAGVANALAHKYH	Hemoglobin subunit beta n=102 Tax=Primates Rep	1	1	P68871		0	0.000241	2	725.64856	1450.28984	0.25
VVAGVANALAHKYH	Hemoglobin subunit beta n=102 Tax=Primates Rep	1	1	P68871		0	4.42E-05	2	725.86377	1450.72026	0.46
VVAGVANALAHKYH	Hemoglobin subunit beta n=102 Tax=Primates Rep	1	1	P68871		0	0.000312	2	725.92517	1450.84307	0.52
IYVDDGLISLQVK	Pyruvate kinase n=4 Tax=Simiiformes RepID=E9PF	5	1	E9PF79		0	0.000113	2	732.45172	1463.89617	0.54
IPPDSEATLVLVGR	Band 3 anion transport protein n=4 Tax=Simiiform	1	1	P02730		0	7.44E-07	2	734.33447	1467.66167	0.42
GILAADESVGTMGNR	Fructose-bisphosphate aldolase B n=20 Tax=Euthe	1	1	P05062		0	4.19E-05	2	745.95844	1490.90959	0.09
ADFLEQPVLGFVR	Band 3 anion transport protein n=4 Tax=Simiiform	1	1	P02730		0	0.000927	2	746.42529	1491.84331	0.52
VKEGMNIVEAMER	Peptidyl-prolyl cis-trans isomerase A n=98 Tax=Th	1	1	P62937		0	2.67E-05	2	753.87347	1506.73967	0.5
VKEGMDIVEAMER	PREDICTED: peptidyl-prolyl cis-trans isomerase A-l	1	1	UPI000013CABC		0	0.000362	2	754.45203	1507.89678	0.58

Sequence	Protein Descriptions	# Proteins	# Protein Groups	Protein Group Accessions	Modifications	q-Value	PEP	Charge	m/z [Da]	MH+ [Da]	Delta Mass [Da]
DSSREPGGSLSFLR	Isoform Er14 of Ankyrin-1 n=9 Tax=Simiiformes Re	2	1	P16157-15		0	0.00122	2	754.77173	1508.53618	0.39
DSSREPGGSLSFLR	Isoform Er14 of Ankyrin-1 n=9 Tax=Simiiformes Re	2	1	P16157-15		0	0.00122	2	754.81958	1508.63188	0.44
FLQDYFDGNLKR	cDNA PSEC0148 fis, clone PLACE1007202, highly si	3	1	B3KQT2		0	0.0102	2	758.23474	1515.46221	-0.15
mDDREDLVYQAK	Uncharacterized protein n=3 Tax=Simiiformes Rep	2	1	F7GZ69	N-Term(Acetyl)	0.00774	0.119	2	763.04321	1525.07915	0.19
VGAHAGEYGAEALER	Hemoglobin subunit alpha n=29 Tax=Primates Rep	1	1	P69905		0	0.000288	1	1529.75647	1529.75647	0.02
VGAHAGEYGAEALER	Hemoglobin subunit alpha n=29 Tax=Primates Rep	1	1	P69905		0	9.21E-05	1	1529.76892	1529.76892	0.03
VGAHAGEYGAEALER	Hemoglobin subunit alpha n=29 Tax=Primates Rep	1	1	P69905		0	0.000543	1	1529.83289	1529.83289	0.1
VGAHAGEYGAEALER	Hemoglobin subunit alpha n=29 Tax=Primates Rep	1	1	P69905		0	0.000166	1	1529.85315	1529.85315	0.12
VGAHAGEYGAEALER	Hemoglobin subunit alpha n=29 Tax=Primates Rep	1	1	P69905		0	1.19E-05	2	765.61194	1530.2166	0.24
VGAHAGEYGAEALER	Hemoglobin subunit alpha n=29 Tax=Primates Rep	1	1	P69905		0	1.19E-05	2	765.72729	1530.44731	0.36
VGAHAGEYGAEALER	Hemoglobin subunit alpha n=29 Tax=Primates Rep	1	1	P69905		0	7.29E-06	2	765.75873	1530.51018	0.39
VGAHAGEYGAEALER	Hemoglobin subunit alpha n=29 Tax=Primates Rep	1	1	P69905		0	1.74E-06	2	765.77679	1530.54631	0.41
VGAHAGEYGAEALER	Hemoglobin subunit alpha n=29 Tax=Primates Rep	1	1	P69905		0	0.000318	2	765.79388	1530.58049	0.42
VGAHAGEYGAEALER	Hemoglobin subunit alpha n=29 Tax=Primates Rep	1	1	P69905		0	1.25E-06	2	765.82813	1530.64897	0.46
VGAHAGEYGAEALER	Hemoglobin subunit alpha n=29 Tax=Primates Rep	1	1	P69905		0	7.57E-06	2	765.83179	1530.6563	0.46
VGAHAGEYGAEALER	Hemoglobin subunit alpha n=29 Tax=Primates Rep	1	1	P69905		0	3.92E-05	2	765.89771	1530.78813	0.53
VGAHAGEYGAEALER	Hemoglobin subunit alpha n=29 Tax=Primates Rep	1	1	P69905		0	4.54E-06	2	765.99133	1530.97539	0.62
LLSGEDVGQDEGATR	Spectrin beta chain, erythrocyte n=14 Tax=Euarch	2	2	P15508;P11277		0	3.67E-05	2	774.21313	1547.41899	0.34
MELNEAWEDLQGR	Spectrin alpha chain, erythrocyte n=16 Tax=Simiif	1	1	P02549		0	0.000133	2	795.94476	1590.88225	0.08
MELNEAWEDLQGR	Spectrin alpha chain, erythrocyte n=16 Tax=Simiif	1	1	P02549		0	0.000125	2	795.95203	1590.89678	0.09
MELNEAWEDLQGR	Spectrin alpha chain, erythrocyte n=16 Tax=Simiif	1	1	P02549		0	9.30E-05	2	796.11151	1591.21575	0.25
MELNEAWEDLQGR	Spectrin alpha chain, erythrocyte n=16 Tax=Simiif	1	1	P02549		0	0.000112	2	796.16113	1591.31499	0.3
LSESHPDATEDLQR	Spectrin alpha chain, erythrocyte n=16 Tax=Simiif	1	1	P02549		0	1.97E-05	2	799.56555	1598.12383	0.19
YQSSPAKPDSSFYK	Band 3 anion transport protein n=4 Tax=Simiiform	1	1	P02730		0	3.48E-05	2	803.31482	1605.62236	0.43
YQSSPAKPDSSFYK	Band 3 anion transport protein n=4 Tax=Simiiform	1	1	P02730		0	4.48E-05	2	803.33203	1605.65679	0.45
ISEILLDHGAPIQAK	Isoform Er14 of Ankyrin-1 n=9 Tax=Simiiformes Re	2	1	P16157-15		0	0.000311	2	803.38538	1605.76348	0.43
ISEILLDHGAPIQAK	Isoform Er14 of Ankyrin-1 n=9 Tax=Simiiformes Re	2	1	P16157-15		0	0.00239	2	803.46045	1605.91362	0.51
TIEKFEKEAAEMGK	Uncharacterized protein n=5 Tax=Simiiformes Rep	5	1	E7EPH7		0	0.000291	2	806.40771	1611.80815	0.5
TIEKFEKEAAEMGK	Uncharacterized protein n=5 Tax=Simiiformes Rep	5	1	E7EPH7		0	0.00127	2	806.5014	1611.99553	0.59
LDLAGRDLTDYLMK	Actin, cytoplasmic 2 n=1334 RepID=ACTG_HUMAN	1	1	P63261		0	0.00145	2	812.63812	1624.26897	0.21
WNLLQQQTTTTSSK	cDNA FLJ55805, highly similar to Keratin, type II cy	2	1	B4DRW1		0	0.000666	2	818.59058	1636.17388	0.17
WNLLQQQTTTTSSK	cDNA FLJ55805, highly similar to Keratin, type II cy	2	1	B4DRW1		0	6.05E-05	2	818.79602	1636.58476	0.38
SDDKSSLDSLEALMK	Spectrin alpha chain, erythrocyte n=16 Tax=Simiif	1	1	P02549		0	8.85E-05	2	820.53845	1640.06963	0.64
DLAKDITSDTSGDFR	Annexin A1 (Fragment) n=3 Tax=Eutheria RepID=C	2	1	Q5T3N1		0	2.82E-05	2	821.276	1641.54473	0.38
DLAKDITSDTSGDFR	Annexin A1 (Fragment) n=3 Tax=Eutheria RepID=C	2	1	Q5T3N1		0	1.18E-06	2	821.35669	1641.7061	0.47
MVEEGHFAAEDVASR	Spectrin alpha chain, erythrocyte n=16 Tax=Simiif	1	1	P02549		0	3.49E-07	2	824.78906	1648.57085	0.41
MVEEGHFAAEDVASR	Spectrin alpha chain, erythrocyte n=16 Tax=Simiif	1	1	P02549		0	5.91E-07	2	824.87311	1648.73894	0.5
ALTVPELTQQVFDAK	Uncharacterized protein n=6 Tax=Coelomata RepI	4	1	E7ENH1		0	0.00964	2	830.94983	1660.89238	0.5
VLGAFSDGLAHLDNLK	Hemoglobin subunit beta n=102 Tax=Primates Rep	2	2	P68871;E9PFT6		0	6.79E-07	2	835.85126	1670.69524	0.4
VLGAFSDGLAHLDNLK	Hemoglobin subunit beta n=102 Tax=Primates Rep	2	2	P68871;E9PFT6		0	3.15E-07	2	835.89343	1670.77959	0.44
VLGAFSDGLAHLDNLK	Hemoglobin subunit beta n=102 Tax=Primates Rep	2	2	P68871;E9PFT6		0	3.29E-07	2	835.95331	1670.89934	0.5
VLGAFSDGLAHLDNLK	Hemoglobin subunit beta n=102 Tax=Primates Rep	2	2	P68871;E9PFT6		0	5.03E-05	2	835.96179	1670.91631	0.51
ALPFWNEEIVPQIK	Phosphoglycerate mutase (Fragment) n=4 Tax=Eut	7	1	B3EXF4		0	0.000741	2	842.94482	1684.88237	0.49
NSSYFVEWIPNNVK	Uncharacterized protein n=6 Tax=Coelomata RepI	4	1	E7ENH1		0	0.000115	2	849.24683	1697.48638	0.33
NSSYFVEWIPNNVK	Uncharacterized protein n=6 Tax=Coelomata RepI	4	1	E7ENH1		0	0.00149	2	849.32178	1697.63628	0.4
DSTIQVVENGESSQGR	Uncharacterized protein n=3 Tax=Simiiformes Rep	2	1	E9PFJ3		0	1.16E-05	2	853.63641	1706.26555	0.23
DSTIQVVENGESSQGR	Uncharacterized protein n=3 Tax=Simiiformes Rep	2	1	E9PFJ3		0	3.80E-06	2	853.84644	1706.68559	0.44
DSTIQVVENGESSQGR	Uncharacterized protein n=3 Tax=Simiiformes Rep	2	1	E9PFJ3		0	1.50E-05	2	853.86768	1706.72807	0.46
GSLGGGFSSGGFSGGSFSR	Keratin, type I cytoskeletal 10 n=12 Tax=Eutheria I	1	1	P13645		0	5.25E-08	2	854.83905	1708.67082	0.45
NLDIERPTYTNLNR	Uncharacterized protein n=8 Tax=Coelomata RepI	8	1	C9JDS9		0	0.029	2	860.60858	1720.20989	0.66
QLLQANPILEAFGNAK	Myosin-9 n=67 Tax=Tetrapoda RepID=MYH9_HUN	1	1	P35579		0	1.04E-06	2	864.18811	1727.36894	0.21
AMGIMNSFVNDIFER	Histone H2B type 1-J n=102 Tax=Euteleostomi Rep	4	1	P06899		0	5.79E-06	2	872.49829	1743.98931	0.09
AMGIMNSFVNDIFER	Histone H2B type 1-J n=102 Tax=Euteleostomi Rep	4	1	P06899		0	3.99E-06	2	872.81909	1744.63091	0.41
RYQSSPAKPDSSFYK	Band 3 anion transport protein n=4 Tax=Simiiform	1	1	P02730		0	7.52E-06	2	881.35901	1761.71074	0.43
RYQSSPAKPDSSFYK	Band 3 anion transport protein n=4 Tax=Simiiform	1	1	P02730		0	5.87E-06	2	881.44202	1761.87676	0.51
SMTPAQADLEFLENAK	Uncharacterized protein n=2 Tax=Homo sapiens R	5	1	E9PEX0		0	1.96E-05	2	883.35669	1765.7061	0.43
AQGLLSAGHPEGEQIIR	Spectrin beta chain, erythrocyte n=18 Tax=Eutheri	1	1	P11277		0	1.48E-05	2	888.91687	1776.82646	0.44
VSLDVNHFAPDELTVK	UPI0000D48568 related cluster n=1 Tax=Homo sap	2	1	UPI0000D48568		0	6.18E-05	2	892.87219	1784.73711	0.41
VSLDVNHFAPDELTVK	UPI0000D48568 related cluster n=1 Tax=Homo sap	2	1	UPI0000D48568		0	0.000233	2	892.88611	1784.76494	0.42
VSLDVNHFAPDELTVK	UPI0000D48568 related cluster n=1 Tax=Homo sap	2	1	UPI0000D48568		0	4.54E-05	2	892.94775	1784.88823	0.48
QSPASPERDYSPYYK	Keratin, type I cytoskeletal 13 n=13 Tax=Simiiform	1	1	P13646		0	0.000135	2	894.8761	1788.74492	0.46
SYELPDGQVITIGNER	Actin, cytoplasmic 2 n=1334 RepID=ACTG_HUMAN	1	1	P63261		0	3.80E-06	2	896.29297	1791.57866	0.34
FSSDFDELSGWMNEK	Spectrin alpha chain, erythrocyte n=16 Tax=Simiif	1	1	P02549		0	5.86E-06	2	896.69214	1792.377	0.31
FSSDFDELSGWMNEK	Spectrin alpha chain, erythrocyte n=16 Tax=Simiif	1	1	P02549		0	2.57E-06	2	896.92212	1792.83696	0.54
NIETIINTFHQYSVK	Protein S100-A9 n=5 Tax=Hominoidea RepID=S10	1	1	P06702		0	0.00214	2	904.46094	1807.9146	0.49
RFDEILEASDGIMVAR	Pyruvate kinase n=4 Tax=Simiiformes RepID=E9PF	5	1	E9PF79		0	0.00505	2	911.60339	1822.19951	0.14
VLAEMREQYEAMAER	Keratin, type I cytoskeletal 13 n=13 Tax=Simiiform	1	1	P13646		0.00295	0.0403	2	913.61963	1826.23198	0.19
VLAEMREQYEAMAER	Keratin, type I cytoskeletal 13 n=13 Tax=Simiiform	1	1	P13646		0	0.0168	2	913.84045	1826.67363	0.41
VLAEMREQYEAMAER	Keratin, type I cytoskeletal 13 n=13 Tax=Simiiform	1	1	P13646		0	0.0332	2	913.87537	1826.74346	0.44
SIYGEKFEDENFILK	Peptidyl-prolyl cis-trans isomerase A n=98 Tax=Th	1	1	P62937		0	0.000248	2	916.83142	1832.65557	0.37
SIYGEKFEDENFILK	Peptidyl-prolyl cis-trans isomerase A n=98 Tax=Th	1	1	P62937		0	3.06E-05	2	916.87463	1832.74199	0.42
SIYGEKFEDENFILK	Peptidyl-prolyl cis-trans isomerase A n=98 Tax=Th	1	1	P62937		0	1.17E-05	2	916.99243	1832.97759	0.53
TYFPHFDLSHGSAQVK	Hemoglobin subunit alpha n=29 Tax=Primates Rep	1	1	P69905		0	7.88E-05	2	917.63416	1834.26103	0.18
TYFPHFDLSHGSAQVK	Hemoglobin subunit alpha n=29 Tax=Primates Rep	1	1	P69905		0	1.90E-05	2	917.80701	1834.60674	0.36
TYFPHFDLSHGSAQVK	Hemoglobin subunit alpha n=29 Tax=Primates Rep	1	1	P69905		0	2.87E-05	2	917.83484	1834.6624	0.39
TYFPHFDLSHGSAQVK	Hemoglobin subunit alpha n=29 Tax=Primates Rep	1	1	P69905		0	6.29E-05	2	917.875	1834.74272	0.43
TYFPHFDLSHGSAQVK	Hemoglobin subunit alpha n=29 Tax=Primates Rep	1	1	P69905		0	2.55E-05	2	917.87854	1834.7498	0.43
TLQGLEIELQSQLSmK	Keratin, type I cytoskeletal 19 n=9 Tax=Eutheria R	4	3	P08727;A4FUA1;P13646	M15(Oxidation)	0	8.26E-07	2	917.90637	1834.80547	0.42
TLQGLEIELQSQLSmK	Keratin, type I cytoskeletal 19 n=9 Tax=Eutheria R	4	3	P08727;A4FUA1;P13646	M15(Oxidation)	0	1.16E-06	2	917.90894	1834.81059	0.42
TYFPHFDLSHGSAQVK	Hemoglobin subunit alpha n=29 Tax=Primates Rep	1	1	P69905		0	4.87E-05	2	917.948	1834.88872	0.5
TLQGLEIELQSQLSmK	Keratin, type I cytoskeletal 19 n=9 Tax=Eutheria R	4	3	P08727;A4FUA1;P13646	M15(Oxidation)	0	9.00E-06	2	918.01917	1835.03105	0.53
ANLQIDQINTDLNLER	Myosin-9 n=67 Tax=Tetrapoda RepID=MYH9_HUN	1	1	P35579		0	3.55E-05	2	935.58887	1870.17046	0.1
aSGVAVSDGVIKVFNDMK	Uncharacterized protein n=2 Tax=Simiiformes Rep	2	1	F7GZR9	N-Term(Acetyl)	0	3.72E-05	2	940.4231	1879.83891	0.44
FQDGDLTLYQSNTILR	Uncharacterized protein n=1 Tax=Homo sapiens R	2	1	A8MX94		0	6.10E-08	2	942.8064	1884.60552	0.33
FQDGDLTLYQSNTILR	Uncharacterized protein n=1 Tax=Homo sapiens R	2	1	A8MX94		0	9.68E-08	2	942.90039	1884.7935	0.42
VAPEEHPVLLTEAPLNPK	Actin, cytoplasmic 2 n=1334 RepID=ACTG_HUMAN	1	1	P63261		0	0.00222	2	977.9032	1954.79912	0.37
VAPEEHPVLLTEAPLNPK	Actin, cytoplasmic 2 n=1334 RepID=ACTG_HUMAN	1	1	P63261		0	1.69E-06	2	977.91406	1954.82085	0.38
VAPEEHPVLLTEAPLNPK	Actin, cytoplasmic 2 n=1334 RepID=ACTG_HUMAN	1	1	P63261		0	1.66E-06	2	977.92615	1954.84502	0.39
VAPEEHPVLLTEAPLNPK	Actin, cytoplasmic 2 n=1334 RepID=ACTG_HUMAN	1	1	P63261		0	2.71E-05	2	977.92993	1954.85259	0.39
HSHAGELEALGGVKPAVLTR	Band 3 anion transport protein n=4 Tax=Simiiform	1	1	P02730		0	9.84E-09	2	1021.83765	2042.66802	0.28
AAWGKVGAHAGEYGAEALER	Hemoglobin subunit alpha n=29 Tax=Primates Rep	1	1	P69905		0	4.40E-09	2	1022.41675	2043.82622	0.41
AAWGKVGAHAGEYGAEALER	Hemoglobin subunit alpha n=29 Tax=Primates Rep	1	1	P69905		0	1.37E-09	2	1022.42371	2043.84014	0.42
AAWGKVGAHAGEYGAEALER	Hemoglobin subunit alpha n=29 Tax=Primates Rep	1	1	P69905		0	5.96E-09	2	1022.44751	2043.88774	0.44
AAWGKVGAHAGEYGAEALER	Hemoglobin subunit alpha n=29 Tax=Primates Rep	1	1	P69905		0	3.99E-09	2	1022.66919	2044.3311	0.66
SAELNKEVSTNTAMIQTSK	GUCA1B protein (Fragment) n=1 Tax=Homo sapier	2	2	A4FUA1;P13646		0	1.84E-06	2	1026.84534	2052.6834	0.33
SAELNKEVSTNTAMIQTSK	GUCA1B protein (Fragment) n=1 Tax=Homo sapier	2	2	A4FUA1;P13646		0	4.18E-06	2	1026.92969	2052.8521	0.41
SAELNKEVSTNTAMIQTSK	GUCA1B protein (Fragment) n=1 Tax=Homo sapier	2	2	A4FUA1;P13646		0	5.43E-05	2	1027.06165	2053.11601	0.54
FFESFGDLSTPDAVMGNPK	Hemoglobin subunit beta n=102 Tax=Primates Rep	1	1	P68871		0	2.37E-05	2	1030.25854	2059.50981	0.28
FFESFGDLSTPDAVMGNPK	Hemoglobin subunit beta n=102 Tax=Primates Rep	1	1	P68871		0	0.000423	2	1030.28113	2059.55498	0.3
FFESFGDLSTPDAVMGNPK	Hemoglobin subunit beta n=102 Tax=Primates Rep	1	1	P68871		0	3.58E-06	2	1030.28918	2059.57109	0.31
FFESFGDLSTPDAVMGNPK	Hemoglobin subunit beta n=102 Tax=Primates Rep	1	1	P68871		0	8.35E-06	2	1030.34045	2059.67363	0.36
FFESFGDLSTPDAVMGNPK	Hemoglobin subunit beta n=102 Tax=Primates Rep	1	1	P68871		0	1.31E-05	2	1030.36926	2059.73125	0.39
FFESFGDLSTPDAVMGNPK	Hemoglobin subunit beta n=102 Tax=Primates Rep	1	1	P68871		0	2.09E-06	2	1030.3728	2059.73833	0.4
FFESFGDLSTPDAVMGNPK	Hemoglobin subunit beta n=102 Tax=Primates Rep	1	1	P68871		0	3.06E-06	2	1030.39551	2059.78374	0.42
FFESFGDLSTPDAVMGNPK	Hemoglobin subunit beta n=102 Tax=Primates Rep	1	1	P68871		0	4.18E-06	2	1030.41125	2059.81523	0.43
FFESFGDLSTPDAVMGNPK	Hemoglobin subunit beta n=102 Tax=Primates Rep	1	1	P68871		0	0.00098	2	1030.64563	2060.28398	0.67
FFESFGDLSTPDAVmGNPK	Hemoglobin subunit beta n=102 Tax=Primates Rep	1	1	P68871	M15(Oxidation)	0	1.77E-08	2	1037.802	2074.59673	-0.17
FFESFGDLSTPDAVmGNPK	Hemoglobin subunit beta n=102 Tax=Primates Rep	1	1	P68871	M15(Oxidation)	0	1.81E-08	2	1038.06128	2075.11528	0.09
FFESFGDLSTPDAVmGNPK	Hemoglobin subunit beta n=102 Tax=Primates Rep	1	1	P68871	M15(Oxidation)	0	7.30E-09	2	1038.34607	2075.68486	0.37
FFESFGDLSTPDAVmGNPK	Hemoglobin subunit beta n=102 Tax=Primates Rep	1	1	P68871	M15(Oxidation)	0	8.13E-07	2	1038.36523	2075.72319	0.39
FFESFGDLSTPDAVmGNPK	Hemoglobin subunit beta n=102 Tax=Primates Rep	1	1	P68871	M15(Oxidation)	0	5.74E-06	2	1038.37109	2075.73491	0.4
FFESFGDLSTPDAVmGNPK	Hemoglobin subunit beta n=102 Tax=Primates Rep	1	1	P68871	M15(Oxidation)	0	8.56E-06	2	1038.38037	2075.75347	0.41

Sequence	Protein Descriptions	# Proteins	# Protein Groups	Protein Group Accessions	Modifications	q-Value	PEP	Charge	m/z [Da]	MH+ [Da]	Delta Mass [Da]
FFESFGDLSTPDAVmGNPK	Hemoglobin subunit beta n=102 Tax=Primates Rep	1	1	P68871	M15(Oxidation)	0	0.000886	2	1038.41272	2075.81816	0.44
PGGLLLGDVAPNFEANTTVGR	cDNA FLJ51310, moderately similar to Peroxiredox	2	1	B4DUK1		0	1.59E-07	2	1049.62878	2098.25029	0.08
PGGLLLGDVAPNFEANTTVGR	cDNA FLJ51310, moderately similar to Peroxiredox	2	1	B4DUK1		0	6.52E-08	2	1049.92371	2098.84014	0.37
PGGLLLGDVAPNFEANTTVGR	cDNA FLJ51310, moderately similar to Peroxiredox	2	1	B4DUK1		0	9.22E-07	2	1049.94446	2098.88164	0.39
PGGLLLGDVAPNFEANTTVGR	cDNA FLJ51310, moderately similar to Peroxiredox	2	1	B4DUK1		0	3.19E-07	2	1049.96448	2098.92168	0.41
EAVEKEFEPLLNWMKDK	Endoplasmin n=58 Tax=Amniota RepID=ENPL_HUI	1	1	P14625		0	0.0127	3	703.05579	2107.15281	0.37
GLDAHLEQIFQEAHGMVAR	Spectrin beta chain, erythrocyte n=18 Tax=Eutheri	1	1	P11277		0	1.18E-05	3	708.08832	2122.2504	0.07
aSPDWGYDDKNGPEQWSK	Carbonic anhydrase 1 n=8 Tax=Catarrhini RepID=C	1	1	P00915	N-Term(Acetyl)	0	3.71E-05	2	1061.82056	2122.63384	0.36
aSPDWGYDDKNGPEQWSK	Carbonic anhydrase 1 n=8 Tax=Catarrhini RepID=C	1	1	P00915	N-Term(Acetyl)	0	1.23E-05	2	1061.86084	2122.7144	0.4
aSPDWGYDDKNGPEQWSK	Carbonic anhydrase 1 n=8 Tax=Catarrhini RepID=C	1	1	P00915	N-Term(Acetyl)	0	3.40E-05	2	1061.87134	2122.7354	0.41
aSPDWGYDDKNGPEQWSK	Carbonic anhydrase 1 n=8 Tax=Catarrhini RepID=C	1	1	P00915	N-Term(Acetyl)	0	3.54E-06	2	1062.09106	2123.17485	0.63
SAVTALWGKVNVDEVGGEALGR	Hemoglobin subunit beta n=102 Tax=Primates Rep	1	1	P68871		0	3.72E-08	2	1115.03845	2229.06963	0.45
SAVTALWGKVNVDEVGGEALGR	Hemoglobin subunit beta n=102 Tax=Primates Rep	1	1	P68871		0	4.86E-08	2	1115.03894	2229.0706	0.45
VYVELQELVMDEKNQELR	Band 3 anion transport protein n=4 Tax=Simiiform	1	1	P02730		0	0.000814	3	745.88623	2235.64414	0.17
VYVELQELVMDEKNQELR	Band 3 anion transport protein n=4 Tax=Simiiform	1	1	P02730		0	3.69E-06	2	1118.44128	2235.87529	0.37
VYVELQELVMDEKNQELR	Band 3 anion transport protein n=4 Tax=Simiiform	1	1	P02730		0	8.61E-06	2	1118.4801	2235.95293	0.41
EIINVGHSFHVNFEDNDNR	Carbonic anhydrase 1 n=8 Tax=Catarrhini RepID=C	1	1	P00915		0	6.35E-05	3	752.97333	2256.90543	0.29
FFESFGDLSTPDAVMGNPKVK	Hemoglobin subunit beta n=102 Tax=Primates Rep	1	1	P68871		0	7.34E-05	3	763.08923	2287.25315	0.38
GGPGSAVSPYPTFNPSSDVAALHK	Annexin A1 (Fragment) n=3 Tax=Eutheria RepID=C	2	1	Q5T3N1		0	6.53E-06	3	786.48773	2357.44864	0.43
GGGGGGYGSGGSSYGSGGGSYGSGGGGGGGR	Keratin, type II cytoskeletal 1 n=7 Tax=Eutheria Re	1	1	P04264		0	2.59E-09	2	1192.96936	2384.93144	0.49
ILNNGHAFNVEFDDSQDKAVLK	Carbonic anhydrase 2 n=8 Tax=Primates RepID=CA	1	1	P00918		0	6.70E-06	3	825.69489	2475.0701	0.28
ILNNGHAFNVEFDDSQDKAVLK	Carbonic anhydrase 2 n=8 Tax=Primates RepID=CA	1	1	P00918		0	5.62E-07	3	825.83325	2475.4852	0.42
ILNNGHAFNVEFDDSQDKAVLK	Carbonic anhydrase 2 n=8 Tax=Primates RepID=CA	1	1	P00918		0	3.44E-06	3	825.85559	2475.55222	0.44
HQGVmVGMGQKDSYVGDEAQSKR	Actin, cytoplasmic 2 n=1334 RepID=ACTG_HUMAN	1	1	P63261	M5(Oxidation)	0	5.67E-08	3	842.12415	2524.35788	0.4
ILGADTSVDLEETGRVLSIGDGIAR	ATP synthase subunit alpha n=3 Tax=Eutheria Rep	2	1	B4DY56		0	5.79E-06	3	853.54382	2558.61692	0.42
DSTIQVVENGESSQGRFSVQMFR	Uncharacterized protein n=3 Tax=Simiiformes Rep	2	1	E9PFJ3		0	0.000505	3	868.14307	2602.41465	0.39
SLLSNVEGDNAVPMQHNNRPTQPLK	Carbonic anhydrase 1 n=8 Tax=Catarrhini RepID=C	1	1	P00915		0	0.000696	3	920.85193	2760.54123	0.38
GVSEETLKEFSTIYKHFDENLTGR	Spectrin alpha chain, erythrocyte n=16 Tax=Simiifc	1	1	P02549		0	2.67E-05	3	934.50952	2801.51401	0.38
IGEWELIQESGVPLKPLFGPGYTGIR	Ubiquitin carboxyl-terminal hydrolase 5 n=20 Tax=	1	1	P45974		0	5.98E-05	3	953.14478	2857.41977	0.3
SLLSNVEGDNAVPMQHNNRPTQPLKGR	Carbonic anhydrase 1 n=8 Tax=Catarrhini RepID=C	1	1	P00915		0	1.08E-07	3	991.93237	2973.78257	0.42
VHLTPEEKSAVTALWGKVNVDEVGGEALGR	Hemoglobin subunit beta n=102 Tax=Primates Rep	1	1	P68871		0	5.99E-09	3	1054.90686	3162.70603	0.35
VHLTPEEKSAVTALWGKVNVDEVGGEALGR	Hemoglobin subunit beta n=102 Tax=Primates Rep	1	1	P68871		0	2.21E-08	3	1054.91101	3162.71848	0.35
TTGIVMDSGDGVTHTVPIYEGYALPHAILR	Actin, cytoplasmic 2 n=1334 RepID=ACTG_HUMAN	1	1	P63261		0	5.28E-08	3	1062.20667	3184.60544	0.33
GSYGSGGSSYGSGGGSYGSGGGGGGHGSYGSGSSSGGYR	Keratin, type II cytoskeletal 1 n=7 Tax=Eutheria Re	1	1	P04264		0	3.36E-08	3	1105.04285	3313.11399	0.27
GSYGSGGSSYGSGGGSYGSGGGGGGHGSYGSGSSSGGYR	Keratin, type II cytoskeletal 1 n=7 Tax=Eutheria Re	1	1	P04264		0	4.13E-07	3	1105.1947	3313.56955	0.42

2DLCSCI_31 Protein Summary

index	Prot_hit	Accession	Description	Mascot Score	Coverage	# Peptides	# PSMs	# AAs	MW [kDa]	calc. pI
1	1.1	334126439	translation elongation factor Tu [Enterobacter hormaeche	620.27	25.00%	7	18	376	41.1	5.24
2	1.2	206581075	translation elongation factor Tu [Klebsiella pneumoniae 3	620.27	23.86%	7	18	394	43.2	5.53
3	2	334125929	elongation factor G [Enterobacter hormaechei ATCC 4916	219.67	8.81%	4	4	704	77.5	5.34
4	3	334125399	enolase [Enterobacter hormaechei ATCC 49162]	355.04	12.04%	4	6	432	45.6	5.31
5	4	334124376	DNA-binding protein VicH [Enterobacter hormaechei ATC	82.49	10.22%	1	1	137	15.6	5.16
6	5	334123809	chaperone GroEL [Enterobacter hormaechei ATCC 49162]	930.79	28.47%	12	21	548	57.1	4.94
7	6	334122625	formate acetyltransferase [Enterobacter hormaechei ATC	224.84	7.76%	4	7	760	85	6.09
8	7.1	334122578	outer membrane protein A [Enterobacter hormaechei AT	104.29	3.35%	2	3	358	38.3	5.24
9	7.2	197284675	outer membrane protein A [Proteus mirabilis HI4320]	104.29	3.31%	2	3	362	39	5.85
10	8.1	206579217	ribosomal protein L2 [Klebsiella pneumoniae 342]	210.65	13.19%	3	5	273	29.8	10.93
11	8.2	334125920	50S ribosomal protein L2 [Enterobacter hormaechei ATCC	210.65	13.19%	3	5	273	29.8	10.93
12	9.1	206578965	ribosomal protein L29 [Klebsiella pneumoniae 342]	61.43	34.92%	1	1	63	7.2	10.27
13	9.2	334125915	50S ribosomal protein L29 [Enterobacter hormaechei ATC	61.43	34.92%	1	1	63	7.2	10.59
14	10	206576984	outer membrane protein TolC [Klebsiella pneumoniae 342	157.23	7.74%	3	4	491	53.3	6.19
15	11.1	206578717	fructose-bisphosphate aldolase [Klebsiella pneumoniae 34	68.59	11.42%	2	2	359	39.2	5.96
16	11.2	334125530	fructose-bisphosphate aldolase [Enterobacter hormaeche	68.59	11.42%	2	2	359	39.2	5.86
17	12	206580560	autonomous glycyl radical cofactor [Klebsiella pneumonia	75.57	31.50%	2	2	127	14.2	5.02
18	13	206576322	colicin I receptor [Klebsiella pneumoniae 342]	134.82	2.44%	1	2	657	73.1	5.41
19	14.1	206580610	aldehyde-alcohol dehydrogenase [Klebsiella pneumoniae	396.76	8.64%	4	6	891	95.9	6.79
20	14.2	26247570	bifunctional acetaldehyde-CoA/alcohol dehydrogenase [E	396.76	8.64%	4	6	891	96.1	6.79
21	15	206576961	quinate dehydrogenase (pyrroloquinoline-quinone) [Kleb	80.25	2.41%	1	1	790	85	7.09
22	16.1	206577927	NADP-specific glutamate dehydrogenase [Klebsiella pneu	281.24	11.86%	3	5	447	48.5	7.01
23	16.2	334122327	glutamate dehydrogenase [Enterobacter hormaechei ATC	281.24	11.86%	3	5	447	48.3	6.62
24	17	206580183	glyceraldehyde-3-phosphate dehydrogenase (phosphoryl	185.13	15.11%	3	5	331	35.5	7.11
25	18.1	206577508	outer membrane protein A [Klebsiella pneumoniae 342]	127.72	4.00%	1	2	350	37.4	6.25
26	18.2	26246978	outer membrane protein A [Escherichia coli CFT073]	127.72	3.69%	1	2	379	41	6.71
27	19	206577432	formate acetyltransferase [Klebsiella pneumoniae 342]	338.73	10.00%	5	9	760	85.2	5.85
28	20.1	206576199	alkyl hydroperoxide reductase subunit F [Klebsiella pneun	87.51	4.61%	1	1	521	56	5.59
29	20.2	334122925	alkyl hydroperoxide reductase [Enterobacter hormaechei	87.51	4.61%	1	1	521	55.8	5.5
30	21.1	206577586	alkyl hydroperoxide reductase subunit C [Klebsiella pneur	132.91	17.11%	2	3	187	20.8	5.17
31	21.2	334122926	alkyl hydroperoxide reductase C [Enterobacter hormaech	132.91	15.92%	2	3	201	22.4	5.02
32	21.3	26246586	alkyl hydroperoxide reductase subunit C [Escherichia coli	132.91	17.11%	2	3	187	20.7	5.17
33	24	206576800	antioxidant, AhpC/TSA family [Klebsiella pneumoniae 342	118.49	12.50%	2	3	200	22.3	5.29
34	25	206577768	dihydrolipoyllysine-residue acetyltransferase [Klebsiella p	161.94	2.70%	1	2	630	65.7	5.31
35	26	206579361	ribosomal protein S6 [Klebsiella pneumoniae 342]	109.96	14.50%	1	1	131	15.1	5.38
36	27.1	206580695	DNA-binding protein HU, alpha subunit [Klebsiella pneum	164.67	16.67%	1	2	90	9.5	9.58
37	27.2	334123929	DNA-binding protein HU-beta [Enterobacter hormaechei	164.67	16.67%	1	2	90	9.5	9.58
38	28.1	206578905	DNA-directed RNA polymerase, beta' subunit [Klebsiella p	187.86	2.63%	2	3	1407	155.3	6.8
39	28.2	26250759	DNA-directed RNA polymerase subunit beta' [Escherichia	187.86	2.63%	2	3	1407	155.1	7.08
40	29.1	206579488	DNA-directed RNA polymerase, beta subunit [Klebsiella p	214.34	4.25%	3	4	1342	150.4	5.29
41	29.2	334123945	DNA-directed RNA polymerase subunit beta [Enterobacte	214.34	4.21%	3	4	1353	151.6	5.27
42	29.3	26250758	DNA-directed RNA polymerase subunit beta [Escherichia	214.34	4.21%	3	4	1353	151.8	5.27
43	30.1	206577086	ribosomal protein L10 [Klebsiella pneumoniae 342]	96.04	17.58%	2	2	165	17.8	8.98
44	30.2	334123947	50S ribosomal protein L10 [Enterobacter hormaechei ATC	96.04	17.58%	2	2	165	17.8	8.98
45	30.3	26250755	50S ribosomal protein L10 [Escherichia coli CFT073]	96.04	17.58%	2	2	165	17.7	8.98
46	31.1	206575827	ATP synthase F0, B subunit [Klebsiella pneumoniae 342]	230.53	9.62%	1	4	156	17.4	5.8
47	31.2	334121919	ATP synthase F0 sector subunit B [Enterobacter hormaecl	230.53	9.62%	1	4	156	17.4	5.8
48	32.1	206577341	ATP synthase F1, beta subunit [Klebsiella pneumoniae 34	227.21	13.48%	5	6	460	50.2	5.03
49	32.2	334121923	ATP synthase F1 sector beta subunit [Enterobacter horma	227.21	13.48%	5	6	460	50.2	5
50	33.1	197285060	NAD(P) transhydrogenase subunit alpha [Proteus mirabili	169.17	3.34%	1	2	509	54.4	6.44
51	33.2	334122109	NAD(P) transhydrogenase subunit alpha [Enterobacter hc	169.17	3.19%	1	2	533	56.8	6.28
52	33.3	206576623	NAD(P) transhydrogenase, alpha subunit [Klebsiella pneu	169.17	3.31%	1	2	514	54.6	6.71
53	33.4	26247850	NAD(P) transhydrogenase subunit alpha [Escherichia coli	169.17	3.22%	1	2	528	56.4	5.85
54	34	197286390	60 Kda chaperonin [Proteus mirabilis HI4320]	143.37	6.20%	2	4	548	57.6	4.89
55	35.1	197286887	F0F1 ATP synthase subunit alpha [Proteus mirabilis HI432	208.79	7.80%	3	4	513	55.5	5.69
56	35.2	206580919	ATP synthase F1, alpha subunit [Klebsiella pneumoniae 34	208.79	7.80%	3	4	513	55.1	6
57	35.3	26250478	F0F1 ATP synthase subunit alpha [Escherichia coli CFT073	208.79	7.80%	3	4	513	55.2	6.13
58	36.1	26246451	transcriptional regulator HU subunit beta [Escherichia col	95.57	16.67%	1	1	90	9.2	9.7
59	36.2	334123164	DNA-binding protein HU-beta [Enterobacter hormaechei	95.57	16.67%	1	1	90	9.1	9.77
60	37.1	26246936	30S ribosomal protein S1 [Escherichia coli CFT073]	106.29	7.54%	2	3	557	61.1	4.98
61	37.2	334122618	30S ribosomal protein S1 [Enterobacter hormaechei ATCC	106.29	7.54%	2	3	557	61.2	4.98
62	38.1	26247927	major outer membrane lipoprotein [Escherichia coli CFT0	176.06	30.77%	2	4	78	8.3	9.25
63	38.2	334122238	major outer membrane lipoprotein [Enterobacter hormae	176.06	30.77%	2	4	78	8.4	9.31
64	39	26248038	glyceraldehyde-3-phosphate dehydrogenase [Escherichia	123.37	8.38%	2	3	334	35.9	6.8
65	40	26249182	phosphopyruvate hydratase [Escherichia coli CFT073]	417.5	13.19%	4	8	432	45.6	5.48
66	41.1	26249880	DNA-directed RNA polymerase subunit alpha [Escherichia	242.37	18.54%	4	6	329	36.5	5.06
67	41.2	334125900	DNA-directed RNA polymerase subunit alpha [Enterobact	242.37	18.54%	4	6	329	36.4	5.06

index	Prot_hit	Accession	Description	Mascot Score	Coverage	# Peptides	# PSMs	# AAs	MW [kDa]	calc. pI
68	42.1	161486053	ketol-acid reductoisomerase [Escherichia coli CFT073]	78.13	6.92%	2	3	491	54	5.31
69	42.2	334121775	ketol-acid reductoisomerase [Enterobacter hormaechei A	78.13	6.92%	2	3	491	54.1	5.25
70	42.3	206575721	ketol-acid reductoisomerase [Klebsiella pneumoniae 342]	78.13	6.92%	2	3	491	54	5.38
71	43	26251035	chaperonin GroEL [Escherichia coli CFT073]	574.68	19.53%	9	15	548	57.3	4.94
72	44	P04264	Keratin, type II cytoskeletal 1 n=7 Tax=Eutheria RepID=K2	213.64	7.45%	4	6	644	66	8.12
73	45.1	E9PFJ3	Uncharacterized protein n=3 Tax=Simiiformes RepID=E9P	378.23	5.36%	2	5	578	63.3	5.31
74	45.2	P07911	Uromodulin n=2 Tax=Catarrhini RepID=UROM_HUMAN	378.23	4.84%	2	5	640	69.7	5.24
75	45.3	Q5R5C1	Uromodulin n=8 Tax=Simiiformes RepID=UROM_PONAB	378.23	4.84%	2	5	641	69.8	5.24
76	46.1	B4DW52	Uncharacterized protein n=12 Tax=Coelomata RepID=B4D	129.13	17.29%	3	3	347	38.6	5.35
77	46.2	P63261	Actin, cytoplasmic 2 n=1334 RepID=ACTG_HUMAN	129.13	16.00%	3	3	375	41.8	5.48
78	47.1	P13646	Keratin, type I cytoskeletal 13 n=13 Tax=Simiiformes RepI	857.7	29.91%	8	21	458	49.6	4.96
79	47.2	P08727	Keratin, type I cytoskeletal 19 n=9 Tax=Eutheria RepID=K1	356.14	6.25%	2	6	400	44.1	5.14
80	48.1	B4DRW1	cDNA FLJ55805, highly similar to Keratin, type II cytoskele	647.41	21.73%	9	20	474	51.7	6.81
81	48.2	P19013	Keratin, type II cytoskeletal 4 n=14 Tax=Simiiformes RepID	647.41	19.29%	9	20	534	57.2	6.61
82	49	F6RMN8	Uncharacterized protein n=4 Tax=Simiiformes RepID=F6R	479.04	14.95%	8	17	535	57.8	8.22
83	50	P04792	Heat shock protein beta-1 n=6 Tax=Simiiformes RepID=HS	101.04	16.10%	2	2	205	22.8	6.4
84	51.1	A8MX94	Uncharacterized protein n=1 Tax=Homo sapiens RepID=A	180.75	9.20%	1	2	174	19.5	5.97
85	51.2	P09211	Glutathione S-transferase P n=4 Tax=Simiiformes RepID=(	180.75	7.62%	1	2	210	23.3	5.64
86	52	E7EU87	Uncharacterized protein n=3 Tax=Simiiformes RepID=E7E	265.42	10.99%	6	10	555	58.9	5.97
87	52.2	P13647	Keratin, type II cytoskeletal 5 n=17 Tax=Eutheria RepID=K	265.42	10.34%	6	10	590	62.3	7.74
88	53	P04083	Annexin A1 n=14 Tax=Eutheria RepID=ANXA1_HUMAN	742.81	38.44%	10	17	346	38.7	7.02

2DLCSCI_31 Peptide Summary

Sequence	Protein Descriptions	# Proteins	# Protein Groups	Protein Group Accessions	Modifications	q-Value	PEP	Charge	m/z [Da]	MH+ [Da]	Delta Mass [Da]
VGLFGGAGVGK	ATP synthase F1, beta subunit [Klebsiella pne	2	1	206577341		0	0.118	2	481.90405	962.80083	0.63
ITMQNLNDR	Keratin, type I cytoskeletal 13 n=13 Tax=Simii	2	1	P13646		0	0.0184	2	552.97864	1104.95	0.2
AQYEEIAQR	cDNA FLJ55805, highly similar to Keratin, type	4	2	B4DRW1;F6RMN8		0	0.0317	2	554.67041	1108.33354	0.4
AGIALNDNFVK	glyceraldehyde-3-phosphate dehydrogenase	2	2	206580183;26248038		0	0.00895	1	1161.70154	1161.70154	0.08
YEELQVTAGR	Uncharacterized protein n=4 Tax=Simiiforme	1	1	F6RMN8		0	0.000489	2	583.46655	1165.92583	0.17
LIAEAMDKVGK	chaperone GroEL [Enterobacter hormaechei	2	2	334123809;26251035		0	0.0152	2	588.13733	1175.26738	0.31
WTLLQEQGTK	Uncharacterized protein n=3 Tax=Simiiforme	3	2	E7EU87;F6RMN8		0	0.0228	2	602.95068	1204.89409	0.63
AQSVVDYLVSK	outer membrane protein A [Enterobacter hor	2	1	334122578		0	0.0119	2	605.09082	1209.17436	0.26
NDLDNAVEGLR	outer membrane protein TolC [Klebsiella pne	1	1	206576984		0	0.0196	2	608.62878	1216.25029	0.33
MGSEVFHNLAK	enolase [Enterobacter hormaechei ATCC 491	1	1	334125399		0	0.00571	2	617.1073	1233.20732	0.3
HQVVNDLPLGR	antioxidant, AhpC/TSA family [Klebsiella pneu	1	1	206576800		0	0.0127	2	624.83337	1248.65947	0.49
TPAQFDADELR	Annexin A1 n=14 Tax=Eutheria RepID=ANXA1	1	1	P04083		0	0.000904	2	632.27112	1263.53496	0.47
SLDLDSIIAEVK	Keratin, type II cytoskeletal 1 n=7 Tax=Euther	1	1	P04264		0	0.00715	1	1302.78418	1302.78418	0.07
IVGLQTEAPLKR	formate acetyltransferase [Enterobacter horr	2	2	334122625;206577432		0	0.0159	2	663.3783	1325.74932	0.48
NLDLDSIIAEVK	Keratin, type II cytoskeletal 2 epidermal n=7	4	2	E7EU87;F6RMN8		0	0.00152	1	1329.79602	1329.79602	0.07
SLTEIKDVLASR	DNA-directed RNA polymerase subunit alpha	2	1	26249880		0	7.05E-05	2	666.52478	1332.04228	0.14
DLEHPIEVPVGK	ATP synthase F1, beta subunit [Klebsiella pne	2	1	206577341		0	0.03	2	667.21667	1333.42607	0.36
TAAENDFVVLKK	cDNA FLJ55805, highly similar to Keratin, type	2	1	B4DRW1		0	0.000377	2	668.34143	1335.67558	0.47
DSGLDISYALRK	ketol-acid reductoisomerase [Escherichia coli	3	1	161486053		0	0.0683	2	669.89038	1338.77349	0.53
SKAEAEALYQTK	cDNA FLJ55805, highly similar to Keratin, type	4	1	B4DRW1		0	0.000451	2	670.27698	1339.54668	0.43
QSVDQPVQTGYK	F0F1 ATP synthase subunit alpha [Proteus mi	3	1	197286887		0	0.00511	2	675.5094	1350.01152	0.17
NLDLDSIIAEVR	cDNA FLJ55805, highly similar to Keratin, type	2	1	B4DRW1		0	0.00326	2	679.94019	1358.87309	0.57
NKLEGLEDALQK	Uncharacterized protein n=4 Tax=Simiiforme	1	1	F6RMN8		0	0.00186	2	679.75854	1358.50981	0.39
VDQLSNDVNAMR	major outer membrane lipoprotein [Escherich	2	1	26247927		0	0.00182	1	1361.68347	1361.68347	0.04
RAQSVVDYLVSK	outer membrane protein A [Enterobacter hor	2	1	334122578		0	0.000734	2	683.34564	1365.68401	0.47
LGVLGFEVDHER	acetate kinase [Klebsiella pneumoniae 342]	3	1	206580316		0	0.00567	2	686.41785	1371.82842	0.56
AGDQIQSGVDAAIK	ribosomal protein L2 [Klebsiella pneumoniae	2	1	206579217		0	0.000122	2	687.20319	1373.3991	0.35
AFDQIDNAPEEK	translation elongation factor Tu [Enterobacte	2	1	334126439		0	0.000971	1	1376.6582	1376.6582	0.03
SMQDPIADMLTR	30S ribosomal protein S8 [Pseudomonas aeru	5	1	15599445		0	0.00107	2	689.58838	1378.16948	0.26
GAAQNIIPSSTGAAK	glyceraldehyde-3-phosphate dehydrogenase	1	1	206580183		0	0.000829	2	693.82904	1386.6508	0.46
NKLAELEEALQK	Uncharacterized protein n=3 Tax=Simiiforme	2	1	E7EU87		0	0.00152	2	693.91962	1386.83196	0.53
LFAGNATPELAQR	ribose-phosphate pyrophosphokinase [Protei	4	1	197284954		0	0.109	2	694.82397	1388.64067	0.45
MQGSVTEFLKPR	DNA-directed RNA polymerase subunit alpha	2	1	26249880		0	0.000631	2	697.4104	1393.81352	0.54
YNYLINELNIK	outer membrane protein TolC [Klebsiella pne	1	1	206576984		0	0.00278	2	699.06433	1397.12139	0.19
VDSLNDEINFLK	cDNA FLJ55805, highly similar to Keratin, type	2	1	B4DRW1		0	0.00134	2	704.16187	1407.31645	0.3
mIGFPSSAGSVSPR	Keratin, type I cytoskeletal 13 n=13 Tax=Simii	1	1	P13646	M1(Oxidation)	0	8.09E-05	2	705.36029	1409.7133	0.51
AVLEVAGVHNVLAK	30S ribosomal protein S5 [Enterobacter horm	2	1	334125906		0	0.00133	2	710.53418	1420.06108	0.11
LIDEALLDQYAR	peptidyl-prolyl cis-trans isomerase (rotamase	1	1	26246452		0	0.00445	2	710.69763	1420.38799	0.32
DWENPGVTQLNR	beta-D-galactosidase [Escherichia coli CFT07	1	1	26246355		0	0.00976	2	715.07117	1429.13506	0.22
LMVEQILTDLQK	UDP-glucose 4-epimerase [Klebsiella pneumo	3	1	206580627		0	0.0243	2	716.31409	1431.6209	0.41
ILNEMRDQYEK	Keratin, type I cytoskeletal 14 n=8 Tax=Euthe	2	1	P02533		0	0.0343	2	720.02838	1439.04949	0.18
LATLPTYEEAIAR	ribosomal protein L10 [Klebsiella pneumonia	3	1	206577086		0	0.00298	2	724.93347	1448.85967	0.54
DGFVDGWAQAGTAR	50S ribosomal protein L6 [Enterobacter horm	1	1	334125908		0	0.00024	2	726.31799	1451.62871	0.48
VQGKDEVILTLNK	DNA-directed RNA polymerase subunit alpha	2	1	26249880		0	0.00801	2	729.58588	1458.16448	0.66
FLEQQNQVLQTK	Keratin, type II cytoskeletal 1 n=7 Tax=Euther	2	1	P04264		0	7.05E-05	2	738.4834	1475.95952	0.09
WELLQQVDTSTR	Keratin, type II cytoskeletal 1 n=7 Tax=Euther	1	1	P04264		0	0.00536	2	738.71472	1476.42217	0.34
SRTEAESWYQTK	Uncharacterized protein n=3 Tax=Simiiforme	2	1	E7EU87		0	0.00404	2	743.81744	1486.62761	0.47
ATLGEVGNAEHMLR	ribosomal protein L2 [Klebsiella pneumoniae	2	1	206579217		0	0.00532	2	749.43774	1497.86821	0.06
MQLNSTEISELIK	F0F1 ATP synthase subunit alpha [Proteus mi	3	1	197286887		0	0.000344	2	754.04871	1507.09014	0.65
LLKEYQELMNVK	Uncharacterized protein n=4 Tax=Simiiforme	1	1	F6RMN8		0	0.00641	2	754.90271	1508.79814	0.49
LLRDYQELMNTK	Keratin, type II cytoskeletal 1 n=7 Tax=Euther	1	1	P04264		0.0025	0.149	2	762.89807	1524.78887	0.5
AYGIEHPDEGVALR	antioxidant, AhpC/TSA family [Klebsiella pneu	1	1	206576800		0	0.00638	2	764.41003	1527.81279	0.53
SQLIDKIAAGADISK	transcriptional regulator HU subunit beta [Es	2	1	26246451		0	6.85E-05	2	765.80042	1530.59355	0.37
GVDEATIIDILTKR	Annexin A1 n=14 Tax=Eutheria RepID=ANXA1	1	1	P04083		0	0.000241	2	773.02271	1545.03813	0.58
TGVSNTLENEFKGR	chaperone protein Skp [Klebsiella pneumonia	2	1	206577097		0	0.000184	2	776.92322	1552.83916	0.53
AAVEEGVVAGGGTALVR	60 Kda chaperonin [Proteus mirabilis HI4320]	1	1	197286390		0	0.00178	2	779.02478	1557.04228	0.6
IQLVGDDLFVTNTK	enolase [Enterobacter hormaechei ATCC 491	2	2	334125399;26249182		0	0.00136	2	782.51953	1564.03179	0.6
REEESAAAAEIEER	DNA-binding protein VicH [Enterobacter horr	1	1	334124376		0	4.41E-05	2	795.81604	1590.6248	0.44
NVVLDKSFGAPTITK	chaperone GroEL [Enterobacter hormaechei	2	2	334123809;26251035		0	2.96E-06	2	795.88403	1590.76079	0.44
YHFEQSSAAIQSAR	cytochrome d ubiquinol oxidase, subunit I [Kl	1	1	206578246		0	0.0022	2	798.4458	1595.88432	0.56
EGIAGWSGENLPLVR	rhodanese domain protein [Klebsiella pneum	1	1	206578346		0	0.000879	2	799.80835	1598.60942	0.39
IATDPFVGNLTFFR	elongation factor G [Enterobacter hormaeche	1	1	334125929		0	0.0285	2	799.93079	1598.8543	0.51
SLGQFNLDGINPAPR	chaperone DnaK [Enterobacter hormaechei A	3	1	334123523		0	0.00211	2	800.23981	1599.47234	0.32
VGEEVEIVGIKETAK	translation elongation factor Tu [Enterobacte	2	1	334126439		0	0.000128	2	801.20898	1601.41069	0.27
YEEIVKEVSTYIK	Eukaryotic translation elongation factor 1 alp	3	1	A9X7H1		0	0.0498	2	801.526	1602.04473	0.6
AFDQIDNAPEEKAR	translation elongation factor Tu [Enterobacte	2	1	334126439		0	0.0257	2	802.74811	1604.48894	0.36
SFGAPTITKDGVSVAR	chaperone GroEL [Enterobacter hormaechei	2	2	334123809;26251035		0	0.000253	2	803.89746	1606.78765	0.46
ALTGHLEEVVLALLK	Annexin A1 n=14 Tax=Eutheria RepID=ANXA1	1	1	P04083		0	0.000113	2	803.76172	1606.51616	0.28
TRPDLSEIFDDSSK	phosphate acetyltransferase [Enterobacter h	3	1	334124951		0	0.000195	2	805.80066	1610.59404	0.41
SDNLKTEMDEYIR	PTS system, glucose-specific IIBC component	4	1	206579407		0	0.00607	2	807.75385	1614.50041	0.38
VLYEMDGVPEELAR	50S ribosomal protein L16 [Escherichia coli Cl	2	1	26249903		0	0.00154	2	811.43549	1621.8637	0.54
LDLAGRDLTDYLMK	Uncharacterized protein n=12 Tax=Coelomat	2	1	B4DW52		0	0.00402	2	812.53333	1624.05937	0.11
TQLIDVIADKADLSK	DNA-binding protein HU, alpha subunit [Kleb	2	1	206580695		0	5.84E-06	2	815.89996	1630.79265	0.44
WNLLQQQTTTTSSK	cDNA FLJ55805, highly similar to Keratin, type	2	1	B4DRW1		0	3.54E-05	2	818.58655	1636.16582	0.17
DLAKDITSDTSGDFR	Annexin A1 n=14 Tax=Eutheria RepID=ANXA1	1	1	P04083		0	7.82E-05	2	820.88306	1640.75884	-0.01
DLDLAQANATDQLKK	ATP synthase F0, B subunit [Klebsiella pneum	2	1	206575827		0	2.16E-05	2	822.83301	1644.65874	0.4
LGYPITDDLDIYTR	outer membrane protein A [Klebsiella pneum	2	1	206577508		0	5.60E-06	2	828.14771	1655.28813	0.23
TPAQFDADELRAAMK	Annexin A1 n=14 Tax=Eutheria RepID=ANXA1	1	1	P04083		0	0.0369	2	832.92426	1664.84123	0.52
LAGLTAQNEDQNVGIK	chaperone GroEL [Enterobacter hormaechei	1	1	334123809		0	6.61E-08	2	836.05682	1671.10637	0.12
QFNYSAEELDSVIR	glutamate synthase (NADPH), large subunit [	3	1	206576300		0	0.0128	2	836.53809	1672.0689	0.63
TRELDKLENSLGGIK	ribosomal protein S2 [Klebsiella pneumoniae	2	1	206581012		0	0.0152	2	837.64441	1674.28154	0.68
DVDAAYLNKVELEAK	cDNA FLJ55805, highly similar to Keratin, type	2	1	B4DRW1		0	0.00369	2	839.60693	1678.20659	0.17
NFDNMREDEGLADR	alkyl hydroperoxide reductase subunit C [Kle	3	1	206577586		0	0.000421	2	841.83899	1682.6707	0.47
MEFPEPVISIAVEPK	elongation factor G [Enterobacter hormaeche	1	1	334125929		0	0.00275	2	843.82837	1686.64946	0.38
AAAFEGELIPASQIDR	ribosomal protein L10 [Klebsiella pneumonia	3	1	206577086		0	0.00219	2	844.55212	1688.09697	0.12
GLGTDEDTLIEILASR	Annexin A1 n=14 Tax=Eutheria RepID=ANXA1	1	1	P04083		0	1.88E-05	2	852.17889	1703.35051	0.23
SEIDMNDIKAFYQK	Annexin A1 n=14 Tax=Eutheria RepID=ANXA1	1	1	P04083		0	0.000696	2	851.48511	1701.96294	0.07
LWDTVMEGVKQENR	formate acetyltransferase [Klebsiella pneumo	1	1	206577432		0	0.000173	2	853.39868	1705.79009	0.48
DSTIQVVENGESSQGR	Uncharacterized protein n=3 Tax=Simiiforme	3	1	E9PFJ3		0	1.43E-07	2	853.54138	1706.07549	0.14
LLDEGRAGENVGVLLR	translation elongation factor Tu [Enterobacte	2	1	334126439		0	0.00779	2	856.46149	1711.9157	0.48
NLDIERPTYTNLNR	Uncharacterized protein n=8 Tax=Coelomata	8	1	C9JDS9		0	0.0652	2	860.45337	1719.89946	0.51
DRGEDALIIYDDLSK	F0F1 ATP synthase subunit alpha [Proteus mi	3	1	197286887		0	0.000138	2	862.54968	1724.09209	0.62
MNIGQILETHLGMAAK	DNA-directed RNA polymerase, beta subunit	3	1	206579488		0	1.21E-05	2	864.37378	1727.74028	0.42
HLGPDTDVPAGDIGVGGR	NADP-specific glutamate dehydrogenase [Kle	2	1	206577927		0	1.97E-05	2	867.46509	1733.9229	0.53
GGYFPVPPVDSSQDIR	glutamine synthetase [Klebsiella pneumoniae	2	1	206580180		0	0.00304	2	867.84644	1734.68559	0.42
TIVDVLDATTTLYNAK	outer membrane protein TolC [Klebsiella pne	1	1	206576984		0	4.35E-05	2	869.73669	1738.46611	0.27
YDDAPNNVAQAVIEAR	elongation factor G [Enterobacter hormaeche	1	1	334125929		0	5.75E-05	2	873.51685	1746.02641	0.09
VYSGVVNSGDTILNSVK	elongation factor G [Enterobacter hormaeche	1	1	334125929		0	3.96E-07	2	876.85889	1752.7105	0.4
VLDTKWTLLQEQGTK	Uncharacterized protein n=3 Tax=Simiiforme	3	2	E7EU87;F6RMN8		0	0.000386	2	880.9613	1760.91533	0.48
SLGFDKVFMYLSDSR	Uncharacterized protein n=3 Tax=Simiiforme	3	1	E9PFJ3		0	0.000935	2	882.93896	1764.87065	0
AQSLDALSSMANIAGYR	NAD(P) transhydrogenase subunit alpha [Pro	4	1	197285060		0	5.49E-06	2	884.93176	1768.85625	0.49
MAVAESGMGIVEDKVIK	aldehyde-alcohol dehydrogenase [Klebsiella p	2	1	206580610		0	5.24E-06	2	889.20203	1777.39678	0.24
MVVTLIHPIAMDDGLR	translation elongation factor Tu [Enterobacte	2	1	334126439		0	0.00378	2	891.0791	1781.15093	0.1
ISNIREMLPVLEAVAK	chaperone GroEL [Enterobacter hormaechei	2	2	334123809;26251035		0	0.0278	2	892.62769	1784.24809	0.62
VSLDVNHFAPDELTVK	Heat shock protein beta-1 n=6 Tax=Simiiform	1	1	P04792		0	0.000161	2	892.69299	1784.37871	0.23

Sequence	Protein Descriptions	# Proteins	# Protein Groups	Protein Group Accessions	Modifications	q-Value	PEP	Charge	m/z [Da]	MH+ [Da]	Delta Mass [Da]
QSPASPERDYSPYYK	Keratin, type I cytoskeletal 13 n=13 Tax=Simii	1	1	P13646		0	0.00584	2	894.86316	1788.71904	0.45
SYELPDGQVITIGNER	Uncharacterized protein n=12 Tax=Coelomat	2	1	B4DW52		0	1.43E-05	2	896.39673	1791.78618	0.45
SELNEKLATAWEGFAK	formate acetyltransferase [Enterobacter horr	2	2	334122625;206577432		0	0.000514	2	897.86096	1794.71465	0.4
VELEAALQQAKEELAR	cDNA FLJ55805, highly similar to Keratin, type	2	1	B4DRW1		0	0.00017	2	899.89526	1798.78325	0.41
GITINTSHVEYDTPTR	translation elongation factor Tu [Enterobacte	2	1	334126439		0	2.24E-06	2	902.79089	1804.57451	0.34
TLQGLEIELQSQLSMK	Keratin, type I cytoskeletal 13 n=13 Tax=Simii	2	1	P13646		0	7.91E-07	2	909.94556	1818.88384	0.46
VLAEMREQYEAMAER	Keratin, type I cytoskeletal 13 n=13 Tax=Simii	1	1	P13646		0	0.00726	2	913.77954	1826.55181	0.35
FDGTVEVKDGHLIVNGK	glyceraldehyde-3-phosphate dehydrogenase	1	1	26248038		0	0.00231	2	914.5426	1828.07793	0.06
FLFNNGYADQITSVLK	aldehyde-alcohol dehydrogenase [Klebsiella p	2	1	206580610		0	2.17E-07	2	916.08093	1831.15459	0.61
NLSDVATKQEGLESVLK	Plakoglobin (Fragment) n=1 Tax=Homo sapie	2	1	Q7KZ86		0	0.00059	2	916.34521	1831.68315	0.35
TLAQLNPESSLFIIASK	Glucose-6-phosphate isomerase n=2 Tax=Cat	3	1	B4DVJ0		0	0.00357	2	916.93542	1832.86357	0.42
VPDIGADEVEITEILVK	dihydrolipoyllysine-residue acetyltransferase	1	1	206577768		0	2.19E-06	2	920.64172	1840.27617	0.14
QLDPLVVGQEHYDTAR	ATP synthase F1, beta subunit [Klebsiella pne	2	1	206577341		0	0.00849	2	921.59387	1842.18047	0.63
DTTTIIDGVGEEAAIQGR	chaperone GroEL [Enterobacter hormaechei .	2	2	334123809;26251035		0	0.00138	2	923.82385	1846.64043	0.36
GDWQNEVNVRDFIQK	formate acetyltransferase [Enterobacter horr	2	2	334122625;206577432		0	0.000458	2	924.93237	1848.85747	0.48
QIVSNAGEEPSVVANNVK	chaperone GroEL [Enterobacter hormaechei .	1	1	334123809		0	0.00174	2	927.95471	1854.90215	-0.03
GLEVKDLEHPIEVPVGK	ATP synthase F1, beta subunit [Klebsiella pne	2	1	206577341		0	0.000181	2	930.3147	1859.62212	0.3
AANAGGVATSGLEMAQNAAR	NADP-specific glutamate dehydrogenase [Kle	2	1	206577927		0	6.37E-07	2	930.56128	1860.11528	0.11
EYASFTQEQVDKIFR	aldehyde-alcohol dehydrogenase [Klebsiella p	2	1	206580610		0	6.73E-05	2	931.45715	1861.90703	0.5
EVMTTLWPFLEENPR	NADP-specific glutamate dehydrogenase [Kle	2	1	206577927		0	4.02E-05	2	931.96265	1862.91802	0.5
FQDGDLTLYQSNTILR	Uncharacterized protein n=1 Tax=Homo sapi	2	1	A8MX94		0	1.30E-06	2	942.90344	1884.79961	0.43
QLAEDPFNNWVALNKK	30S ribosomal protein S1 [Escherichia coli CF	2	1	26246936		0	0.0481	2	944.44177	1887.87627	0.45
VTAEDVLKPGTADILVPR	DNA-directed RNA polymerase, beta' subunit	2	1	206578905		0	0.0108	2	948.09253	1895.17778	0.56
ITEQDAQEMIDHLVMK	formate acetyltransferase [Enterobacter horr	1	1	334122625		0	7.20E-05	2	951.44604	1901.88481	0.49
AAYLQETGKPLDETLKK	Annexin A1 n=14 Tax=Eutheria RepID=ANXA1	1	1	P04083		0	0.000892	2	953.10925	1905.21123	0.09
NIAIEHSGYSVFAGVGER	ATP synthase F1, beta subunit [Klebsiella pne	2	1	206577341		0	1.11E-07	2	953.81506	1906.62285	0.34
LATQSNEITIPVTFESR	Heat shock protein beta-1 n=6 Tax=Simiiform	1	1	P04792		0	0.0398	2	953.82446	1906.64165	0.33
WKEGEATLAPSLDLVGKI	alkyl hydroperoxide reductase subunit C [Klel	3	1	206577586		0	0.000257	2	964.57166	1928.13603	0.54
AFTSEEFTHFLEELTK	phosphopyruvate hydratase [Escherichia coli	1	1	26249182		0	0.000546	2	965.474	1929.94072	0.51
HNDFDLNWIPVDAIER	colicin I receptor [Klebsiella pneumoniae 342	1	1	206576322		0	1.62E-05	2	977.94385	1954.88042	0.47
RVELEAALQQAKEELAR	cDNA FLJ55805, highly similar to Keratin, type	2	1	B4DRW1		0	1.12E-05	2	978.08801	1955.16875	0.55
SLNFLDFEQPIAELEAK	acetyl-CoA carboxylase carboxyltransferase s	4	1	197286115		0	0.0047	2	982.7605	1964.51372	0.26
ELLSQYDFPGDDTPIVR	translation elongation factor Tu [Enterobacte	2	1	334126439		0	0.000164	2	983.33777	1965.66826	0.35
FIEQDPEGQYGLEAAFR	DNA-directed RNA polymerase, beta subunit	3	1	206579488		0	4.39E-06	2	985.80542	1970.60356	0.34
EIELEDKFENMGAQMVK	chaperone GroEL [Enterobacter hormaechei .	3	3	334123809;197286390;26251035		0	0.00591	2	1006.41919	2011.8311	0.44
QTDELTGLSSLVVLDSAER	DNA-directed RNA polymerase, beta' subunit	2	1	206578905		0	8.17E-07	2	1017.41687	2033.82646	0.39
LVWQVPVGTVEDTGPLGIR	quinate dehydrogenase (pyrroloquinoline-qu	1	1	206576961		0	3.66E-07	2	1019.00623	2037.00517	0.44
SAELNKEVSTNTAMIQTSK	Keratin, type I cytoskeletal 13 n=13 Tax=Simii	1	1	P13646		0	1.53E-05	2	1026.80115	2052.59502	0.28
EKPTWLEVDAGKMEGTFK	ribosomal protein S4 [Klebsiella pneumoniae	3	1	206575742		0	0.000246	2	1033.60437	2066.20146	0.09
GDRSEDFGVNEDLADSDAR	Annexin A1 n=14 Tax=Eutheria RepID=ANXA1	1	1	P04083		0	1.33E-08	2	1034.94006	2068.87285	0.49
SAELNKEVSTNTAmIQTSK	Keratin, type I cytoskeletal 13 n=13 Tax=Simii	1	1	P13646	M14(Oxidation)	0	1.02E-06	2	1034.78662	2068.56597	0.27
VLGDKIQLVGDDLFVTNTK	enolase [Enterobacter hormaechei ATCC 491	2	2	334125399;26249182		0	1.05E-05	2	1038.65259	2076.2979	0.58
REDFANETADDSEAGDSEE	ribosomal protein S6 [Klebsiella pneumoniae	1	1	206579361		0	8.44E-08	2	1044.19885	2087.39043	0.3
SKIFDFVKPGVITGDDVQK	fructose-bisphosphate aldolase [Klebsiella pn	2	1	206578717		0	0.0102	3	698.73071	2094.17759	0.35
SGETEDATIADLAVGTAAGQIK	enolase [Enterobacter hormaechei ATCC 491	2	2	334125399;26249182		0	2.71E-09	2	1060.20654	2119.40581	0.68
VQVINPNSMAALGIEVVDVR	HflC protein [Klebsiella pneumoniae 342]	1	1	206580136		0	0.000335	2	1062.875	2124.74272	0.3
TIEELRDKILTATIENNR	Keratin, type I cytoskeletal 13 n=13 Tax=Simii	1	1	P13646		0	0.000661	3	710.83093	2130.47824	0.44
ADIDYNTSEAHTTYGVIGVK	ribosomal protein S3 [Klebsiella pneumoniae	3	1	206580744		0	0.000665	2	1077.9259	2154.84453	0.4
AYTNFDAERDALNIETAIK	Uncharacterized protein n=2 Tax=Simiiforme	2	1	F7GML9		0	0.00511	2	1078.63818	2156.26909	0.6
AGDQIQSGVDAAIKAGNTLPMR	ribosomal protein L2 [Klebsiella pneumoniae	2	1	206579217		0	1.81E-05	3	738.45923	2213.36313	0.08
QQIEEATSDYDREKLQER	chaperonin GroEL [Escherichia coli CFT073]	1	1	26251035		0	0.00756	2	1120.06104	2239.11479	0.53
ETLLDAVEHPEKYPQLTIR	autonomous glycyl radical cofactor [Klebsiell	1	1	206580560		0	0.0864	3	751.81348	2253.42588	0.41
NVVLDKSFGAPTITKDGVSVAR	chaperone GroEL [Enterobacter hormaechei .	2	2	334123809;26251035		0	4.35E-06	2	1138.00476	2275.00224	0.38
EMLLDAMENPEKYPQLTIR	formate acetyltransferase [Klebsiella pneumo	1	1	206577432		0	0.00162	2	1146.49878	2291.99028	0.42
AEAESWYQTKYEELQVTAGR	Uncharacterized protein n=4 Tax=Simiiforme	1	1	F6RMN8		0	0.000158	2	1180.54541	2360.08354	0.48
DGAALGYSHGFNIVEVGEQIRK	ketol-acid reductoisomerase [Escherichia coli	3	1	161486053		0	0.000915	3	787.33386	2359.98703	-0.07
AANDDLLNSFWLLDSEKGEAR	autonomous glycyl radical cofactor [Klebsiella	1	1	206580560		0	0.00318	2	1182.88477	2364.76225	0.31
VVINKDTTTIIDGVGEEAAIQGR	chaperone GroEL [Enterobacter hormaechei .	2	2	334123809;26251035		0	0.00693	3	800.75574	2400.25266	0.33
NEQDGGDLVYFQGHISPGVYAR	pyruvate dehydrogenase (acetyl-transferring	2	1	206576843		0	0.000279	3	808.36633	2423.08445	0.31
TNEAYLQGQLGNPKGEDQPNKK	fructose-bisphosphate aldolase [Klebsiella pn	2	1	206578717		0	0.0677	3	810.84094	2430.50827	0.43
DLAKDITSDTSGDFRNALLSLAK	Annexin A1 n=14 Tax=Eutheria RepID=ANXA1	1	1	P04083		0	4.84E-06	3	818.14203	2452.41153	0.38
VLESAIANAEHNDGADIDDLKVAK	50S ribosomal protein L22 [Proteus mirabilis	2	1	197287073		0	0.00289	3	837.17194	2509.50125	0.41
VINDNFGIVEGLmTTVHATTATQK	glyceraldehyde-3-phosphate dehydrogenase	1	1	206580183	M13(Oxidation)	0	0.0138	3	859.66431	2576.97837	0.23
VDQLSNDVNAMRSDVQAAKDDAAR	major outer membrane lipoprotein [Escheric	2	1	26247927		0	0.0119	3	864.0575	2590.15793	0.31
AMEAPLRQIVSNAGEEPSVVANNVK	chaperone GroEL [Enterobacter hormaechei .	1	1	334123809		0	0.00111	2	1312.50793	2624.00859	0.33
FGGQVLDTVDIENYISVPKTEGQK	alkyl hydroperoxide reductase subunit F [Klel	2	1	206576199		0	3.72E-05	3	880.14355	2638.41611	0.36
EKSVEELNAELLNLLREQFNLR	ribosomal protein L29 [Klebsiella pneumonia	2	1	206578965		0	9.59E-06	3	886.90784	2658.70896	0.43
SRTEAESWYQTKYEELQQTAGR	Uncharacterized protein n=3 Tax=Simiiforme	2	1	E7EU87		0	3.61E-05	3	888.03442	2662.08872	0.28
IVIKVNEDEMYPGEAGIDIYNLTK	DNA-directed RNA polymerase, beta subunit	3	1	206579488		0	0.000174	3	909.14453	2725.41904	0.35
TDLDKLVIEMETNGTIDPEEAIRR	DNA-directed RNA polymerase subunit alpha	2	1	26249880		0	0.00206	3	920.51245	2759.5228	0.38
EGVITVEDGTGLEDELDVVEGMQFDR	chaperone GroEL [Enterobacter hormaechei .	1	1	334123809		0	0.000152	3	951.73376	2853.18674	0.29
AAYSSGKPAIGVGAGNTPVVIDETADIKR	aldehyde-alcohol dehydrogenase [Klebsiella p	2	1	206580610		0	6.58E-09	3	953.48804	2858.44956	0.31
RAVIESENSAERDQLLENLQEGMEVK	30S ribosomal protein S1 [Escherichia coli CF	2	1	26246936		0	4.33E-06	3	996.81458	2988.42917	0.32
TTGIVMDSGDGVTHTVPIYEGYALPHAILR	Uncharacterized protein n=12 Tax=Coelomat	2	1	B4DW52		0	0.000109	3	1062.21484	3184.62998	0.34
NHEEEMKEFSNQVVGQVNVEMDATPGIDLTR	Keratin, type I cytoskeletal 13 n=13 Tax=Simii	1	1	P13646		0	2.53E-05	3	1173.28381	3517.83689	0.4

2DLCSCI_34 Protein Summary

index	Prot_hit	Accession	Description	Mascot Score	Coverage	# Peptides	# PSMs	# AAs	MW [kDa]	calc. pI
1	1.1	334126439	translation elongation factor Tu [Enteroba	259.21	16.49%	5	8	376	41.1	5.24
2	1.2	206581075	translation elongation factor Tu [Klebsiella	259.21	15.74%	5	8	394	43.2	5.53
3	2.1	334125906	30S ribosomal protein S5 [Enterobacter hc	52.55	8.43%	1	1	166	17.5	10.05
4	2.2	206580716	ribosomal protein S5 [Klebsiella pneumoni	52.55	8.38%	1	1	167	17.6	10.11
5	3	334124376	DNA-binding protein VicH [Enterobacter h	93.41	10.22%	1	1	137	15.6	5.16
6	4	334123949	50S ribosomal protein L11 [Enterobacter h	100.95	9.86%	1	1	142	14.9	9.63
7	5	334123809	chaperone GroEL [Enterobacter hormaech	774.57	30.11%	10	16	548	57.1	4.94
8	6.1	334123523	chaperone DnaK [Enterobacter hormaeche	62.89	2.35%	1	1	637	69.1	4.96
9	6.2	206580041	chaperone protein DnaK [Klebsiella pneum	62.89	2.35%	1	1	638	69.1	4.97
10	6.3	26245936	molecular chaperone DnaK [Escherichia cc	62.89	2.35%	1	1	638	69.1	4.97
11	7	334122625	formate acetyltransferase [Enterobacter h	236.46	10.39%	5	6	760	85	6.09
12	8	334122578	outer membrane protein A [Enterobacter	105.67	5.59%	2	3	358	38.3	5.24
13	9	206577432	formate acetyltransferase [Klebsiella pneu	182.41	8.03%	4	5	760	85.2	5.85
14	10.1	206577586	alkyl hydroperoxide reductase subunit C [I	117.43	17.11%	3	3	187	20.8	5.17
15	10.2	334122926	alkyl hydroperoxide reductase C [Enteroba	117.43	15.92%	3	3	201	22.4	5.02
16	10.3	26246586	alkyl hydroperoxide reductase subunit C [I	117.43	17.11%	3	3	187	20.7	5.17
17	11	206576800	antioxidant, AhpC/TSA family [Klebsiella pı	68.79	7.00%	1	1	200	22.3	5.29
18	12.1	206576843	pyruvate dehydrogenase (acetyl-transferri	86.71	1.92%	1	2	887	99.5	5.64
19	12.2	334123432	pyruvate decarboxylase, E1 component of	86.71	1.92%	1	2	887	99.6	5.69
20	12.3	26246055	pyruvate dehydrogenase subunit E1 [Esch	86.71	1.92%	1	2	887	99.6	5.68
21	13.1	206580695	DNA-binding protein HU, alpha subunit [Kl	62.94	16.67%	1	1	90	9.5	9.58
22	13.2	334123929	DNA-binding protein HU-beta [Enterobact	62.94	16.67%	1	1	90	9.5	9.58
23	14.1	197285345	bifunctional acetaldehyde-CoA/alcohol de	87.63	2.14%	1	1	887	96.2	6.38
24	14.2	334124374	aldehyde-alcohol dehydrogenase [Enterok	87.63	2.07%	1	1	920	99.1	6.81
25	14.3	206580610	aldehyde-alcohol dehydrogenase [Klebsiel	87.63	2.13%	1	1	891	95.9	6.79
26	14.4	26247570	bifunctional acetaldehyde-CoA/alcohol de	87.63	2.13%	1	1	891	96.1	6.79
27	15	197286390	60 Kda chaperonin [Proteus mirabilis HI43	158.69	8.39%	2	4	548	57.6	4.89
28	16.1	197286616	50S ribosomal protein L10 [Proteus mirabi	104.53	7.88%	1	2	165	17.6	8.68
29	16.2	334123947	50S ribosomal protein L10 [Enterobacter h	104.53	7.88%	1	2	165	17.8	8.98
30	16.3	206577086	ribosomal protein L10 [Klebsiella pneumor	104.53	7.88%	1	2	165	17.8	8.98
31	16.4	26250755	50S ribosomal protein L10 [Escherichia col	104.53	7.88%	1	2	165	17.7	8.98
32	17.1	197286887	F0F1 ATP synthase subunit alpha [Proteus	227.1	2.53%	1	3	513	55.5	5.69
33	17.2	334121921	ATP synthase F1 sector alpha subunit [Ente	227.1	2.53%	1	3	513	55.3	6.13
34	17.3	206580919	ATP synthase F1, alpha subunit [Klebsiella	227.1	2.53%	1	3	513	55.1	6
35	17.4	26250478	F0F1 ATP synthase subunit alpha [Escheric	227.1	2.53%	1	3	513	55.2	6.13
36	18.1	197287068	50S ribosomal protein L3 [Proteus mirabili	84.35	9.09%	1	2	209	22.5	9.86
37	18.2	334125923	50S ribosomal protein L3 [Enterobacter hc	84.35	9.09%	1	2	209	22.2	9.82
38	18.3	206576036	ribosomal protein L3 [Klebsiella pneumoni	84.35	9.09%	1	2	209	22.2	9.91
39	18.4	26249915	50S ribosomal protein L3 [Escherichia coli	84.35	9.09%	1	2	209	22.2	9.91
40	19.1	197287127	transcription termination factor Rho [Prot	87.39	5.25%	1	1	419	46.9	7.25
41	19.2	334126167	transcription termination factor Rho [Ente	87.39	5.25%	1	1	419	47	7.25
42	19.3	206576949	transcription termination factor Rho [Kleb	87.39	5.25%	1	1	419	47	7.25
43	19.4	26250521	transcription termination factor Rho [Esch	87.39	4.97%	1	1	443	49.6	7.3
44	20.1	161486147	autonomous glycyl radical cofactor GrcA [I	244.8	16.54%	1	3	127	14.3	5.17
45	20.2	206580560	autonomous glycyl radical cofactor [Klebsi	244.8	16.54%	1	3	127	14.2	5.02
46	21	26249182	phosphopyruvate hydratase [Escherichia c	227.61	13.19%	3	4	432	45.6	5.48
47	22	P35579	Myosin-9 n=67 Tax=Tetrapoda RepID=MYI	185.28	2.45%	3	4	1960	226.4	5.6
48	23	P15924	Desmoplakin n=28 Tax=Mammalia RepID=	76.4	0.45%	1	1	2871	331.6	6.81
49	24	P04264	Keratin, type II cytoskeletal 1 n=7 Tax=Eutl	1746.82	28.11%	14	35	644	66	8.12
50	25	P35527	Keratin, type I cytoskeletal 9 n=4 Tax=Cata	96.6	6.42%	2	3	623	62	5.24
51	26	P13645	Keratin, type I cytoskeletal 10 n=12 Tax=Eu	240.51	15.41%	6	7	584	58.8	5.21
52	27	P02533	Keratin, type I cytoskeletal 14 n=8 Tax=Eut	317.41	11.23%	5	10	472	51.5	5.16
53	28	Q7RTS7	Keratin, type II cytoskeletal 74 n=7 Tax=Sir	373.36	6.81%	3	14	529	57.8	7.71
54	29	P06702	Protein S100-A9 n=5 Tax=Hominoidea Rep	121.49	24.56%	2	3	114	13.2	6.13
55	30	O60437	Periplakin n=3 Tax=Hominoidea RepID=PE	126.93	1.99%	2	2	1756	204.6	5.6
56	31	P62937	Peptidyl-prolyl cis-trans isomerase A n=98	108.16	9.09%	1	2	165	18	7.81
57	32.1	E9PFJ3	Uncharacterized protein n=3 Tax=Simiiforı	235.29	5.02%	2	4	578	63.3	5.31
58	32.2	P07911	Uromodulin n=2 Tax=Catarrhini RepID=UR	235.29	4.53%	2	4	640	69.7	5.24
59	33	A4FUA1	GUCA1B protein (Fragment) n=1 Tax=Hom	1314.14	58.67%	3	24	75	8.6	5.41
60	34	P13646	Keratin, type I cytoskeletal 13 n=13 Tax=Si	2129.98	34.06%	12	55	458	49.6	4.96
61	35.1	B4DRW1	cDNA FLJ55805, highly similar to Keratin, t	1113.26	24.05%	11	33	474	51.7	6.81
62	35.2	P19013	Keratin, type II cytoskeletal 4 n=14 Tax=Sir	1113.26	21.35%	11	33	534	57.2	6.61
63	36	F6RMN8	Uncharacterized protein n=4 Tax=Simiiforı	607.51	13.46%	7	23	535	57.8	8.22

index	Prot_hit	Accession	Description	Score	Coverage	# Peptides	# PSMs	# AAs	MW [kDa]	calc. pI
64	37.1	P06899	Histone H2B type 1-J n=102 Tax=Euteleost	98.7	11.90%	1	1	126	13.9	10.32
65	37.2	P62807	Histone H2B type 1-C/E/F/G/I n=44 Tax=Eu	98.7	11.90%	1	1	126	13.9	10.32
66	37.3	B4DR52	Histone H2B n=5 Tax=Euarchontoglires Re	98.7	9.04%	1	1	166	18	10.32
67	37.4	P58876	Histone H2B type 1-D n=136 Tax=Coeloma	98.7	11.90%	1	1	126	13.9	10.32
68	38.1	UPI0000D48568	UPI0000D48568 related cluster n=1 Tax=H	116.71	13.98%	2	3	186	20.4	9.06
69	38.2	P04792	Heat shock protein beta-1 n=6 Tax=Simiifo	116.71	12.68%	2	3	205	22.8	6.4
70	39.1	A8MX94	Uncharacterized protein n=1 Tax=Homo sa	132.02	9.20%	1	1	174	19.5	5.97
71	39.2	P09211	Glutathione S-transferase P n=4 Tax=Simii	132.02	7.62%	1	1	210	23.3	5.64
72	40.1	E7EU87	Uncharacterized protein n=3 Tax=Simiiforn	422.23	7.03%	4	17	555	58.9	5.97
73	40.2	P13647	Keratin, type II cytoskeletal 5 n=17 Tax=Eu	422.23	6.61%	4	17	590	62.3	7.74
74	41	P04083	Annexin A1 n=14 Tax=Eutheria RepID=ANX	939.97	27.75%	8	19	346	38.7	7.02
75	42.1	Q7KZ86	Plakoglobin (Fragment) n=1 Tax=Homo sap	48.53	9.94%	1	1	171	18.8	8.85
76	42.2	P14923	Junction plakoglobin n=31 Tax=Theria Rep	48.53	2.28%	1	1	745	81.7	6.14
77	43.1	B4DVJ0	Glucose-6-phosphate isomerase n=2 Tax=(	75.31	3.20%	1	1	531	60	8.65
78	43.2	B4DG39	Glucose-6-phosphate isomerase n=5 Tax=I	75.31	2.99%	1	1	569	64.3	8.91
79	43.3	P06744	Glucose-6-phosphate isomerase n=17 Tax=	75.31	3.05%	1	1	558	63.1	8.32
80	44	Q9UBG3	Cornulin n=6 Tax=Catarrhini RepID=CRNN_	59.98	3.23%	1	1	495	53.5	6.1
81	45.1	Q96GV1	Enolase (Fragment) n=2 Tax=Eutheria RepI	123.98	6.52%	1	3	184	20.4	7.03
82	45.2	P06733	Alpha-enolase n=53 Tax=Euteleostomi Rep	123.98	2.76%	1	3	434	47.1	7.39

2DLCSCI_34 Peptide Summary

Sequence	Protein Descriptions	# Proteins	# Protein Groups	Protein Group Accessions	Modifications	q-Value	PEP	Charge	m/z [Da]	MH+ [Da]	Delta Mass [Da]
NLDLDSI	Keratin (Fragment) n=2 Tax=Eutheria RepID=Q9UEK9_H	1	1	Q9UEK9		0.00706	0.304	1	789.65686	789.65686	0.26
YENELALR	Keratin, type I cytoskeletal 13 n=13 Tax=Simiiformes Re	1	1	P13646		0	0.00227	2	504.59619	1008.18511	0.34
LLEGEEYR	cDNA FLJ55805, highly similar to Keratin, type II cytoske	2	1	B4DRW1		0	0.000396	2	505.0282	1009.04912	0.27
DLTDYLmK	ACTB protein (Fragment) n=3 Tax=Euteleostomi RepID=	8	1	A0AUL6	M7(Oxidation)	0	0.00857	1	1014.47729	1014.47729	0
SDVLFNFNK	outer membrane protein A [Enterobacter hormaechei A	1	1	334122578		0	0.00126	1	1083.7218	1083.7218	0.17
DAEAWFNEK	Keratin, type I cytoskeletal 10 n=12 Tax=Eutheria RepID	1	1	P13645		0	0.00186	1	1109.52161	1109.52161	0.03
DAEEWFHAK	GUCA1B protein (Fragment) n=1 Tax=Homo sapiens Re	1	1	A4FUA1		0	0.00434	2	566.50684	1132.0064	-0.25
LFDQAFGLPR	UPI0000D48568 related cluster n=1 Tax=Homo sapiens	2	1	UPI0000D48568		0	0.00179	2	583.00739	1165.00749	0.69
YEELQITAGR	Keratin, type II cytoskeletal 1 n=7 Tax=Eutheria RepID=	1	1	P04264		0	8.47E-08	2	590.80835	1180.60942	0.5
WTLLQEQGTK	Uncharacterized protein n=3 Tax=Simiiformes RepID=E	3	2	E7EU87;F6RMN8		0	0.003	2	602.83856	1204.66985	0.52
AQSVVDYLVSK	outer membrane protein A [Enterobacter hormaechei A	1	1	334122578		0	0.000967	2	605.34656	1209.68584	0.52
LKYENELALR	Keratin, type I cytoskeletal 13 n=13 Tax=Simiiformes Re	1	1	P13646		0	9.92E-05	2	625.1228	1249.23833	0.27
TPAQFDADELR	Annexin A1 n=14 Tax=Eutheria RepID=ANXA1_HUMAN	1	1	P04083		0	0.000107	2	632.10077	1263.19426	0.3
ALNSIIDVYHK	Protein S100-A8 n=5 Tax=Hominoidea RepID=S10A8_H	1	1	P05109		0	0.0089	2	637.03558	1273.06389	0.18
ALEEANADLEVK	Keratin, type I cytoskeletal 14 n=8 Tax=Eutheria RepID=	2	2	P02533;P13646		0	4.23E-06	1	1301.63452	1301.63452	-0.02
SLDLDSIIAEVK	Keratin, type II cytoskeletal 1 n=7 Tax=Eutheria RepID=	1	1	P04264		0	9.44E-07	2	652.2124	1303.41753	0.35
AGALNSNDAFVLK	Gelsolin (Amyloidosis, Finnish type) n=3 Tax=Catarrhini	3	1	Q5T0H8		0	0.00018	2	660.74561	1320.48393	0.39
IVGLQTEAPLKR	formate acetyltransferase [Enterobacter hormaechei A	2	2	334122625;206577432		0	0.000462	2	662.9939	1324.98052	0.09
NLDLDSIIAEVK	Uncharacterized protein n=3 Tax=Simiiformes RepID=E	3	2	E7EU87;F6RMN8		0	1.93E-07	2	665.61285	1330.21843	0.25
TAAENDFVVLKK	cDNA FLJ55805, highly similar to Keratin, type II cytoske	3	1	B4DRW1		0	3.98E-06	2	668.37512	1335.74297	0.51
SKAEAEALYQTK	cDNA FLJ55805, highly similar to Keratin, type II cytoske	4	2	B4DRW1;Q7RTS7		0	1.07E-06	2	670.25024	1339.49321	0.4
RVELEAALQQAK	cDNA FLJ55805, highly similar to Keratin, type II cytoske	2	1	B4DRW1		0	2.10E-05	2	678.64282	1356.27837	0.26
NLDLDSIIAEVR	cDNA FLJ55805, highly similar to Keratin, type II cytoske	3	1	B4DRW1		0	3.26E-08	2	679.65588	1358.30449	0.29
DLDLDSIIAEVR	Keratin, type II cytoskeletal 74 n=7 Tax=Simiiformes Re	1	1	Q7RTS7		0	1.97E-05	2	680.13831	1359.26933	0.28
NKLEGLEDALQK	Uncharacterized protein n=4 Tax=Simiiformes RepID=F	1	1	F6RMN8		0	7.70E-07	2	679.70551	1358.40373	0.34
SAYQEAMDISKK	14-3-3 protein sigma n=20 Tax=Eutheria RepID=1433S_	1	1	P31947		0	0.019	2	686.38702	1371.76677	0.55
AFDQIDNAPEEK	translation elongation factor Tu [Enterobacter hormaec	2	1	334126439		0	0.000433	2	689.02301	1377.03874	0.2
TRLEQEIATYR	Keratin, type I cytoskeletal 14 n=8 Tax=Eutheria RepID=	2	2	P02533;P13646		0	0.000188	2	690.87036	1380.73345	0.5
ALEESNYELEGK	Keratin, type I cytoskeletal 10 n=12 Tax=Eutheria RepID	1	1	P13645		0	7.30E-06	2	691.92523	1382.84319	0.6
MIGFPSSAGSVSPR	Keratin, type I cytoskeletal 13 n=13 Tax=Simiiformes Re	1	1	P13646		0	3.44E-08	2	697.04578	1393.08428	0.2
VDSLNDEINFLK	cDNA FLJ55805, highly similar to Keratin, type II cytoske	3	1	B4DRW1		0	3.26E-09	2	704.4856	1407.96391	0.62
ADTLTDEINFLR	Uncharacterized protein n=4 Tax=Simiiformes RepID=F	1	1	F6RMN8		0	5.83E-05	2	704.58215	1408.15703	0.22
mIGFPSSAGSVSPR	Keratin, type I cytoskeletal 13 n=13 Tax=Simiiformes Re	1	1	P13646	M1(Oxidation)	0	1.61E-11	2	705.3761	1409.74492	0.53
TALQPMVSALNIR	Uncharacterized protein n=3 Tax=Simiiformes RepID=E	2	1	E9PFJ3		0	0.000184	2	707.7345	1414.46172	0.34
AVLEVAGVHNVLAK	30S ribosomal protein S5 [Enterobacter hormaechei AT	2	1	334125906		0	7.92E-06	2	710.94006	1420.87285	0.52
IAKEEMESEMAR	cysteine desulfurase IscS [Klebsiella pneumoniae 342]	2	1	206578600		0	0.0132	2	712.06763	1423.12798	-0.26
YISPDQLADLYK	Enolase (Fragment) n=2 Tax=Eutheria RepID=Q96GV1_	2	1	Q96GV1		0	1.06E-06	2	713.90002	1426.79277	0.53
IRLENEIQTYR	Keratin, type I cytoskeletal 10 n=12 Tax=Eutheria RepID	1	1	P13645		0	0.000374	2	718.15448	1435.30168	0.27
AGAPFGPGANPMHGR	formate acetyltransferase [Enterobacter hormaechei A	2	2	334122625;206577432		0	1.33E-07	2	719.34906	1437.69084	0.5
ILNEMRDQYEK	Keratin, type I cytoskeletal 14 n=8 Tax=Eutheria RepID=	1	1	P02533		0	6.72E-05	2	720.05133	1439.09538	0.2
LATLPTYEEAIAR	50S ribosomal protein L10 [Proteus mirabilis HI4320]	4	1	197286616		0	1.69E-08	2	724.8877	1448.76811	0.49
SRAEAESWYQTK	Uncharacterized protein n=4 Tax=Simiiformes RepID=F	1	1	F6RMN8		0	0.00448	2	728.82263	1456.63799	0.48
LGHPDTLNQGEFK	Protein S100-A9 n=5 Tax=Hominoidea RepID=S10A9_H	1	1	P06702		0	2.42E-05	2	728.99866	1456.99004	0.63
AADMTGSDIEAMTR	50S ribosomal protein L11 [Enterobacter hormaechei A	1	1	334123949		0	3.64E-10	2	735.27069	1469.53411	0.45
WELLQQVDTSTR	Keratin, type II cytoskeletal 1 n=7 Tax=Eutheria RepID=	1	1	P04264		0	8.48E-08	2	739.03387	1477.06047	0.66
FLEQQNQVLQTK	Keratin, type II cytoskeletal 1 n=7 Tax=Eutheria RepID=	1	1	P04264		0	1.22E-07	2	738.73999	1476.4727	0.34
FLEQQNQVLETK	Keratin, type II cytoskeletal 74 n=7 Tax=Simiiformes Re	1	1	Q7RTS7		0	6.75E-05	2	739.4762	1477.94512	0.59
SRTEAESWYQTK	Uncharacterized protein n=3 Tax=Simiiformes RepID=E	2	1	E7EU87		0	0.000345	2	743.44922	1485.89116	0.1
SQYEQLAEQNRK	Keratin, type I cytoskeletal 10 n=12 Tax=Eutheria RepID	1	1	P13645		0	0.000716	2	747.8479	1494.68852	0.48
MQLNSTEISELIK	F0F1 ATP synthase subunit alpha [Proteus mirabilis HI4	4	1	197286887		0	3.28E-11	2	754.00653	1507.00578	0.61
SLYEADLVDEAKR	phosphoglycerate kinase [Klebsiella pneumoniae 342]	3	1	206579376		0	0.000104	2	755.53906	1510.07085	0.66
TLELQGLINDLQR	Desmoplakin n=28 Tax=Mammalia RepID=DESP_HUMA	1	1	P15924		0	1.75E-06	2	757.5929	1514.17851	0.67
LLRDYQELMNTK	Keratin, type II cytoskeletal 1 n=7 Tax=Eutheria RepID=	1	1	P04264		0	0.000412	2	762.81409	1524.6209	0.42
AYGIEHPDEGVALR	antioxidant, AhpC/TSA family [Klebsiella pneumoniae 3	1	1	206576800		0	2.14E-07	2	764.03522	1527.06316	0.15
MLREYQELMSVK	cDNA FLJ55805, highly similar to Keratin, type II cytoske	3	1	B4DRW1		0	0.000331	2	764.45721	1527.90715	0.57
GVDEATIIDILTKR	Annexin A1 n=14 Tax=Eutheria RepID=ANXA1_HUMAN	1	1	P04083		0	6.48E-09	2	772.85425	1544.70122	0.42
NVVLDKSFGAPTITK	chaperone GroEL [Enterobacter hormaechei ATCC 4916	1	1	334123809		0	1.95E-08	2	795.67126	1590.33525	0.22
REEESAAAAEIEER	DNA-binding protein VicH [Enterobacter hormaechei A	1	1	334124376		0	1.99E-10	2	795.77728	1590.54729	0.4
SLGQFNLDGINPAPR	chaperone DnaK [Enterobacter hormaechei ATCC 4916	3	1	334123523		0	2.65E-05	2	800.44928	1599.89128	0.53
NKLNDLEDALQQAK	Keratin, type II cytoskeletal 1 n=7 Tax=Eutheria RepID=	1	1	P04264		0	2.24E-06	2	800.88782	1600.76836	0.47
VGEEVEIVGIKETAK	translation elongation factor Tu [Enterobacter hormaec	2	1	334126439		0	4.19E-07	2	801.47919	1601.9511	0.54
AFDQIDNAPEEKAR	translation elongation factor Tu [Enterobacter hormaec	2	1	334126439		0	0.0276	2	802.85144	1604.6956	0.46
EGEATLAPSLDLVGKI	alkyl hydroperoxide reductase subunit C [Klebsiella pne	3	1	206577586		0	2.29E-07	2	807.47644	1613.9456	0.53
TQLIDVIADKADLSK	DNA-binding protein HU, alpha subunit [Klebsiella pneu	2	1	206580695		0	6.31E-07	2	816.07471	1631.14214	0.62
WNLLQQQTTTTSSK	cDNA FLJ55805, highly similar to Keratin, type II cytoske	2	1	B4DRW1		0	2.29E-10	2	818.62781	1636.24834	0.21
SLNNQFASFIDKVR	Keratin, type II cytoskeletal 1 n=7 Tax=Eutheria RepID=	1	1	P04264		0	1.93E-06	2	820.60449	1640.20171	0.67
DLAKDITSDTSGDFR	Annexin A1 n=14 Tax=Eutheria RepID=ANXA1_HUMAN	1	1	P04083		0	1.83E-11	2	821.0484	1641.08953	0.16
ALEEAmEQKAELER	Myosin-9 n=67 Tax=Tetrapoda RepID=MYH9_HUMAN	1	1	P35579	M6(Oxidation)	0	0.000232	2	832.42963	1663.85198	0.53
TPAQFDADELRAAMK	Annexin A1 n=14 Tax=Eutheria RepID=ANXA1_HUMAN	1	1	P04083		0	1.45E-06	2	832.89368	1664.78008	0.48
LAGLTAQNEDQNVGIK	chaperone GroEL [Enterobacter hormaechei ATCC 4916	1	1	334123809		0	2.09E-05	2	836.34369	1671.6801	0.4
DVDAAYLNKVELEAK	cDNA FLJ55805, highly similar to Keratin, type II cytoske	2	1	B4DRW1		0	5.74E-05	2	839.53528	1678.06328	0.1
NFDNMREDEGLADR	alkyl hydroperoxide reductase subunit C [Klebsiella pne	3	1	206577586		0	0.000117	2	841.51538	1682.02349	0.15
GLGTDEDTLIEILASR	Annexin A1 n=14 Tax=Eutheria RepID=ANXA1_HUMAN	1	1	P04083		0	2.25E-10	2	852.24658	1703.48589	0.3
DSTIQVVENGESSQGR	Uncharacterized protein n=3 Tax=Simiiformes RepID=E	2	1	E9PFJ3		0	8.02E-09	2	853.62445	1706.24162	0.22
GSLGGGFSSGGFSGGSFSR	Keratin, type I cytoskeletal 10 n=12 Tax=Eutheria RepID	1	1	P13645		0	3.18E-15	2	854.95825	1708.90923	0.57
QISNLQQSISDAEQR	Keratin, type II cytoskeletal 1 n=7 Tax=Eutheria RepID=	1	1	P04264		0	1.17E-13	2	859.16223	1717.31719	0.23
QLLQANPILEAFGNAK	Myosin-9 n=67 Tax=Tetrapoda RepID=MYH9_HUMAN	1	1	P35579		0	2.00E-07	2	864.54883	1728.09038	0.57
SEDFGVNEDLADSDAR	Annexin A1 n=14 Tax=Eutheria RepID=ANXA1_HUMAN	1	1	P04083		0	2.03E-11	2	870.87317	1740.73906	0.5
GAVPGATGSDLIVKPAVKA	50S ribosomal protein L3 [Proteus mirabilis HI4320]	4	1	197287068		0	3.59E-06	2	876.37097	1751.73467	0.36
VLDTKWTLLQEQGTK	Uncharacterized protein n=3 Tax=Simiiformes RepID=E	3	2	E7EU87;F6RMN8		0	1.18E-07	2	880.8324	1760.65752	0.35
AmGIMNSFVNDIFER	Histone H2B type 1-J n=102 Tax=Euteleostomi RepID=H	4	1	P06899	M2(Oxidation)	0	2.36E-09	2	880.87219	1760.73711	0.46
AIAQVGTISANSDETVGK	chaperone GroEL [Enterobacter hormaechei ATCC 4916	1	1	334123809		0	0.00435	2	881.33398	1761.66069	0.38
sELEQLRQEAEQLR	Uncharacterized protein n=1 Tax=Homo sapiens RepID=	3	1	C9JD14	N-Term(Acetyl)	0	0.00499	2	886.47278	1771.93828	0.52
VSLDVNHFAPDELTVK	UPI0000D48568 related cluster n=1 Tax=Homo sapiens	2	1	UPI0000D48568		0	3.03E-06	2	892.77454	1784.5418	0.31
ISPQIQLSGQTEQTQK	Cornulin n=6 Tax=Catarrhini RepID=CRNN_HUMAN	1	1	Q9UBG3		0	6.62E-09	2	894.13647	1787.26567	0.67
QSPASPERDYSPYYK	Keratin, type I cytoskeletal 13 n=13 Tax=Simiiformes Re	1	1	P13646		0	7.61E-07	2	894.79309	1788.57891	0.38
VELEAALQQAKEELAR	cDNA FLJ55805, highly similar to Keratin, type II cytoske	2	1	B4DRW1		0	2.21E-07	2	900.1189	1799.23052	0.63
GITINTSHVEYDTPTR	translation elongation factor Tu [Enterobacter hormaec	2	1	334126439		0	4.38E-06	2	902.87585	1804.74443	0.43
NIETIINTFHQYSVK	Protein S100-A9 n=5 Tax=Hominoidea RepID=S10A9_H	1	1	P06702		0	5.66E-07	2	904.10266	1807.19805	0.13
TLQGLEIELQSQLSMK	GUCA1B protein (Fragment) n=1 Tax=Homo sapiens Re	2	2	A4FUA1;P13646		0	1.98E-12	2	909.67896	1818.35063	0.19
VLAEMREQYEAMAER	Keratin, type I cytoskeletal 13 n=13 Tax=Simiiformes Re	1	1	P13646		0	0.000819	2	913.87158	1826.73589	0.44
NLSDVATKQEGLESVLK	Plakoglobin (Fragment) n=1 Tax=Homo sapiens RepID=	2	1	Q7KZ86		0	2.44E-05	2	916.1416	1831.27593	0.15
SIYGEKFEDENFILK	Peptidyl-prolyl cis-trans isomerase A n=98 Tax=Theria R	1	1	P62937		0	2.22E-07	2	916.96411	1832.92095	0.51
TLAQLNPESSLFIIASK	Glucose-6-phosphate isomerase n=2 Tax=Catarrhini Re	3	1	B4DVJ0		0	9.00E-09	2	916.98438	1832.96147	0.47
TLQGLEIELQSQLSmK	GUCA1B protein (Fragment) n=1 Tax=Homo sapiens Re	2	2	A4FUA1;P13646	M15(Oxidation)	0	9.39E-10	2	917.64233	1834.27739	0.16
LAALNPESNTAGLDIFAK	Chloride intracellular channel protein 1 n=24 Tax=Coelo	1	1	O00299		0	0.0275	2	923.56055	1846.11382	0.57
GDWQNEVNVRDFIQK	formate acetyltransferase [Enterobacter hormaechei A	2	2	334122625;206577432		0	1.24E-05	2	924.85034	1848.69341	0.4
TLNDMRQEYEQLIAK	Keratin, type I cytoskeletal 9 n=4 Tax=Catarrhini RepID=	1	1	P35527		0	6.83E-05	2	926.9624	1852.91753	0.5
FQDGDLTLYQSNTILR	Uncharacterized protein n=1 Tax=Homo sapiens RepID=	2	1	A8MX94		0	1.87E-12	2	942.99072	1884.97417	0.51
ILNEMRDQYEKMAEK	Keratin, type I cytoskeletal 14 n=8 Tax=Eutheria RepID=	1	1	P02533		0	0.0052	2	949.98169	1898.9561	0.52
ITEQDAQEMIDHLVMK	formate acetyltransferase [Enterobacter hormaechei A	1	1	334122625		0	1.92E-06	2	951.38745	1901.76763	0.43
ITMQNLNDRLASYLEK	Keratin, type I cytoskeletal 13 n=13 Tax=Simiiformes Re	1	1	P13646		0	6.42E-05	2	955.52649	1910.0457	0.53
KVPDPVLEESFQQLQR	Periplakin n=3 Tax=Hominoidea RepID=PEPL_HUMAN	1	1	O60437		0	7.34E-10	2	957.62183	1914.23638	0.61
DAEEWFFTKTEELNR	Keratin, type I cytoskeletal 14 n=8 Tax=Eutheria RepID=	1	1	P02533		0	1.46E-07	2	958.29883	1915.59038	0.35
WKEGEATLAPSLDLVGKI	alkyl hydroperoxide reductase subunit C [Klebsiella pne	3	1	206577586		0	4.35E-10	2	964.65027	1928.29326	0.62
AFTSEEFTHFLEELTK	phosphopyruvate hydratase [Escherichia coli CFT073]	1	1	26249182		0	1.26E-07	2	965.48108	1929.95488	0.51
LQVELDNVTGLLSQSDSK	Myosin-9 n=67 Tax=Tetrapoda RepID=MYH9_HUMAN	1	1	P35579		0	1.88E-11	2	973.84192	1946.67656	0.33

Sequence	Protein Descriptions	# Proteins	# Protein Groups	Protein Group Accessions	Modifications	q-Value	PEP	Charge	m/z [Da]	MH+ [Da]	Delta Mass [Da]
RVELEAALQQAKEELAR	cDNA FLJ55805, highly similar to Keratin, type II cytoske	2	1	B4DRW1		0	4.09E-13	2	977.91992	1954.83257	0.38
ELLSQYDFPGDDTPIVR	translation elongation factor Tu [Enterobacter hormaec	2	1	334126439		0	2.39E-08	2	983.00964	1965.01201	0.03
LDSELKNMQDMVEDYR	Keratin, type II cytoskeletal 1 n=7 Tax=Eutheria RepID=	1	1	P04264		0	2.40E-06	2	993.95605	1986.90483	0.51
THNLEPYFESFINNLR	Keratin, type II cytoskeletal 1 n=7 Tax=Eutheria RepID=	1	1	P04264		0	7.73E-07	2	997.97949	1994.95171	0.49
EIELEDKFENMGAQMVK	chaperone GroEL [Enterobacter hormaechei ATCC 4916	2	2	334123809;197286390		0	2.41E-07	2	1006.45813	2011.90898	0.48
ILINTPASQGGIGDLYNFK	bifunctional acetaldehyde-CoA/alcohol dehydrogenase	4	1	197285345		0	5.75E-14	2	1011.48279	2021.9583	0.44
DWLQAIESVIREEGVER	pyruvate dehydrogenase (acetyl-transferring), homodir	3	1	206576843		0	1.92E-06	3	677.44177	2030.31076	0.43
AMAGLGQPLPQEELDAMIR	Calmodulin-like protein 5 n=4 Tax=Hominidae RepID=C	1	1	Q9NZT1		0	0.00011	2	1020.89868	2040.79009	0.38
SAELNKEVSTNTAMIQTSK	GUCA1B protein (Fragment) n=1 Tax=Homo sapiens Re	2	2	A4FUA1;P13646		0	2.29E-11	2	1026.96497	2052.92266	0.45
VLDSEKLLYVLEADAAIAK	Periplakin n=3 Tax=Hominoidea RepID=PEPL_HUMAN	1	1	O60437		0	1.53E-06	2	1031.72595	2062.44463	0.65
GDRSEDFGVNEDLADSDAR	Annexin A1 n=14 Tax=Eutheria RepID=ANXA1_HUMAN	1	1	P04083		0	3.28E-12	2	1034.8761	2068.74492	0.43
SAELNKEVSTNTAmIQTSK	GUCA1B protein (Fragment) n=1 Tax=Homo sapiens Re	2	2	A4FUA1;P13646	M14(Oxidation)	0	2.44E-11	2	1035.03198	2069.05669	0.52
VLGDKIQLVGDDLFVTNTK	phosphopyruvate hydratase [Escherichia coli CFT073]	1	1	26249182		0	2.75E-06	2	1038.73987	2076.47246	0.67
SGETEDATIADLAVGTAAGQIK	phosphopyruvate hydratase [Escherichia coli CFT073]	1	1	26249182		0	1.36E-09	2	1060.07532	2119.14336	0.54
FNNFINDSLLEGAVDALER	6,7-dimethyl-8-ribityllumazine synthase [Proteus mirab	1	1	197283995		0	0.0811	3	713.28564	2137.84238	0.26
QAWFIENEEQEYVQTVK	Annexin A1 n=14 Tax=Eutheria RepID=ANXA1_HUMAN	1	1	P04083		0	3.17E-10	2	1071.49963	2141.99199	0.49
NKLNDLEDALQQAKEDLAR	Keratin, type II cytoskeletal 1 n=7 Tax=Eutheria RepID=	1	1	P04264		0	5.23E-13	2	1092.97839	2184.94951	0.41
NYTPYEGDESFLAGATDATTK	formate acetyltransferase [Enterobacter hormaechei A	1	1	334122625		0	1.27E-13	2	1126.57434	2252.14141	0.57
NVVLDKSFGAPTITKDGVSVAR	chaperone GroEL [Enterobacter hormaechei ATCC 4916	1	1	334123809		0	4.53E-09	3	759.16864	2275.49137	0.42
AEAESLYQSKYEELQITAGR	Keratin, type II cytoskeletal 1 n=7 Tax=Eutheria RepID=	1	1	P04264		0	2.56E-09	2	1144.06421	2287.12114	0.5
TDLEMQIESLNEELAYMKK	Keratin, type I cytoskeletal 13 n=13 Tax=Simiiformes Re	1	1	P13646		0	2.76E-06	3	762.80658	2286.40519	0.43
EMLLDAMENPEKYPQLTIR	formate acetyltransferase [Klebsiella pneumoniae 342]	1	1	206577432		0	2.14E-06	2	1146.54163	2292.07598	0.47
AANDDLLNSFWLLDSEKGEAR	autonomous glycyl radical cofactor GrcA [Escherichia c	2	1	161486147		0	2.27E-11	2	1183.07288	2365.13848	0.5
GGGGGGYGSGGSSYGSGGGSYGSGGGGGGGR	Keratin, type II cytoskeletal 1 n=7 Tax=Eutheria RepID=	1	1	P04264		0	1.70E-17	2	1192.90918	2384.81108	0.43
SGEDIFGDGVLEILQDGFGFLR	transcription termination factor Rho [Proteus mirabilis	4	1	197287127		0	1.03E-10	2	1192.9939	2384.98052	0.4
VVINKDTTTIIDGVGEEAAIQGR	chaperone GroEL [Enterobacter hormaechei ATCC 4916	1	1	334123809		0	2.75E-12	2	1200.57678	2400.14629	0.43
NLDLDSIIAEVKAQYEEIAQR	Uncharacterized protein n=4 Tax=Simiiformes RepID=F	1	1	F6RMN8		0	1.48E-10	2	1210.02209	2419.03691	0.39
SKAEAESLYQSKYEELQITAGR	Keratin, type II cytoskeletal 1 n=7 Tax=Eutheria RepID=	1	1	P04264		0	5.86E-11	2	1251.42273	2501.83818	0.29
RVVINKDTTTIIDGVGEEAAIQGR	chaperone GroEL [Enterobacter hormaechei ATCC 4916	1	1	334123809		0	4.54E-09	3	852.92786	2556.76902	0.46
VDQLSNDVNAmRSDVQAAKDDAAR	major outer membrane lipoprotein [Escherichia coli CF	2	1	26247927	M11(Oxidation)	0	0.000798	3	869.52112	2606.5488	0.44
TLTIADNGIGMNRDEVIDHLGTIAK	chaperone HtpG [Enterobacter hormaechei ATCC 4916	1	1	334123123		0	0.0115	3	890.22485	2668.66001	0.43
NYSPYYNTIDDLKDQIVDLTVGNNK	Keratin, type I cytoskeletal 9 n=4 Tax=Catarrhini RepID=	1	1	P35527		0	5.38E-06	3	968.46912	2903.3928	0.33
AIAQVGTISANSDETVGKLIAEAMDKVGK	chaperone GroEL [Enterobacter hormaechei ATCC 4916	1	1	334123809		0	3.53E-06	3	973.11487	2917.33005	0.27
AIAQVGTISANSDETVGTLIAQAmEKVGK	60 Kda chaperonin [Proteus mirabilis HI4320]	1	1	197286390	M24(Oxidation)	0	0.0872	3	973.41028	2918.21628	-0.1
TIDDLKNQILNLTTDNANILLQIDNAR	Keratin, type I cytoskeletal 10 n=12 Tax=Eutheria RepID	1	1	P13645		0	1.52E-10	3	1018.59094	3053.75827	0.38
VGKEGVITVEDGTGLEDELDVVEGMQFDR	chaperone GroEL [Enterobacter hormaechei ATCC 4916	1	1	334123809		0	2.19E-10	3	1046.57861	3137.72129	0.41
VLDELTLSKTDLEmQIESLNEELAYMKK	Keratin, type I cytoskeletal 13 n=13 Tax=Simiiformes Re	1	1	P13646	M14(Oxidation)	0	2.25E-13	3	1101.00439	3300.99863	0.45
AGEGNYGYNAATEEYGNMIDFGILDPTKVTR	chaperone GroEL [Enterobacter hormaechei ATCC 4916	1	1	334123809		0	1.75E-13	3	1123.30396	3367.89731	0.45

2DLCSCI_39 Protein Summary

index	Prot_hit	Accession	Description	Mascot Score	Coverage	# Peptides	# PSMs	# AAs	MW [kDa]	calc. pI
1	1.1	206579217	ribosomal protein L2 [Klebsiella pneumo	96.33	8.06%	2	2	273	29.8	10.93
2	1.2	334125920	50S ribosomal protein L2 [Enterobacter	96.33	8.06%	2	2	273	29.8	10.93
3	2.1	206578965	ribosomal protein L29 [Klebsiella pneum	95.74	34.92%	2	2	63	7.2	10.27
4	2.2	334125915	50S ribosomal protein L29 [Enterobacter	95.74	34.92%	2	2	63	7.2	10.59
5	3	206577508	outer membrane protein A [Klebsiella pı	208.59	7.43%	2	5	350	37.4	6.25
6	4.1	206580559	ribosomal protein S1 [Klebsiella pneumo	235.33	10.41%	3	4	557	61.1	4.98
7	4.2	334122618	30S ribosomal protein S1 [Enterobacter	235.33	10.41%	3	4	557	61.2	4.98
8	4.3	26246936	30S ribosomal protein S1 [Escherichia co	235.33	10.41%	3	4	557	61.1	4.98
9	5.1	206577586	alkyl hydroperoxide reductase subunit C	82.84	8.56%	1	1	187	20.8	5.17
10	5.2	334122926	alkyl hydroperoxide reductase C [Entero	82.84	7.96%	1	1	201	22.4	5.02
11	5.3	26246586	alkyl hydroperoxide reductase subunit C	82.84	8.56%	1	1	187	20.7	5.17
12	6.1	206575727	trigger factor [Klebsiella pneumoniae 34	81.04	6.25%	2	2	432	48	4.89
13	6.2	334123168	trigger factor [Enterobacter hormaechei	81.04	6.25%	2	2	432	48.1	4.87
14	6.3	26246446	trigger factor [Escherichia coli CFT073]	81.04	6.25%	2	2	432	48.2	4.86
15	7	206577504	ribosomal protein L9 [Klebsiella pneumo	117.33	20.13%	2	5	149	15.7	6.58
16	8	206579361	ribosomal protein S6 [Klebsiella pneumo	302.23	35.11%	4	7	131	15.1	5.38
17	9	206581075	translation elongation factor Tu [Klebsie	381.16	16.50%	4	8	394	43.2	5.53
18	10.1	197287068	50S ribosomal protein L3 [Proteus mirab	260.45	9.09%	2	6	209	22.5	9.86
19	10.2	334125923	50S ribosomal protein L3 [Enterobacter	260.45	9.09%	2	6	209	22.2	9.82
20	10.3	206576036	ribosomal protein L3 [Klebsiella pneumo	260.45	9.09%	2	6	209	22.2	9.91
21	10.4	26249915	50S ribosomal protein L3 [Escherichia co	260.45	9.09%	2	6	209	22.2	9.91
22	11.1	197287073	50S ribosomal protein L22 [Proteus mira	73.54	22.73%	1	1	110	12.1	10.18
23	11.2	334125918	50S ribosomal protein L22 [Enterobacter	73.54	22.73%	1	1	110	12.2	10.18
24	12.1	197287127	transcription termination factor Rho [Pro	98.47	5.25%	1	1	419	46.9	7.25
25	12.2	334126167	transcription termination factor Rho [En	98.47	5.25%	1	1	419	47	7.25
26	12.3	206576949	transcription termination factor Rho [Kle	98.47	5.25%	1	1	419	47	7.25
27	12.4	26250521	transcription termination factor Rho [Esc	98.47	4.97%	1	1	443	49.6	7.3
28	13.1	26247927	major outer membrane lipoprotein [Escl	182.06	30.77%	2	4	78	8.3	9.25
29	13.2	334122238	major outer membrane lipoprotein [Ente	182.06	30.77%	2	4	78	8.4	9.31
30	14	161486111	ATP-dependent RNA helicase DeaD [Escl	89.92	2.70%	1	1	629	70.5	8.72
31	15.1	26249880	DNA-directed RNA polymerase subunit a	68.74	7.29%	1	1	329	36.5	5.06
32	15.2	334125900	DNA-directed RNA polymerase subunit a	68.74	7.29%	1	1	329	36.4	5.06
33	16	P04264	Keratin, type II cytoskeletal 1 n=7 Tax=Eu	108.01	4.81%	1	1	644	66	8.12
34	17.1	P08727	Keratin, type I cytoskeletal 19 n=9 Tax=E	209.23	8.75%	2	3	400	44.1	5.14
35	17.2	B4DE59	cDNA FLJ60424, highly similar to Junctio	209.23	6.22%	2	3	563	62.6	5.17
36	18	O60437	Periplakin n=3 Tax=Hominoidea RepID=I	82.43	0.85%	1	1	1756	204.6	5.6
37	19.1	E9PFJ3	Uncharacterized protein n=3 Tax=Simiifo	101.07	2.77%	1	1	578	63.3	5.31
38	19.2	P07911	Uromodulin n=2 Tax=Catarrhini RepID=U	101.07	2.50%	1	1	640	69.7	5.24
39	19.3	Q5R5C1	Uromodulin n=8 Tax=Simiiformes RepID	101.07	2.50%	1	1	641	69.8	5.24
40	20	A4FUA1	GUCA1B protein (Fragment) n=1 Tax=Ho	475.45	58.67%	3	8	75	8.6	5.41
41	21.1	P13646-3	Isoform 3 of Keratin, type I cytoskeletal	685.04	22.86%	8	21	420	45.8	4.88
42	21.2	P13646	Keratin, type I cytoskeletal 13 n=13 Tax=	685.04	20.96%	8	21	458	49.6	4.96
43	22.1	B4DRW1	cDNA FLJ55805, highly similar to Keratin	570.84	20.46%	8	17	474	51.7	6.81
44	22.2	P19013	Keratin, type II cytoskeletal 4 n=14 Tax=S	570.84	18.16%	8	17	534	57.2	6.61
45	23.1	E7EUE8	Uncharacterized protein n=14 Tax=Euthe	622.1	16.54%	8	20	520	55.8	6.27
46	23.2	P02538	Keratin, type II cytoskeletal 6A n=25 Tax	622.1	15.25%	8	20	564	60	8
47	24.1	C9JZN1	Uncharacterized protein n=5 Tax=Catarr	84.39	11.84%	2	3	152	17	9.13
48	24.2	P62879	Guanine nucleotide-binding protein G(I)	84.39	5.29%	2	3	340	37.3	6
49	25	P04792	Heat shock protein beta-1 n=6 Tax=Simi	92.57	16.10%	2	2	205	22.8	6.4
50	26.1	E7EU87	Uncharacterized protein n=3 Tax=Simiifo	414.57	8.47%	4	11	555	58.9	5.97
51	26.2	P13647	Keratin, type II cytoskeletal 5 n=17 Tax=I	414.57	7.97%	4	11	590	62.3	7.74
52	27	P04083	Annexin A1 n=14 Tax=Eutheria RepID=A	688.06	31.79%	8	13	346	38.7	7.02
53	28.1	Q96KK5	Histone H2A type 1-H n=10 Tax=Mamma	79.38	14.06%	2	2	128	13.9	10.89
54	28.2	P0C0S8	Histone H2A type 1 n=295 Tax=Eukaryot	79.38	13.85%	2	2	130	14.1	10.9
55	28.3	Q16777	Histone H2A type 2-C n=33 Tax=Eumeta	79.38	13.95%	2	2	129	14	10.9
56	28.4	C9JE22	Histone H2A n=7 Tax=Eutheria RepID=C9	79.38	11.92%	2	2	151	16.1	10.23
57	28.5	Q99878	Histone H2A type 1-J n=4 Tax=Eutheria F	79.38	14.06%	2	2	128	13.9	10.89
58	29	Q9UBG3	Cornulin n=6 Tax=Catarrhini RepID=CRN	125.62	3.23%	1	2	495	53.5	6.1

2DLCSCI_39 Peptide Summary

Sequence	Protein Descriptions	# Proteins	# Protein Groups	Protein Group Accessions	Modifications	q-Value	PEP	Charge	m/z [Da]	MH+ [Da]	Delta Mass [Da]
SLYGLGGSK	Uncharacterized protein n=14 Tax=Eutheria F	2	1	E7EUE8		0	0.00274	2	441.53241	882.05754	0.29
aMVSEFLK	Annexin A1 n=14 Tax=Eutheria RepID=ANXA1	1	1	P04083	N-Term(Acetyl)	0.00497	0.248	1	966.59521	966.59521	0.1
YENELALR	Isoform 3 of Keratin, type I cytoskeletal 13 n=	2	1	P13646-3		0	0.0252	2	504.69507	1008.38286	0.43
EQYEAMAER	Isoform 3 of Keratin, type I cytoskeletal 13 n=	2	1	P13646-3		0	0.163	2	564.1687	1127.33013	0.42
DIADAVTAAGVK	ribosomal protein L9 [Klebsiella pneumoniae	1	1	206577504		0	0.00619	1	1130.72339	1130.72339	0.12
DAEEWFHAK	GUCA1B protein (Fragment) n=1 Tax=Homo s	1	1	A4FUA1		0	0.0688	2	566.9455	1132.88371	0.19
HAVTEASPMVK	ribosomal protein S6 [Klebsiella pneumoniae	1	1	206579361		0	0.00204	2	585.7829	1170.55852	0.48
WTLLQEQGTK	Uncharacterized protein n=14 Tax=Eutheria F	5	2	E7EUE8;E7EU87		0	0.0115	2	602.77625	1204.54521	0.45
SLEEAGAEVEVK	ribosomal protein L7/L12 [Klebsiella pneumo	2	1	206576265		0	0.00509	1	1260.54688	1260.54688	-0.08
NDEELNKLLGK	Histone H2A type 1-H n=10 Tax=Mammalia R	5	1	Q96KK5		0	0.00476	2	637.31714	1273.627	0.47
VGTVTPNVAEAVK	50S ribosomal protein L1 [Proteus mirabilis H	4	1	197286617		0	0.00104	2	642.99603	1284.98479	0.13
ALEEANADLEVK	Isoform 3 of Keratin, type I cytoskeletal 13 n=	2	1	P13646-3		0	0.000628	1	1301.65759	1301.65759	0
QLGEDPWVAIAK	ribosomal protein S1 [Klebsiella pneumoniae	3	1	206580559		0	0.0231	2	664.40265	1327.79802	0.55
NLDLDSIIAEVK	Uncharacterized protein n=14 Tax=Eutheria F	5	2	E7EUE8;E7EU87		0	1.24E-05	2	665.55933	1330.11138	0.19
TAAENDFVVLKK	cDNA FLJ55805, highly similar to Keratin, typ	2	1	B4DRW1		0	0.000535	2	668.38794	1335.7686	0.52
SKAEAEALYQTK	cDNA FLJ55805, highly similar to Keratin, typ	4	1	B4DRW1		0	0.0016	2	670.14417	1339.28105	0.3
TAAENEFVTLKK	Uncharacterized protein n=14 Tax=Eutheria F	3	1	E7EUE8		0	0.00227	2	676.35681	1351.70635	0.49
RVELEAALQQAK	cDNA FLJ55805, highly similar to Keratin, typ	2	1	B4DRW1		0	0.0489	2	678.86151	1356.71575	0.48
NLDLDSIIAEVR	cDNA FLJ55805, highly similar to Keratin, typ	2	1	B4DRW1		0	1.40E-05	2	679.81299	1358.6187	0.44
NKLEGLEDALQK	Uncharacterized protein n=14 Tax=Eutheria F	3	1	E7EUE8		0	0.000106	2	679.78021	1358.55315	0.41
VDQLSNDVNAMR	major outer membrane lipoprotein [Escheric	2	1	26247927		0	6.49E-05	2	681.49469	1361.9821	0.17
EKPTWLEVDAGK	ribosomal protein S4 [Klebsiella pneumoniae	3	1	206575742		0	0.0481	2	687.21692	1373.42656	0.36
AGDQIQSGVDAAIK	ribosomal protein L2 [Klebsiella pneumoniae	2	1	206579217		0	0.0055	2	687.34308	1373.67888	0.49
TRLEQEIATYR	Isoform 3 of Keratin, type I cytoskeletal 13 n=	2	1	P13646-3		0	0.0419	2	690.87256	1380.73784	0.51
YTGAITAAAGTIHR	ribosomal protein S6 [Klebsiella pneumoniae	1	1	206579361		0	0.000395	2	702.39746	1403.78765	0.52
ELDKLENSLGGIK	ribosomal protein S2 [Klebsiella pneumoniae	2	1	206581012		0	0.00387	2	708.4624	1415.91753	0.07
DGYADGWAQAGTAR	50S ribosomal protein L6 [Escherichia coli CF	1	1	26249892		0	0.000111	2	720.25732	1439.50737	0.44
SRAEAESWYQTK	Uncharacterized protein n=14 Tax=Eutheria F	3	1	E7EUE8		0	0.0599	2	728.50659	1456.00591	0.16
IGSEAYNQQLSEK	outer membrane protein A [Klebsiella pneum	1	1	206577508		0	0.00225	2	734.30579	1467.6043	0.45
ELPELTEEFIKR	trigger factor [Klebsiella pneumoniae 342]	3	1	206575727		0	0.00277	2	752.87634	1504.74541	0.47
GVDEATIIDILTKR	Annexin A1 n=14 Tax=Eutheria RepID=ANXA1	1	1	P04083		0	7.51E-06	2	772.92676	1544.84624	0.49
NVALEEQAVEAVLAK	trigger factor [Klebsiella pneumoniae 342]	3	1	206575727		0	0.0376	2	793.08044	1585.15361	0.65
VGEEVEIVGIKETAK	translation elongation factor Tu [Klebsiella pr	1	1	206581075		0	2.98E-05	2	801.42517	1601.84307	0.48
ALTGHLEEVVLALLK	Annexin A1 n=14 Tax=Eutheria RepID=ANXA1	1	1	P04083		0	9.45E-05	2	803.97571	1606.94414	0.49
EGEATLAPSLDLVGKI	alkyl hydroperoxide reductase subunit C [Kle	3	1	206577586		0	5.12E-05	2	807.36792	1613.72856	0.42
WNLLQQQTTTTSSK	cDNA FLJ55805, highly similar to Keratin, typ	2	1	B4DRW1		0	2.07E-05	2	818.87439	1636.7415	0.45
DLAKDITSDTSGDFR	Annexin A1 n=14 Tax=Eutheria RepID=ANXA1	1	1	P04083		0	1.23E-06	2	821.36493	1641.72258	0.47
TPAQFDADELRAAMK	Annexin A1 n=14 Tax=Eutheria RepID=ANXA1	1	1	P04083		0	0.000336	2	832.88086	1664.75444	0.47
DVDAAYLNKVELEAK	cDNA FLJ55805, highly similar to Keratin, typ	2	1	B4DRW1		0	0.00306	2	839.47668	1677.94609	0.04
GAVPGATGSDLIVKPAVK	50S ribosomal protein L3 [Proteus mirabilis H	4	1	197287068		0	0.000662	2	840.84314	1680.679	0.36
AAAFEGELIPASQIDR	ribosomal protein L10 [Klebsiella pneumonia	3	1	206577086		0	0.000104	2	844.71973	1688.43218	0.28
GLGTDEDTLIEILASR	Annexin A1 n=14 Tax=Eutheria RepID=ANXA1	1	1	P04083		0	5.93E-08	2	852.14136	1703.27544	0.19
DSTIQVVENGESSQGR	Uncharacterized protein n=3 Tax=Simiiforme	3	1	E9PFJ3		0	3.80E-06	2	853.79388	1706.58049	0.39
GAVPGATGSDLIVKPAVKA	50S ribosomal protein L3 [Proteus mirabilis H	4	1	197287068		0	5.62E-06	2	876.40491	1751.80254	0.4
VLDTKWTLLQEQGTK	Uncharacterized protein n=3 Tax=Simiiforme	3	1	E7EU87		0	4.87E-06	2	880.83618	1760.66509	0.35
sELEQLRQEAEQLR	Uncharacterized protein n=5 Tax=Catarrhini I	2	1	C9JZN1	N-Term(Acetyl)	0	0.041	2	886.47498	1771.94267	0.52
VSLDVNHFAPDELTVK	Heat shock protein beta-1 n=6 Tax=Simiiform	1	1	P04792		0	0.00415	2	892.79358	1784.57988	0.33
VPDPVLEESFQQLQR	Periplakin n=3 Tax=Hominoidea RepID=PEPL_	1	1	O60437		0	9.81E-06	2	893.41113	1785.81499	0.45
ISPQIQLSGQTEQTQK	Cornulin n=6 Tax=Catarrhini RepID=CRNN_HU	1	1	Q9UBG3		0	6.69E-06	2	893.79468	1786.58208	0.32
QSPASPERDYSPYYK	Isoform 3 of Keratin, type I cytoskeletal 13 n=	2	1	P13646-3		0	0.0704	2	894.80103	1788.59477	0.39
VELEAALQQAKEELAR	cDNA FLJ55805, highly similar to Keratin, typ	2	1	B4DRW1		0	0.000112	2	899.89709	1798.78691	0.41
GITINTSHVEYDTPTR	translation elongation factor Tu [Klebsiella pr	1	1	206581075		0	0.00295	2	902.83875	1804.67021	0.39
NGYNSAALNGDMNQALR	ATP-dependent RNA helicase DeaD [Escherich	1	1	161486111		0	2.84E-05	2	905.17029	1809.3333	0.25
TLQGLEIELQSQLSMK	Keratin, type I cytoskeletal 19 n=9 Tax=Euthe	5	3	P08727;A4FUA1;P13646-3		0	1.78E-06	2	909.95081	1818.89433	0.46
NLEPLFETYLSVLRK	cDNA FLJ55805, highly similar to Keratin, typ	2	1	B4DRW1		0	0.000334	2	911.89868	1822.79009	0.39
VLAEMREQYEAMAER	Isoform 3 of Keratin, type I cytoskeletal 13 n=	2	1	P13646-3		0	0.0244	2	913.71814	1826.429	0.29
VLAEmREQYEAMAER	Isoform 3 of Keratin, type I cytoskeletal 13 n=	2	1	P13646-3	M5(Oxidation)	0	0.0152	2	921.81592	1842.62456	0.39
VKHPSEIVNVGDEITVK	ribosomal protein S1 [Klebsiella pneumoniae	3	1	206580559		0	7.43E-07	2	932.87207	1864.73686	0.36
VFMQPASEGTGIIAGGAMR	30S ribosomal protein S5 [Enterobacter horm	2	1	334125906		0	0.0127	2	947.63501	1894.26274	0.66
LATQSNEITIPVTFESR	Heat shock protein beta-1 n=6 Tax=Simiiform	1	1	P04792		0	0.0257	2	953.89661	1906.78594	0.4
LSDLAHVEGDVVDLNALK	ribosomal protein L15 [Klebsiella pneumonia	1	1	206576059		0	0.000849	2	954.90845	1908.80962	0.4
ELLSQYDFPGDDTPIVR	translation elongation factor Tu [Klebsiella pr	1	1	206581075		0	5.66E-05	2	983.32861	1965.64995	0.35
IIELAGHLDTYIPEPER	translation elongation factor Tu [Klebsiella pr	1	1	206581075		0	1.52E-07	2	983.93958	1966.87187	0.42
DWLQAIESVIREEGVER	pyruvate dehydrogenase (acetyl-transferring	3	1	206576843		0	0.00868	2	1015.46167	2029.91606	0.44
TLEEEAKTHEAQIQEMR	Myosin-9 n=67 Tax=Tetrapoda RepID=MYH9_	1	1	P35579		0	0.0218	3	681.70728	2043.10727	0.04
SAELNKEVSTNTAMIQTSK	GUCA1B protein (Fragment) n=1 Tax=Homo s	3	2	A4FUA1;P13646-3		0	3.55E-06	2	1026.9679	2052.92851	0.45
GDRSEDFGVNEDLADSDAR	Annexin A1 n=14 Tax=Eutheria RepID=ANXA1	1	1	P04083		0	5.31E-07	2	1034.53076	2068.05425	0.08
SAELNKEVSTNTAmIQTSK	GUCA1B protein (Fragment) n=1 Tax=Homo s	3	2	A4FUA1;P13646-3	M14(Oxidation)	0	8.85E-06	2	1034.82556	2068.64385	0.31
NVGEHEVDFQVHSEVFAK	ribosomal protein L9 [Klebsiella pneumoniae	1	1	206577504		0	0.00286	3	691.24536	2071.72153	0.24
REDFANETADDSEAGDSEE	ribosomal protein S6 [Klebsiella pneumoniae	1	1	206579361		0	2.02E-10	2	1044.24744	2087.4876	0.35
VYAGNEHNHAAQQPQVLDI	50S ribosomal protein L13 [Escherichia coli C	3	1	161486103		0	0.000385	2	1052.84155	2104.67583	0.33
HLQLAIRNDEELNKLLGK	Histone H2A type 1-H n=10 Tax=Mammalia R	5	1	Q96KK5		0	0.00774	3	702.48535	2105.4415	0.42
AGDQIQSGVDAAIKAGNTLPMR	ribosomal protein L2 [Klebsiella pneumoniae	2	1	206579217		0	7.63E-05	3	738.76343	2214.27573	0.38
TEELNREVAGHTEQLQMSR	Keratin, type I cytoskeletal 19 n=9 Tax=Euthe	2	1	P08727		0	0.000562	3	743.7533	2229.24533	0.39
sELEQLRQEAEQLRNQIR	Uncharacterized protein n=5 Tax=Catarrhini I	2	1	C9JZN1	N-Term(Acetyl)	0	0.016	3	761.78979	2283.35483	0.39
AEAESWYQTKYEELQVTAGR	Uncharacterized protein n=14 Tax=Eutheria F	3	1	E7EUE8		0	9.35E-06	2	1180.40552	2359.80376	0.34
ERREDFANETADDSEAGDSEE	ribosomal protein S6 [Klebsiella pneumoniae	1	1	206579361		0	0.007	2	1186.86743	2372.72759	0.39
GGGGGGYGSGGSSYGSGGGSYGSGGGGGGGR	Keratin, type II cytoskeletal 1 n=7 Tax=Euther	1	1	P04264		0	3.56E-07	2	1192.86548	2384.72368	0.39
SGEDIFGDGVLEILQDGFGFLR	transcription termination factor Rho [Proteu	4	1	197287127		0	4.14E-07	2	1193.25354	2385.4998	0.66
SVEELNAELLNLLREQFNLR	ribosomal protein L29 [Klebsiella pneumonia	2	1	206578965		0	0.194	2	1201.09888	2401.19048	0.45
TEAESWYQTKYEELQQTAGR	Uncharacterized protein n=3 Tax=Simiiforme	2	1	E7EU87		0	1.06E-05	2	1209.87891	2418.75054	0.31
NLDLDSIIAEVKAQYEEIAQR	Uncharacterized protein n=14 Tax=Eutheria F	3	1	E7EUE8		0	7.72E-08	2	1210.05432	2419.10137	0.43
DLAKDITSDTSGDFRNALLSLAK	Annexin A1 n=14 Tax=Eutheria RepID=ANXA1	1	1	P04083		0	0.000392	3	818.00464	2451.99936	0.24
VDQLSNDVNAmRSDVQAAKDDAAR	major outer membrane lipoprotein [Escheric	2	1	26247927	M11(Oxidation)	0	1.17E-05	3	869.51355	2606.5261	0.43
KVLESAIANAEHNDGADIDDLKVAK	50S ribosomal protein L22 [Proteus mirabilis	2	1	197287073		0	6.93E-06	3	879.91919	2637.74302	0.46
EKSVEELNAELLNLLREQFNLR	ribosomal protein L29 [Klebsiella pneumonia	2	1	206578965		0	4.41E-06	3	886.89746	2658.67783	0.42
TDLDKLVIEMETNGTIDPEEAIRR	DNA-directed RNA polymerase subunit alpha	2	1	26249880		0	1.14E-05	3	920.43909	2759.30271	0.3
DGSAVVMGFTDRIGSEAYNQQLSEKR	outer membrane protein A [Klebsiella pneum	1	1	206577508		0	0.00216	3	953.50061	2858.48728	0.04
GVVVAIDKDVVLVDAGLKSESAIPAEQFK	ribosomal protein S1 [Klebsiella pneumoniae	3	1	206580559		0	7.93E-07	3	1000.32605	2998.9636	0.44

2DLCSCI_33 Protein Summary

index	Prot_hit	Accession	Description	Mascot Score	Coverage	# Peptides	# PSMs	# AAs	MW [kDa]	calc. pI
1	1	P35579	Myosin-9 n=67 Tax=Tetrapoda	442.23	4.29%	6	9	1960	226.4	5.6
2	2	P06702	Protein S100-A9 n=5 Tax=Homi	233.47	31.58%	2	4	114	13.2	6.13

2DLCSCI_33 Peptide Summary

Sequence	Protein Descriptions	# Proteins	# Protein Groups	Protein Group Accessions	Modifications	q-Value	PEP	Charge	m/z [Da]	MH+ [Da]	Delta Mass [Da]
RDLGEELEALK	Myosin-9 n=67 Tax=Tetrapoda RepID=MYH9_	1	1	P35579		0	0.291	2	637.50842	1274.00957	0.67
DSTIQVVENGESSQGR	Uncharacterized protein n=3 Tax=Simiiforme	3	1	E9PFJ3		0	0.257	2	853.94507	1706.88286	0.54
sELDQLRQEAEQLK	Guanine nucleotide-binding protein G(I)/G(S	1	1	P62873	N-Term(Acetyl)	0	0.0768	2	865.35803	1729.70879	0.42
VIEHIMEDLDTNADK	Protein S100-A9 n=5 Tax=Hominoidea RepID	1	1	P06702		0	0.058	2	872.52966	1744.05205	0.61
TELEDTLDSTAAQQELR	Myosin-9 n=67 Tax=Tetrapoda RepID=MYH9_	1	1	P35579		0	0.0111	2	960.93542	1920.86357	0.47
ELESQISELQEDLESER	Myosin-9 n=67 Tax=Tetrapoda RepID=MYH9_	1	1	P35579		0	0.00751	2	1017.77063	2034.53398	0.29
TLEEEAKTHEAQIQEMR	Myosin-9 n=67 Tax=Tetrapoda RepID=MYH9_	1	1	P35579		0	0.0186	2	1022.62573	2044.24419	0.63
GDRSEDFGVNEDLADSDAR	cDNA FLJ51887, highly similar to Annexin A1	2	1	B4DL19		0	0.00602	2	1035.06653	2069.12578	0.62
MHEGDEGPGHHHKPGLGEGTP	Protein S100-A9 n=5 Tax=Hominoidea RepID	1	1	P06702		0	0.0351	2	1088.93066	2176.85405	0.45
NKLNDLEDALQQAKEDLAR	Keratin, type II cytoskeletal 1 n=7 Tax=Euthe	1	1	P04264		0	0.0397	3	728.8902	2184.65604	0.18
MQQNIQELEEQLEEEESAR	Myosin-9 n=67 Tax=Tetrapoda RepID=MYH9_	1	1	P35579		0	0.0201	2	1167.60022	2334.19316	0.57
KMQQNIQELEEQLEEEESARQK	Myosin-9 n=67 Tax=Tetrapoda RepID=MYH9_	1	1	P35579		0	0.0304	3	906.82544	2718.46177	0.39

2DLCSCI_51 Protein Summary

index	Prot_hit	Accession	Description	Mascot Score	Coverage	# Peptides	# PSMs	# AAs	MW [kDa]	calc. pI
1	1.1	334126439	translation elongation factor Tu [Enterobacter hormaechei ATC(	423.76	17.55%	4	10	376	41.1	5.24
2	1.2	161486096	elongation factor Tu [Escherichia coli CFT073]	423.76	16.75%	4	10	394	43.3	5.45
3	1.3	26250749	elongation factor Tu [Escherichia coli CFT073]	423.76	16.75%	4	10	394	43.3	5.45
4	2.1	334125906	30S ribosomal protein S5 [Enterobacter hormaechei ATCC 4916	354.27	19.88%	2	6	166	17.5	10.05
5	2.2	206580716	ribosomal protein S5 [Klebsiella pneumoniae 342]	354.27	19.76%	2	6	167	17.6	10.11
6	3.1	334124951	phosphate acetyltransferase [Enterobacter hormaechei ATCC 4!	161.45	4.49%	2	3	713	77	5.48
7	3.2	206580523	phosphate acetyltransferase [Klebsiella pneumoniae 342]	161.45	4.48%	2	3	715	77.2	5.47
8	3.3	26248686	phosphate acetyltransferase [Escherichia coli CFT073]	161.45	4.48%	2	3	714	77.2	5.41
9	4.1	334124672	flagellin [Enterobacter hormaechei ATCC 49162]	561.9	8.44%	3	8	569	58.3	4.92
10	4.2	334125162	bacterial flagellin subfamily protein [Enterobacter hormaechei /	561.9	7.15%	3	8	671	68.4	4.84
11	5	334123809	chaperone GroEL [Enterobacter hormaechei ATCC 49162]	1441.12	35.95%	11	28	548	57.1	4.94
12	6.1	334123523	chaperone DnaK [Enterobacter hormaechei ATCC 49162]	110.62	4.40%	2	2	637	69.1	4.96
13	6.2	206580041	chaperone protein DnaK [Klebsiella pneumoniae 342]	110.62	4.39%	2	2	638	69.1	4.97
14	6.3	26245936	molecular chaperone DnaK [Escherichia coli CFT073]	110.62	4.39%	2	2	638	69.1	4.97
15	7	334123429	dihydrolipoyl dehydrogenase [Enterobacter hormaechei ATCC 4	273.02	13.68%	3	6	475	50.7	6.16
16	8	334122958	ferrienterobactin receptor [Enterobacter hormaechei ATCC 491	312.97	10.46%	4	9	746	82.6	5.67
17	9	334122625	formate acetyltransferase [Enterobacter hormaechei ATCC 491(	194.6	8.95%	4	6	760	85	6.09
18	10.1	206580744	ribosomal protein S3 [Klebsiella pneumoniae 342]	201.74	5.17%	1	3	232	25.8	10.3
19	10.2	334125917	30S ribosomal protein S3 [Enterobacter hormaechei ATCC 4916	201.74	5.15%	1	3	233	26	10.27
20	10.3	197287074	30S ribosomal protein S3 [Proteus mirabilis HI4320]	201.74	5.15%	1	3	233	25.9	10.21
21	11.1	206579163	ribosomal protein L5 [Klebsiella pneumoniae 342]	84.35	8.38%	1	1	179	20.3	9.39
22	11.2	334125911	50S ribosomal protein L5 [Enterobacter hormaechei ATCC 4916:	84.35	8.38%	1	1	179	20.3	9.39
23	11.3	26249896	50S ribosomal protein L5 [Escherichia coli CFT073]	84.35	8.38%	1	1	179	20.3	9.48
24	12.1	206579040	polyribonucleotide nucleotidyltransferase [Klebsiella pneumoni	291.6	3.94%	2	4	711	76.8	5.17
25	12.2	334125777	polyribonucleotide nucleotidyltransferase [Enterobacter horma	291.6	3.93%	2	4	712	77	5.19
26	12.3	26249747	polynucleotide phosphorylase/polyadenylase [Escherichia coli C	291.6	3.78%	2	4	740	80.5	5.54
27	13.1	206576322	colicin I receptor [Klebsiella pneumoniae 342]	86	3.35%	2	2	657	73.1	5.41
28	13.2	229106376	colicin I receptor [Escherichia coli CFT073]	86	3.32%	2	2	663	73.8	5.29
29	14.1	206580341	threonyl-tRNA synthetase [Klebsiella pneumoniae 342]	66.54	2.02%	1	1	642	73.9	6.23
30	14.2	26247970	threonyl-tRNA synthetase [Escherichia coli CFT073]	66.54	2.02%	1	1	642	74	6.19
31	15	206577432	formate acetyltransferase [Klebsiella pneumoniae 342]	174.34	7.89%	4	5	760	85.2	5.85
32	16.1	206580931	fimbrillin MatB [Klebsiella pneumoniae 342]	146.23	9.23%	1	2	195	20	4.88
33	16.2	26246302	hypothetical protein c0404 [Escherichia coli CFT073]	146.23	9.23%	1	2	195	20.1	4.88
34	17.1	206577768	dihydrolipoyllysine-residue acetyltransferase [Klebsiella pneum	169.35	8.89%	3	4	630	65.7	5.31
35	17.2	334123431	pyruvate dehydrogenase complex E2, dihydrolipoamide acetyltı	169.35	8.85%	3	4	633	65.9	5.15
36	18.1	206578905	DNA-directed RNA polymerase, beta' subunit [Klebsiella pneum	219.1	4.76%	4	5	1407	155.3	6.8
37	18.2	26250759	DNA-directed RNA polymerase subunit beta' [Escherichia coli Cl	219.1	4.76%	4	5	1407	155.1	7.08
38	19.2	206579488	DNA-directed RNA polymerase, beta subunit [Klebsiella pneumo	291.18	5.96%	4	6	1342	150.4	5.29
39	19.2	334123945	DNA-directed RNA polymerase subunit beta [Enterobacter horn	291.18	5.91%	4	6	1353	151.6	5.27
40	19.3	26250758	DNA-directed RNA polymerase subunit beta [Escherichia coli CF	291.18	5.91%	4	6	1353	151.8	5.27
41	20.1	206577086	ribosomal protein L10 [Klebsiella pneumoniae 342]	215.36	29.09%	3	5	165	17.8	8.98
42	20.2	334123947	50S ribosomal protein L10 [Enterobacter hormaechei ATCC 491(	215.36	29.09%	3	5	165	17.8	8.98
43	20.3	26250755	50S ribosomal protein L10 [Escherichia coli CFT073]	215.36	29.09%	3	5	165	17.7	8.98
44	21.1	206577888	ribosomal protein L11 [Klebsiella pneumoniae 342]	138.7	9.86%	1	2	142	14.9	9.63
45	21.2	26250753	50S ribosomal protein L11 [Escherichia coli CFT073]	138.7	9.86%	1	2	142	14.9	9.63
46	22.1	206580919	ATP synthase F1, alpha subunit [Klebsiella pneumoniae 342]	229.87	9.16%	3	6	513	55.1	6
47	22.2	26250478	F0F1 ATP synthase subunit alpha [Escherichia coli CFT073]	229.87	9.16%	3	6	513	55.2	6.13
48	23	197286390	60 Kda chaperonin [Proteus mirabilis HI4320]	276.5	12.59%	4	7	548	57.6	4.89
49	24.1	197286835	glutamate dehydrogenase [Proteus mirabilis HI4320]	114.74	4.48%	1	1	446	48.4	7.03
50	24.2	334122327	glutamate dehydrogenase [Enterobacter hormaechei ATCC 491(	114.74	4.47%	1	1	447	48.3	6.62
51	24.3	206577927	NADP-specific glutamate dehydrogenase [Klebsiella pneumonia	114.74	4.47%	1	1	447	48.5	7.01
52	24.4	26248016	glutamate dehydrogenase [Escherichia coli CFT073]	114.74	4.47%	1	1	447	48.5	6.4
53	25.1	197287068	50S ribosomal protein L3 [Proteus mirabilis HI4320]	79.69	9.09%	1	1	209	22.5	9.86
54	25.2	334125923	50S ribosomal protein L3 [Enterobacter hormaechei ATCC 4916:	79.69	9.09%	1	1	209	22.2	9.82
55	25.3	206576036	ribosomal protein L3 [Klebsiella pneumoniae 342]	79.69	9.09%	1	1	209	22.2	9.91
56	25.4	26249915	50S ribosomal protein L3 [Escherichia coli CFT073]	79.69	9.09%	1	1	209	22.2	9.91
57	26.1	197287076	50S ribosomal protein L29 [Proteus mirabilis HI4320]	157.97	25.40%	1	2	63	7.3	10.27
58	26.2	26249901	50S ribosomal protein L29 [Escherichia coli CFT073]	157.97	25.40%	1	2	63	7.3	9.99
59	27.1	197287093	DNA-directed RNA polymerase subunit alpha [Proteus mirabilis	97	8.21%	2	2	329	36.5	5.11
60	27.2	334125900	DNA-directed RNA polymerase subunit alpha [Enterobacter hor	97	8.21%	2	2	329	36.4	5.06
61	27.3	26249880	DNA-directed RNA polymerase subunit alpha [Escherichia coli C	97	8.21%	2	2	329	36.5	5.06
62	28.1	197287189	50S ribosomal protein L9 [Proteus mirabilis HI4320]	106.9	9.40%	1	2	149	15.8	6.32
63	28.2	334123750	5-methyltetrahydropteroyltriglutamate--homocysteine S-methy	106.9	9.40%	1	2	149	15.6	7.42
64	28.3	206577504	ribosomal protein L9 [Klebsiella pneumoniae 342]	106.9	9.40%	1	2	149	15.7	6.58
65	28.4	26251101	50S ribosomal protein L9 [Escherichia coli CFT073]	106.9	9.40%	1	2	149	15.8	6.58
66	29.1	197287224	cell division protein [Proteus mirabilis HI4320]	79.43	2.17%	1	1	646	70.9	5.83
67	29.2	334125787	ATP-dependent metallopeptidase HflB [Enterobacter hormaech	79.43	2.16%	1	1	647	71	6.11
68	29.3	206578792	ATP-dependent metallopeptidase HflB [Klebsiella pneumoniae 3	79.43	2.16%	1	1	647	71.1	6.11
69	30	15596155	porin [Pseudomonas aeruginosa PAO1]	181.37	4.06%	1	4	443	48.3	5.16
70	31	15596170	peptidoglycan associated lipoprotein OprL precursor [Pseudom	158.98	21.43%	2	2	168	17.9	6.34
71	32	15596289	flagellin type B [Pseudomonas aeruginosa PAO1]	842.98	21.11%	6	13	488	49.2	5.62
72	33	15596783	dihydrolipoamide succinyltransferase [Pseudomonas aeruginos	73.53	4.65%	1	1	409	42.9	5.55

index	Prot_hit	Accession	Description	Mascot Score	Coverage	# Peptides	# PSMs	# AAs	MW [kDa]	calc. pI
73	34	15596974	porin [Pseudomonas aeruginosa PAO1]	621.64	21.43%	5	13	350	37.6	5.14
74	35	15597001	DNA-binding protein HU [Pseudomonas aeruginosa PAO1]	102.46	25.56%	2	2	90	9.1	9.99
75	36	15598162	acyl carrier protein [Pseudomonas aeruginosa PAO1]	105.24	20.51%	1	2	78	8.7	4.12
76	37	15598965	inosine 5'-monophosphate dehydrogenase [Pseudomonas aeru	201.18	4.09%	1	3	489	51.7	6.68
77	38.1	15599445	30S ribosomal protein S8 [Pseudomonas aeruginosa PAO1]	145.76	9.23%	1	4	130	14.2	9.57
78	38.2	334125909	30S ribosomal protein S8 [Enterobacter hormaechei ATCC 4916	145.76	9.23%	1	4	130	14.1	9.39
79	38.3	206580677	ribosomal protein S8 [Klebsiella pneumoniae 342]	145.76	9.23%	1	4	130	14.1	9.42
80	38.4	197287082	30S ribosomal protein S8 [Proteus mirabilis HI4320]	145.76	9.23%	1	4	130	14.2	9.23
81	38.5	26249894	30S ribosomal protein S8 [Escherichia coli CFT073]	145.76	9.23%	1	4	130	14.1	9.42
82	39	15599581	chaperonin GroEL [Pseudomonas aeruginosa PAO1]	972.87	35.83%	11	21	547	57	5.14
83	40	15599888	ketol-acid reductoisomerase [Pseudomonas aeruginosa PAO1]	239	5.62%	1	4	338	36.4	5.85
84	41	15600562	phosphate ABC transporter periplasmic phosphate-binding prot	182.81	8.98%	1	3	323	34.5	9.09
85	42	15600747	F0F1 ATP synthase subunit beta [Pseudomonas aeruginosa PAO	501.51	17.69%	5	10	458	49.5	5.11
86	43	15600748	F0F1 ATP synthase subunit gamma [Pseudomonas aeruginosa P	47.49	6.64%	1	1	286	31.5	7.87
87	44.1	116516768	eno gene product [Streptococcus pneumoniae D39]	105.34	5.76%	2	3	434	47.1	4.81
88	44.2	225858936	phosphopyruvate hydratase [Streptococcus pneumoniae 70585	105.34	5.76%	2	3	434	47.1	4.81
89	44.3	149003747	phosphopyruvate hydratase [Streptococcus pneumoniae SP14-I	105.34	5.73%	2	3	436	47.3	4.83
90	45.1	116516621	rplL gene product [Streptococcus pneumoniae D39]	182.24	19.67%	3	5	122	12.4	4.49
91	45.2	303254509	50S ribosomal protein L7/L12 [Streptococcus pneumoniae BS45	182.24	19.67%	3	5	122	12.5	4.49
92	46.1	116516442	gap gene product [Streptococcus pneumoniae D39]	392.14	18.21%	4	9	335	35.8	5.47
93	46.2	225859769	glyceraldehyde-3-phosphate dehydrogenase, type I [Streptococ	392.14	18.21%	4	9	335	35.8	5.59
94	46.3	182684949	glyceraldehyde-3-phosphate dehydrogenase [Streptococcus pn	392.14	16.99%	4	9	359	38.7	6.34
95	46.4	169832817	glyceraldehyde-3-phosphate dehydrogenase, type I [Streptococ	392.14	18.21%	4	9	335	35.8	5.47
96	46.5	298501510	glyceraldehyde-3-phosphate dehydrogenase [Streptococcus pn	392.14	17.73%	4	9	344	37	5.96
97	46.6	15903867	glyceraldehyde-3-phosphate dehydrogenase [Streptococcus pn	392.14	16.99%	4	9	359	38.7	6.13
98	46.7	183603733	glyceraldehyde-3-phosphate dehydrogenase, type I [Streptococ	392.14	17.73%	4	9	344	37	5.96
99	47.1	116515886	unnamed protein product [Streptococcus pneumoniae D39]	100.04	1.47%	1	2	883	97.2	6.55
100	47.2	15903879	bifunctional acetaldehyde-CoA/alcohol dehydrogenase [Streptc	100.04	1.46%	1	2	890	98.1	6.48
101	48.1	116515871	rplR gene product [Streptococcus pneumoniae D39]	77.98	25.42%	1	1	118	12.9	10.4
102	48.2	182683204	50S ribosomal protein L18 [Streptococcus pneumoniae CGSP14]	77.98	22.90%	1	1	131	14.4	10.49
103	48.3	111658785	hypothetical protein SpneT_02000082 [Streptococcus pneumor	77.98	24.79%	1	1	121	13.2	10.43
104	49.1	116515356	tuf gene product [Streptococcus pneumoniae D39]	336	4.27%	2	5	398	43.9	4.97
105	49.2	182684444	elongation factor Tu [Streptococcus pneumoniae CGSP14]	336	4.21%	2	5	404	44.7	4.94
106	50	169834089	50S ribosomal protein L11 [Streptococcus pneumoniae Hungary	79.78	17.02%	1	1	141	14.8	9.7
107	51.1	26246055	pyruvate dehydrogenase subunit E1 [Escherichia coli CFT073]	942.61	21.20%	13	27	887	99.6	5.68
108	51.2	206576843	pyruvate dehydrogenase (acetyl-transferring), homodimeric typ	564.21	14.43%	8	16	887	99.5	5.64
109	52	161486328	dihydrolipoamide dehydrogenase [Escherichia coli CFT073]	426.07	14.14%	4	9	474	50.7	6.15
110	53.1	26246108	serine endoprotease [Escherichia coli CFT073]	122.8	4.22%	1	2	474	49.3	8.56
111	53.1	334123382	protease do [Enterobacter hormaechei ATCC 49162]	122.8	4.19%	1	2	477	49.4	8.56
112	54	26246124	periplasmic chaperone [Escherichia coli CFT073]	83.19	9.94%	1	1	161	17.7	9.7
113	55	26246476	acriflavin resistance protein A [Escherichia coli CFT073]	98.08	5.13%	1	2	409	43.5	8.43
114	56	26246561	outer membrane receptor FepA [Escherichia coli CFT073]	989.75	23.32%	10	22	746	82	5.45
115	57.1	161486279	succinate dehydrogenase flavoprotein subunit [Escherichia coli	149.4	2.38%	1	2	588	64.4	6.27
116	57.2	334122842	succinate dehydrogenase [Enterobacter hormaechei ATCC 4916	149.4	2.38%	1	2	588	64.4	6.02
117	57.3	206580158	succinate dehydrogenase, flavoprotein subunit [Klebsiella pneu	149.4	2.38%	1	2	588	64.5	6.23
118	57.4	197284464	succinate dehydrogenase flavoprotein subunit [Proteus mirabili	149.4	2.38%	1	2	588	64.7	6.02
119	57.5	15596780	succinate dehydrogenase flavoprotein subunit [Pseudomonas a	149.4	2.37%	1	2	590	63.5	6.49
120	58	26246711	peptidoglycan-associated outer membrane lipoprotein [Escheri	197.32	8.29%	1	4	181	19.7	6.8
121	59	26246790	outer membrane protein X [Escherichia coli CFT073]	118.93	8.67%	1	2	173	18.8	7.17
122	60.1	26246936	30S ribosomal protein S1 [Escherichia coli CFT073]	323.59	16.88%	5	7	557	61.1	4.98
123	60.2	334122618	30S ribosomal protein S1 [Enterobacter hormaechei ATCC 4916	323.59	16.88%	5	7	557	61.2	4.98
124	61	26246978	outer membrane protein A [Escherichia coli CFT073]	234.25	15.83%	5	10	379	41	6.71
125	62	26247566	global DNA-binding transcriptional dual regulator H-NS [Escheri	85.19	10.22%	1	1	137	15.5	5.47
126	63	26247570	bifunctional acetaldehyde-CoA/alcohol dehydrogenase [Escheri	1498.61	25.25%	17	36	891	96.1	6.79
127	64	26247735	hypothetical protein c1877 [Escherichia coli CFT073]	62.51	5.10%	1	1	353	38.7	9.31
128	65.1	26247927	major outer membrane lipoprotein [Escherichia coli CFT073]	197.29	15.38%	1	4	78	8.3	9.25
129	65.2	334122238	major outer membrane lipoprotein [Enterobacter hormaechei A	197.29	15.38%	1	4	78	8.4	9.31
130	66	26248038	glyceraldehyde-3-phosphate dehydrogenase [Escherichia coli CI	488.47	19.76%	5	13	334	35.9	6.8
131	67	26248190	flagellin [Escherichia coli CFT073]	577.89	9.24%	3	7	595	60.9	4.91
132	68	26248274	putative inner membrane ABC-transporter [Escherichia coli CFT	64.1	2.67%	1	1	600	66.2	9.03
133	69	26248288	putative pesticin receptor [Escherichia coli CFT073]	991.03	32.10%	11	23	673	73.6	5.73
134	70	26248619	DNA gyrase subunit A [Escherichia coli CFT073]	180.81	2.17%	1	2	875	96.9	5.2
135	71.1	26248830	malic enzyme [Escherichia coli CFT073]	98.86	2.64%	1	2	759	82.4	5.5
136	71.2	334125040	NADP-dependent malate dehydrogenase (oxaloacetate-decarbo	98.86	2.64%	1	2	759	82.3	5.35
137	73	26248869	inosine 5'-monophosphate dehydrogenase [Escherichia coli CFT	315.86	7.24%	2	5	511	54.6	6.42
138	74.1	161486147	autonomous glycyl radical cofactor GrcA [Escherichia coli CFT07	91.92	26.77%	2	2	127	14.3	5.17
139	74.2	206580560	autonomous glycyl radical cofactor [Klebsiella pneumoniae 342]	91.92	26.77%	2	2	127	14.2	5.02
140	75	26249182	phosphopyruvate hydratase [Escherichia coli CFT073]	550.99	23.84%	5	10	432	45.6	5.48
141	76	26249208	L-fucose isomerase [Escherichia coli CFT073]	122.44	6.26%	2	3	591	65	6.07
142	77	161486121	outer membrane channel protein [Escherichia coli CFT073]	102.75	5.48%	2	3	493	53.7	6.09
143	78	161486111	ATP-dependent RNA helicase DeaD [Escherichia coli CFT073]	162.95	6.84%	2	2	629	70.5	8.72
144	79	26249906	50S ribosomal protein L22 [Escherichia coli CFT073]	80.73	21.82%	1	1	110	12.2	10.23
145	80	26249916	30S ribosomal protein S10 [Escherichia coli CFT073]	92.47	14.56%	1	2	103	11.7	9.69

index	Prot_hit	Accession	Description	Mascot Score	Coverage	# Peptides	# PSMs	# AAs	MW [kDa]	calc. pI
146	81	26249938	30S ribosomal protein S7 [Escherichia coli CFT073]	283.4	11.54%	2	5	156	17.6	10.3
147	82	26250130	outer membrane heme/hemoglobin receptor [Escherichia coli C	559.72	17.28%	6	12	648	71.1	5.22
148	83.1	26250476	F0F1 ATP synthase subunit beta [Escherichia coli CFT073]	580.81	25.43%	6	11	460	50.3	5.01
149	83.2	334121923	ATP synthase F1 sector beta subunit [Enterobacter hormaechei	580.81	25.43%	6	11	460	50.2	5
150	84	26250714	peroxidase/catalase HPI [Escherichia coli CFT073]	118.35	2.17%	1	1	739	81.4	5.31
151	85.1	161486036	glycerol dehydrogenase [Escherichia coli CFT073]	210.67	4.63%	1	3	367	38.7	4.88
152	85.2	334121800	glycerol dehydrogenase [Enterobacter hormaechei ATCC 49162	210.67	4.63%	1	3	367	38.9	5.1
153	85.3	206579133	glycerol dehydrogenase [Klebsiella pneumoniae 342]	210.67	4.63%	1	3	367	38.6	5.1
154	84	26251030	aspartate ammonia-lyase [Escherichia coli CFT073]	58.34	4.67%	1	1	493	54	5.76
155	85	26251035	chaperonin GroEL [Escherichia coli CFT073]	1528.99	36.13%	12	31	548	57.3	4.94
156	86	P62805	Histone H4 n=219 Tax=Eukaryota RepID=H4_HUMAN	111.98	30.10%	2	3	103	11.4	11.36
157	87	P04264	Keratin, type II cytoskeletal 1 n=7 Tax=Eutheria RepID=K2C1_HU	428.67	9.94%	3	5	644	66	8.12
158	88	P13645	Keratin, type I cytoskeletal 10 n=12 Tax=Eutheria RepID=K1C10_	98.17	4.62%	1	2	584	58.8	5.21
159	89	P05109	Protein S100-A8 n=5 Tax=Hominoidea RepID=S10A8_HUMAN	134.52	24.73%	3	5	93	10.8	7.03
160	90	P06702	Protein S100-A9 n=5 Tax=Hominoidea RepID=S10A9_HUMAN	1398.2	78.07%	7	37	114	13.2	6.13
161	91.1	E9PFJ3	Uncharacterized protein n=3 Tax=Simiiformes RepID=E9PFJ3_H	3325.36	14.88%	10	78	578	63.3	5.31
162	91.2	P07911	Uromodulin n=2 Tax=Catarrhini RepID=UROM_HUMAN	3325.36	13.44%	10	78	640	69.7	5.24
163	92.1	Q9UP81	Mutant beta-globin n=1 Tax=Homo sapiens RepID=Q9UP81_HU	181.63	35.96%	2	3	89	9.7	8.19
164	92.2	B3VL86	Mutant beta-globin n=4 Tax=Homo sapiens RepID=B3VL86_HUI	181.63	23.02%	2	3	139	14.9	6.77
165	92.3	P68871	Hemoglobin subunit beta n=102 Tax=Primates RepID=HBB_HUN	181.63	21.77%	2	3	147	16	7.28
166	92.4	Q14473	Beta-globin gene from a thalassemia patient, n=1 Tax=Homo sa	181.63	18.29%	2	3	175	18.9	6.79
167	93.1	B4DW52	Uncharacterized protein n=12 Tax=Coelomata RepID=B4DW52_	257.3	25.65%	5	8	347	38.6	5.35
168	93.2	P63261	Actin, cytoplasmic 2 n=1334 RepID=ACTG_HUMAN	257.3	23.73%	5	8	375	41.8	5.48
169	94.1	P06899	Histone H2B type 1-J n=102 Tax=Euteleostomi RepID=H2B1J_HU	179.87	11.90%	1	2	126	13.9	10.32
170	94.2	P62807	Histone H2B type 1-C/E/F/G/I n=44 Tax=Euteleostomi RepID=H2	179.87	11.90%	1	2	126	13.9	10.32
171	94.3	B4DR52	Histone H2B n=5 Tax=Euarchontoglires RepID=B4DR52_HUMAN	179.87	9.04%	1	2	166	18	10.32
172	94.4	P58876	Histone H2B type 1-D n=136 Tax=Coelomata RepID=H2B1D_HU	179.87	11.90%	1	2	126	13.9	10.32
173	95.1	P01834	Ig kappa chain C region n=2 Tax=Homo sapiens RepID=IGKC_HU	121.21	35.85%	2	2	106	11.6	5.87
174	95.2	UPI000011049E	IGG CTM01 FAB (LIGHT CHAIN) n=2 Tax=Homo sapiens RepID=U	121.21	17.35%	2	2	219	24.1	7.74
175	95.3	Q0KKI6	Immunoblobulin light chain (Fragment) n=3 Tax=Homo sapiens	121.21	17.35%	2	2	219	24	8.06
176	95.4	Q5EFE6	Anti-RhD monoclonal T125 kappa light chain n=1 Tax=Homo sap	121.21	16.24%	2	2	234	25.7	8.43
177	95.5	Q6GMX0	Putative uncharacterized protein n=14 Tax=Euarchontoglires Re	121.21	16.10%	2	2	236	25.8	7.97
178	95.6	Q6P5S8	IGK@ protein n=6 Tax=Homo sapiens RepID=Q6P5S8_HUMAN	121.21	16.10%	2	2	236	25.8	6.33
179	95.7	Q7Z3Y4	Putative uncharacterized protein n=3 Tax=Homininae RepID=Q7	121.21	16.10%	2	2	236	25.7	7.59
180	95.8	UPI0000110495	ANTIBODY A5B7 (LIGHT CHAIN) n=1 Tax=Homo sapiens RepID=U	121.21	17.84%	2	2	213	23.1	8.06
181	95.9	UPI0000110769	ANTIBODY n=1 Tax=Homo sapiens RepID=UPI0000110769	121.21	17.76%	2	2	214	23.6	6.33
182	95.10	UPI000014CDC4	OMALIZUMAB LIGHT CHAIN n=2 Tax=Homo sapiens RepID=UPI	121.21	17.43%	2	2	218	23.9	5.39
183	95.11	UPI00003CEBF2	HU3S193 Fab fragment, light chain n=3 Tax=Euarchontoglires Re	121.21	17.35%	2	2	219	24	8.06
184	95.12	UPI0001A7C522	HUMAN IGG ABT-325 n=1 Tax=Homo sapiens RepID=UPI0001A7	121.21	17.76%	2	2	214	23.5	6.55
185	95.13	UPI0001A7C54C	Fab Z13e1 n=3 Tax=Euarchontoglires RepID=UPI0001A7C54C	121.21	17.92%	2	2	212	23.1	7.71
186	95.14	UPI0001C591D8	Humanized recombinant Fab fragment of a murin n=5 Tax=Euar	121.21	16.59%	2	2	229	25.1	5.62
187	95.15	UPI0001D3410B	Immunoglobulin light chain n=1 Tax=Homo sapiens RepID=UPI0	121.21	17.43%	2	2	218	23.3	8.05
188	95.16	UPI0001F2FE82	Fab 2G12, light chain n=2 Tax=Homo sapiens RepID=UPI0001F2	121.21	17.84%	2	2	213	23.2	8.13
189	95.17	UPI0002003CF0	IMMUNOGLOBULIN A1 LIGHT CHAIN n=1 Tax=Homo sapiens Re	121.21	17.35%	2	2	219	24	6.77
190	95.18	UPI000211B3C2	B3 (IGKV4-1) light chain n=1 Tax=Homo sapiens RepID=UPI0002	121.21	17.27%	2	2	220	24.2	6.55
191	95.19	UPI0002177BCC	S4 FAB LIGHT CHAIN n=1 Tax=Homo sapiens RepID=UPI000217	121.21	18.01%	2	2	211	22.7	7.77
192	96	P01833	Polymeric immunoglobulin receptor n=5 Tax=Hominoidea RepII	91.81	2.62%	1	1	764	83.2	5.74
193	97	Q6MZU6	Putative uncharacterized protein DKFZp686C15213 n=1 Tax=Ho	147.18	8.41%	2	3	464	51.1	7.71
194	98.1	P01876	Ig alpha-1 chain C region n=2 Tax=Homo sapiens RepID=IGHA1_	556.05	14.73%	4	12	353	37.6	6.51
195	98.2	Q96DK0	CDNA FLJ25298 fis, clone STM07683, highly similar to Protein Tr	556.05	10.48%	4	12	496	53.5	6.65
196	98.3	Q6N090	Putative uncharacterized protein DKFZp686G21220 (Fragment)	556.05	10.28%	4	12	506	54.4	6.6
197	98.4	Q6ZW64	cDNA FLJ41552 fis, clone COLON2004478, highly similar to Prote	556.05	10.53%	4	12	494	53.3	6.52
198	98.5	Q8WY24	SNC66 protein n=1 Tax=Homo sapiens RepID=Q8WY24_HUMAN	556.05	10.46%	4	12	497	53.6	6.67
199	99.1	A1L195	TUBB2B protein (Fragment) n=16 Tax=Fungi/Metazoa group Re	109.49	23.94%	2	2	188	20.6	5.05
200	99.2	Q13885	Tubulin beta-2A chain n=339 RepID=TBB2A_HUMAN	109.49	10.11%	2	2	445	49.9	4.89
201	99.3	Q5ST81	Tubulin beta polypeptide n=8 Tax=Eukaryota RepID=Q5ST81_HU	109.49	12.10%	2	2	372	41.7	4.91
202	99.4	E9PBJ4	Uncharacterized protein n=2 Tax=Homo sapiens RepID=E9PBJ4_	109.49	18.52%	2	2	243	27.4	4.49
203	100.1	P01857	Ig gamma-1 chain C region n=3 Tax=Homo sapiens RepID=IGHG	372.33	19.70%	4	14	330	36.1	8.19
204	100.2	A8K008	cDNA FLJ78387 n=2 Tax=Homo sapiens RepID=A8K008_HUMAN	372.33	13.77%	4	14	472	51.6	8.16
205	100.3	Q6GMX6	IGH@ protein n=2 Tax=Homo sapiens RepID=Q6GMX6_HUMAN	372.33	13.98%	4	14	465	51.1	8.69
206	100.4	Q6N094	Putative uncharacterized protein DKFZp686O01196 n=3 Tax=Ho	372.33	13.54%	4	14	480	52.6	8.18
207	100.5	Q6N097	Putative uncharacterized protein DKFZp686H20196 n=1 Tax=Ho	372.33	13.51%	4	14	481	52.7	8.48
208	101	P11678	Eosinophil peroxidase n=6 Tax=Simiiformes RepID=PERE_HUMA	104.06	3.50%	3	4	715	81	10.29
209	102	P05164	Myeloperoxidase n=6 Tax=Catarrhini RepID=PERM_HUMAN	190.57	12.35%	6	6	745	83.8	8.97
210	103.1	D6RHJ6	Uncharacterized protein n=1 Tax=Homo sapiens RepID=D6RHJ6	85.85	7.64%	1	1	157	17.7	6.32
211	103.2	P01591	Immunoglobulin J chain n=12 Tax=Simiiformes RepID=IGJ_HUM	85.85	7.55%	1	1	159	18.1	5.24
212	104	P01871	Ig mu chain C region n=4 Tax=Homo sapiens RepID=IGHM_HUN	80.73	3.54%	1	1	452	49.3	6.77
213	105.1	P0CG05	Ig lambda-2 chain C regions n=4 Tax=Homo sapiens RepID=LAC2	474.26	34.91%	3	11	106	11.3	7.24
214	105.2	A0A5E4	Putative uncharacterized protein n=1 Tax=Homo sapiens RepID=	474.26	15.74%	3	11	235	24.7	5.94
215	105.3	A2NUT2	Lambda-chain (AA -20 to 215) n=5 Tax=Euarchontoglires RepID=	474.26	15.74%	3	11	235	24.6	7.62
216	105.4	C6KXN3	Lambda light chain of human immunoglobulin surface antigen-r	474.26	15.95%	3	11	232	24.7	5.54
217	105.5	Q567P1	IGL@ protein n=3 Tax=Homo sapiens RepID=Q567P1_HUMAN	474.26	15.74%	3	11	235	24.8	7.3
218	105.6	Q6GMW3	IGL@ protein n=7 Tax=Homininae RepID=Q6GMW3_HUMAN	474.26	15.81%	3	11	234	24.6	6.37

index	Prot_hit	Accession	Description	Mascot Score	Coverage	# Peptides	# PSMs	# AAs	MW [kDa]	calc. pI
219	105.7	Q6GMX3	IGL@ protein n=3 Tax=Homo sapiens RepID=Q6GMX3_HUMAN	474.26	15.68%	3	11	236	24.7	6.89
220	105.8	Q6IPQ0	IGL@ protein n=6 Tax=Homo sapiens RepID=Q6IPQ0_HUMAN	474.26	15.68%	3	11	236	24.8	6.76
221	105.9	Q7Z2U7	Putative uncharacterized protein n=1 Tax=Homo sapiens RepID=	474.26	15.81%	3	11	234	25	6.38
222	105.10	Q8N5F4	IGL@ protein n=4 Tax=Hominoidea RepID=Q8N5F4_HUMAN	474.26	15.88%	3	11	233	24.9	5.34
223	105.11	UPI000158A17D	Bence Jones KWR Protein - Immunoglobulin Ligh n=1 Tax=Homo	474.26	17.05%	3	11	217	22.7	6.16
224	105.12	UPI0001BEF2DB	Fab 537-10D, light chain n=1 Tax=Homo sapiens RepID=UPI0001	474.26	17.05%	3	11	217	22.9	7.25
225	105.13	UPI0001D63C09	Antibody PG16 Light Chain n=3 Tax=Homo sapiens RepID=UPI00	474.26	17.13%	3	11	216	22.7	6.99
226	105.14	UPI0001D63C0B	Antibody PG9 light chain n=1 Tax=Homo sapiens RepID=UPI000	474.26	17.13%	3	11	216	22.8	8.5
227	106.1	Q9UL88	Myosin-reactive immunoglobulin heavy chain variable region (F	154.17	14.50%	1	2	131	14.1	9.63
228	106.2	UPI0001BEF2D9	Fab 447-52D, heavy chain n=1 Tax=Homo sapiens RepID=UPI00	154.17	8.09%	1	2	235	25.2	8.5
229	107	P01619	Ig kappa chain V-III region B6 n=1 Tax=Homo sapiens RepID=KV	72.47	16.67%	1	1	108	11.6	9.25
230	108.1	B1N7B8	Cryocrystalglobulin CC1 kappa light chain variable region (Fragm	88.02	16.82%	1	1	107	11.8	7.99
231	108.2	A0N5G5	Rheumatoid factor D5 light chain (Fragment) n=1 Tax=Homo sa	88.02	15.25%	1	1	118	12.8	8.97
232	109	P02788	Lactotransferrin n=24 Tax=Hominoidea RepID=TRFL_HUMAN	190.97	6.06%	2	3	710	78.1	8.12
233	110.1	B1AM77	Stomatin n=5 Tax=Mammalia RepID=B1AM77_HUMAN	68.29	16.26%	1	1	123	13.5	8.02
234	110.2	E7EQ93	Uncharacterized protein n=3 Tax=Simiiformes RepID=E7EQ93_H	68.29	10.70%	1	1	187	20.8	8.87
235	110.3	P27105	Erythrocyte band 7 integral membrane protein n=13 Tax=Euthe	68.29	6.94%	1	1	288	31.7	7.88

2DLCSCI_51 Peptide Summary

Sequence	Protein Descriptions	# Proteins	# Protein Groups	Protein Group Accessions	Modifications	q-Value	PEP	Charge	m/z [Da]	MH+ [Da]	Delta Mass [Da]
FVGQGGAR	Uncharacterized protein n=3 Tax=Simiiforme	2	1	E9PFJ3		0.0039	0.174	2	396.44769	791.88811	0.24
VGLFGGAGVGK	F0F1 ATP synthase subunit beta [Escherichia	3	2	26250476;15600747		0	0.000799	2	481.72162	962.43596	0.45
GFLIGGTSGR	pyruvate dehydrogenase subunit E1 [Escher	2	1	26246055		0	0.0241	2	483.16995	965.33263	0.41
VGYVSGWGR	HP protein n=9 Tax=Simiiformes RepID=Q6N	2	1	Q6NSB4		0	0.00326	2	491.37305	981.73882	0.62
GVNVLADAVK	chaperone GroEL [Enterobacter hormaechei	3	3	334123809;197286390;26251035		0	0.00119	2	493.92648	986.84569	0.64
KDLQNFLK	Protein S100-A9 n=5 Tax=Hominoidea RepID	1	1	P06702		0.00105	0.0432	2	503.75531	1006.50334	0.47
VGGTGMFTVR	Uncharacterized protein n=3 Tax=Simiiforme	2	1	E9PFJ3		0	0.000553	2	513.16632	1025.32536	0.4
SDVLFTFNK	outer membrane protein A [Escherichia coli	1	1	26246978		0.00105	0.048	1	1070.56335	1070.56335	0.01
GFSPKDVLVR	Ig alpha-1 chain C region n=2 Tax=Homo sap	5	1	P01876		0	0.000857	2	559.5741	1118.14092	0.25
DWVSVVTPAR	Uncharacterized protein n=3 Tax=Simiiforme	2	1	E9PFJ3		0	0.00225	1	1129.64185	1129.64185	0.04
VFmYLSDSR	Uncharacterized protein n=3 Tax=Simiiforme	2	1	E9PFJ3	M3(Oxidation)	0	0.0234	2	567.84558	1134.68389	0.58
NAIIFSPHPR	bifunctional acetaldehyde-CoA/alcohol dehy	1	1	26247570		0	0.031	2	576.58423	1152.16118	0.26
AGIALNDNFVK	glyceraldehyde-3-phosphate dehydrogenase	1	1	26248038		0	0.00246	1	1161.62341	1161.62341	0
GPSVFPLAPSSK	Ig gamma-1 chain C region n=3 Tax=Homo sa	5	1	P01857		0	0.015	1	1186.6012	1186.6012	-0.05
AVFPSIVGRPR	Uncharacterized protein n=12 Tax=Coeloma	2	1	B4DW52		0	0.00167	2	600.38806	1199.76885	0.53
WLQGSQELPR	Ig alpha-1 chain C region n=2 Tax=Homo sap	5	1	P01876		0	0.000467	2	607.7467	1214.48613	0.43
ALNSIIDVYHK	Protein S100-A8 n=5 Tax=Hominoidea RepID	1	1	P05109		0	0.000636	2	637.30371	1273.60015	0.45
FNIDADKVNPR	pyruvate dehydrogenase subunit E1 [Escher	1	1	26246055		0.0039	0.133	2	645.17358	1289.33989	0.34
FATHGGYLLQGK	bifunctional acetaldehyde-CoA/alcohol dehy	1	1	26247570		0	0.000108	2	646.82385	1292.64043	0.48
VNVDEVGGEALGR	Mutant beta-globin n=1 Tax=Homo sapiens	4	1	Q9UP81		0	0.000163	2	657.95886	1314.91045	0.12
IGLDLAALNMQR	Eosinophil peroxidase n=6 Tax=Simiiformes	1	1	P11678		0	0.00564	2	658.34155	1315.67583	0.48
AMIALDGTPNKGK	eno gene product [Streptococcus pneumoni	3	1	116516768		0	0.0403	2	658.59509	1316.18291	0.24
IVGLQTEAPLKR	formate acetyltransferase [Enterobacter hor	2	2	334122625;206577432		0	0.00116	2	663.06421	1325.12114	0.16
GKVQLLGSGSILR	pyruvate dehydrogenase subunit E1 [Escher	2	1	26246055		0	0.000301	2	664.85461	1328.70195	0.45
MLVGVNVLADAVK	chaperonin GroEL [Pseudomonas aeruginosa	1	1	15599581		0	1.02E-06	2	665.11377	1329.22026	0.23
SLTEIKDVLASR	DNA-directed RNA polymerase subunit alpha	3	1	197287093		0	0.0192	2	666.72961	1332.45195	0.35
LVADSITSQLER	ribosomal protein S3 [Klebsiella pneumoniae	3	1	206580744		0	2.74E-06	2	666.80713	1332.60698	0.45
GIYKLETIEGSK	pyruvate dehydrogenase subunit E1 [Escher	2	1	26246055		0	0.000967	2	669.58264	1338.15801	0.21
ALNIENIIAEIK	rplL gene product [Streptococcus pneumoni	2	1	116516621		0	0.00103	2	671.12689	1341.24651	0.23
IGLDLPALNMQR	Myeloperoxidase n=6 Tax=Catarrhini RepID=	1	1	P05164		0	0.0144	2	671.4032	1341.79912	0.53
EASILELNDLVK	rplL gene product [Streptococcus pneumoni	2	1	116516621		0	0.00169	2	672.7843	1344.56133	0.41
YPVVPVHLDTTI	Eosinophil cationic protein n=3 Tax=Hominir	1	1	P12724		0	0.00106	2	677.81927	1354.63127	0.45
VDQLSNDVNAMR	major outer membrane lipoprotein [Escheric	2	1	26247927		0	7.22E-06	2	681.48462	1361.96196	0.16
TMTDPIADMLTR	30S ribosomal protein S8 [Staphylococcus ep	2	1	242371883		0	0.000555	2	683.37714	1365.747	0.55
MKLNEVKEFVK	rpmC gene product [Streptococcus pneumor	1	1	116516938		0.0039	0.119	3	455.96713	1365.88684	0.38
LSNQISGLNVATR	flagellin type B [Pseudomonas aeruginosa PA	1	1	15596289		0	6.79E-06	1	1372.77991	1372.77991	0.03
VINQLTGGLAGMAK	dihydrolipoamide dehydrogenase [Escherich	1	1	161486328		0	1.23E-06	2	687.28271	1373.55815	0.4
AVAAGMNPMDLKR	chaperone GroEL [Enterobacter hormaechei	4	4	334123809;197286390;15599581;26251035		0	5.02E-05	2	687.57355	1374.13982	0.22
WNLDKIAEGLSK	ABC superfamily ATP binding cassette transp	7	1	227517533		0	0.000221	2	687.88086	1374.75444	0.51
TNYLGLLAINQR	Eosinophil peroxidase n=6 Tax=Simiiformes	1	1	P11678		0	0.0139	2	688.45831	1375.90935	0.07
SMQDPLADMLTR	30S ribosomal protein S8 [Pseudomonas aer	5	1	15599445		0	3.64E-05	2	689.88477	1378.76225	0.56
RAQSVVDYLISK	outer membrane protein A [Escherichia coli	1	1	26246978		0	0.00198	2	690.40289	1379.79851	0.52
VMTDPIADFLTR	rpsH gene product [Streptococcus pneumon	5	1	116515705		0	0.000224	2	690.47406	1379.94084	0.62
SSEDPNEDIVER	Uncharacterized protein n=1 Tax=Homo sap	2	1	D6RHJ6		0	6.90E-05	2	695.70758	1390.40788	0.4
QYDINEAIALLK	50S ribosomal protein L1 [Escherichia coli CF	2	1	26250754		0.00105	0.0549	2	696.36438	1391.72148	0.48
SmQDPIADMLTR	30S ribosomal protein S8 [Pseudomonas aer	5	1	15599445	M2(Oxidation)	0.0039	0.0943	2	697.78876	1394.57024	0.46
YNYLINQLNIK	outer membrane channel protein [Escherich	1	1	161486121		0	0.00162	2	698.60675	1396.20622	0.22
YGAGSSVNGVIDTR	putative pesticin receptor [Escherichia coli C	1	1	26248288		0	4.28E-05	2	698.79431	1396.58135	0.45
STDYGIFQINSR	UPI0000052769 related cluster n=1 Tax=Hor	2	1	UPI0000052769		0	0.00143	2	701.46362	1401.91997	0.62
DVLVNEYGVEGGR	porin [Pseudomonas aeruginosa PAO1]	1	1	15596974		0	7.37E-05	1	1406.76477	1406.76477	0.07
AGDEGKLFGSIGTR	50S ribosomal protein L9 [Proteus mirabilis	4	1	197287189		0	1.91E-05	2	704.82825	1408.64922	0.46
AFEANPSEELFR	rpsT gene product [Streptococcus pneumon	6	1	116515680		0	0.000319	2	705.75507	1410.50286	0.42
TALQPMVSALNIR	Uncharacterized protein n=3 Tax=Simiiforme	2	1	E9PFJ3		0	5.90E-05	2	707.60657	1414.20586	0.21
EGRPSEGETLIAR	polyribonucleotide nucleotidyltransferase [K	3	1	206579040		0	0.00514	2	708.36572	1415.72417	0.5
AVLEVAGVHNVLAK	30S ribosomal protein S5 [Enterobacter horr	2	1	334125906		0	5.07E-06	2	710.6604	1420.31352	0.24
IEDQLGEVAEYR	eno gene product [Streptococcus pneumoni	3	1	116516768		0	4.77E-05	2	711.81555	1422.62383	0.47
TALQPmVSALNIR	Uncharacterized protein n=3 Tax=Simiiforme	2	1	E9PFJ3	M6(Oxidation)	0	0.000685	2	715.78027	1430.55327	0.39
YGNGAAGGVVNIITK	ferrienterobactin receptor [Enterobacter ho	2	2	334122958;26246561		0	7.41E-07	2	717.41028	1433.81328	0.02
KSGVLTGLPDAYGR	formate acetyltransferase [Enterobacter hor	2	2	334122625;206577432		0	2.83E-05	2	717.85693	1434.70659	0.47
VEGGQHLNVNVLR	autonomous glycyl radical cofactor GrcA [Es	2	1	161486147		0	0.00194	2	718.01959	1435.03191	0.13
MAVAESGMGIVEDK	bifunctional acetaldehyde-CoA/alcohol dehy	1	1	26247570		0	1.59E-08	2	719.2804	1437.55351	0.44
HYNPDEIVLGKR	xylose isomerase [Klebsiella pneumoniae 34	4	1	206575717		0	0.00629	3	481.35709	1442.05671	0.43
GIDKAVTAAVEELK	chaperonin GroEL [Escherichia coli CFT073]	1	1	26251035		0	1.83E-05	2	722.78607	1444.56487	0.38
FPTDQLTPDQER	Myeloperoxidase n=6 Tax=Catarrhini RepID=	1	1	P05164		0	0.00054	2	723.99231	1446.97734	0.15
YTLAGTEVSALLGR	F0F1 ATP synthase subunit beta [Escherichia	3	2	26250476;15600747		0	8.14E-08	2	726.3877	1451.76811	0.49
AADMTGADIEAMTR	ribosomal protein L11 [Klebsiella pneumonia	2	1	206577888		0	8.64E-06	2	727.25171	1453.49614	0.43
LGHPDTLNQGEFK	Protein S100-A9 n=5 Tax=Hominoidea RepID	1	1	P06702		0	3.91E-05	2	728.8335	1456.65972	0.47
AQIIQQAGNSVLSK	flagellin [Enterobacter hormaechei ATCC 49	2	1	334124672		0	2.70E-06	2	729.33319	1457.65911	0.42
LGGNSLLDLVVFGR	succinate dehydrogenase flavoprotein subur	5	1	161486279		0	2.68E-07	2	730.94745	1460.88762	0.53
RIGLDLAALNMQR	Eosinophil peroxidase n=6 Tax=Simiiformes	1	1	P11678		0	0.0352	2	736.01086	1471.01445	0.1
ATQDAYVGWNDIK	putative pesticin receptor [Escherichia coli C	1	1	26248288		0	0.00973	2	741.31635	1481.62541	0.46
AKDFEDAVEKAEK	bifunctional acetaldehyde-CoA/alcohol dehy	1	1	26247570		0	0.00106	2	740.80798	1480.60869	0.44
TVTAmDVVYALKR	Histone H4 n=219 Tax=Eukaryota RepID=H4_	1	1	P62805	M5(Oxidation)	0	0.000445	2	742.36475	1483.72222	0.46
AKLESLVEDLVNR	chaperone DnaK [Enterobacter hormaechei	3	1	334123523		0	0.00159	2	743.84735	1486.68743	0.43
NGALNAAIVGQPAYK	bifunctional acetaldehyde-CoA/alcohol dehy	1	1	26247570		0	4.32E-06	1	1486.72473	1486.72473	-0.08
VPTPNVSVVDLTVR	glyceraldehyde-3-phosphate dehydrogenase	1	1	26248038		0	5.34E-07	2	748.94598	1496.88469	0.52
AQYLIDQLLAEAR	pyruvate dehydrogenase subunit E1 [Escher	1	1	26246055		0	8.73E-05	2	752.76617	1504.52507	0.35
MQLNSTEISELIK	ATP synthase F1, alpha subunit [Klebsiella p	2	1	206580919		0	4.40E-07	2	753.70105	1506.39482	0.3
QFPFLASIQNQGR	AZU1 protein (Fragment) n=1 Tax=Homo sap	2	1	Q86SR2		0	0.000114	2	753.90967	1506.81206	0.51
LGIQAFEPVLIEGK	DNA-directed RNA polymerase, beta' subuni	2	1	206578905		0.00207	0.0672	2	757.71295	1514.41863	0.28
DNRDWVSVVTPAR	Uncharacterized protein n=3 Tax=Simiiforme	2	1	E9PFJ3		0	0.0284	3	506.03394	1516.08725	0.44
DASGVTFTWTPSSGK	Ig alpha-1 chain C region n=2 Tax=Homo sap	5	1	P01876		0	8.47E-06	2	770.9342	1540.86113	0.07
KLNQEQVDYIVAK	unnamed protein product [Streptococcus pr	2	1	116515886		0	1.24E-05	2	774.87512	1548.74297	0.45
TNSTWPTTWFAPR	L-fucose isomerase [Escherichia coli CFT073]	1	1	26249208		0	0.00837	2	783.02551	1565.04375	0.14
LGAPDTPLEAVIAAAR	putative inner membrane ABC-transporter [	1	1	26248274		0	8.17E-07	2	783.35858	1565.70989	0.42
REEESAAAAEVEER	global DNA-binding transcriptional dual regu	1	1	26247566		0	1.81E-06	2	788.80469	1576.6021	0.44
AAVEEGVVPGGGVALVR	chaperonin GroEL [Pseudomonas aeruginosa	1	1	15599581		0	3.49E-06	2	790.75995	1580.51262	0.32
QKLESQISTLYGGR	cell division protein [Proteus mirabilis HI432	3	1	197287224		0	2.27E-06	2	790.86029	1580.7133	0.43
IKDTDFAAETAALSK	flagellin type B [Pseudomonas aeruginosa PA	1	1	15596289		0	5.19E-06	2	791.06433	1581.12139	0.15
GLNVDSTGAAISVPVGK	F0F1 ATP synthase subunit beta [Pseudomor	1	1	15600747		0	6.66E-07	2	793.27826	1585.54924	0.35
SADLISIGPMLQGMR	phosphate acetyltransferase [Enterobacter h	3	1	334124951		0	6.33E-06	2	795.41333	1589.81938	0.5
MHELAEKNYDVIK	threonyl-tRNA synthetase [Klebsiella pneum	2	1	206580341		0	9.67E-05	2	795.61786	1590.22844	0.21
NVVLDKSFGAPTITK	chaperone GroEL [Enterobacter hormaechei	3	3	334123809;15599581;26251035		0	3.09E-06	2	795.82281	1590.63835	0.37
IATDPFVGNLTFFR	elongation factor G (EF-G) [Proteus mirabilis	4	1	197286622		0	0.000166	2	799.85352	1598.69975	0.43
SLGQFNLDGINPAPR	chaperone DnaK [Enterobacter hormaechei	3	1	334123523		0	5.92E-05	2	800.39209	1599.7769	0.47
EAGVQEADFLANVDK	bifunctional acetaldehyde-CoA/alcohol dehy	1	1	26247570		0	0.00757	2	803.3988	1605.79033	0.01
FNQIGSLTETLAAIK	phosphopyruvate hydratase [Escherichia col	1	1	26249182		0	2.44E-09	2	803.64899	1606.2907	0.2
SFGAPTITKDGVSVAR	chaperone GroEL [Enterobacter hormaechei	2	2	334123809;26251035		0	8.42E-07	2	803.52979	1606.05229	0.1
ALTVNTNIASLNTQR	flagellin type B [Pseudomonas aeruginosa PA	1	1	15596289		0	1.28E-07	2	808.46796	1615.92864	0.03
QVGSGVTTDQVQAEAK	Ig mu chain C region n=4 Tax=Homo sapiens	1	1	P01871		0	6.91E-06	2	809.72607	1618.44487	0.32
TMPGVNLTGNSTSGQR	ferrienterobactin receptor [Enterobacter ho	2	2	334122958;26246561		0	7.83E-05	2	810.82471	1620.64214	0.43
SVDVGTWIAGVGYRF	outer membrane protein X [Escherichia coli	1	1	26246790		0	2.23E-06	2	814.49255	1627.97783	0.58
AQYLIDQLLAEARK	pyruvate dehydrogenase subunit E1 [Escher	1	1	26246055		0	5.59E-05	2	816.74878	1632.49028	0.29
NVSLTGGVDNLFDKR	outer membrane receptor FepA [Escherichia	1	1	26246561		0	1.67E-06	2	818.30762	1635.60796	0.38
ELAVQVAEAMTDFSK	ATP-dependent RNA helicase DeaD [Escheri	1	1	161486111		0	3.07E-07	2	820.20728	1639.40727	0.3
SLNNQFASFIDKVR	Keratin, type II cytoskeletal 1 n=7 Tax=Euthe	1	1	P04264		0	0.000101	2	820.3985	1639.78972	0.47
LDLAGRDLTDYLmK	Uncharacterized protein n=12 Tax=Coeloma	2	1	B4DW52	M13(Oxidation)	0	0.019	2	820.83862	1640.66997	0.42
FINELLPVIDSLDR	heat shock protein GrpE [Escherichia coli CF	1	1	26248977		0	0.0201	2	822.98883	1644.97038	0.54
VGSFEQALISYFQR	F0F1 ATP synthase subunit alpha [Pseudomo	1	1	15600749		0	0.000939	2	823.22406	1645.44084	0.3
IPEGLAGGTEGKFNEK	outer membrane receptor FepA [Escherichia	1	1	26246561		0	1.43E-08	2	824.33813	1647.66899	0.42
TLTATLPAYLNALTGK	preprotein translocase, SecA subunit [Klebsi	3	1	206580132		0	0.000651	2	824.93994	1648.87261	0.47

Sequence	Protein Descriptions	# Proteins	# Protein Groups	Protein Group Accessions	Modifications	q-Value	PEP	Charge	m/z [Da]	MH+ [Da]	Delta Mass [Da]
LGYPITDDLDIYTR	outer membrane protein A [Escherichia coli	1	1	26246978		0	1.95E-05	2	828.55542	1656.10356	0.64
LIDQATAEIVETAKR	30S ribosomal protein S10 [Escherichia coli C	1	1	26249916		0	3.34E-05	2	829.75482	1658.50237	0.3
LHILGVMEQAINAPR	polyribonucleotide nucleotidyltransferase [K	3	1	206579040		0	1.32E-08	2	831.86169	1662.71611	0.4
QLDEGLAGDNVGVLLR	tuf gene product [Streptococcus pneumonia	2	1	116515356		0	3.05E-08	2	835.37738	1669.74748	0.43
IQDADYATEVSNMSK	flagellin [Enterobacter hormaechei ATCC 49	3	2	334124672;26248190		0	3.30E-08	2	836.75781	1672.50835	0.38
INSAKDDAAGLQISNR	flagellin type B [Pseudomonas aeruginosa P	1	1	15596289		0	2.65E-08	2	837.34271	1673.67815	0.41
ADSSPVKAGVETTTPSK	Ig lambda-2 chain C regions n=4 Tax=Homo s	14	1	P0CG05		0	2.96E-07	2	838.21405	1675.42082	0.28
LVSWYDNETGYSNK	glyceraldehyde-3-phosphate dehydrogenase	1	1	26248038		0	6.65E-05	2	838.37817	1675.74907	-0.01
YTTDLFSLDVAYNR	outer membrane heme/hemoglobin recepto	1	1	26250130		0	0.000891	2	839.77991	1678.55254	0.37
FNWYVDGVEVHNAK	Ig gamma-1 chain C region n=3 Tax=Homo sa	5	1	P01857		0	0.00592	2	839.81018	1678.61308	0.41
AAAFEGELIPASQIDR	ribosomal protein L10 [Klebsiella pneumonia	3	1	206577086		0	3.66E-05	2	844.81689	1688.62651	0.38
INDLTDPVMLAHLLK	gap gene product [Streptococcus pneumonia	7	1	116516442		0	5.56E-05	2	847.47571	1693.94414	0.5
DSTIQVVENGESSQGR	Uncharacterized protein n=3 Tax=Simiiforme	2	1	E9PFJ3		0	1.13E-07	2	853.80328	1706.59929	0.4
KQIEEATSDYDREK	chaperone GroEL [Enterobacter hormaechei	1	1	334123809		0	8.70E-05	2	856.80237	1712.59746	0.39
QQIEEATSDYDREK	chaperonin GroEL [Escherichia coli CFT073]	1	1	26251035		0	3.33E-05	2	856.80237	1712.59746	0.41
GISYETATFPWAASGR	dihydrolipoyl dehydrogenase [Enterobacter	2	2	334123429;161486328		0	0.000796	2	857.49133	1713.97539	0.08
GTWQIDSAQSLSGLVR	outer membrane heme/hemoglobin recepto	1	1	26250130		0	0.000546	2	859.82202	1718.63677	0.38
DRGEDALIIYDDLSK	ATP synthase F1, alpha subunit [Klebsiella pi	2	1	206580919		0	0.000383	2	861.87653	1722.74578	-0.05
EGVQEDILEILLNLK	DNA-directed RNA polymerase subunit alpha	3	1	197287093		0	2.62E-06	2	864.02649	1727.0457	0.54
MNIGQILETHLGMAAK	DNA-directed RNA polymerase, beta subunit	3	1	206579488		0	5.34E-06	2	864.44104	1727.8748	0.49
FAPGWSWDINGNVIR	putative pesticin receptor [Escherichia coli C	1	1	26248288		0	1.06E-08	2	866.78235	1732.55742	0.35
SDFAQMLDAHANFLR	peptidoglycan-associated outer membrane l	1	1	26246711		0	5.34E-06	2	868.73694	1736.4666	0.32
VPTPTGSVTELVAVLEK	gap gene product [Streptococcus pneumonia	7	1	116516442		0	1.60E-08	2	870.93994	1740.87261	0.45
TIVDVLDATTTLYNAK	outer membrane protein TolC [Klebsiella pn	2	1	161486121		0	3.36E-05	2	869.95862	1738.90996	0.49
VIEHIMEDLDTNADK	Protein S100-A9 n=5 Tax=Hominoidea RepID	1	1	P06702		0	3.36E-05	2	872.31628	1743.62529	0.4
YAASSYLSLTPEQWK	Ig lambda-2 chain C regions n=4 Tax=Homo s	14	1	P0CG05		0	2.55E-05	2	872.86646	1744.72563	0.43
AMGIMNSFVNDIFER	Histone H2B type 1-J n=102 Tax=Euteleostom	4	1	P06899		0	4.27E-08	2	872.87695	1744.74663	0.46
SAMEPVWNGKEFVPR	dihydrolipoyllysine-residue acetyltransferase	2	1	206577768		0	7.39E-05	2	874.34753	1747.68779	0.41
IAIVNMGSLFQQVAQK	periplasmic chaperone [Escherichia coli CFT0	1	1	26246124		0	1.16E-07	2	874.63782	1748.26836	0.66
GAVPGATGSDLIVKPAVKA	50S ribosomal protein L3 [Proteus mirabilis I	4	1	197287068		0	4.59E-08	2	876.03467	1751.06206	0.03
ENSYADIKNLADFMK	porin [Pseudomonas aeruginosa PAO1]	1	1	15596974		0	1.47E-06	2	880.40222	1759.79717	0.48
SLGFDKVFMYLSDSR	Uncharacterized protein n=3 Tax=Simiiforme	2	1	E9PFJ3		0	1.68E-08	2	883.13159	1765.25591	0.2
LADLRGQNEDQNVGIK	chaperonin GroEL [Escherichia coli CFT073]	1	1	26251035		0	7.65E-05	2	885.89819	1770.78911	0.44
LLAAGIGDALATWFEAR	glycerol dehydrogenase [Escherichia coli CFT	3	1	161486036		0	2.82E-08	2	888.19519	1775.3831	0.22
MAVAESGMGIVEDKVIK	bifunctional acetaldehyde-CoA/alcohol dehy	1	1	26247570		0	7.68E-09	2	889.35437	1777.70146	0.39
IDFSSGWLQDEITLR	outer membrane heme/hemoglobin recepto	1	1	26250130		0	4.04E-07	2	890.87646	1780.74565	0.43
SLGFDKVFmYLSDSR	Uncharacterized protein n=3 Tax=Simiiforme	2	1	E9PFJ3	M9(Oxidation)	0	0.0011	2	891.38831	1781.76933	0.46
MVVTLIHPIAMDDGLR	translation elongation factor Tu [Enterobact	3	1	334126439		0	0.00382	2	890.98462	1780.96196	0.01
ISNIREMLPVLEAVAK	chaperone GroEL [Enterobacter hormaechei	3	3	334123809;15599581;26251035		0	0.00254	2	892.50366	1784.00005	0.49
TNFSLTGPLGDEFSFR	outer membrane receptor FepA [Escherichia	1	1	26246561		0	3.20E-06	2	894.91321	1788.81914	0.48
LAPDDQPWEMGFAASR	putative pesticin receptor [Escherichia coli C	1	1	26248288		0	7.76E-05	2	895.90308	1790.79888	-0.01
SYELPDGQVITIGNER	Uncharacterized protein n=12 Tax=Coeloma	2	1	B4DW52		0	3.71E-06	2	895.97034	1790.9334	0.02
KQLDEGLAGDNVGVLLR	tuf gene product [Streptococcus pneumonia	2	1	116515356		0	4.53E-08	2	899.43567	1797.86406	0.44
GITINTSHVEYDTPTR	translation elongation factor Tu [Enterobact	3	1	334126439		0	2.72E-07	2	902.91992	1804.83257	0.47
SSSSLLPELSAEANPFR	cytochrome d ubiquinol oxidase subunit III [	1	1	26249813		0	0.000624	2	902.91968	1804.83208	-0.04
EGLNVLQYFISTHGAR	DNA-directed RNA polymerase, beta' subuni	2	1	206578905		0	0.0166	2	903.28894	1805.5706	0.32
NIETIINTFHQYSVK	Protein S100-A9 n=5 Tax=Hominoidea RepID	1	1	P06702		0	0.000543	2	904.65381	1808.30034	0.68
ADEAYRKADEALGAAQK	Outer membrane lipoprotein OprI precursor	1	1	15598049		0	0.00708	3	603.17975	1807.52469	0.21
GSLPIALDEVITDGHKR	bifunctional acetaldehyde-CoA/alcohol dehy	1	1	26247570		0	4.02E-07	2	910.88306	1820.75884	-0.11
DLSEELQILEAGLFSR	DNA-directed RNA polymerase, beta subunit	3	1	206579488		0	2.04E-05	2	911.05115	1821.09502	0.58
LMTEFNYNSVMQVPR	ribosomal protein L5 [Klebsiella pneumoniae	3	1	206579163		0	2.72E-06	2	914.96472	1828.92217	0.03
ITTVQEAIDYIVAHQQ	acyl carrier protein [Pseudomonas aerugino	1	1	15598162		0	2.50E-06	2	915.58154	1830.15581	0.61
FLFNNGYADQITSVLK	bifunctional acetaldehyde-CoA/alcohol dehy	1	1	26247570		0	7.87E-08	2	915.79004	1830.5728	0.31
LNIPMLSAAMDTVTEAR	inosine 5'-monophosphate dehydrogenase [	1	1	26248869		0	6.87E-06	2	916.70825	1832.40923	-0.26
QEPSQGTTTFAVTSILR	Ig alpha-1 chain C region n=2 Tax=Homo sap	5	1	P01876		0	7.88E-09	2	918.87573	1836.74419	0.4
STAESIVYSALETLAQR	30S ribosomal protein S7 [Escherichia coli CF	1	1	26249938		0	5.97E-05	2	920.03015	1839.05303	0.05
VGVLSNDFFVNLLDMR	peroxidase/catalase HPI [Escherichia coli CF	1	1	26250714		0	2.35E-08	2	920.42505	1839.84282	0.45
VSLKTALQPMVSALNIR	Uncharacterized protein n=3 Tax=Simiiforme	2	1	E9PFJ3		0	2.48E-06	2	921.49817	1841.98906	0.46
GDWQNEVNVRDFIQK	formate acetyltransferase [Enterobacter hor	2	2	334122625;206577432		0	6.86E-05	2	924.49829	1847.98931	0.04
VDFSKFGEIEEVELGR	dihydrolipoyllysine-residue acetyltransferase	2	1	206577768		0	2.68E-05	2	927.96777	1854.92827	0.5
VSLKTALQPmVSALNIR	Uncharacterized protein n=3 Tax=Simiiforme	2	1	E9PFJ3	M10(Oxidation)	0	9.11E-06	2	929.42212	1857.83696	0.39
AANAGGVATSGLEMAQNAAR	glutamate dehydrogenase [Proteus mirabilis	4	1	197286835		0	4.74E-08	2	930.52856	1860.04985	0.07
EYASFTQEQVDKIFR	bifunctional acetaldehyde-CoA/alcohol dehy	1	1	26247570		0	0.000346	2	931.45874	1861.9102	0.5
FWWENEGVFSmQQR	Myeloperoxidase n=6 Tax=Catarrhini RepID=	1	1	P05164	M11(Oxidation)	0	0.00238	2	930.81482	1860.62236	0.4
GGGIPDLIAIYQDASGNAK	ketol-acid reductoisomerase [Pseudomonas	1	1	15599888		0	1.06E-07	2	930.95435	1860.90141	0.48
QIVANAGDEPSVVVDKVK	chaperonin GroEL [Pseudomonas aeruginosa	1	1	15599581		0	0.000315	2	934.89355	1868.77983	0.38
TFDTPTHPNSLALSADGK	hypothetical protein c1877 [Escherichia coli	1	1	26247735		0	4.32E-06	2	936.82092	1872.63457	0.36
TTPPVLDSDGSFFLYSK	Ig gamma-1 chain C region n=3 Tax=Homo sa	5	1	P01857		0	8.47E-05	2	937.82959	1874.6519	0.37
ALNSIIDVYHKYSLIK	Protein S100-A8 n=5 Tax=Hominoidea RepID	1	1	P05109		0	5.89E-05	2	939.52649	1878.0457	0.5
KVVMTGPSKDNTPMFVK	glyceraldehyde-3-phosphate dehydrogenase	1	1	26248038		0.00105	0.0641	3	627.37921	1880.12308	0.38
EVQLVESGGGLVKPGGSLR	Myosin-reactive immunoglobulin heavy chai	2	1	Q9UL88		0	5.65E-10	2	941.83533	1882.66338	0.31
zIVLTzSPGTLSLSPGzR	Ig kappa chain V-III region B6 n=1 Tax=Homo	1	1	P01619	Z1(E); Z6(Q); Z17(E)	0	9.87E-07	2	942.90222	1884.79717	0.4
MNKSELIDAIAASADIPK	DNA-binding protein HU [Pseudomonas aeru	1	1	15597001		0	4.99E-05	2	943.76733	1886.52739	-0.23
QLAEDPFNNWVALNKK	30S ribosomal protein S1 [Escherichia coli CF	2	1	26246936		0	2.34E-05	2	944.46448	1887.92168	0.47
VFMQPASEGTGIIAGGAMR	30S ribosomal protein S5 [Enterobacter horr	2	1	334125906		0	2.19E-07	2	947.40808	1893.80889	0.44
VTAEDVLKPGTADILVPR	DNA-directed RNA polymerase, beta' subuni	2	1	206578905		0	3.34E-05	2	947.82849	1894.64971	0.29
EIVLTQSPATLSLSPGER	Cryocrystalglobulin CC1 kappa light chain va	2	1	B1N7B8		0	6.38E-06	2	949.91687	1898.82646	0.4
EKSVEELNTELLNLLR	50S ribosomal protein L29 [Proteus mirabilis	2	1	197287076		0	2.06E-06	2	951.07288	1901.13848	0.55
IWDSTDALELKEVPER	dihydrolipoamide dehydrogenase [Escherich	1	1	161486328		0	2.66E-06	2	951.3158	1901.62432	0.33
ATVFIFPDLNTGNTTYK	phosphate acetyltransferase [Enterobacter h	3	1	334124951		0	3.70E-06	2	951.96936	1902.93144	0.48
LVVTPLGSLAFQYAEGIK	fimbrillin MatB [Klebsiella pneumoniae 342]	2	1	206580931		0	1.99E-06	2	953.86951	1906.73174	0.33
TTPPMLDSDGSFFLYSK	Putative uncharacterized protein DKFZp686	1	1	Q6MZU6		0	4.30E-05	2	953.80994	1906.6126	0.36
AFTSEEFTHFLEELTK	phosphopyruvate hydratase [Escherichia col	1	1	26249182		0	3.33E-05	2	965.45117	1929.89507	0.48
TVAAPSVFIFPPSDEQLK	Ig kappa chain C region n=2 Tax=Homo sapie	19	1	P01834		0	0.000557	2	973.89575	1946.78423	0.38
LGHPDTLNQGEFKELVR	Protein S100-A9 n=5 Tax=Hominoidea RepID	1	1	P06702		0	6.59E-05	2	977.40723	1953.80718	0.39
VAPEEHPVLLTEAPLNPK	Uncharacterized protein n=12 Tax=Coeloma	2	1	B4DW52		0	1.07E-05	2	977.9718	1954.93633	0.44
FLSQPFFVAEVFTGSPGK	F0F1 ATP synthase subunit beta [Escherichia	3	2	26250476;15600747		0	2.42E-08	2	979.82031	1958.63335	0.31
VVNIASYQVSPNDVVSIR	30S ribosomal protein S4 [Escherichia coli CF	1	1	26249881		0	0.000118	2	981.10193	1961.19658	0.57
IIELAGFLDSYIPEPER	translation elongation factor Tu [Enterobact	3	1	334126439		0	0.00221	2	981.58618	1962.16509	0.07
VFGTGGTGDHSLGLGASAFGR	outer membrane heme/hemoglobin recepto	1	1	26250130		0	7.59E-10	2	982.90564	1964.804	0.42
ELLSQYDFPGDDTPIVR	translation elongation factor Tu [Enterobact	3	1	334126439		0	0.00793	2	983.45959	1965.91191	0.48
QIGIYSPNGQQYTPQDR	pyruvate dehydrogenase subunit E1 [Escher	2	1	26246055		0	1.82E-05	2	983.34985	1965.69243	0.37
EISPYSIVGLSATWDVTK	outer membrane receptor FepA [Escherichia	1	1	26246561		0	1.63E-07	2	983.52087	1966.03447	0.01
KSTAESIVYSALETLAQR	30S ribosomal protein S7 [Escherichia coli CF	1	1	26249938		0	1.63E-07	2	984.453	1967.89873	0.43
ERPVATGHDEQSWAQNR	peptidoglycan associated lipoprotein OprL p	1	1	15596170		0	2.51E-08	2	991.29321	1981.57915	0.33
WDFAPLQSLELEAGYSR	outer membrane receptor FepA [Escherichia	1	1	26246561		0	1.81E-07	2	991.51013	1982.01299	0.02
SAQGGIINIVTQQPDSTPR	putative pesticin receptor [Escherichia coli C	1	1	26248288		0	0.000209	2	991.8053	1982.60332	0.29
GMGPENTLILIDGKPVSSR	outer membrane receptor FepA [Escherichia	1	1	26246561		0	8.69E-05	3	661.86938	1983.5936	-0.15
VKENSYADIKNLADFMK	porin [Pseudomonas aeruginosa PAO1]	1	1	15596974		0	8.60E-07	2	993.96631	1986.92534	0.46
AKYPDLQIIGGNVATAAGAR	inosine 5'-monophosphate dehydrogenase [	1	1	26248869		0	2.24E-09	2	993.95752	1986.90776	0.42
NAEFMSLAEIEGGIANFGK	dihydrolipoamide succinyltransferase [Pseu	1	1	15596783		0	1.28E-05	2	1000.02979	1999.05229	0.54
IINELEGIFEGAGWNVIK	pyruvate dehydrogenase subunit E1 [Escher	1	1	26246055		0	5.93E-07	2	1001.85376	2002.70024	0.32
DATNKAADIVLQAAIAAGAPK	bifunctional acetaldehyde-CoA/alcohol dehy	1	1	26247570		0	1.21E-05	2	1005.28052	2009.55376	0.23
EIELEDKFENMGAQMVK	chaperone GroEL [Enterobacter hormaechei	3	3	334123809;197286390;26251035		0	1.83E-05	2	1006.32874	2011.65019	0.35
LIQLMNETVDGDYQTFK	pyruvate dehydrogenase subunit E1 [Escher	1	1	26246055		0	6.46E-08	2	1008.40369	2015.8001	0.41
QTVYAFLGDGEMDEPESK	pyruvate dehydrogenase subunit E1 [Escher	2	1	26246055		0	8.75E-07	2	1008.77368	2016.54009	0.33
YDLNLASYGVPGLTFMVR	porin [Pseudomonas aeruginosa PAO1]	1	1	15596155		0	1.38E-05	2	1009.07983	2017.15239	0.56
ILINTPASQGGIGDLYNFK	bifunctional acetaldehyde-CoA/alcohol dehy	1	1	26247570		0	3.71E-08	2	1011.42908	2021.85088	0.39
ALNLQDKQAIVAEVSEVAK	ribosomal protein L10 [Klebsiella pneumonia	3	1	206577086		0	6.40E-09	2	1013.73499	2026.46269	0.17
LAVNLVGPHYFDGDNQLR	putative pesticin receptor [Escherichia coli C	1	1	26248288		0	2.25E-06	2	1014.74939	2028.4915	0.23
DWLQAIESVIREEGVER	pyruvate dehydrogenase subunit E1 [Escher	2	1	26246055		0	0.000194	2	1015.50452	2030.00176	0.48
YDLNLASYGVPGLTFmVR	porin [Pseudomonas aeruginosa PAO1]	1	1	15596155	M16(Oxidation)	0	1.42E-05	2	1016.96448	2032.92168	0.45

Sequence	Protein Descriptions	# Proteins	# Protein Groups	Protein Group Accessions	Modifications	q-Value	PEP	Charge	m/z [Da]	MH+ [Da]	Delta Mass [Da]
QTDELTGLSSLVVLDSAER	DNA-directed RNA polymerase, beta' subuni	2	1	206578905		0	1.96E-10	2	1017.22681	2033.44634	0.2
QSNGETAPNDESINNMLAK	outer membrane heme/hemoglobin recepto	1	1	26250130		0	4.36E-07	2	1017.35327	2033.69927	0.39
TALVANPWQLGNVAAMLER	DNA gyrase subunit A [Escherichia coli CFT0	1	1	26248619		0	2.04E-08	2	1027.9375	2054.86772	0.39
FFESFGDLSTPDAVMGNPK	Mutant beta-globin n=1 Tax=Homo sapiens	4	1	Q9UP81		0	3.39E-07	2	1030.38135	2059.75542	0.4
VVSWYDNEMSYTAQLVR	gap gene product [Streptococcus pneumoni	7	1	116516442		0	6.21E-06	2	1031.03333	2061.05937	0.04
YLVLQGVSPAQLELVSYGK	peptidoglycan associated lipoprotein OprL p	1	1	15596170		0	3.10E-06	2	1032.96582	2064.92436	0.39
DYGVGSDVYSVTSFTELAR	pyruvate dehydrogenase subunit E1 [Escher	2	1	26246055		0	5.64E-08	2	1033.93628	2066.86528	0.45
VLGDKIQLVGDDLFVTNTK	phosphopyruvate hydratase [Escherichia col	1	1	26249182		0	0.000345	3	692.74585	2076.223	0.36
GPMSSLYGSDALGGVVNIITK	colicin I receptor [Klebsiella pneumoniae 34	2	1	206576322		0	5.90E-06	2	1040.35132	2079.69536	0.31
AQVINTNSLSLLTQNNINK	flagellin [Enterobacter hormaechei ATCC 49	3	2	334124672;26248190		0	9.63E-11	2	1042.93445	2084.86162	-0.13
GHYTEGAELVDSVLDVVRK	TUBB2B protein (Fragment) n=16 Tax=Fungi,	4	1	A1L195		0	3.65E-06	3	696.43262	2087.2833	0.07
IDSGLYLGSGYFTAIQNLR	Lactotransferrin n=24 Tax=Hominoidea RepI	1	1	P02788		0	1.20E-08	2	1044.81018	2088.61308	0.27
NATDNAGELISDLQLIYNK	F0F1 ATP synthase subunit gamma [Pseudor	1	1	15600748		0	5.38E-05	2	1046.96289	2092.9185	0.43
APMILALANPEPEILPPLAK	malic enzyme [Escherichia coli CFT073]	2	1	26248830		0	1.22E-07	2	1049.93542	2098.86357	0.33
GIELNIPLVSAAMDTVTEAR	inosine 5'-monophosphate dehydrogenase [	1	1	15598965		0	8.45E-08	2	1051.05212	2101.09697	0.5
VFLENVIRDAVTYTEHAK	Histone H4 n=219 Tax=Eukaryota RepID=H4_	1	1	P62805		0	0.000953	3	702.79718	2106.37699	0.43
QYAPMSVAQQSLVLFAAER	ATP synthase F1, alpha subunit [Klebsiella p	2	1	206580919		0	9.41E-05	2	1055.37061	2109.73393	0.33
AQAQAEIEQELNSVKDALR	F0F1 ATP synthase subunit B [Pseudomonas	1	1	15600751		0	0.000465	2	1056.96643	2112.92558	-0.08
SGETEDATIADLAVGTAAGQIK	phosphopyruvate hydratase [Escherichia col	1	1	26249182		0	1.52E-10	2	1059.79883	2118.59038	0.27
NSTIVFPLPIDMLQGIIGAK	Stomatin n=5 Tax=Mammalia RepID=B1AM7	3	1	B1AM77		0	1.17E-05	2	1064.60461	2128.20195	0.51
mLTELEKALNSIIDVYHK	Protein S100-A8 n=5 Tax=Hominoidea RepID	1	1	P05109	M1(Oxidation)	0	4.32E-05	3	712.02161	2134.05027	0.31
VNAVGYGESRPVADNATAEGR	porin [Pseudomonas aeruginosa PAO1]	1	1	15596974		0	1.73E-07	3	711.82043	2133.44675	0.14
QVDDGDMINPATGSDDLGGTR	putative pesticin receptor [Escherichia coli C	1	1	26248288		0	1.64E-08	2	1067.82068	2134.63408	0.35
VDNALQSGNSQESVTEQDSK	Ig kappa chain C region n=2 Tax=Homo sapie	19	1	P01834		0	1.49E-09	2	1068.82507	2136.64287	0.34
FKNTIDNLTNISENATNAR	flagellin type B [Pseudomonas aeruginosa P	1	1	15596289		0	4.37E-11	2	1068.92981	2136.85234	0.39
ILLNPQDKDGSFSVVITGLR	Polymeric immunoglobulin receptor n=5 Tax	1	1	P01833		0	8.98E-09	2	1087.23059	2173.45391	0.63
MHEGDEGPGHHHKPGLGEGTP	Protein S100-A9 n=5 Tax=Hominoidea RepID	1	1	P06702		0	1.02E-07	2	1088.58765	2176.16802	0.1
NKLNDLEDALQQAKEDLAR	Keratin, type II cytoskeletal 1 n=7 Tax=Euthe	1	1	P04264		0	1.83E-06	3	729.17114	2185.49887	0.46
mHEGDEGPGHHHKPGLGEGTP	Protein S100-A9 n=5 Tax=Hominoidea RepID	1	1	P06702	M1(Oxidation)	0	8.96E-05	2	1096.83044	2192.65361	0.35
GPMSSLYGSDALGGVVNIITKK	colicin I receptor [Klebsiella pneumoniae 34	2	1	206576322		0	0.000371	3	736.7157	2208.13254	0.32
AYKAPSLYQTNPNYILYSK	outer membrane receptor FepA [Escherichia	2	2	26246561;334122958		0	0.000264	3	745.74628	2235.22428	0.36
SGLNAENYENFIQTDAAINR	serine endoprotease [Escherichia coli CFT07	2	1	26246108		0	1.03E-06	2	1120.95667	2240.90605	0.42
LDSAVTNLNNTTTNLSEAQSR	flagellin [Escherichia coli CFT073]	1	1	26248190		0	7.16E-09	2	1125.49146	2249.97563	0.44
TNVPHIFAIGDIVGQPMLAHK	dihydrolipoyl dehydrogenase [Enterobacter	2	2	334123429;161486328		0	1.26E-07	2	1130.25964	2259.51201	0.65
ARLEEGLNPNAILVPQQGVTR	acriflavin resistance protein A [Escherichia c	1	1	26246476		0	1.09E-06	2	1138.0498	2275.09233	-0.08
TNVPHIFAIGDIVGQPmLAHK	dihydrolipoyl dehydrogenase [Enterobacter	2	2	334123429;161486328	M17(Oxidation)	0	6.95E-05	3	759.08319	2275.23502	0.34
VLPGLNIENSGNMLFSTISLR	putative pesticin receptor [Escherichia coli C	1	1	26248288		0	1.42E-09	2	1138.50659	2276.00591	0.4
EMLLDAMENPEKYPQLTIR	formate acetyltransferase [Klebsiella pneum	1	1	206577432		0	0.000402	3	764.73132	2292.17942	0.35
QILLDTYYGRDYVEGETAAK	bifunctional acetaldehyde-CoA/alcohol deh	1	1	26247570		0	0.000128	2	1153.67859	2306.3499	0.61
MPSAVGYQPTLAEEMGVLQER	F0F1 ATP synthase subunit beta [Escherichia	3	2	26250476;15600747		0	2.29E-06	2	1153.94482	2306.88237	0.38
QSNNKYAASSYLSLTPEQWK	Ig lambda-2 chain C regions n=4 Tax=Homo	14	1	P0CG05		0	3.66E-07	2	1158.53223	2316.05718	0.46
AVSNEIVRFPTDQLTPDQER	Myeloperoxidase n=6 Tax=Catarrhini RepID=	1	1	P05164		0	1.23E-06	3	772.65198	2315.94138	0.26
MNKSELIDAIAASADIPKAVAGR	DNA-binding protein HU [Pseudomonas aer	1	1	15597001		0	2.09E-09	3	781.50488	2342.5001	0.42
DGAALGYSHGFNIVEVGEQIRK	ketol-acid reductoisomerase [Escherichia co	3	1	161486053		0	0.000154	3	787.78223	2361.33213	0.38
AANDDLLNSFWLLDSEKGEAR	autonomous glycyl radical cofactor GrcA [Es	2	1	161486147		0	0.000911	3	789.12012	2365.3458	0.4
SKFNLSGPIQDGLLYGSVTLLR	putative pesticin receptor [Escherichia coli C	1	1	26248288		0	1.97E-06	2	1189.98486	2378.96245	0.33
GGGGGGYGSGGSSYGSGGGSYGSGGGGGGGR	Keratin, type II cytoskeletal 1 n=7 Tax=Euthe	1	1	P04264		0	2.32E-12	2	1192.83496	2384.66265	0.36
VVINKDTTTIIDGVGEEAAIQGR	chaperone GroEL [Enterobacter hormaechei	2	2	334123809;26251035		0	1.70E-07	2	1200.52356	2400.03984	0.38
ANDAAGDGTTTATVLAQAIVNEGLK	chaperonin GroEL [Pseudomonas aeruginos	1	1	15599581		0	9.45E-10	2	1201.44824	2401.88921	0.33
ANDAAGDGTTTATVLAQAIITEGLK	chaperone GroEL [Enterobacter hormaechei	2	2	334123809;26251035		0	2.40E-10	2	1201.92896	2402.85063	0.3
NEQDGGDLVYFQGHISPGVYAR	pyruvate dehydrogenase subunit E1 [Escher	2	1	26246055		0	5.08E-06	3	808.42291	2423.25418	0.37
QILLDTYYGRDYVEGETAAKK	bifunctional acetaldehyde-CoA/alcohol deh	1	1	26247570		0	0.0329	3	812.1571	2434.45676	0.41
ALQAIEGLKGDNEEQNVGIALLR	chaperonin GroEL [Pseudomonas aeruginos	1	1	15599581		0	4.68E-08	2	1226.52759	2452.0479	0.36
GAELANSFKPDVIIALGGGSPMDAAK	bifunctional acetaldehyde-CoA/alcohol deh	1	1	26247570		0	1.71E-08	2	1265.65674	2530.3062	0.5
LADIGRVEESLGYNAIAAGFQGQR	L-fucose isomerase [Escherichia coli CFT073]	1	1	26249208		0	2.35E-08	2	1268.59399	2536.18071	0.44
VLESAIANAEHNDGADIDDLKVTK	50S ribosomal protein L22 [Escherichia coli C	1	1	26249906		0	2.17E-07	3	847.14618	2539.42398	0.39
GFYPSDIAVEWESNGQPENNYK	Putative uncharacterized protein DKFZp686	6	2	Q6MZU6;P01857		0	0.0013	2	1272.88184	2544.7564	0.31
WLPAEYEDGFSLPYGWTPGVKR	Myeloperoxidase n=6 Tax=Catarrhini RepID=	1	1	P05164		0	0.000165	3	857.11169	2569.32053	0.36
QYPSTSTTVEGHTDSVGTDAYNQK	porin [Pseudomonas aeruginosa PAO1]	1	1	15596974		0	1.35E-05	3	863.11145	2587.3198	0.39
ALIVTDRFLFNNGYADQITSVLK	bifunctional acetaldehyde-CoA/alcohol deh	1	1	26247570		0	1.79E-05	3	867.21277	2599.62375	0.41
LYGNLDKTQADAWDINQGHQSAR	outer membrane receptor FepA [Escherichia	1	1	26246561		0	9.60E-10	3	868.07886	2602.22202	0.33
DSTIQVVENGESSQGRFSVQMFR	Uncharacterized protein n=3 Tax=Simiiforme	2	1	E9PFJ3		0	0.00724	2	1301.75952	2602.51177	0.64
GLLTEAELDDIFSVQNLMHPAYK	aspartate ammonia-lyase [Escherichia coli C	1	1	26251030		0	1.63E-05	3	869.21167	2605.62046	0.44
ALQAIEGLKGDNEEQNVGIALLRR	chaperonin GroEL [Pseudomonas aeruginos	1	1	15599581		0	0.000311	3	870.09338	2608.2656	0.28
NANDGISLAQTAEGALQQSTNILQR	flagellin type B [Pseudomonas aeruginosa P	1	1	15596289		0	0.000684	2	1307.56848	2614.12969	0.4
AQVQEIAETKMPDLNAANIESAMR	50S ribosomal protein L11 [Streptococcus pr	1	1	169834089		0	5.03E-07	3	877.80566	2631.40244	0.37
FQLFGSPSGQKDLLFKDSAIGFSR	Lactotransferrin n=24 Tax=Hominoidea RepI	1	1	P02788		0	2.26E-05	3	882.60925	2645.81321	0.15
KGAELANSFKPDVIIALGGGSPMDAAK	bifunctional acetaldehyde-CoA/alcohol deh	1	1	26247570		0	0.000402	3	886.87585	2658.61301	0.41
ALNIENIIAEIKEASILELNDLVK	rplL gene product [Streptococcus pneumoni	2	1	116516621		0	1.37E-05	2	1333.90442	2666.80156	0.65
DGSVVVLGYTDRIGSDAYNQALSER	outer membrane protein A [Escherichia coli	1	1	26246978		0	6.72E-06	3	896.15503	2686.45053	0.38
YINIGVAVDTPNGLVVPVFKDVNKK	dihydrolipoyllysine-residue acetyltransferas	2	1	206577768		0	4.99E-05	3	900.71704	2700.13657	0.21
IVIKVNEDEMYPGEAGIDIYNLTK	DNA-directed RNA polymerase, beta subuni	3	1	206579488		0	0.000308	3	909.23364	2725.68637	0.44
AGRPLLIVAEDVEGEALATLVVNNMR	chaperonin GroEL [Pseudomonas aeruginos	1	1	15599581		0	3.95E-05	3	917.87292	2751.60422	0.37
LVSWYDNETGYSNKVLDLIAHISK	glyceraldehyde-3-phosphate dehydrogenase	1	1	26248038		0	1.03E-05	3	922.93542	2766.79172	0.46
EFFGSSQLSQFMDQNNPLSEITHK	DNA-directed RNA polymerase, beta subuni	3	1	206579488		0	1.92E-07	3	929.13318	2785.38498	0.36
SGPFGQIFRPDNFVFGQSGAGNNWAK	TUBB2B protein (Fragment) n=16 Tax=Fungi,	4	1	A1L195		0	8.65E-07	3	933.84949	2799.53391	0.4
VAQGYKPSGYNIVPTAGLDAKPFVAEK	putative pesticin receptor [Escherichia coli C	1	1	26248288		0	4.20E-05	3	941.28882	2821.8519	0.45
AVIESENSAERDQLLENLQEGMEVK	30S ribosomal protein S1 [Escherichia coli C	2	1	26246936		0	3.05E-06	3	944.72058	2832.14719	0.26
SLMFmQWGQLLDHDLDFTPEPAAR	Myeloperoxidase n=6 Tax=Catarrhini RepID=	1	1	P05164	M5(Oxidation)	0	0.000379	3	945.83362	2835.4863	0.39
DGSVVVLGYTDRIGSDAYNQALSERR	outer membrane protein A [Escherichia coli	1	1	26246978		0	0.000341	3	948.25134	2842.73948	0.44
MTESFAQLFEESLKEIETRPGSIVR	30S ribosomal protein S1 [Escherichia coli C	2	1	26246936		0	2.77E-07	3	966.9104	2898.71665	0.42
AIAQVGTISANSDETVGTLIAQAmEKVGK	60 Kda chaperonin [Proteus mirabilis HI432	1	1	197286390	M24(Oxidation)	0	0.0138	3	973.65002	2918.93552	0.14
SASANNVSSTVVSAPELSDAGVTASDKLPR	putative pesticin receptor [Escherichia coli C	1	1	26248288		0	0.000516	3	977.95691	2931.85617	0.46
GQPVTGSEKDEVSPYSIVGLSATWDVTK	ferrienterobactin receptor [Enterobacter ho	1	1	334122958		0	0.00237	3	984.3457	2951.02256	0.18
NLTDYGAFVDLGGVDGLLHITDMAWKR	30S ribosomal protein S1 [Escherichia coli C	2	1	26246936		0	2.48E-06	3	993.12073	2977.34763	-0.05
RAVIESENSAERDQLLENLQEGMEVK	30S ribosomal protein S1 [Escherichia coli C	2	1	26246936		0	0.000108	3	996.84253	2988.51303	0.35
GMNTAVGDEGGYAPNLGSNAEALAVIAEAVK	phosphopyruvate hydratase [Escherichia col	1	1	26249182		0	4.08E-05	3	997.4657	2990.38254	0.31
YQELKDIIAILGMDELSEEDKLVVAR	F0F1 ATP synthase subunit beta [Escherichia	2	1	26250476		0	1.85E-08	3	997.88464	2991.63938	0.35
IQKLTTYRNAIPTQSVLTITSNVVYGK	formate acetyltransferase [Enterobacter hor	1	1	334122625		0	0.00638	3	1003.87305	3009.60459	0.31
VPTPTGSVTELVAVLEKNVTVDEVNAAMK	gap gene product [Streptococcus pneumoni	7	1	116516442		0	1.20E-06	3	1004.9845	3012.93894	0.45
TAAFSLPLLQNLDPELKAPQILVLAPTR	ATP-dependent RNA helicase DeaD [Escheri	1	1	161486111		0	4.70E-07	3	1010.88086	3030.62802	0.3
QIALTEQGAQWLEENREQVEMIEER	hypothetical protein c3824 [Escherichia coli	1	1	26249655		0	0.000349	3	1010.89661	3030.67527	0.4
SNTGIYAQVIDDVAGVTLASASTLDKEVSK	rplR gene product [Streptococcus pneumoni	3	1	116515871		0	4.12E-06	3	1018.48792	3053.44919	0.29
TIDDLKNQILNLTTDNANILLQIDNAR	Keratin, type I cytoskeletal 10 n=12 Tax=Eut	1	1	P13645		0	1.91E-06	3	1018.67322	3054.0051	0.46
GIMEGEYDHLPEQAFYMVGSIEEAVEK	F0F1 ATP synthase subunit beta [Escherichia	2	1	26250476		0	7.90E-08	3	1024.89294	3072.66428	0.42
ASGVSGNLSSVGSDTLANLMTMWAEEYKR	phosphate ABC transporter periplasmic pho	1	1	15600562		0	1.19E-06	3	1025.70996	3075.11533	0.22
TVINFDNAIIAAGSRPIELPFIPHEDPR	dihydrolipoyl dehydrogenase [Enterobacter	1	1	334123429		0	0.000335	3	1035.34949	3104.03391	0.47
VGKEGVITVEEGSGLENELSVVEGMQFDR	chaperonin GroEL [Pseudomonas aeruginos	1	1	15599581		0	2.12E-09	3	1036.92871	3108.77158	0.42
GYLSPYFINKPETGAVELESPFILLADK	chaperone GroEL [Enterobacter hormaechei	2	2	334123809;26251035		0	4.72E-05	3	1038.29858	3112.8812	0.42
AAAFEGELIPASQIDRLATLPTYEEAIAR	ribosomal protein L10 [Klebsiella pneumoni	3	1	206577086		0	0.00294	3	1039.77698	3117.31638	0.23
VGKEGVITVEDGTGLEDELDVVEGMQFDR	chaperone GroEL [Enterobacter hormaechei	1	1	334123809		0	2.48E-07	3	1046.36121	3137.06906	0.19
ELNSTQPFQLLETSPQFIYQAQSGLTGR	putative inner membrane protein transloca	1	1	26250446		0	0.000367	3	1052.31494	3154.93027	0.45
TTGIVMDSGDGVTHTVPIYEGYALPHAILR	Uncharacterized protein n=12 Tax=Coeloma	2	1	B4DW52		0	2.72E-05	3	1062.12561	3184.36228	0.25
FYTNYWVPNPNLRPETNETQEYGFGLR	outer membrane heme/hemoglobin recepto	1	1	26250130		0	0.000249	3	1102.95447	3306.84885	0.43
VIEHIMEDLDTNADKQLSFEEFIMLMAR	Protein S100-A9 n=5 Tax=Hominoidea RepID	1	1	P06702		0	1.21E-09	3	1113.9563	3339.85434	0.41
VIEHIMEDLDTNADKQLSFEEFImLMAR	Protein S100-A9 n=5 Tax=Hominoidea RepID	1	1	P06702	M24(Oxidation)	0	4.67E-09	3	1119.15222	3355.44211	0.28

2DLCSCI_36 Protein Summary

index	Prot_hit	Accession	Description	Mascot Score	Coverage	# Peptides	# PSMs	# AAs	MW [kDa]	calc. pI
1	1	334124896	outer membrane protein N [Enterobacter hormaechei ATCC 49	165.28	11.02%	2	3	363	40	4.61
2	2	26246561	outer membrane receptor FepA [Escherichia coli CFT073]	172.29	2.28%	1	2	746	82	5.45
3	3	26248604	outer membrane porin protein C [Escherichia coli CFT073]	266.82	10.67%	2	5	375	41.2	4.83
4	4	26249182	phosphopyruvate hydratase [Escherichia coli CFT073]	166.14	3.70%	1	3	432	45.6	5.48
5	5	P05109	Protein S100-A8 n=5 Tax=Hominoidea RepID=S10A8_HUMAN	326.4	37.63%	5	10	93	10.8	7.03
6	6.1	P06702	Protein S100-A9 n=5 Tax=Hominoidea RepID=S10A9_HUMAN	2872.03	84.21%	10	60	114	13.2	6.13
7	6.2	116517025	unnamed protein product [Streptococcus pneumoniae D39]	48.18	3.13%	1	1	192	22	8.94
8	6.3	225858457	uracil DNA glycosylase family protein [Streptococcus pneumon	48.18	3.13%	1	1	192	22.1	8.69
9	6.4	15900532	hypothetical protein SP_0624 [Streptococcus pneumoniae TIGI	48.18	3.13%	1	1	192	22.1	8.69
10	6.5	182683555	hypothetical protein SPCG_0585 [Streptococcus pneumoniae C	48.18	3.13%	1	1	192	22.1	8.69
11	6.6	307067239	hypothetical protein SPAP_0610 [Streptococcus pneumoniae A	48.18	3.13%	1	1	192	22.1	8.69
12	6.7	168485735	uracil DNA glycosylase family protein [Streptococcus pneumon	48.18	3.13%	1	1	192	22.1	8.69
13	6.8	149002306	hypothetical protein CGSSp14BS69_01654 [Streptococcus pneu	48.18	3.09%	1	1	194	22.3	7.55
14	6.9	148984004	hypothetical protein CGSSp3BS71_07719 [Streptococcus pneur	48.18	3.09%	1	1	194	22.3	8.66
15	7	P80511	Protein S100-A12 n=4 Tax=Hominoidea RepID=S10AC_HUMAN	625.58	36.96%	4	14	92	10.6	6.25
16	8.1	E9PFJ3	Uncharacterized protein n=3 Tax=Simiiformes RepID=E9PFJ3_H	374.35	5.71%	3	8	578	63.3	5.31
17	8.2	P07911	Uromodulin n=2 Tax=Catarrhini RepID=UROM_HUMAN	374.35	5.16%	3	8	640	69.7	5.24
18	9.1	E9PG30	Uncharacterized protein n=21 Tax=Coelomata RepID=E9PG30_	104.41	9.01%	2	2	333	37.1	5.54
19	9.2	P62736	Actin, aortic smooth muscle n=27 Tax=Eukaryota RepID=ACTA_	104.41	7.96%	2	2	377	42	5.39
20	9.3	P63261	Actin, cytoplasmic 2 n=1334 RepID=ACTG_HUMAN	104.41	8.00%	2	2	375	41.8	5.48
21	9.4	B4DW52	Uncharacterized protein n=12 Tax=Coelomata RepID=B4DW52_	104.41	8.65%	2	2	347	38.6	5.35
22	9.5	B4E335	Uncharacterized protein n=3 Tax=Coelomata RepID=B4E335_H	104.41	8.55%	2	2	351	39.2	5.59
23	9.6	F5H0N0	Uncharacterized protein n=11 Tax=Simiiformes RepID=F5H0N0	104.41	9.01%	2	2	333	37.4	5.58
24	9.7	Q5T8M7	Actin, alpha 1, skeletal muscle n=5 Tax=Simiiformes RepID=Q5T	104.41	8.77%	2	2	342	37.8	5.58
25	10.1	F6PJX6	Uncharacterized protein n=6 Tax=Eutheria RepID=F6PJX6_HOR	97.83	9.09%	1	1	308	35.6	4.72
26	10.2	P13646	Keratin, type I cytoskeletal 13 n=13 Tax=Simiiformes RepID=K1(	97.83	6.11%	1	1	458	49.6	4.96
27	10.3	P13646-3	Isoform 3 of Keratin, type I cytoskeletal 13 n=7 Tax=Eutheria Re	97.83	6.67%	1	1	420	45.8	4.88
28	11.1	P06899	Histone H2B type 1-J n=102 Tax=Euteleostomi RepID=H2B1J_HI	112.51	11.90%	1	1	126	13.9	10.32
29	11.2	P62807	Histone H2B type 1-C/E/F/G/I n=44 Tax=Euteleostomi RepID=H	112.51	11.90%	1	1	126	13.9	10.32
30	11.3	B4DR52	Histone H2B n=5 Tax=Euarchontoglires RepID=B4DR52_HUMAI	112.51	9.04%	1	1	166	18	10.32
31	11.4	P58876	Histone H2B type 1-D n=136 Tax=Coelomata RepID=H2B1D_HU	112.51	11.90%	1	1	126	13.9	10.32
32	12	P05164	Myeloperoxidase n=6 Tax=Catarrhini RepID=PERM_HUMAN	661.02	10.87%	5	13	745	83.8	8.97
33	13	P02788	Lactotransferrin n=24 Tax=Hominoidea RepID=TRFL_HUMAN	334.66	5.35%	3	6	710	78.1	8.12

2DLCSCI_36 Peptide Summary

Sequence	Protein Descriptions	# Proteins	# Protein Groups	Protein Group Accessions	Modifications	q-Value	PEP	Charge	m/z [Da]	MH+ [Da]	Delta Mass [Da]
mSQIER	Protein S100-A9 n=5 Tax=Hominoidea RepID=S10A	9	1	P06702	M1(Oxidation)	0.00761	0.0502	2	390.44839	779.88951	0.26
mLTELEK	Protein S100-A8 n=5 Tax=Hominoidea RepID=S10A	1	1	P05109	M1(Oxidation)	0.00761	0.0764	2	440.44702	879.88677	0.22
KDLQNFLKK	Protein S100-A9 n=5 Tax=Hominoidea RepID=S10A	1	1	P06702		0.00589	0.0137	2	567.62634	1134.24541	0.29
GHFDTLSKGELK	Protein S100-A12 n=4 Tax=Hominoidea RepID=S10.	1	1	P80511		0.00761	0.0315	2	666.58105	1332.15483	0.23
IGLDLPALNMQR	Myeloperoxidase n=6 Tax=Catarrhini RepID=PERM_	1	1	P05164		0.00761	0.0246	2	671.40002	1341.79277	0.53
TALQPMVSALNIR	Uncharacterized protein n=3 Tax=Simiiformes RepI	2	1	E9PFJ3		0	5.76E-05	2	707.58606	1414.16484	0.19
GNFHAVYRDDLK	Protein S100-A8 n=5 Tax=Hominoidea RepID=S10A	1	1	P05109		0	0.00263	2	718.06683	1435.12639	0.21
LGHPDTLNQGEFK	Protein S100-A9 n=5 Tax=Hominoidea RepID=S10A	1	1	P06702		0	5.25E-06	1	1455.74548	1455.74548	0.02
YGHTLIQPFMFR	Myeloperoxidase n=6 Tax=Catarrhini RepID=PERM_	1	1	P05164		0	0.00853	2	755.56805	1510.12883	0.18
SRDHGLPGYNAWR	Myeloperoxidase n=6 Tax=Catarrhini RepID=PERM_	1	1	P05164		0.00761	0.0314	2	765.04639	1529.0855	0.17
ILLAELEQLKGQGK	Vimentin n=45 Tax=Amniota RepID=VIME_HUMAN	1	1	P08670		0	0.000154	2	770.6803	1540.35332	0.22
SVNGKEDAIWNLLR	Lactotransferrin n=24 Tax=Hominoidea RepID=TRFI	1	1	P02788		0	1.30E-05	2	808.16284	1615.31841	0.23
QLSFEEFIMLMAR	Protein S100-A9 n=5 Tax=Hominoidea RepID=S10A	1	1	P06702		0	0.000135	2	808.49487	1615.98247	0.59
LDLAGRDLTDYLMK	Uncharacterized protein n=21 Tax=Coelomata RepI	7	1	E9PG30		0	0.000165	2	812.8728	1624.73833	0.45
DSTIQVVENGESSQGR	Uncharacterized protein n=3 Tax=Simiiformes RepI	2	1	E9PFJ3		0	1.23E-06	2	853.78229	1706.5573	0.38
VIEHIMEDLDTNADK	Protein S100-A9 n=5 Tax=Hominoidea RepID=S10A	1	1	P06702		0	2.65E-06	2	872.3988	1743.79033	0.48
AMGIMNSFVNDIFER	Histone H2B type 1-J n=102 Tax=Euteleostomi RepI	4	1	P06899		0	5.75E-07	2	872.91675	1744.82622	0.5
SYELPDGQVITIGNER	Uncharacterized protein n=21 Tax=Coelomata RepI	7	1	E9PG30		0	2.00E-06	2	896.35254	1791.6978	0.4
NIETIINTFHQYSVK	Protein S100-A9 n=5 Tax=Hominoidea RepID=S10A	1	1	P06702		0	0.000185	2	904.62744	1808.24761	0.65
FQLFGSPSGQKDLLFK	Lactotransferrin n=24 Tax=Hominoidea RepID=TRFI	1	1	P02788		0	3.02E-07	2	906.67505	1812.34282	0.19
VSLKTALQPMVSALNIR	Uncharacterized protein n=3 Tax=Simiiformes RepI	2	1	E9PFJ3		0	7.56E-07	2	921.43396	1841.86064	0.4
ALNSIIDVYHKYSLIK	Protein S100-A8 n=5 Tax=Hominoidea RepID=S10A	1	1	P05109		0	3.27E-06	2	939.49988	1877.99248	0.47
AFTSEEFTHFLEELTK	phosphopyruvate hydratase [Escherichia coli CFT07	1	1	26249182		0	5.93E-06	2	965.27869	1929.5501	0.31
LGHPDTLNQGEFKELVR	Protein S100-A9 n=5 Tax=Hominoidea RepID=S10A	1	1	P06702		0	0.00226	2	977.44202	1953.87676	0.43
IIELAGFLDSYIPEPER	translation elongation factor Tu [Enterobacter horr	3	1	334126439		0	0.000835	2	981.95593	1962.90459	0.44
WDFAPLQSLELEAGYSR	outer membrane receptor FepA [Escherichia coli CF	1	1	26246561		0	1.88E-07	2	992.0614	1983.11553	0.58
LGHPDTLNQGEFKELVRK	Protein S100-A9 n=5 Tax=Hominoidea RepID=S10A	1	1	P06702		0	1.12E-05	2	1041.49731	2081.98735	0.44
MLTELEKALNSIIDVYHK	Protein S100-A8 n=5 Tax=Hominoidea RepID=S10A	1	1	P05109		0	0.00079	3	706.68909	2118.05271	0.31
mLTELEKALNSIIDVYHK	Protein S100-A8 n=5 Tax=Hominoidea RepID=S10A	1	1	P05109	M1(Oxidation)	0	9.29E-08	2	1067.60022	2134.19316	0.53
MHEGDEGPGHHHKPGLGEGTP	Protein S100-A9 n=5 Tax=Hominoidea RepID=S10A	1	1	P06702		0	1.86E-05	2	1088.92346	2176.83965	0.44
mHEGDEGPGHHHKPGLGEGTP	Protein S100-A9 n=5 Tax=Hominoidea RepID=S10A	1	1	P06702	M1(Oxidation)	0	0.000121	2	1096.91199	2192.8167	0.43
AVSNEIVRFPTDQLTPDQER	Myeloperoxidase n=6 Tax=Catarrhini RepID=PERM_	1	1	P05164		0	0.0104	2	1158.47742	2315.94756	0.39
YDANNIYLAAQYTQTYNATR	outer membrane protein N [Enterobacter hormaec	2	2	334124896;26248604		0	9.03E-08	2	1177.8136	2354.61992	0.26
TKLEEHLEGIVNIFHQYSVR	Protein S100-A12 n=4 Tax=Hominoidea RepID=S10.	1	1	P80511		0	2.25E-06	2	1206.62073	2412.23418	-0.02
NMSTYVDYKINLLDDNQFTR	outer membrane porin protein C [Escherichia coli C	1	1	26248604		0	5.39E-06	2	1225.9967	2450.98613	0.41
NMSTYVDYKINLLDENDFTR	outer membrane protein N [Enterobacter hormaec	1	1	334124896		0.00761	0.0229	3	818.0011	2451.98874	0.28
tKLEEHLEGIVNIFHQYSVR	Protein S100-A12 n=4 Tax=Hominoidea RepID=S10.	1	1	P80511	N-Term(Acetyl)	0	4.97E-08	3	819.11005	2455.31559	0.35
TKLEEHLEGIVNIFHQYSVRK	Protein S100-A12 n=4 Tax=Hominoidea RepID=S10.	1	1	P80511		0	1.04E-06	2	1271.0553	2541.10332	0.37
MTKLEEHLEGIVNIFHQYSVR	Protein S100-A12 n=4 Tax=Hominoidea RepID=S10.	1	1	P80511		0	0.00186	3	848.33832	2543.0004	-0.1
SQYEQLAEQNRKDAEAWFNEK	Keratin, type I cytoskeletal 10 n=12 Tax=Eutheria R	1	1	P13645		0	0.00205	3	862.19165	2584.5604	0.12
FQLFGSPSGQKDLLFKDSAIGFSR	Lactotransferrin n=24 Tax=Hominoidea RepID=TRFI	1	1	P02788		0	1.75E-07	2	1323.60083	2646.19438	0.41
mLTELEKALNSIIDVYHKYSLIK	Protein S100-A8 n=5 Tax=Hominoidea RepID=S10A	1	1	P05109	M1(Oxidation)	0	6.35E-07	3	913.58215	2738.73191	0.42
SLMFMQWGQLLDHDLDFTPEPAAR	Myeloperoxidase n=6 Tax=Catarrhini RepID=PERM_	1	1	P05164		0	2.80E-06	2	1410.09778	2819.18828	0.43
DLGEELEALKTELEDTLDSTAAQQELR	Myosin-9 n=67 Tax=Tetrapoda RepID=MYH9_HUM	1	1	P35579		0.00761	0.101	3	1006.88245	3018.63279	0.38
VLDELTLSKTDLEMQIESLNEELAYMKK	Uncharacterized protein n=6 Tax=Eutheria RepID=F	3	1	F6PJX6		0	1.70E-06	3	1095.61755	3284.83811	0.39
VIEHIMEDLDTNADKQLSFEEFIMLMAR	Protein S100-A9 n=5 Tax=Hominoidea RepID=S10A	1	1	P06702		0	1.88E-06	2	1670.20459	3339.4019	0.4
VIEHIMEDLDTNADKQLSFEEFImLMAR	Protein S100-A9 n=5 Tax=Hominoidea RepID=S10A	1	1	P06702	M24(Oxidation)	0	0.00594	2	1678.2832	3355.55913	0.48

2DLCSCI_1 Protein Summary

index	Prot_hit	Accession	Description	Mascot Score	Coverage	# Peptides	# PSMs	# AAs	MW [kDa]	calc. pI
1	1	P62805	Histone H4 n=219 Tax=Eukaryota RepID=H4_HUMAN	114.77	39.81%	3	3	103	11.4	11.36
2	2	P35579	Myosin-9 n=67 Tax=Tetrapoda RepID=MYH9_HUMAN	128.95	1.58%	2	2	1960	226.4	5.6
3	3.1	Q3BDU5	Rhabdomyosarcoma antigen MU-RMS-40.12 n=10 Tax=Theria R	102.03	3.08%	1	1	487	55.6	6.65
4	3.2	P02545	Prelamin-A/C n=44 Tax=Eutheria RepID=LMNA_HUMAN	102.03	2.26%	1	1	664	74.1	7.02
5	3.3	Q5TCI9	Lamin A/C (Fragment) n=3 Tax=Catarrhini RepID=Q5TCI9_HUM	102.03	2.92%	1	1	513	57.8	6.18
6	4	P15924	Desmoplakin n=28 Tax=Mammalia RepID=DESP_HUMAN	152.25	1.60%	3	4	2871	331.6	6.81
7	5	P04264	Keratin, type II cytoskeletal 1 n=7 Tax=Eutheria RepID=K2C1_HU	3443.04	36.49%	26	79	644	66	8.12
8	6	P35527	Keratin, type I cytoskeletal 9 n=4 Tax=Catarrhini RepID=K1C9_H	383.7	7.54%	2	5	623	62	5.24
9	7	P13645	Keratin, type I cytoskeletal 10 n=12 Tax=Eutheria RepID=K1C10_	552.7	20.03%	10	19	584	58.8	5.21
10	8	P02533	Keratin, type I cytoskeletal 14 n=8 Tax=Eutheria RepID=K1C14_	493.74	14.41%	7	23	472	51.5	5.16
11	9	P05109	Protein S100-A8 n=5 Tax=Hominoidea RepID=S10A8_HUMAN	109.15	17.20%	2	3	93	10.8	7.03
12	10	P35908	Keratin, type II cytoskeletal 2 epidermal n=7 Tax=Simiiformes R	1112.85	15.81%	10	34	639	65.4	8
13	11	P12035	Keratin, type II cytoskeletal 3 n=7 Tax=Catarrhini RepID=K2C3_	696.4	13.69%	9	31	628	64.4	6.48
14	12	P02538	Keratin, type II cytoskeletal 6A n=25 Tax=Euarchontoglires RepI	1957.39	30.85%	22	74	564	60	8
15	13	Q8N1N4	Keratin, type II cytoskeletal 78 n=7 Tax=Hominoidea RepID=K2C	371.67	7.31%	4	13	520	56.8	6.02
16	14	P06702	Protein S100-A9 n=5 Tax=Hominoidea RepID=S10A9_HUMAN	261.19	28.95%	3	7	114	13.2	6.13
17	15	O60437	Periplakin n=3 Tax=Hominoidea RepID=PEPL_HUMAN	542.53	9.00%	10	15	1756	204.6	5.6
18	16	P62937	Peptidyl-prolyl cis-trans isomerase A n=98 Tax=Theria RepID=P	219.98	24.24%	3	7	165	18	7.81
19	17.1	E9PFJ3	Uncharacterized protein n=3 Tax=Simiiformes RepID=E9PFJ3_H	694.38	7.96%	4	20	578	63.3	5.31
20	17.2	P07911	Uromodulin n=2 Tax=Catarrhini RepID=UROM_HUMAN	694.38	7.19%	4	20	640	69.7	5.24
21	18.1	F5H0N0	Uncharacterized protein n=11 Tax=Simiiformes RepID=F5H0N0_	652.16	23.12%	8	21	333	37.4	5.58
22	18.2	P63261	Actin, cytoplasmic 2 n=1334 RepID=ACTG_HUMAN	652.16	20.53%	8	21	375	41.8	5.48
23	18.3	B4DW52	Uncharacterized protein n=12 Tax=Coelomata RepID=B4DW52_	652.16	22.19%	8	21	347	38.6	5.35
24	18.4	B4E335	Uncharacterized protein n=3 Tax=Coelomata RepID=B4E335_HU	652.16	21.94%	8	21	351	39.2	5.59
25	19	A4FUA1	GUCA1B protein (Fragment) n=1 Tax=Homo sapiens RepID=A4F	1207.45	68.00%	4	23	75	8.6	5.41
26	20	P13646	Keratin, type I cytoskeletal 13 n=13 Tax=Simiiformes RepID=K1C	2872.19	50.00%	22	96	458	49.6	4.96
27	21.1	B4DRW1	cDNA FLJ55805, highly similar to Keratin, type II cytoskeletal 4 n	3272.98	54.01%	27	94	474	51.7	6.81
28	21.2	P19013	Keratin, type II cytoskeletal 4 n=14 Tax=Simiiformes RepID=K2C	3272.98	47.94%	27	94	534	57.2	6.61
29	22.1	P06899	Histone H2B type 1-J n=102 Tax=Euteleostomi RepID=H2B1J_HU	111.17	11.11%	1	2	126	13.9	10.32
30	22.2	P62807	Histone H2B type 1-C/E/F/G/I n=44 Tax=Euteleostomi RepID=H	111.17	11.11%	1	2	126	13.9	10.32
31	22.3	Q96A08	Histone H2B type 1-A n=22 Tax=Eutheria RepID=H2B1A_HUMA	111.17	11.02%	1	2	127	14.2	10.32
32	22.4	B4DR52	Histone H2B n=5 Tax=Euarchontoglires RepID=B4DR52_HUMAN	111.17	8.43%	1	2	166	18	10.32
33	22.5	P58876	Histone H2B type 1-D n=136 Tax=Coelomata RepID=H2B1D_HU	111.17	11.11%	1	2	126	13.9	10.32
34	23	P04792	Heat shock protein beta-1 n=6 Tax=Simiiformes RepID=HSPB1_	344.37	33.17%	5	9	205	22.8	6.4
35	24.1	E7EUJ4	Pyruvate kinase n=4 Tax=Simiiformes RepID=E7EUJ4_HUMAN	70.53	4.37%	1	1	366	40.2	8.03
36	24.2	P14618	Pyruvate kinase isozymes M1/M2 n=16 Tax=Simiiformes RepID=	70.53	3.01%	1	1	531	57.9	7.84
37	24.3	B4DRT3	Pyruvate kinase n=2 Tax=Homo sapiens RepID=B4DRT3_HUMA	70.53	3.13%	1	1	511	55.9	7.5
38	24.4	B4DUU6	Pyruvate kinase n=4 Tax=Simiiformes RepID=B4DUU6_HUMAN	70.53	3.10%	1	1	516	56.2	8.44
39	24.5	E9PF79	Pyruvate kinase n=4 Tax=Simiiformes RepID=E9PF79_HUMAN	70.53	3.49%	1	1	458	50	7.64
40	24.6	P52480	Pyruvate kinase isozymes M1/M2 n=40 Tax=Tetrapoda RepID=K	70.53	3.01%	1	1	531	57.8	7.47
41	25.1	E7EU87	Uncharacterized protein n=3 Tax=Simiiformes RepID=E7EU87_H	1071.16	21.44%	13	38	555	58.9	5.97
42	25.2	P13647	Keratin, type II cytoskeletal 5 n=17 Tax=Eutheria RepID=K2C5_H	1071.16	20.17%	13	38	590	62.3	7.74
43	26	P04083	Annexin A1 n=14 Tax=Eutheria RepID=ANXA1_HUMAN	1646.95	45.66%	14	35	346	38.7	7.02
44	27	UPI00020CE287	UPI00020CE287 related cluster n=1 Tax=Homo sapiens RepID=U	715.14	47.76%	8	53	201	23.1	4.97
45	28	P14923	Junction plakoglobin n=31 Tax=Theria RepID=PLAK_HUMAN	158.88	7.79%	4	4	745	81.7	6.14
46	29	Q9UBG3	Cornulin n=6 Tax=Catarrhini RepID=CRNN_HUMAN	344.14	13.74%	4	5	495	53.5	6.1
47	30	P04080	Cystatin-B n=14 Tax=Simiiformes RepID=CYTB_HUMAN	326.21	45.92%	3	8	98	11.1	7.56
48	31.1	A4K467	Actinin alpha4 isoform n=3 Tax=Simiiformes RepID=A4K467_HU	131.07	5.37%	2	2	521	59.5	4.94
49	31.2	O43707	Alpha-actinin-4 n=40 Tax=Tetrapoda RepID=ACTN4_HUMAN	131.07	3.07%	2	2	911	104.8	5.44
50	31.3	D6PXK4	Alpha actinin 4 short isoform n=8 Tax=Eutheria RepID=D6PXK4_	131.07	4.05%	2	2	692	79.9	5.17
51	32	P27482	Calmodulin-like protein 3 n=5 Tax=Euarchontoglires RepID=CAL	83.59	11.41%	1	1	149	16.9	4.42
52	33	Q01469	Fatty acid-binding protein, epidermal n=10 Tax=Simiiformes Rep	63.04	11.85%	1	1	135	15.2	7.01
53	34.1	Q92817	Envoplakin n=5 Tax=Simiiformes RepID=EVPL_HUMAN	194.38	1.67%	2	4	2033	231.5	6.96
54	34.2	B7ZLH8	EVPL protein n=4 Tax=Simiiformes RepID=B7ZLH8_HUMAN	194.38	1.65%	2	4	2055	233.7	7.25
55	35.1	F5H651	Uncharacterized protein n=5 Tax=Catarrhini RepID=F5H651_HU	66.21	2.01%	1	1	646	72.8	9.26
56	35.2	O95171	Sciellin n=6 Tax=Catarrhini RepID=SCEL_HUMAN	66.21	1.89%	1	1	688	77.5	9.38
57	36	P18054	Arachidonate 12-lipoxygenase, 12S-type n=5 Tax=Eutheria RepI	130.93	4.22%	2	3	663	75.6	6.21
58	37.1	Q0QET7	Glyceraldehyde-3-phosphate dehydrogenase (Fragment) n=4 Ta	159.78	16.96%	2	4	230	24.6	8.51
59	37.2	E7EUT5	Glyceraldehyde-3-phosphate dehydrogenase n=3 Tax=Eutheria	159.78	15.00%	2	4	260	27.9	6.95
60	37.3	P04406	Glyceraldehyde-3-phosphate dehydrogenase n=42 Tax=Eutheria	159.78	11.64%	2	4	335	36	8.46
61	38.1	P01834	Ig kappa chain C region n=2 Tax=Homo sapiens RepID=IGKC_HU	378.25	57.55%	4	6	106	11.6	5.87
62	38.2	UPI000011049E	IGG CTM01 FAB (LIGHT CHAIN) n=2 Tax=Homo sapiens RepID=U	378.25	27.85%	4	6	219	24.1	7.74
63	38.3	Q0KKI6	Immunoblobulin light chain (Fragment) n=3 Tax=Homo sapiens	378.25	27.85%	4	6	219	24	8.06
64	38.4	Q5EFE6	Anti-RhD monoclonal T125 kappa light chain n=1 Tax=Homo sa	378.25	26.07%	4	6	234	25.7	8.43
65	38.5	Q6GMX0	Putative uncharacterized protein n=14 Tax=Euarchontoglires Re	378.25	25.85%	4	6	236	25.8	7.97
66	38.6	Q6P5S8	IGK@ protein n=6 Tax=Homo sapiens RepID=Q6P5S8_HUMAN	378.25	25.85%	4	6	236	25.8	6.33
67	38.7	Q7Z3Y4	Putative uncharacterized protein n=3 Tax=Homininae RepID=Q7	378.25	25.85%	4	6	236	25.7	7.59
68	38.8	UPI0000110495	ANTIBODY A5B7 (LIGHT CHAIN) n=1 Tax=Homo sapiens RepID=	378.25	28.64%	4	6	213	23.1	8.06
69	38.9	UPI0000110769	ANTIBODY n=1 Tax=Homo sapiens RepID=UPI0000110769	378.25	28.50%	4	6	214	23.6	6.33
70	38.10	UPI000014CDC4	OMALIZUMAB LIGHT CHAIN n=2 Tax=Homo sapiens RepID=UPI	378.25	27.98%	4	6	218	23.9	5.39
71	38.11	UPI00003CEBF2	HU3S193 Fab fragment, light chain n=3 Tax=Euarchontoglires R	378.25	27.85%	4	6	219	24	8.06
72	38.12	UPI0001A7C522	HUMAN IGG ABT-325 n=1 Tax=Homo sapiens RepID=UPI0001A7	378.25	28.50%	4	6	214	23.5	6.55

index	Prot_hit	Accession	Description	Mascot Score	Coverage	# Peptides	# PSMs	# AAs	MW [kDa]	calc. pI
73	38.13	UPI0001A7C54C	Fab Z13e1 n=3 Tax=Euarchontoglires RepID=UPI0001A7C54C	378.25	28.77%	4	6	212	23.1	7.71
74	38.14	UPI0001C591D8	Humanized recombinant Fab fragment of a murin n=5 Tax=Euar	378.25	26.64%	4	6	229	25.1	5.62
75	38.15	UPI0001D3410B	Immunoglobulin light chain n=1 Tax=Homo sapiens RepID=UPI0	378.25	27.98%	4	6	218	23.3	8.05
76	38.16	UPI0001F2FE82	Fab 2G12, light chain n=2 Tax=Homo sapiens RepID=UPI0001F2	378.25	28.64%	4	6	213	23.2	8.13
77	38.17	UPI0002003CF0	IMMUNOGLOBULIN A1 LIGHT CHAIN n=1 Tax=Homo sapiens Re	378.25	27.85%	4	6	219	24	6.77
78	38.18	UPI000211B3C2	B3 (IGKV4-1) light chain n=1 Tax=Homo sapiens RepID=UPI0002	378.25	27.73%	4	6	220	24.2	6.55
79	38.19	UPI0002177BCC	S4 FAB LIGHT CHAIN n=1 Tax=Homo sapiens RepID=UPI000217	378.25	28.91%	4	6	211	22.7	7.77
80	39.1	C7DJS1	Glutathione S-transferase pi (Fragment) n=3 Tax=Homo sapiens	190.28	13.25%	1	3	151	16.7	5.1
81	39.2	P09211	Glutathione S-transferase P n=4 Tax=Simiiformes RepID=GSTP1_	190.28	9.52%	1	3	210	23.3	5.64
82	40.1	E5RGE1	Uncharacterized protein n=6 Tax=Tetrapoda RepID=E5RGE1_HU	107.25	26.92%	1	1	52	5.9	4.78
83	40.2	E7ESK7	Uncharacterized protein n=7 Tax=Eutheria RepID=E7ESK7_HUM	107.25	10.22%	1	1	137	15.7	5.27
84	40.3	P63104	14-3-3 protein zeta/delta n=26 Tax=Amniota RepID=1433Z_HU	107.25	5.71%	1	1	245	27.7	4.79
85	41.1	B4DWQ3	Phosphoglycerate kinase n=7 Tax=Theria RepID=B4DWQ3_HUM	74.97	10.56%	1	1	161	17	5.6
86	41.2	B4E1H9	Phosphoglycerate kinase n=6 Tax=Amniota RepID=B4E1H9_HU	74.97	5.20%	1	1	327	35	8.32
87	41.3	P00558	Phosphoglycerate kinase 1 n=36 Tax=Theria RepID=PGK1_HUM	74.97	4.08%	1	1	417	44.6	8.1
88	42	P07737	Profilin-1 n=21 Tax=Theria RepID=PROF1_HUMAN	88.6	12.86%	1	1	140	15	8.27
89	43.1	A1L195	TUBB2B protein (Fragment) n=16 Tax=Fungi/Metazoa group Re	168.82	23.94%	2	3	188	20.6	5.05
90	43.2	Q13885	Tubulin beta-2A chain n=339 RepID=TBB2A_HUMAN	168.82	10.11%	2	3	445	49.9	4.89
91	43.3	Q5ST81	Tubulin beta polypeptide n=8 Tax=Eukaryota RepID=Q5ST81_HU	168.82	12.10%	2	3	372	41.7	4.91
92	43.4	E9PBJ4	Uncharacterized protein n=2 Tax=Homo sapiens RepID=E9PBJ4_	168.82	18.52%	2	3	243	27.4	4.49
93	44.1	Q05CF8	KNG1 protein n=1 Tax=Homo sapiens RepID=Q05CF8_HUMAN	83.94	6.53%	1	1	291	33	6.67
94	44.2	C9JEX1	Uncharacterized protein n=3 Tax=Hominoidea RepID=C9JEX1_H	83.94	4.86%	1	1	391	43.8	6.43
95	44.3	P01042	Kininogen-1 n=11 Tax=Catarrhini RepID=KNG1_HUMAN	83.94	2.95%	1	1	644	71.9	6.81
96	45.1	C9K0S6	Uncharacterized protein n=2 Tax=Simiiformes RepID=C9K0S6_H	82.13	44.68%	1	1	47	5.3	5.21
97	45.2	C9JEV8	Uncharacterized protein n=10 Tax=Coelomata RepID=C9JEV8_H	82.13	13.73%	1	1	153	16.9	5.53
98	45.3	Q13748	Tubulin alpha-3C/D chain n=18 Tax=Theria RepID=TBA3C_HUM	82.13	4.67%	1	1	450	49.9	5.1
99	45.4	Q71U36	Tubulin alpha-1A chain n=597 RepID=TBA1A_HUMAN	82.13	4.66%	1	1	451	50.1	5.06
100	45.5	A8MUB1	Tubulin, alpha 1 (Testis specific), isoform CRA_a n=3 Tax=Simiif	82.13	4.85%	1	1	433	48.3	5.01
101	45.6	B3KPW9	Tubulin, alpha 8, isoform CRA_b n=17 Tax=Amniota RepID=B3K	82.13	5.48%	1	1	383	42.9	5.08
102	45.7	F5H5D3	Uncharacterized protein n=5 Tax=Simiiformes RepID=F5H5D3_	82.13	4.05%	1	1	519	57.7	5.07
103	45.8	E9PGK3	Uncharacterized protein n=4 Tax=Catarrhini RepID=E9PGK3_HU	82.13	6.44%	1	1	326	36.7	8.44
104	45.9	Q8WU19	TUBA1B protein n=2 Tax=Homininae RepID=Q8WU19_HUMAN	82.13	6.27%	1	1	335	37.2	4.98
105	45.10	C9JJQ8	Uncharacterized protein n=1 Tax=Homo sapiens RepID=C9JJQ8_	82.13	10.61%	1	1	198	21.7	6.28
106	45.11	Q29196	Tubulin alpha-1 chain (Fragment) n=15 Tax=Eukaryota RepID=Q	82.13	17.65%	1	1	119	13.1	5.2
107	45.12	Q6QMJ5	Tubulin alpha-1 (Fragment) n=1 Tax=Homo sapiens RepID=Q6Q	82.13	21.43%	1	1	98	10.8	7.52

2DLCSCI_1 Peptide Summary

Sequence	Protein Descriptions	# Proteins	# Protein Groups	Protein Group Accessions	Modifications	q-Value	PEP	Charge	m/z [Da]	MH+ [Da]	Delta Mass [Da]
FSVQMFR	Uncharacterized protein n=3 Tax=Simiiformes Repl	2	1	E9PFJ3		0.00223	0.03	2	457.99939	914.9915	0.27
AGLQFPVGR	Histone H2A n=1 Tax=Homo sapiens RepID=C9J0D1	10	1	C9J0D1		0	0.000153	2	472.93671	944.86614	0.17
IEISELNR	Keratin, type II cytoskeletal 1 n=7 Tax=Eutheria Rep	2	2	P04264;P35908		0	0.00124	2	487.45093	973.89458	0.18
AGFAGDDAPR	Uncharacterized protein n=11 Tax=Simiiformes Rep	4	1	F5H0N0		0	3.36E-05	2	489.19128	977.37529	0.46
RVPFSLLR	Heat shock protein beta-1 n=6 Tax=Simiiformes Rep	1	1	P04792		0.00118	0.0177	2	494.59576	988.18425	0.29
LAEPSQLLK	cDNA FLJ60424, highly similar to Junction plakoglo	2	1	P14923		0.00223	0.0421	2	500.41028	999.81328	0.61
YENELALR	Keratin, type I cytoskeletal 13 n=13 Tax=Simiiforme	1	1	P13646		0	0.00132	2	504.58755	1008.16783	0.33
LLEGEEYR	cDNA FLJ55805, highly similar to Keratin, type II cyt	3	2	B4DRW1;P12035		0	0.0024	2	505.0383	1009.06932	0.29
SGFSSVSVSR	Keratin, type II cytoskeletal 6A n=25 Tax=Euarchont	1	1	P02538		0	0.0002	2	507.17773	1013.34819	0.42
DLTDYLmK	Uncharacterized protein n=11 Tax=Simiiformes Rep	4	1	F5H0N0	M7(Oxidation)	0.00118	0.0039	1	1014.6084	1014.6084	0.13
QLDSIVGER	Keratin, type II cytoskeletal 6A n=25 Tax=Euarchont	4	2	P02538;E7EU87		0	0.000357	2	509.02005	1017.03282	0.25
VILEIDNAR	Keratin, type I cytoskeletal 13 n=13 Tax=Simiiforme	1	1	P13646		0.00118	0.00478	1	1042.68494	1042.68494	0.1
LASYLDKVR	Keratin, type I cytoskeletal 10 n=12 Tax=Eutheria R	3	2	P13645;P02533		0	0.00243	2	533.01794	1065.02861	0.21
AQYEDIAQK	Keratin, type II cytoskeletal 1 n=7 Tax=Eutheria Rep	1	1	P04264		0	0.0019	2	533.50323	1065.99919	0.24
FASFIDKVR	Keratin, type II cytoskeletal 2 epidermal n=7 Tax=Si	8	4	P35908;P12035;P02538;E7EU87		0.00118	0.00267	2	542.23669	1083.46611	0.43
LTMQNLNDR	Keratin, type I cytoskeletal 19 n=9 Tax=Eutheria Rep	2	1	P13646		0	8.56E-05	2	552.94165	1104.87602	0.16
AQYEEIAQR	cDNA FLJ55805, highly similar to Keratin, type II cyt	5	3	B4DRW1;P35908;P02538		0	0.000765	2	554.42322	1107.83916	0.15
DAEAWFNEK	Keratin, type I cytoskeletal 10 n=12 Tax=Eutheria R	1	1	P13645		0.00118	0.00592	1	1109.57544	1109.57544	0.09
LEQEIATYR	Keratin, type I cytoskeletal 14 n=8 Tax=Eutheria Rep	4	2	P02533;P13646		0	0.000451	2	562.2356	1123.46391	0.44
EQYEAMAER	Keratin, type I cytoskeletal 13 n=13 Tax=Simiiforme	1	1	P13646		0	0.000193	2	564.36853	1127.72978	0.62
DWVSVVTPAR	Uncharacterized protein n=3 Tax=Simiiformes Repl	2	1	E9PFJ3		0	0.00113	1	1129.73828	1129.73828	0.14
DAEEWFHAK	GUCA1B protein (Fragment) n=1 Tax=Homo sapien	1	1	A4FUA1		0	0.000966	2	567.0777	1133.14812	0.32
GYSFTTTAER	Uncharacterized protein n=11 Tax=Simiiformes Rep	4	1	F5H0N0		0	0.000417	2	567.01886	1133.03044	0.25
KLLEGEEYR	cDNA FLJ55805, highly similar to Keratin, type II cyt	3	2	B4DRW1;P12035		0	0.00227	2	569.22662	1137.44597	0.43
ILTATIENNR	Keratin, type I cytoskeletal 13 n=13 Tax=Simiiforme	1	1	P13646		0	3.88E-05	2	572.96973	1144.93218	0.15
EITALAPSTMK	Uncharacterized protein n=11 Tax=Simiiformes Rep	4	1	F5H0N0		0	0.000229	1	1161.65247	1161.65247	0.03
LFDQAFGLPR	Heat shock protein beta-1 n=6 Tax=Simiiformes Rep	1	1	P04792		0	0.000175	2	582.74091	1164.47453	0.43
YEELQVTAGR	Keratin, type II cytoskeletal 6A n=25 Tax=Euarchont	2	1	P02538		0	5.40E-05	2	583.52936	1166.05144	0.23
YEELQITAGR	Keratin, type II cytoskeletal 1 n=7 Tax=Eutheria Rep	1	1	P04264		0	1.93E-05	2	590.51373	1180.02019	0.21
ISGLIYEETR	Histone H4 n=219 Tax=Eukaryota RepID=H4_HUMA	1	1	P62805		0	0.00127	2	591.35321	1181.69914	0.54
AVFPSIVGRPR	Uncharacterized protein n=11 Tax=Simiiformes Rep	4	1	F5H0N0		0.00118	0.00413	2	600.09583	1199.18437	0.24
QSVEADINGLR	Keratin, type I cytoskeletal 10 n=12 Tax=Eutheria R	2	2	P13645;P13646		0	1.13E-05	2	601.43927	1201.87126	0.13
WTLLQEQGTK	Keratin, type II cytoskeletal 6A n=25 Tax=Euarchont	4	2	P02538;E7EU87		0	5.09E-05	2	602.82251	1204.63774	0.5
AEAESWYQTK	Keratin, type II cytoskeletal 6A n=25 Tax=Euarchont	2	1	P02538		0	0.00214	2	607.40295	1213.79863	0.62
LKYENELALR	Keratin, type I cytoskeletal 13 n=13 Tax=Simiiforme	1	1	P13646		0	0.000485	2	625.22961	1249.45195	0.38
VSNKGLPAPIEK	Putative uncharacterized protein DKFZp686C15213	2	1	Q6MZU6		0.00118	0.0032	2	626.97766	1252.94805	0.11
TPAQFDADELR	Annexin A1 n=14 Tax=Eutheria RepID=ANXA1_HUM	1	1	P04083		0	7.70E-06	2	632.23682	1263.46636	0.43
GAFSSVSMSGGAGR	cDNA FLJ55805, highly similar to Keratin, type II cyt	2	1	B4DRW1		0	4.26E-05	2	636.23779	1271.46831	0.44
ALNSIIDVYHK	Protein S100-A8 n=5 Tax=Hominoidea RepID=S10A	1	1	P05109		0	0.00253	2	636.85107	1272.69487	0
NTKSEIAELNR	cDNA FLJ55805, highly similar to Keratin, type II cyt	2	1	B4DRW1		0	0.00191	2	638.19641	1275.38555	0.36
LALDLEIATYR	Keratin, type II cytoskeletal 1 n=7 Tax=Eutheria Rep	3	2	P04264;B4DRW1		0	6.23E-05	2	640.05005	1279.09282	0.69
VVLQQDPQQAR	Periplakin n=3 Tax=Hominoidea RepID=PEPL_HUM	1	1	O60437		0	0.000231	2	641.76794	1282.52861	0.42
GAFSSVSmSGGAGR	cDNA FLJ55805, highly similar to Keratin, type II cyt	2	1	B4DRW1	M8(Oxidation)	0	3.46E-06	2	644.18506	1287.36284	0.39
NHEEEMKDLR	Keratin, type I cytoskeletal 10 n=12 Tax=Eutheria R	1	1	P13645		0.00118	0.0109	2	650.98785	1300.96843	0.19
ALEEANADLEVK	Keratin, type I cytoskeletal 14 n=8 Tax=Eutheria Rep	2	2	P02533;P13646		0	0.000363	1	1301.73645	1301.73645	0.08
SLDLDSIIAEVK	Keratin, type II cytoskeletal 1 n=7 Tax=Eutheria Rep	1	1	P04264		0	1.86E-07	2	652.12775	1303.24822	0.27
NSKIEISELNR	Keratin, type II cytoskeletal 1 n=7 Tax=Eutheria Rep	1	1	P04264		0	0.00079	2	652.09021	1303.17314	0.24
NTKQEIAEINR	Keratin, type II cytoskeletal 6A n=25 Tax=Euarchont	1	1	P02538		0	0.000385	2	658.79553	1316.58379	0.44
HGGGGGGFGGGGFGSR	Keratin, type II cytoskeletal 2 epidermal n=7 Tax=Si	1	1	P35908		0	6.33E-11	2	660.8858	1320.76433	0.09
TDTDGKFLYHK	Uncharacterized protein n=1 Tax=Homo sapiens Re	2	1	B7Z8R6		0	0.00051	2	663.24573	1325.48418	0.42
SQVVAGTNYFIK	Cystatin-B n=14 Tax=Simiiformes RepID=CYTB_HUM	1	1	P04080		0	0.000659	1	1326.55713	1326.55713	-0.15
NLDLDSIIAEVK	Keratin, type II cytoskeletal 2 epidermal n=7 Tax=Si	6	4	P35908;P02538;E7EU87;UPI00020CE287		0	2.84E-05	2	665.74579	1330.4843	0.38
TAAENDFVVLKK	cDNA FLJ55805, highly similar to Keratin, type II cyt	2	1	B4DRW1		0	0.000212	2	668.27307	1335.53887	0.4
STEGGQSLDSLIK	Uncharacterized protein n=5 Tax=Catarrhini RepID=	2	1	F5H651		0	3.17E-06	2	668.35132	1335.69536	0.51
SKAEAEALYQTK	cDNA FLJ55805, highly similar to Keratin, type II cyt	5	2	B4DRW1;P12035		0	0.000176	1	1338.69336	1338.69336	0
SKAEAESLYQSK	Keratin, type II cytoskeletal 1 n=7 Tax=Eutheria Rep	1	1	P04264		0	0.000166	2	671.01904	1341.03081	0.18
LPGDNALDMFQK	Arachidonate 12-lipoxygenase, 12S-type n=5 Tax=E	1	1	P18054		0	2.69E-05	2	675.36304	1349.7188	0.53
TAAENEFVTLKK	Keratin, type II cytoskeletal 3 n=7 Tax=Catarrhini R	3	2	P12035;P02538		0	5.17E-05	2	676.00146	1350.99565	0.13
RVEPYGENFNK	APOA4 protein (Fragment) n=1 Tax=Homo sapiens	2	1	Q13784		0	0.0018	2	677.30682	1353.60637	0.47
RVELEAALQQAK	cDNA FLJ55805, highly similar to Keratin, type II cyt	2	1	B4DRW1		0	2.24E-05	2	678.80377	1356.60027	0.42
NLDLDSIIAEVR	cDNA FLJ55805, highly similar to Keratin, type II cyt	3	1	B4DRW1		0	9.02E-08	2	679.57916	1358.15105	0.21
NKLEGLEDALQK	Keratin, type II cytoskeletal 6A n=25 Tax=Euarchont	2	1	P02538		0	2.99E-05	1	1357.80688	1357.80688	0.07
NTKHEISEMNR	Uncharacterized protein n=3 Tax=Simiiformes Repl	3	2	E7EU87;UPI00020CE287		0	0.000498	2	680.23083	1359.45439	0.4
SAYQEAMDISKK	14-3-3 protein sigma n=20 Tax=Eutheria RepID=143	1	1	P31947		0	0.000959	2	686.35681	1371.70635	0.52
ATLENDFVVLKK	Keratin, type II cytoskeletal 78 n=7 Tax=Hominoide	1	1	Q8N1N4		0	0.000801	2	689.36292	1377.71855	0.47
TRLEQEIATYR	Keratin, type I cytoskeletal 14 n=8 Tax=Eutheria Rep	2	2	P02533;P13646		0	0.00178	2	690.625	1380.24272	0.26
VSFELFADKVPK	Peptidyl-prolyl cis-trans isomerase A n=98 Tax=The	1	1	P62937		0	3.81E-05	2	690.58301	1380.15874	0.2
SLNNQFASFIDK	Keratin, type II cytoskeletal 1 n=7 Tax=Eutheria Rep	1	1	P04264		0	3.56E-05	2	692.82166	1384.63603	0.47
VGWEQLLTTIAR	Actinin alpha4 isoform n=3 Tax=Simiiformes RepID=	3	1	A4K467		0	3.79E-05	2	694.18457	1387.36186	0.29
GVDEATIIDILTK	Annexin A1 n=14 Tax=Eutheria RepID=ANXA1_HUM	1	1	P04083		0	2.60E-05	2	694.60083	1388.19438	0.21
MIGFPSSAGSVSPR	Keratin, type I cytoskeletal 13 n=13 Tax=Simiiforme	1	1	P13646		0	4.73E-07	2	697.28247	1393.55766	0.43
TNAENEFVTIKK	Keratin, type II cytoskeletal 1 n=7 Tax=Eutheria Rep	1	1	P04264		0	0.00112	2	697.58746	1394.16765	0.22
TLNNKFASFIDK	Keratin, type II cytoskeletal 2 epidermal n=7 Tax=Si	6	4	P35908;P12035;P02538;E7EU87		0	0.000289	2	699.84021	1398.67314	0.47
TLNNQFASFIDK	Keratin, type II cytoskeletal 78 n=7 Tax=Hominoide	1	1	Q8N1N4		0	9.53E-05	2	699.84021	1398.67314	0.48
LALDIEIATYRK	cDNA FLJ55805, highly similar to Keratin, type II cyt	2	1	B4DRW1		0	0.000977	2	703.63367	1406.26006	0.23
VDSLNDEINFLK	cDNA FLJ55805, highly similar to Keratin, type II cyt	2	1	B4DRW1		0	1.11E-05	2	704.24597	1407.48467	0.38
GNPTVEVDLFTSK	Alpha-enolase n=53 Tax=Euteleostomi RepID=ENOA	1	1	P06733		0	0.00115	2	704.21143	1407.41557	0.35
ADTLTDEINFLR	Keratin, type II cytoskeletal 6A n=25 Tax=Euarchont	2	1	P02538		0	0.000432	2	704.47192	1407.93657	0.11
mIGFPSSAGSVSPR	Keratin, type I cytoskeletal 13 n=13 Tax=Simiiforme	1	1	P13646	M1(Oxidation)	0	0.00025	1	1408.59949	1408.59949	-0.09
TTAENEFVMLKK	Uncharacterized protein n=3 Tax=Simiiformes Repl	2	1	E7EU87		0	0.00243	2	705.91736	1410.82744	0.05
GALQNIIPASTGAAK	Glyceraldehyde-3-phosphate dehydrogenase (Frag	3	1	Q0QET7		0	0.00226	2	706.48743	1411.96758	0.09
TALQPMVSALNIR	Uncharacterized protein n=3 Tax=Simiiformes Repl	2	1	E9PFJ3		0	0.000108	2	707.49054	1413.9738	0.09
ELDVEVSRLEAR	Periplakin n=3 Tax=Hominoidea RepID=PEPL_HUM	1	1	O60437		0.0043	0.0898	2	708.56494	1416.12261	0.19
VHVGDEDFVHLR	Cystatin-B n=14 Tax=Simiiformes RepID=CYTB_HUM	1	1	P04080		0	1.14E-06	2	712.24292	1423.47856	0.38
xDALMDEINFMK	UPI00020CE287 related cluster n=1 Tax=Homo sap	1	1	UPI00020CE287	N-Term(Acetyl); X1(G)	0	5.80E-06	2	713.77802	1426.54875	0.46
VDALMDEINFMK	Uncharacterized protein n=3 Tax=Simiiformes Repl	3	2	E7EU87;UPI00020CE287		0	7.00E-06	2	713.77802	1426.54875	0.44
TTAENEFVmLKK	Uncharacterized protein n=3 Tax=Simiiformes Repl	2	1	E7EU87	M9(Oxidation)	0	0.000381	2	714.27728	1427.54729	0.41
TMQDSVEDFKTK	cDNA FLJ55805, highly similar to Keratin, type II cyt	2	1	B4DRW1		0	4.99E-05	2	715.35144	1429.6956	0.51
TALQPmVSALNIR	Uncharacterized protein n=3 Tax=Simiiformes Repl	2	1	E9PFJ3	M6(Oxidation)	0	8.53E-05	2	715.94214	1430.877	0.55
IRLENEIQTYR	Keratin, type I cytoskeletal 10 n=12 Tax=Eutheria R	1	1	P13645		0	0.000343	2	717.99011	1434.97295	0.1
ILNEMRDQYEK	Keratin, type I cytoskeletal 14 n=8 Tax=Eutheria Rep	1	1	P02533		0	0.00107	2	720.31628	1439.62529	0.46
VGEAWQDRPGAPR	UPI000059DBAE related cluster n=1 Tax=Homo sap	7	1	UPI000059DBAE		0.00118	0.00737	2	720.28601	1439.56474	0.42
SRAEAESWYQTK	Keratin, type II cytoskeletal 6A n=25 Tax=Euarchont	2	1	P02538		0.0043	0.0839	2	728.81628	1456.62529	0.47
LGHPDTLNQGEFK	Protein S100-A9 n=5 Tax=Hominoidea RepID=S10A	1	1	P06702		0	3.36E-05	2	728.51379	1456.02031	0.15
RQLLEGELETLR	Periplakin n=3 Tax=Hominoidea RepID=PEPL_HUM	1	1	O60437		0	0.000619	2	729.37305	1457.73882	0.46
WELLQQVDTSTR	Keratin, type II cytoskeletal 1 n=7 Tax=Eutheria Rep	1	1	P04264		0	1.52E-07	2	738.81348	1476.61968	0.44
FLEQQNQVLQTK	Keratin, type II cytoskeletal 1 n=7 Tax=Eutheria Rep	2	2	P04264;P35908		0	3.48E-05	2	738.81537	1476.62346	0.42
FLEQQNKVLETK	Keratin, type II cytoskeletal 3 n=7 Tax=Catarrhini R	3	3	P12035;P02538;Q8N1N4		0	4.44E-05	2	739.11719	1477.2271	0.21
FQNSLKELQLQK	Desmoplakin n=28 Tax=Mammalia RepID=DESP_HU	1	1	P15924		0.00118	0.00363	2	738.82153	1476.63579	0.41
TVTAmDVVYALKR	Histone H4 n=219 Tax=Eukaryota RepID=H4_HUMA	1	1	P62805	M5(Oxidation)	0.00118	0.00564	2	742.54529	1484.0833	0.64
NEGTATYAAAVLFR	Junction plakoglobin n=31 Tax=Theria RepID=PLAK_	1	1	P14923		0	7.59E-05	2	742.85645	1484.70561	0.48
SRTEAESWYQTK	Uncharacterized protein n=3 Tax=Simiiformes Repl	3	2	E7EU87;UPI00020CE287		0	0.00182	2	743.81342	1486.61955	0.46
SQYEQLAEQNRK	Keratin, type I cytoskeletal 10 n=12 Tax=Eutheria R	1	1	P13645		0.00118	0.0192	2	747.57666	1494.14604	0.21
SLLGEVEQNLQAAK	Periplakin n=3 Tax=Hominoidea RepID=PEPL_HUM	1	1	O60437		0	6.32E-05	2	750.86371	1500.72014	0.46
VKEGMNIVEAMER	Peptidyl-prolyl cis-trans isomerase A n=98 Tax=The	1	1	P62937		0	5.29E-05	2	753.77844	1506.54961	0.4
LLKEYQELMNVK	Keratin, type II cytoskeletal 6A n=25 Tax=Euarchont	2	1	P02538		0	0.000387	2	754.84839	1508.6895	0.44
VLKQVHPDTGISSK	Histone H2B type 1-J n=102 Tax=Euteleostomi Repl	5	1	P06899		0	8.64E-05	2	755.42505	1509.84282	0.5
AITGILRPPLEQGR	Periplakin n=3 Tax=Hominoidea RepID=PEPL_HUM	1	1	O60437		0	0.00188	2	761.50037	1521.99346	0.55
LLRDYQELMNTK	Keratin, type II cytoskeletal 1 n=7 Tax=Eutheria Rep	1	1	P04264		0	0.000408	2	762.78796	1524.56865	0.39
SPNQNVQQAAAGALR	Plakophilin-1 n=15 Tax=Eutheria RepID=PKP1_MOU	2	1	P97350		0	0.00079	2	762.9707	1524.93413	0.07
MLREYQELMSVK	cDNA FLJ55805, highly similar to Keratin, type II cyt	2	1	B4DRW1		0.00118	0.00637	2	763.49506	1525.98284	-0.39
LLREYQELMNTK	Uncharacterized protein n=3 Tax=Simiiformes Repl	3	2	E7EU87;UPI00020CE287		0	0.00261	2	769.74768	1538.48808	0.34
LLRDYQELmNTK	Keratin, type II cytoskeletal 1 n=7 Tax=Eutheria Rep	1	1	P04264	M9(Oxidation)	0	0.000123	2	770.77893	1540.55058	0.38
mLREYQELMSVK	cDNA FLJ55805, highly similar to Keratin, type II cyt	2	1	B4DRW1	M1(Oxidation)	0	0.0025	2	772.21143	1543.41557	0.33
GVDEATIIDILTKR	Annexin A1 n=14 Tax=Eutheria RepID=ANXA1_HUM	1	1	P04083		0	9.02E-07	2	772.1275	1543.24773	-0.31
MQKEITALAPSTMK	Uncharacterized protein n=11 Tax=Simiiformes Rep	4	1	F5H0N0		0	7.69E-06	2	775.27252	1549.53777	0.36
SVTEQGAELSNEER	Uncharacterized protein n=6 Tax=Tetrapoda RepID	3	1	E5RGE1		0	8.05E-07	2	775.2467	1549.48613	0.39
GTDVNVFNTILTTR	Annexin A1 n=14 Tax=Eutheria RepID=ANXA1_HUM	1	1	P04083		0	3.49E-06	2	776.48431	1551.96135	0.57
NKLEGLEDALQKAK	Keratin, type II cytoskeletal 6A n=25 Tax=Euarchont	2	1	P02538		0.00329	0.0754	2	779.36609	1557.7249	0.43
YEEIVKEVSTYIK	Eukaryotic translation elongation factor 1 alpha (Fr	3	1	A9X7H1		0	0.000216	2	800.85681	1600.70635	-0.07
NKLNDLEDALQQAK	Keratin, type II cytoskeletal 1 n=7 Tax=Eutheria Rep	1	1	P04264		0	8.28E-07	2	800.92261	1600.83794	0.5
ALTGHLEEVVLALLK	Annexin A1 n=14 Tax=Eutheria RepID=ANXA1_HUM	1	1	P04083		0	6.92E-06	2	803.08032	1605.15337	-0.4
VISGVLQLGNIVFKK	Myosin-9 n=67 Tax=Tetrapoda RepID=MYH9_HUM	1	1	P35579		0	0.00162	2	808.51813	1616.02898	0.52
LDLAGRDLTDYLMK	Uncharacterized protein n=11 Tax=Simiiformes Rep	4	1	F5H0N0		0	0.000823	2	812.97534	1624.94341	0.55
LAGTQPLEVLEAVQR	Ubiquitin-like modifier activating enzyme 1 n=2 Tax	2	1	Q5JRR6		0.00118	0.0105	2	812.97888	1624.95049	0.52
WNLLQQQTTTTSSK	cDNA FLJ55805, highly similar to Keratin, type II cyt	2	1	B4DRW1		0	4.24E-07	2	818.83606	1636.66484	0.42
SLNNQFASFIDKVR	Keratin, type II cytoskeletal 1 n=7 Tax=Eutheria Rep	1	1	P04264		0	4.04E-06	2	820.30444	1639.60161	0.37

Sequence	Protein Descriptions	# Proteins	# Protein Groups	Protein Group Accessions	Modifications	q-Value	PEP	Charge	m/z [Da]	MH+ [Da]	Delta Mass [Da]
LDLAGRDLTDYLmK	Uncharacterized protein n=11 Tax=Simiiformes Rep	4	1	F5H0N0	M13(Oxidation)	0.00118	0.00502	2	820.88739	1640.7675	0.47
DLAKDITSDTSGDFR	Annexin A1 n=14 Tax=Eutheria RepID=ANXA1_HUM	1	1	P04083		0	5.95E-06	2	821.03638	1641.06548	0.14
AQLGGPEAAKSDETAAK	Heat shock protein beta-1 n=6 Tax=Simiiformes Rep	1	1	P04792		0	3.25E-07	2	822.82507	1644.64287	0.41
TLNNKFASFIDKVR	Keratin, type II cytoskeletal 2 epidermal n=7 Tax=Si	6	4	P35908;P12035;P02538;E7EU87		0	0.0025	2	827.26434	1653.52141	0.3
TLNNQFASFIDKVR	Keratin, type II cytoskeletal 78 n=7 Tax=Hominoide	1	1	Q8N1N4		0	0.000987	2	827.26434	1653.52141	0.32
SGGGFSSGSAGIINYQR	Keratin, type II cytoskeletal 1 n=7 Tax=Eutheria Rep	1	1	P04264		0	9.74E-07	2	829.80194	1658.59661	0.4
TPAQFDADELRAAMK	Annexin A1 n=14 Tax=Eutheria RepID=ANXA1_HUM	1	1	P04083		0	1.68E-07	2	832.85858	1664.70989	0.45
DVDAAYLNKVELEAK	cDNA FLJ55805, highly similar to Keratin, type II cyt	2	1	B4DRW1		0	0.00013	2	839.80231	1678.59734	0.36
KGTDVNVFNTILTTR	Annexin A1 n=14 Tax=Eutheria RepID=ANXA1_HUM	1	1	P04083		0	1.27E-07	2	840.47693	1679.94658	0.52
NLEPLFETYLSVLR	cDNA FLJ55805, highly similar to Keratin, type II cyt	2	1	B4DRW1		0	0.000262	2	847.90814	1694.80901	0.45
DVDAAYMNKVELQAK	Keratin, type II cytoskeletal 6A n=25 Tax=Euarchont	2	1	P02538		0	1.94E-05	2	847.91754	1694.82781	-0.01
IRIDSLSAQLSQLQK	Rhabdomyosarcoma antigen MU-RMS-40.12 n=10	3	1	Q3BDU5		0	1.79E-06	2	850.94324	1700.8792	0.45
GLGTDEDTLIEILASR	Annexin A1 n=14 Tax=Eutheria RepID=ANXA1_HUM	1	1	P04083		0	2.37E-07	2	852.38452	1703.76177	0.44
DSTIQVVENGESSQGR	Uncharacterized protein n=3 Tax=Simiiformes RepI	2	1	E9PFJ3		0	1.27E-06	2	853.88794	1706.7686	0.49
GSLGGGFSSGGFSGGSFSR	Keratin, type I cytoskeletal 10 n=12 Tax=Eutheria R	1	1	P13645		0	4.90E-09	2	854.80078	1708.59429	0.41
QISNLQQSISDAEQR	Keratin, type II cytoskeletal 1 n=7 Tax=Eutheria Rep	1	1	P04264		0	3.39E-09	2	859.24213	1717.47698	0.31
QLLQANPILEAFGNAK	Myosin-9 n=67 Tax=Tetrapoda RepID=MYH9_HUM	1	1	P35579		0	2.07E-05	2	864.45483	1727.90239	0.48
GIGTNEQAIIDVLTKR	cDNA FLJ58992, moderately similar to Annexin A8	3	1	B4DLF6		0	0.000634	2	864.86755	1728.72783	0.38
SEDFGVNEDLADSDAR	Annexin A1 n=14 Tax=Eutheria RepID=ANXA1_HUM	1	1	P04083		0	5.95E-07	2	870.71045	1740.41362	0.34
VLDTKWTLLQEQGTK	Uncharacterized protein n=3 Tax=Simiiformes RepI	3	1	E7EU87		0	1.77E-06	2	880.3446	1759.68193	-0.14
QTFTPPPQLQQQQVK	Small proline-rich protein 3 n=4 Tax=Homininae Re	1	1	Q9UBC9		0	0.000636	2	884.86462	1768.72197	0.39
ALESPERPFLAILGGAK	Phosphoglycerate kinase n=7 Tax=Theria RepID=B4	3	1	B4DWQ3		0	3.77E-06	2	885.41724	1769.8272	0.42
WVQGEDILSLPEGTAR	Arachidonate 12-lipoxygenase, 12S-type n=5 Tax=E	1	1	P18054		0	7.13E-05	2	886.59692	1772.18657	0.64
QFASQANVVGPWIQTK	Actinin alpha4 isoform n=3 Tax=Simiiformes RepID	3	1	A4K467		0	1.99E-06	2	887.96033	1774.91338	0.49
EYGGLDVLVNNAGIAFK	Carbonyl reductase 1, isoform CRA_c n=2 Tax=Simi	4	1	B4DFK7		0	0.000215	2	891.09753	1781.18779	0.63
VSLDVNHFAPDELTVK	Heat shock protein beta-1 n=6 Tax=Simiiformes Rep	1	1	P04792		0	5.56E-06	2	892.84644	1784.68559	0.38
ISPQIQLSGQTEQTQK	Cornulin n=6 Tax=Catarrhini RepID=CRNN_HUMAN	1	1	Q9UBG3		0	1.40E-09	2	893.54578	1786.08428	0.08
QSPASPERDYSPYYK	Keratin, type I cytoskeletal 13 n=13 Tax=Simiiforme	1	1	P13646		0	0.00106	2	894.875	1788.74272	0.46
VELEAALQQAKEELAR	cDNA FLJ55805, highly similar to Keratin, type II cyt	2	1	B4DRW1		0	4.69E-08	2	899.55688	1798.10649	0.07
GFDEYMKELGVGIALR	Fatty acid-binding protein, epidermal n=10 Tax=Sin	1	1	Q01469		0	5.94E-05	2	900.08484	1799.1624	0.62
AGVEEQEGLLSFQEDR	Envoplakin n=5 Tax=Simiiformes RepID=EVPL_HUM	2	1	Q92817		0	4.56E-07	2	904.2345	1807.46172	0.31
NIETIINTFHQYSVK	Protein S100-A9 n=5 Tax=Hominoidea RepID=S10A	1	1	P06702		0	0.000412	2	904.50269	1807.99809	0.53
TLQGLEIELQSQLSMK	Keratin, type I cytoskeletal 19 n=9 Tax=Eutheria Re	4	2	A4FUA1;P13646		0	4.77E-08	2	909.61804	1818.22881	0.13
NLEPLFETYLSVLRK	cDNA FLJ55805, highly similar to Keratin, type II cyt	2	1	B4DRW1		0	2.18E-06	2	911.97009	1822.93291	0.46
VQQLQISVDQHGDNLK	cDNA FLJ55805, highly similar to Keratin, type II cyt	2	1	B4DRW1		0	1.46E-07	2	911.8075	1822.60771	0.33
RFDEILEASDGIMVAR	Pyruvate kinase n=4 Tax=Simiiformes RepID=E7EUJ	6	1	E7EUJ4		0	1.22E-05	2	911.82568	1822.64409	0.36
VLAEMREQYEAMAER	Keratin, type I cytoskeletal 13 n=13 Tax=Simiiforme	1	1	P13646		0	0.00136	2	913.39612	1825.78496	-0.04
NLSDVATKQEGLESVLK	Junction plakoglobin n=31 Tax=Theria RepID=PLAK	1	1	P14923		0	0.000834	2	916.42615	1831.84502	0.43
SIYGEKFEDENFILK	Peptidyl-prolyl cis-trans isomerase A n=98 Tax=The	1	1	P62937		0	3.94E-05	2	916.8291	1832.65093	0.37
TLQGLEIELQSQLSmK	Keratin, type I cytoskeletal 19 n=9 Tax=Eutheria Re	4	2	A4FUA1;P13646	M15(Oxidation)	0	2.78E-07	2	917.86438	1834.72148	0.38
QEPSQGTTTFAVTSILR	Ig alpha-2 chain C region n=7 Tax=Hominidae RepID	10	1	P01877		0	0.000988	2	918.83813	1836.66899	0.36
SAIYQLEEEYENLLK	Desmoplakin n=28 Tax=Mammalia RepID=DESP_HU	1	1	P15924		0	1.73E-05	2	921.74829	1842.48931	0.29
TLNDMRQEYEQLIAK	Keratin, type I cytoskeletal 9 n=4 Tax=Catarrhini Re	1	1	P35527		0	0.000281	3	618.37268	1853.10349	0.39
SISNKELSELIEQLQK	Periplakin n=3 Tax=Hominoidea RepID=PEPL_HUM	1	1	O60437		0	5.55E-05	2	930.46704	1859.92681	0.46
ALNSIIDVYHKYSLIK	Protein S100-A8 n=5 Tax=Hominoidea RepID=S10A	1	1	P05109		0	4.75E-06	2	939.47949	1877.95171	0.45
aSGVAVSDGVIKVFNDMK	Uncharacterized protein n=2 Tax=Simiiformes RepI	2	1	F7GZR9	N-Term(Acetyl)	0	0.00036	2	940.27332	1879.53935	0.29
AADTDGDGQVNYEEFVR	Calmodulin-like protein 3 n=5 Tax=Euarchontoglire	1	1	P27482		0	8.40E-07	2	943.60938	1886.21147	0.2
AAYLQETGKPLDETLKK	Annexin A1 n=14 Tax=Eutheria RepID=ANXA1_HUM	1	1	P04083		0	1.22E-05	2	953.33984	1905.67241	0.32
LATQSNEITIPVTFESR	Heat shock protein beta-1 n=6 Tax=Simiiformes Rep	1	1	P04792		0	0.0011	2	953.85815	1906.70903	0.36
ITMQNLNDRLASYLEK	Keratin, type I cytoskeletal 13 n=13 Tax=Simiiforme	1	1	P13646		0	8.66E-06	2	955.39563	1909.78398	0.4
TMQNLEIELQSQLSmK	Keratin, type I cytoskeletal 14 n=8 Tax=Eutheria Re	1	1	P02533	M15(Oxidation)	0.00223	0.0238	2	955.46777	1909.92827	0.49
KVPDPVLEESFQQLQR	Periplakin n=3 Tax=Hominoidea RepID=PEPL_HUM	1	1	O60437		0	3.34E-07	2	957.2926	1913.57793	0.28
TFVNITPAEVGVLVGKDR	Profilin-1 n=21 Tax=Theria RepID=PROF1_HUMAN	1	1	P07737		0	1.07E-05	2	958.41199	1915.8167	0.38
ITmQNLNDRLASYLEK	Keratin, type I cytoskeletal 13 n=13 Tax=Simiiforme	1	1	P13646	M3(Oxidation)	0	0.000633	2	963.32922	1925.65117	0.34
TLVTQNSGVEALIHAILR	Junction plakoglobin n=31 Tax=Theria RepID=PLAK	1	1	P14923		0	2.06E-06	2	968.51868	1936.03008	0.46
LNDLEDALQQAKEDLAR	Keratin, type II cytoskeletal 1 n=7 Tax=Eutheria Rep	1	1	P04264		0	4.97E-08	2	971.88403	1942.76079	0.39
FASFIDKVQFLEQQNK	cDNA FLJ55805, highly similar to Keratin, type II cyt	2	1	B4DRW1		0	8.56E-06	2	971.93213	1942.85698	0.43
TVAAPSVFIFPPSDEQLK	Ig kappa chain C region n=2 Tax=Homo sapiens Rep	19	1	P01834		0	4.08E-07	2	973.9137	1946.82012	0.4
RVELEAALQQAKEELAR	cDNA FLJ55805, highly similar to Keratin, type II cyt	2	1	B4DRW1		0	3.59E-07	2	977.62329	1954.23931	0.08
VAPEEHPVLLTEAPLNPK	Uncharacterized protein n=11 Tax=Simiiformes Rep	4	1	F5H0N0		0	3.03E-05	2	977.88489	1954.7625	0.35
THNLEPYFESFINNLR	Keratin, type II cytoskeletal 1 n=7 Tax=Eutheria Rep	1	1	P04264		0	0.000347	2	997.93005	1994.85283	0.44
QLLAGLDKVASDLDRQEK	Periplakin n=3 Tax=Hominoidea RepID=PEPL_HUM	1	1	O60437		0	7.58E-06	3	667.46545	2000.38181	0.43
LSLQDAVSQGVIDQDMATR	Desmoplakin n=28 Tax=Mammalia RepID=DESP_HU	1	1	P15924		0	6.42E-08	2	1023.98975	2046.97222	-0.02
SAELNKEVSTNTAMIQTSK	GUCA1B protein (Fragment) n=1 Tax=Homo sapien	2	2	A4FUA1;P13646		0	6.44E-09	2	1026.86414	2052.72099	0.35
GDRSEDFGVNEDLADSDAR	Annexin A1 n=14 Tax=Eutheria RepID=ANXA1_HUM	1	1	P04083		0	3.93E-07	2	1034.82678	2068.64629	0.38
SAELNKEVSTNTAmIQTSK	GUCA1B protein (Fragment) n=1 Tax=Homo sapien	2	2	A4FUA1;P13646	M14(Oxidation)	0	5.53E-07	2	1034.90991	2068.81255	0.4
LGHPDTLNQGEFKELVRK	Protein S100-A9 n=5 Tax=Hominoidea RepID=S10A	1	1	P06702		0	0.00145	3	694.55145	2081.6398	0.18
GHYTEGAELVDSVLDVVRK	TUBB2B protein (Fragment) n=16 Tax=Fungi/Metaz	4	1	A1L195		0	7.95E-06	3	696.71906	2088.14261	0.36
MLLDIKTRLEQEIATYR	Keratin, type I cytoskeletal 13 n=13 Tax=Simiiforme	1	1	P13646		0.00223	0.0474	3	698.63477	2093.88974	0.25
VFLENVIRDAVTYTEHAK	Histone H4 n=219 Tax=Eukaryota RepID=H4_HUM	1	1	P62805		0	0.000142	3	702.82556	2106.46213	0.45
DSTYSLSSTLTLSKADYEK	Ig kappa chain C region n=2 Tax=Homo sapiens Rep	19	1	P01834		0	4.23E-05	2	1055.43506	2109.86284	0.42
ALPGQLKPFETLLSQNQGGK	Glutathione S-transferase pi (Fragment) n=3 Tax=H	2	1	C7DJS1		0	1.21E-07	2	1063.98804	2126.9688	0.4
TIEELRDKILTATIENNR	Keratin, type I cytoskeletal 13 n=13 Tax=Simiiforme	1	1	P13646		0	0.000803	2	1065.58765	2130.16802	0.51
VDNALQSGNSQESVTEQDSK	Ig kappa chain C region n=2 Tax=Homo sapiens Rep	19	1	P01834		0	4.14E-10	2	1068.84985	2136.69243	0.36
VTMQNLNDRLASYLDKVR	Keratin, type I cytoskeletal 10 n=12 Tax=Eutheria R	2	2	P13645;P02533		0	4.19E-05	3	713.15106	2137.43863	0.44
DIPTNSPELEETLTHTITK	KNG1 protein n=1 Tax=Homo sapiens RepID=Q05CI	3	1	Q05CF8		0	1.16E-06	2	1070.56067	2140.11406	0.52
MQANADQVERDILETQKR	Envoplakin n=5 Tax=Simiiformes RepID=EVPL_HUM	2	1	Q92817		0	2.18E-05	3	716.11145	2146.3198	0.42
THNLEPYFESFINNLRR	Keratin, type II cytoskeletal 1 n=7 Tax=Eutheria Rep	1	1	P04264		0	0.000227	2	1075.90491	2150.80254	0.36
VQQLQISVDQHGDNLKNTK	cDNA FLJ55805, highly similar to Keratin, type II cyt	2	1	B4DRW1		0	1.00E-07	2	1083.71533	2166.42339	0.65
ILTATIENNRVILEIDNAR	Keratin, type I cytoskeletal 13 n=13 Tax=Simiiforme	1	1	P13646		0	0.000273	3	723.83301	2169.48447	0.43
AEAESLYQSKYEELQITAGR	Keratin, type II cytoskeletal 1 n=7 Tax=Eutheria Rep	1	1	P04264		0	2.56E-07	2	1143.55823	2286.10918	-0.01
TDLEMQIESLNEELAYMKK	Keratin, type I cytoskeletal 13 n=13 Tax=Simiiforme	1	1	P13646		0	1.63E-07	2	1143.41467	2285.82207	0.36
SLDLDSIIAEVKAQYEDIAQK	Keratin, type II cytoskeletal 1 n=7 Tax=Eutheria Rep	1	1	P04264		0	2.43E-07	2	1175.63464	2350.26201	0.52
GGPGSAVSPYPTFNPSSDVAALHK	Annexin A1 n=14 Tax=Eutheria RepID=ANXA1_HUM	1	1	P04083		0	3.09E-08	2	1178.9325	2356.85771	0.35
AEAESWYQTKYEELQVTAGR	Keratin, type II cytoskeletal 6A n=25 Tax=Euarchont	2	1	P02538		0	1.77E-05	2	1180.75256	2360.49785	0.69
AYTNFDAERDALNIETAIKTK	Uncharacterized protein n=2 Tax=Simiiformes RepI	2	1	F7GML9		0	0.000924	3	795.4483	2384.33036	0.04
GGGGGGYGSGGSSYGSGGGSYGSGGGGGGGR	Keratin, type II cytoskeletal 1 n=7 Tax=Eutheria Rep	1	1	P04264		0	6.81E-12	2	1192.83008	2384.65288	0.35
AQYEDIAQKSKAEAESLYQSK	Keratin, type II cytoskeletal 1 n=7 Tax=Eutheria Rep	1	1	P04264		0	3.01E-05	3	796.58118	2387.72898	0.19
GGGFGGGSSFGGGSGFSGGGFGGGGFGGGR	Keratin, type II cytoskeletal 2 epidermal n=7 Tax=Si	1	1	P35908		0	2.62E-11	2	1200.41357	2399.81987	0.4
QLFHPEQLITGKEDAANNYAR	Uncharacterized protein n=2 Tax=Simiiformes RepI	12	1	C9K0S6		0	5.45E-06	3	805.98193	2415.93125	0.24
TEAESWYQTKYEELQQTAGR	Uncharacterized protein n=3 Tax=Simiiformes RepI	3	2	E7EU87;UPI00020CE287		0	1.32E-06	2	1209.85938	2418.71147	0.3
NLDLDSIIAEVKAQYEEIAQR	Keratin, type II cytoskeletal 2 epidermal n=7 Tax=Si	3	2	P35908;P02538		0	5.01E-08	2	1210.07056	2419.13384	0.44
VFQSLPHENKPLTLSNYQTNK	Cystatin-B n=14 Tax=Simiiformes RepID=CYTB_HUM	1	1	P04080		0	2.64E-07	2	1229.92249	2458.83769	0.28
ELDDQEKVLDKYEDVVQGLQK	Periplakin n=3 Tax=Hominoidea RepID=PEPL_HUM	1	1	O60437		0	0.000125	3	831.49536	2492.47153	0.41
LDQGNLHTSVSSAQGQDAAQSEEK	Cornulin n=6 Tax=Catarrhini RepID=CRNN_HUMAN	1	1	Q9UBG3		0	1.14E-08	3	834.49414	2501.46787	0.44
SKAEAESLYQSKYEELQITAGR	Keratin, type II cytoskeletal 1 n=7 Tax=Eutheria Rep	1	1	P04264		0	1.75E-06	2	1251.42517	2501.84307	0.3
FASFIDKVQFLEQQNKVLETK	cDNA FLJ55805, highly similar to Keratin, type II cyt	2	1	B4DRW1		0	2.00E-05	2	1257.06409	2513.1209	0.39
LLNNKFASFIDKVQFLEQQNK	cDNA FLJ55805, highly similar to Keratin, type II cyt	2	1	B4DRW1		0	1.40E-06	3	842.55737	2525.65757	0.43
SQYEQLAEQNRKDAEAWFNEK	Keratin, type I cytoskeletal 10 n=12 Tax=Eutheria R	1	1	P13645		0	0.000715	3	862.53772	2585.59861	0.46
VIHDNFGIVEGLMTTVHAITATQK	Glyceraldehyde-3-phosphate dehydrogenase (Frag	3	1	Q0QET7		0	3.13E-11	3	866.22876	2596.67173	0.44
SRAEAESWYQTKYEELQVTAGR	Keratin, type II cytoskeletal 6A n=25 Tax=Euarchont	2	1	P02538		0	2.95E-05	2	1301.95923	2602.91118	0.33
QISNLQQSISDAEQRGENALKDAK	Keratin, type II cytoskeletal 1 n=7 Tax=Eutheria Rep	1	1	P04264		0	0.00243	3	882.12451	2644.35898	0.34
LDQGNLHTSVSSAQGQDAAQSEEKR	Cornulin n=6 Tax=Catarrhini RepID=CRNN_HUMAN	1	1	Q9UBG3		0	6.89E-07	2	1328.9342	2656.86113	0.3
VQWKVDNALQSGNSQESVTEQDSK	Ig kappa chain C region n=2 Tax=Homo sapiens Rep	19	1	P01834		0	1.15E-05	2	1339.51086	2678.01445	0.37
GGGGSFGYSYGGGSGGGFSASSLGGGFGGGSR	Keratin, type I cytoskeletal 9 n=4 Tax=Catarrhini Re	1	1	P35527		0	1.28E-08	2	1353.32922	2705.65117	0.25
SGPFGQIFRPDNFVFGQSGAGNNWAK	TUBB2B protein (Fragment) n=16 Tax=Fungi/Metaz	4	1	A1L195		0	7.27E-10	3	933.84778	2799.52878	0.4
SQTSQAVTGGHTQIQAGSHTETVEQDR	Cornulin n=6 Tax=Catarrhini RepID=CRNN_HUMAN	1	1	Q9UBG3		0	1.80E-07	3	952.15686	2854.45603	0.37
TLAEEQHKNQLLQEELEALQLQLR	Periplakin n=3 Tax=Hominoidea RepID=PEPL_HUM	1	1	O60437		0	4.10E-05	3	959.24072	2875.70761	0.39
NALLSLAKGDRSEDFGVNEDLADSDAR	Annexin A1 n=14 Tax=Eutheria RepID=ANXA1_HUM	1	1	P04083		0	6.80E-06	3	960.36951	2879.09397	0.24
SAELNKEVSTNTAMIQTSKTEITELR	GUCA1B protein (Fragment) n=1 Tax=Homo sapien	2	2	A4FUA1;P13646		0	1.42E-07	3	965.78296	2895.33432	0.29
FLEQQNQVLQTKWELLQQVDTSTR	Keratin, type II cytoskeletal 1 n=7 Tax=Eutheria Rep	1	1	P04264		0	4.47E-05	2	1467.44373	2933.88017	0.68
VLYDAELSQMQTHVSDTSVVLSMDNNR	cDNA FLJ55805, highly similar to Keratin, type II cyt	2	1	B4DRW1		0	7.50E-05	2	1527.08008	3053.15288	0.36
TLYDAELSQMQSHISDTSVVLSMDNNR	Keratin, type II cytoskeletal 3 n=7 Tax=Catarrhini Re	1	1	P12035		0.0043	0.0969	2	1527.85156	3054.69585	0.14
TIDDLKNQILNLTTDNANILLQIDNAR	Keratin, type I cytoskeletal 10 n=12 Tax=Eutheria R	1	1	P13645		0	1.09E-08	3	1018.54565	3053.62241	0.33
DVDAAYLNKVELEAKVDSLNDEINFLK	cDNA FLJ55805, highly similar to Keratin, type II cyt	2	1	B4DRW1		0	9.51E-11	3	1022.4032	3065.19504	-0.12
TLYDAELSQMQSHISDTSVVLSmDNNR	Keratin, type II cytoskeletal 3 n=7 Tax=Catarrhini Re	1	1	P12035	M23(Oxidation)	0	0.000156	3	1024.495	3071.47043	0.35
NHEEEMKEFSNQVVGQVNVEMDATPGIDLTR	Keratin, type I cytoskeletal 13 n=13 Tax=Simiiforme	1	1	P13646		0	1.09E-08	3	1173.32239	3517.95261	0.44
xDALmDEINFmKMFFDAELSQMQTHVSDTSVVLSMDNNR	UPI00020CE287 related cluster n=1 Tax=Homo sap	1	1	UPI00020CE287	X1(H); M5(Oxidation); M11(Oxidation)	0	0.000268	3	1527.08008	4579.22568	0.06
xDALMDEINFMKMFFDAELSQMQTHVSDTSVVLSMDNNR	UPI00020CE287 related cluster n=1 Tax=Homo sap	1	1	UPI00020CE287	N-Term(Acetyl); X1(K)	0.00118	0.00365	3	1527.08008	4579.22568	-0.29
xDALMDEINFMKMFFDAELSQMQTHVSDTSVVLSMDNNR	UPI00020CE287 related cluster n=1 Tax=Homo sap	1	1	UPI00020CE287	N-Term(Acetyl); X1(Q)	0	0.000981	3	1527.08008	4579.22568	-0.28
xDALMDEINFMKMFFDAELSQMQTHVSDTSVVLSMDNNR	UPI00020CE287 related cluster n=1 Tax=Homo sap	1	1	UPI00020CE287	N-Term(Acetyl); X1(E)	0.00118	0.00427	3	1527.33997	4580.00534	-0.34
xDALMDEINFmKMFFDAELSQMQTHVSDTSVVLSMDNNR	UPI00020CE287 related cluster n=1 Tax=Homo sap	1	1	UPI00020CE287	N-Term(Acetyl); X1(I); M11(Oxidation)	0	0.00183	3	1527.85156	4581.54013	0.16
xDALMDEINFmKMFFDAELSQMQTHVSDTSVVLSMDNNR	UPI00020CE287 related cluster n=1 Tax=Homo sap	1	1	UPI00020CE287	N-Term(Acetyl); X1(N); M11(Oxidation)	0.00118	0.00342	3	1527.85156	4581.54013	-0.16
xDALMDEINFmKMFFDAELSQMQTHVSDTSVVLSMDNNR	UPI00020CE287 related cluster n=1 Tax=Homo sap	1	1	UPI00020CE287	X1(R); M11(Oxidation)	0.00118	0.00857	3	1527.85156	4581.54013	-0.18

2DLCSCI_16 Protein Summary

index	Prot_hit	Accession	Description	Mascot Score	Coverage	# Peptides	# PSMs	# AAs	MW [kDa]	calc. pI
1	1.1	297206263	phosphoglycerate kinase [Lactobacillus jensenii JV-V16]	58.12	2.98%	1	1	403	43	5.4
2	1.2	300361304	phosphoglycerate kinase [Lactobacillus gasseri JV-V03]	58.12	2.98%	1	1	403	43	5.78
3	2.1	297206261	enolase [Lactobacillus jensenii JV-V16]	53.42	3.94%	1	1	432	47	4.84
4	2.2	300361306	enolase [Lactobacillus gasseri JV-V03]	53.42	3.94%	1	1	432	46.9	4.81
5	3.1	297205850	elongation factor EF1A [Lactobacillus jensenii JV-V16]	86.52	4.04%	1	1	396	43.6	5.01
6	3.2	300811976	translation elongation factor Tu [Lactobacillus delbrueckii subsp	86.52	4.04%	1	1	396	43.3	5.01
7	3.3	300361496	elongation factor Tu [Lactobacillus gasseri JV-V03]	86.52	4.04%	1	1	396	43.7	4.98
8	4	297205326	D-lactate dehydrogenase [Lactobacillus jensenii JV-V16]	49.62	5.41%	1	1	333	37.1	5.1
9	5	297205124	50S ribosomal protein L31 [Lactobacillus jensenii JV-V16]	100.8	21.18%	1	1	85	9.5	8.94
10	6	P16401	Histone H1.5 n=15 Tax=Eutheria RepID=H15_HUMAN	143.52	9.73%	2	4	226	22.6	10.92
11	7.1	Q71U36	Tubulin alpha-1A chain n=597 RepID=TBA1A_HUMAN	136.77	6.43%	2	3	451	50.1	5.06
12	7.2	F5H5D3	Uncharacterized protein n=5 Tax=Simiiformes RepID=F5H5D3_I	136.77	5.59%	2	3	519	57.7	5.07
13	8	P11021	78 kDa glucose-regulated protein n=68 Tax=Coelomata RepID=(	65.18	2.14%	1	1	654	72.3	5.16
14	9	P15924	Desmoplakin n=28 Tax=Mammalia RepID=DESP_HUMAN	89.64	1.08%	2	3	2871	331.6	6.81
15	10	P04264	Keratin, type II cytoskeletal 1 n=7 Tax=Eutheria RepID=K2C1_HU	2597.2	45.34%	24	60	644	66	8.12
16	11	P13645	Keratin, type I cytoskeletal 10 n=12 Tax=Eutheria RepID=K1C10_	363.47	10.27%	5	9	584	58.8	5.21
17	12	P02538	Keratin, type II cytoskeletal 6A n=25 Tax=Euarchontoglires RepI	1091.85	26.42%	18	42	564	60	8
18	13	Q8N1N4	Keratin, type II cytoskeletal 78 n=7 Tax=Hominoidea RepID=K2C	210.64	11.54%	5	7	520	56.8	6.02
19	14	P06702	Protein S100-A9 n=5 Tax=Hominoidea RepID=S10A9_HUMAN	464.92	28.95%	4	12	114	13.2	6.13
20	15	P98160	Basement membrane-specific heparan sulfate proteoglycan cor	95.09	0.41%	1	1	4391	468.5	6.51
21	16	P13647	Keratin, type II cytoskeletal 5 n=17 Tax=Eutheria RepID=K2C5_H	514.09	20.17%	11	23	590	62.3	7.74
22	17	O60437	Periplakin n=3 Tax=Hominoidea RepID=PEPL_HUMAN	331.08	5.47%	7	10	1756	204.6	5.6
23	18	P62937	Peptidyl-prolyl cis-trans isomerase A n=98 Tax=Theria RepID=PF	156.65	24.24%	3	4	165	18	7.81
24	19.1	E9PFJ3	Uncharacterized protein n=3 Tax=Simiiformes RepID=E9PFJ3_H	502.43	11.25%	6	12	578	63.3	5.31
25	19.2	P07911	Uromodulin n=2 Tax=Catarrhini RepID=UROM_HUMAN	502.43	10.16%	6	12	640	69.7	5.24
26	20.1	F5H0N0	Uncharacterized protein n=11 Tax=Simiiformes RepID=F5H0N0_	342.51	23.42%	7	13	333	37.4	5.58
27	20.2	P63261	Actin, cytoplasmic 2 n=1334 RepID=ACTG_HUMAN	342.51	20.80%	7	13	375	41.8	5.48
28	20.3	B4E335	Uncharacterized protein n=3 Tax=Coelomata RepID=B4E335_HU	342.51	22.22%	7	13	351	39.2	5.59
29	21	P69905	Hemoglobin subunit alpha n=29 Tax=Primates RepID=HBA_HUM	139.96	10.56%	1	2	142	15.2	8.68
30	22	A4FUA1	GUCA1B protein (Fragment) n=1 Tax=Homo sapiens RepID=A4F	929.78	68.00%	4	19	75	8.6	5.41
31	23.1	P60524	Hemoglobin subunit beta n=66 Tax=Eutheria RepID=HBB_CANF	57.37	9.59%	1	1	146	16	8.05
32	23.2	P68871	Hemoglobin subunit beta n=102 Tax=Primates RepID=HBB_HUM	57.37	9.52%	1	1	147	16	7.28
33	24	P13646	Keratin, type I cytoskeletal 13 n=13 Tax=Simiiformes RepID=K1C	2444.24	43.45%	23	77	458	49.6	4.96
34	25.1	E7EUT5	Glyceraldehyde-3-phosphate dehydrogenase n=3 Tax=Eutheria	136.15	10.38%	2	3	260	27.9	6.95
35	25.2	P04406	Glyceraldehyde-3-phosphate dehydrogenase n=42 Tax=Eutheria	136.15	8.06%	2	3	335	36	8.46
36	26.1	B4DRW1	cDNA FLJ55805, highly similar to Keratin, type II cytoskeletal 4 n	2441.99	48.10%	26	70	474	51.7	6.81
37	26.2	P19013	Keratin, type II cytoskeletal 4 n=14 Tax=Simiiformes RepID=K2C	2441.99	42.70%	26	70	534	57.2	6.61
38	27	F6RMN8	Uncharacterized protein n=4 Tax=Simiiformes RepID=F6RMN8_	1046.42	27.85%	17	41	535	57.8	8.22
39	28.1	E9PF79	Pyruvate kinase n=4 Tax=Simiiformes RepID=E9PF79_HUMAN	162.84	7.42%	2	3	458	50	7.64
40	28.2	P14618	Pyruvate kinase isozymes M1/M2 n=16 Tax=Simiiformes RepID=	162.84	6.40%	2	3	531	57.9	7.84
41	28.3	B4DRT3	Pyruvate kinase n=2 Tax=Homo sapiens RepID=B4DRT3_HUMA	162.84	6.65%	2	3	511	55.9	7.5
42	28.4	B4DUU6	Pyruvate kinase n=4 Tax=Simiiformes RepID=B4DUU6_HUMAN	162.84	6.59%	2	3	516	56.2	8.44
43	28.5	P52480	Pyruvate kinase isozymes M1/M2 n=40 Tax=Tetrapoda RepID=K	162.84	6.40%	2	3	531	57.8	7.47
44	29.1	P06899	Histone H2B type 1-J n=102 Tax=Euteleostomi RepID=H2B1J_HU	168.71	30.16%	3	4	126	13.9	10.32
45	29.2	P62807	Histone H2B type 1-C/E/F/G/I n=44 Tax=Euteleostomi RepID=H2	168.71	30.16%	3	4	126	13.9	10.32
46	29.3	B4DR52	Histone H2B n=5 Tax=Euarchontoglires RepID=B4DR52_HUMAN	168.71	22.89%	3	4	166	18	10.32
47	29.4	P58876	Histone H2B type 1-D n=136 Tax=Coelomata RepID=H2B1D_HU	168.71	30.16%	3	4	126	13.9	10.32
48	30	P04792	Heat shock protein beta-1 n=6 Tax=Simiiformes RepID=HSPB1_I	824.76	56.59%	9	20	205	22.8	6.4
49	31	P09211	Glutathione S-transferase P n=4 Tax=Simiiformes RepID=GSTP1	150.3	14.76%	2	3	210	23.3	5.64
50	32	P04083	Annexin A1 n=14 Tax=Eutheria RepID=ANXA1_HUMAN	1262.15	54.91%	17	28	346	38.7	7.02
51	33	UPI00020CE287	UPI00020CE287 related cluster n=1 Tax=Homo sapiens RepID=U	359.9	51.74%	6	27	201	23.1	4.97
52	34.1	Q06830	Peroxiredoxin-1 n=31 Tax=Mammalia RepID=PRDX1_HUMAN	73.38	6.03%	1	1	199	22.1	8.13
53	34.2	E9PEN3	Uncharacterized protein n=4 Tax=Eutheria RepID=E9PEN3_HUM	73.38	4.67%	1	1	257	29.3	5.69
54	34.3	Q13162	Peroxiredoxin-4 n=11 Tax=Eutheria RepID=PRDX4_HUMAN	73.38	4.43%	1	1	271	30.5	6.29
55	35.1	A9X7H1	Eukaryotic translation elongation factor 1 alpha (Fragment) n=2	100.31	7.34%	1	2	177	19.4	7.28
56	35.2	P68104	Elongation factor 1-alpha 1 n=117 Tax=Euteleostomi RepID=EF1	100.31	2.81%	1	2	462	50.1	9.01
57	35.3	Q6IQ15	EEF1A1 protein n=17 Tax=Theria RepID=Q6IQ15_HUMAN	100.31	2.95%	1	2	441	47.8	9.03
58	36.1	F2Z2S7	Uncharacterized protein n=10 Tax=Catarrhini RepID=F2Z2S7_HU	81.19	1.08%	1	1	1478	167.9	6.62
59	36.2	P35579	Myosin-9 n=67 Tax=Tetrapoda RepID=MYH9_HUMAN	81.19	0.82%	1	1	1960	226.4	5.6
60	36.3	P35749	Myosin-11 n=82 Tax=Amniota RepID=MYH11_HUMAN	81.19	0.81%	1	1	1972	227.2	5.5
61	36.4	F2Z2U9	Uncharacterized protein n=4 Tax=Catarrhini RepID=F2Z2U9_HU	81.19	0.90%	1	1	1779	203	6.27
62	36.5	Q7Z406	Myosin-14 n=13 Tax=Eutheria RepID=MYH14_HUMAN	81.19	0.80%	1	1	1995	227.7	5.6
63	37	P14923	Junction plakoglobin n=31 Tax=Theria RepID=PLAK_HUMAN	103.46	2.42%	1	1	745	81.7	6.14
64	38	P07355	Annexin A2 n=28 Tax=Eutheria RepID=ANXA2_HUMAN	256.22	21.83%	4	8	339	38.6	7.75
65	39	Q9UBG3	Cornulin n=6 Tax=Catarrhini RepID=CRNN_HUMAN	661.89	24.04%	6	10	495	53.5	6.1
66	40	P04080	Cystatin-B n=14 Tax=Simiiformes RepID=CYTB_HUMAN	311.22	45.92%	3	8	98	11.1	7.56
67	41.1	P62784	Histone H4 n=232 Tax=Eukaryota RepID=H4_CAEEL	80.56	11.65%	1	3	103	11.4	11.19
68	41.2	P62805	Histone H4 n=219 Tax=Eukaryota RepID=H4_HUMAN	80.56	11.65%	1	3	103	11.4	11.36
69	42	P31947	14-3-3 protein sigma n=20 Tax=Eutheria RepID=1433S_HUMAN	148.8	17.34%	3	5	248	27.8	4.74
70	43	P06733	Alpha-enolase n=53 Tax=Euteleostomi RepID=ENOA_HUMAN	102.42	9.45%	2	2	434	47.1	7.39
71	44.1	A4K467	Actinin alpha4 isoform n=3 Tax=Simiiformes RepID=A4K467_HU	82.19	3.26%	1	1	521	59.5	4.94
72	44.2	O43707	Alpha-actinin-4 n=40 Tax=Tetrapoda RepID=ACTN4_HUMAN	82.19	1.87%	1	1	911	104.8	5.44

index	Prot_hit	Accession	Description	Mascot Score	Coverage	# Peptides	# PSMs	# AAs	MW [kDa]	calc. pI
73	44.3	D6PXK4	Alpha actinin 4 short isoform n=8 Tax=Eutheria RepID=D6PXK4_	82.19	2.46%	1	1	692	79.9	5.17
74	45	P29508	Serpin B3 n=14 Tax=Hominoidea RepID=SPB3_HUMAN	301.43	14.36%	5	7	390	44.5	6.81
75	46	Q01469	Fatty acid-binding protein, epidermal n=10 Tax=Simiiformes Rep	92.98	11.85%	1	2	135	15.2	7.01
76	47.1	C9J0D1	Histone H2A n=1 Tax=Homo sapiens RepID=C9J0D1_HUMAN	118.31	7.38%	1	3	122	13.2	9.99
77	47.2	P0C0S5	Histone H2A.Z n=78 Tax=Metazoa RepID=H2AZ_HUMAN	118.31	7.03%	1	3	128	13.5	10.58
78	47.3	P0C0S8	Histone H2A type 1 n=295 Tax=Eukaryota RepID=H2A1_HUMAN	118.31	6.92%	1	3	130	14.1	10.9
79	47.4	P16104	Histone H2A.x n=16 Tax=Coelomata RepID=H2AX_HUMAN	118.31	6.29%	1	3	143	15.1	10.74
80	47.5	Q16777	Histone H2A type 2-C n=33 Tax=Eumetazoa RepID=H2A2C_HUM	118.31	6.98%	1	3	129	14	10.9
81	47.6	Q71UI9	Histone H2A.V n=25 Tax=Bilateria RepID=H2AV_HUMAN	118.31	7.03%	1	3	128	13.5	10.58
82	47.7	C9JE22	Histone H2A n=7 Tax=Eutheria RepID=C9JE22_HUMAN	118.31	5.96%	1	3	151	16.1	10.23
83	47.8	Q96KK5	Histone H2A type 1-H n=10 Tax=Mammalia RepID=H2A1H_HUM	118.31	7.03%	1	3	128	13.9	10.89
84	47.9	Q99878	Histone H2A type 1-J n=4 Tax=Eutheria RepID=H2A1J_HUMAN	118.31	7.03%	1	3	128	13.9	10.89
85	47.10	Q8IUE6	Histone H2A type 2-B n=5 Tax=Eutheria RepID=H2A2B_HUMAN	118.31	6.92%	1	3	130	14	10.89
86	48.1	Q92817	Envoplakin n=5 Tax=Simiiformes RepID=EVPL_HUMAN	125.85	1.48%	2	2	2033	231.5	6.96
87	48.2	B7ZLH8	EVPL protein n=4 Tax=Simiiformes RepID=B7ZLH8_HUMAN	125.85	1.46%	2	2	2055	233.7	7.25
88	49.1	P16403	Histone H1.2 n=12 Tax=Eutheria RepID=H12_HUMAN	162.98	12.68%	2	3	213	21.4	10.93
89	49.2	P10412	Histone H1.4 n=20 Tax=Eutheria RepID=H14_HUMAN	162.98	12.33%	2	3	219	21.9	11.03
90	49.3	P16402	Histone H1.3 n=6 Tax=Simiiformes RepID=H13_HUMAN	162.98	12.22%	2	3	221	22.3	11.02
91	49.4	Q02539	Histone H1.1 n=5 Tax=Catarrhini RepID=H11_HUMAN	162.98	12.56%	2	3	215	21.8	10.99
92	50.1	F5H651	Uncharacterized protein n=5 Tax=Catarrhini RepID=F5H651_HU	72.82	2.01%	1	1	646	72.8	9.26
93	50.2	O95171	Sciellin n=6 Tax=Catarrhini RepID=SCEL_HUMAN	72.82	1.89%	1	1	688	77.5	9.38
94	51	P18054	Arachidonate 12-lipoxygenase, 12S-type n=5 Tax=Eutheria RepI	51.74	1.81%	1	1	663	75.6	6.21
95	52.1	P07476	Involucrin n=2 Tax=Homo sapiens RepID=INVO_HUMAN	96.11	4.79%	2	2	585	68.4	4.61
96	52.2	B4DU44	Involucrin n=2 Tax=Homininae RepID=B4DU44_HUMAN	96.11	4.71%	2	2	595	69.6	4.6
97	52.3	Q28423	Involucrin n=2 Tax=Homininae RepID=Q28423_9PRIM	96.11	4.41%	2	2	635	74	4.61
98	53.1	E9PI65	Uncharacterized protein n=1 Tax=Homo sapiens RepID=E9PI65_	125.61	10.12%	1	2	168	17.9	5.29
99	53.2	P11142	Heat shock cognate 71 kDa protein n=167 Tax=Metazoa RepID=	125.61	2.63%	1	2	646	70.9	5.52
100	53.3	P54652	Heat shock-related 70 kDa protein 2 n=38 Tax=Amniota RepID=	125.61	2.66%	1	2	639	70	5.74
101	53.4	B3KTV0	cDNA FLJ38781 fis, clone LIVER2000216, highly similar to HEAT S	125.61	2.74%	1	2	621	67.9	5.45
102	54.1	F6Y193	Uncharacterized protein n=3 Tax=Simiiformes RepID=F6Y193_C	51.74	3.12%	1	1	417	45.1	5.73
103	54.2	P08107	Heat shock 70 kDa protein 1A/1B n=76 Tax=Gnathostomata Rep	51.74	2.03%	1	1	641	70	5.66
104	54.3	E7EQL7	Uncharacterized protein n=2 Tax=Homo sapiens RepID=E7EQL7	51.74	2.31%	1	1	563	61.4	5.69
105	55	P63104	14-3-3 protein zeta/delta n=26 Tax=Amniota RepID=1433Z_HUI	131.68	13.06%	2	2	245	27.7	4.79
106	56	Q09666	Neuroblast differentiation-associated protein AHNAK n=14 Tax=	110.96	1.66%	1	4	5890	628.7	6.15
107	57	C9JZ65	Uncharacterized protein n=1 Tax=Homo sapiens RepID=C9JZ65_	201.68	19.91%	3	4	211	24.4	6.61
108	58	P01040	Cystatin-A n=6 Tax=Catarrhini RepID=CYTA_HUMAN	124.73	14.29%	1	2	98	11	5.5
109	59	Q9UBC9	Small proline-rich protein 3 n=4 Tax=Homininae RepID=SPRR3_I	251.16	27.81%	3	7	169	18.1	8.57
110	60.1	B4DFK7	Carbonyl reductase 1, isoform CRA_c n=2 Tax=Simiiformes RepI	94.3	9.83%	1	2	173	18.7	8.02
111	60.2	A8MTM1	Uncharacterized protein n=1 Tax=Homo sapiens RepID=A8MTM	94.3	7.66%	1	2	222	24.5	8.76
112	60.3	E9PQ63	Uncharacterized protein n=1 Tax=Homo sapiens RepID=E9PQ63	94.3	9.55%	1	2	178	19	6.3
113	60.4	P16152	Carbonyl reductase [NADPH] 1 n=7 Tax=Simiiformes RepID=CBF	94.3	6.14%	1	2	277	30.4	8.32
114	61	P18510	Interleukin-1 receptor antagonist protein n=4 Tax=Catarrhini Re	86.27	9.04%	1	2	177	20	6.19
115	62.1	P01834	Ig kappa chain C region n=2 Tax=Homo sapiens RepID=IGKC_HU	57.53	16.98%	1	1	106	11.6	5.87
116	62.2	UPI000011049E	IGG CTM01 FAB (LIGHT CHAIN) n=2 Tax=Homo sapiens RepID=U	57.53	8.22%	1	1	219	24.1	7.74
117	62.3	Q0KKI6	Immunoblobulin light chain (Fragment) n=3 Tax=Homo sapiens	57.53	8.22%	1	1	219	24	8.06
118	62.4	Q569I7	Putative uncharacterized protein n=1 Tax=Homo sapiens RepID=	57.53	9.52%	1	1	189	20.7	5.27
119	62.5	Q5EFE6	Anti-RhD monoclonal T125 kappa light chain n=1 Tax=Homo sap	57.53	7.69%	1	1	234	25.7	8.43
120	62.6	Q6GMX0	Putative uncharacterized protein n=14 Tax=Euarchontoglires Re	57.53	7.63%	1	1	236	25.8	7.97
121	62.7	Q6P5S8	IGK@ protein n=6 Tax=Homo sapiens RepID=Q6P5S8_HUMAN	57.53	7.63%	1	1	236	25.8	6.33
122	62.8	Q7Z3Y4	Putative uncharacterized protein n=3 Tax=Homininae RepID=Q7	57.53	7.63%	1	1	236	25.7	7.59
123	62.9	UPI0000110495	ANTIBODY A5B7 (LIGHT CHAIN) n=1 Tax=Homo sapiens RepID=I	57.53	8.45%	1	1	213	23.1	8.06
124	62.10	UPI0000110769	ANTIBODY n=1 Tax=Homo sapiens RepID=UPI0000110769	57.53	8.41%	1	1	214	23.6	6.33
125	62.11	UPI000014CDC4	OMALIZUMAB LIGHT CHAIN n=2 Tax=Homo sapiens RepID=UPI	57.53	8.26%	1	1	218	23.9	5.39
126	62.12	UPI00003CEBF2	HU3S193 Fab fragment, light chain n=3 Tax=Euarchontoglires Re	57.53	8.22%	1	1	219	24	8.06
127	62.13	UPI00006EB13F	Fab m18, Light Chain n=1 Tax=Homo sapiens RepID=UPI00006E	57.53	8.45%	1	1	213	23.4	7.27
128	62.14	UPI0001A7C522	HUMAN IGG ABT-325 n=1 Tax=Homo sapiens RepID=UPI0001A7	57.53	8.41%	1	1	214	23.5	6.55
129	62.15	UPI0001A7C54C	Fab Z13e1 n=3 Tax=Euarchontoglires RepID=UPI0001A7C54C	57.53	8.49%	1	1	212	23.1	7.71
130	62.16	UPI0001C591D8	Humanized recombinant Fab fragment of a murin n=5 Tax=Euar	57.53	7.86%	1	1	229	25.1	5.62
131	62.17	UPI0001D3410B	Immunoglobulin light chain n=1 Tax=Homo sapiens RepID=UPI0	57.53	8.26%	1	1	218	23.3	8.05
132	62.18	UPI0001F2FE82	Fab 2G12, light chain n=2 Tax=Homo sapiens RepID=UPI0001F2	57.53	8.45%	1	1	213	23.2	8.13
133	62.19	UPI0002003CF0	IMMUNOGLOBULIN A1 LIGHT CHAIN n=1 Tax=Homo sapiens Re	57.53	8.22%	1	1	219	24	6.77
134	62.20	UPI000211B3C2	B3 (IGKV4-1) light chain n=1 Tax=Homo sapiens RepID=UPI0002	57.53	8.18%	1	1	220	24.2	6.55
135	62.21	UPI0002177BCC	S4 FAB LIGHT CHAIN n=1 Tax=Homo sapiens RepID=UPI000217	57.53	8.53%	1	1	211	22.7	7.77
136	63	P37802	Transgelin-2 n=14 Tax=Theria RepID=TAGL2_HUMAN	96.81	9.05%	1	1	199	22.4	8.25
137	64.1	B4E1Q0	Uncharacterized protein n=2 Tax=Theria RepID=B4E1Q0_HUMA	50.38	12.82%	1	1	156	17.3	5.55
138	64.2	B3KQV6	cDNA FLJ33169 fis, clone ADRGL2000384, highly similar to Serin	50.38	4.88%	1	1	410	45.6	5.36
139	64.3	B4DQY1	cDNA FLJ56133, highly similar to Serine/threonine-protein phos	50.38	3.60%	1	1	555	61.5	5.12
140	64.4	E9PH38	Uncharacterized protein n=2 Tax=Homo sapiens RepID=E9PH38	50.38	3.93%	1	1	509	56.8	5.69
141	64.5	P30153	Serine/threonine-protein phosphatase 2A 65 kDa regulatory sul	50.38	3.40%	1	1	589	65.3	5.11

2DLCSCI_16 Peptide Summary

Sequence	Protein Descriptions	# Proteins	# Protein Groups	Protein Group Accessions	Modifications	q-Value	PEP	Charge	m/z [Da]	MH+ [Da]	Delta Mass [Da]
LASYLEK	Keratin, type I cytoskeletal 13 n=13 Tax=Simiiforme	1	1	P13646		0	0.0202	2	412.52518	824.04308	0.29
VDLHLPR	Serpin B3 n=14 Tax=Hominoidea RepID=SPB3_HUM	1	1	P29508		0.00633	0.164	2	425.52203	850.03679	0.27
SLVNLGGSK	Keratin, type II cytoskeletal 1 n=7 Tax=Eutheria Rep	1	1	P04264		0	6.54E-06	2	438.15469	875.30211	0.4
FSVQMFR	Uncharacterized protein n=3 Tax=Simiiformes RepII	2	1	E9PFJ3		0	0.0323	2	458.03717	915.06706	0.31
AGLQFPVGR	Histone H2A n=1 Tax=Homo sapiens RepID=C9J0D1	10	1	C9J0D1		0	2.10E-05	2	473.23035	945.45342	0.46
LLLPGELAK	Histone H2B type 1-J n=102 Tax=Euteleostomi RepI	4	1	P06899		0	0.0306	2	477.57776	954.14824	0.27
SLLEGQDAK	Keratin, type I cytoskeletal 13 n=13 Tax=Simiiforme	1	1	P13646		0	0.00139	2	481.03378	961.06029	0.28
DVDGVFLSK	Keratin, type II cytoskeletal 78 n=7 Tax=Hominoide	1	1	Q8N1N4		0.00258	0.113	1	979.59631	979.59631	0.09
IISTTTLNK	cDNA FLJ55805, highly similar to Keratin, type II cyt	2	1	B4DRW1		0	1.18E-06	2	496.38043	991.75359	0.59
SISMSVAGSR	cDNA FLJ55805, highly similar to Keratin, type II cyt	2	1	B4DRW1		0	0.0219	2	498.40814	995.80901	0.66
DLTDYLMK	Uncharacterized protein n=11 Tax=Simiiformes Rep	3	1	F5H0N0		0	0.0165	1	998.63318	998.63318	0.15
LLEGEEYR	cDNA FLJ55805, highly similar to Keratin, type II cyt	2	1	B4DRW1		0	0.00405	2	504.94556	1008.88384	0.19
SGFSSVSVSR	Keratin, type II cytoskeletal 6A n=25 Tax=Euarchont	1	1	P02538		0	1.33E-10	2	507.0036	1012.99993	0.25
DLTDYLmK	Uncharacterized protein n=11 Tax=Simiiformes Rep	3	1	F5H0N0	M7(Oxidation)	0	0.00238	1	1014.57007	1014.57007	0.09
NGLSLAALKK	Histone H1.5 n=15 Tax=Eutheria RepID=H15_HUM	1	1	P16401		0	1.88E-05	2	508.00513	1015.00298	0.19
TLLEGEESR	Keratin, type II cytoskeletal 1 n=7 Tax=Eutheria Rep	1	1	P04264		0	0.00587	2	517.55402	1034.10076	0.29
SLYGLGGSKR	Keratin, type II cytoskeletal 6A n=25 Tax=Euarchont	1	1	P02538		0	1.81E-06	2	519.74011	1038.47295	0.45
VILEIDNAR	Keratin, type I cytoskeletal 13 n=13 Tax=Simiiforme	1	1	P13646		0	0.000898	2	522.00427	1043.00127	0.21
LASYLDKVR	Keratin, type I cytoskeletal 10 n=12 Tax=Eutheria R	2	1	P13645		0	0.000148	2	533.09741	1065.18755	0.29
AQYEDIAQK	Keratin, type II cytoskeletal 1 n=7 Tax=Eutheria Rep	1	1	P04264		0	0.000114	2	533.50464	1066.002	0.24
QLSSGVSEIR	Heat shock protein beta-1 n=6 Tax=Simiiformes Rep	1	1	P04792		0	8.52E-05	2	538.66748	1076.32768	0.38
FASFIDKVR	Keratin, type II cytoskeletal 74 n=7 Tax=Simiiformes	4	3	P02538;P13647;F6RMN8		0	5.28E-06	2	541.98688	1082.96648	0.18
AQYEEIANR	Keratin, type II cytoskeletal 5 n=17 Tax=Eutheria Re	2	2	P13647;UPI00020CE287		0	0.00351	2	547.78467	1094.56206	0.52
ITMQNLNDR	Keratin, type I cytoskeletal 13 n=13 Tax=Simiiforme	2	1	P13646		0	4.89E-07	2	553.04883	1105.09038	0.27
AQYEEIAQR	cDNA FLJ55805, highly similar to Keratin, type II cyt	4	3	B4DRW1;P02538;F6RMN8		0	0.0217	2	554.68994	1108.37261	0.42
DAEAWFNEK	Keratin, type I cytoskeletal 10 n=12 Tax=Eutheria R	1	1	P13645		0.00258	0.0606	1	1109.58411	1109.58411	0.09
ISISTSGGSFR	Keratin, type II cytoskeletal 5 n=17 Tax=Eutheria Re	1	1	P13647		0	0.0262	1	1111.65625	1111.65625	0.08
LEQEIATYR	Keratin, type I cytoskeletal 19 n=9 Tax=Eutheria Rep	2	1	P13646		0	5.42E-07	2	562.22076	1123.43425	0.43
EQYEAMAER	Keratin, type I cytoskeletal 13 n=13 Tax=Simiiforme	1	1	P13646		0	0.000427	2	564.22296	1127.43865	0.48
DWVSVVTPAR	Uncharacterized protein n=3 Tax=Simiiformes RepII	2	1	E9PFJ3		0	1.02E-05	1	1129.58435	1129.58435	-0.02
DAEEWFHAK	GUCA1B protein (Fragment) n=1 Tax=Homo sapiens	1	1	A4FUA1		0	0.000845	2	566.83618	1132.66509	0.08
KLLEGEEYR	cDNA FLJ55805, highly similar to Keratin, type II cyt	2	1	B4DRW1		0	0.00318	2	569.0556	1137.10393	0.25
ILTATIENNR	Keratin, type I cytoskeletal 13 n=13 Tax=Simiiforme	1	1	P13646		0	4.74E-07	2	573.05511	1145.10295	0.24
EITALAPSTMK	Uncharacterized protein n=11 Tax=Simiiformes Rep	3	1	F5H0N0		0	0.00051	1	1161.71216	1161.71216	0.09
LFDQAFGLPR	Heat shock protein beta-1 n=6 Tax=Simiiformes Rep	1	1	P04792		0	1.05E-06	2	582.55945	1164.11162	0.25
YEELQVTAGR	Keratin, type II cytoskeletal 6A n=25 Tax=Euarchont	2	2	P02538;F6RMN8		0	6.60E-06	2	583.55066	1166.09404	0.25
HQGVMVGMGQK	Uncharacterized protein n=11 Tax=Simiiformes Rep	3	1	F5H0N0		0	2.07E-05	2	586.70178	1172.39629	0.41
YEELQITAGR	Keratin, type II cytoskeletal 1 n=7 Tax=Eutheria Rep	1	1	P04264		0	8.70E-09	2	590.55511	1180.10295	0.25
AVFPSIVGRPR	Uncharacterized protein n=11 Tax=Simiiformes Rep	3	1	F5H0N0		0	8.03E-05	2	600.35956	1199.71184	0.5
WTLLQEQGTK	Keratin, type II cytoskeletal 6A n=25 Tax=Euarchont	3	3	P02538;P13647;F6RMN8		0	6.49E-06	2	602.77179	1204.5363	0.45
AEAESWYQTK	Keratin, type II cytoskeletal 6A n=25 Tax=Euarchont	2	2	P02538;F6RMN8		0	0.00421	2	607.26416	1213.52104	0.48
DITSDTSGDFR	Annexin A1 n=14 Tax=Eutheria RepID=ANXA1_HUM	1	1	P04083		0	6.05E-08	1	1213.62659	1213.62659	0.09
LKYENELALR	Keratin, type I cytoskeletal 13 n=13 Tax=Simiiforme	1	1	P13646		0	4.40E-06	2	625.30457	1249.60185	0.45
SLVSKGTLVQTK	Histone H1.2 n=12 Tax=Eutheria RepID=H12_HUM	5	2	P16403;P16401		0	4.23E-06	2	631.32135	1261.63542	0.44
TPAQFDADELR	Annexin A1 n=14 Tax=Eutheria RepID=ANXA1_HUM	1	1	P04083		0	5.65E-10	2	632.00928	1263.01128	0.21
GAFSSVSMSGGAGR	cDNA FLJ55805, highly similar to Keratin, type II cyt	2	1	B4DRW1		0	0.000139	2	636.01208	1271.01689	0.22
LALDLEIATYR	Keratin, type II cytoskeletal 1 n=7 Tax=Eutheria Rep	3	2	P04264;B4DRW1		0	2.17E-05	2	639.59448	1278.18169	0.24
VVLQQDPQQAR	Periplakin n=3 Tax=Hominoidea RepID=PEPL_HUM	1	1	O60437		0	7.53E-05	2	641.4812	1281.95513	0.13
GAFSSVSmSGGAGR	cDNA FLJ55805, highly similar to Keratin, type II cyt	2	1	B4DRW1	M8(Oxidation)	0	1.47E-07	2	644.19836	1287.38945	0.41
ALEEANADLEVK	Keratin, type I cytoskeletal 13 n=13 Tax=Simiiforme	1	1	P13646		0	1.00E-09	2	651.55701	1302.10674	0.22
SLDLDSIIAEVK	Keratin, type II cytoskeletal 1 n=7 Tax=Eutheria Rep	1	1	P04264		0	9.15E-09	2	652.10132	1303.19536	0.24
DNIQGITKPAIR	Histone H4 n=232 Tax=Eukaryota RepID=H4_CAEEL	2	1	P62784		0	4.37E-06	2	663.71619	1326.4251	0.34
SQVVAGTNYFIK	Cystatin-B n=14 Tax=Simiiformes RepID=CYTB_HUM	1	1	P04080		0	0.00523	1	1326.70703	1326.70703	0
NLDLDSIIAEVK	Keratin, type II cytoskeletal 6A n=25 Tax=Euarchont	4	4	P02538;P13647;F6RMN8;UPI00020CE287		0	2.14E-09	2	665.60223	1330.19719	0.24
VVDLMAHMASKE	Glyceraldehyde-3-phosphate dehydrogenase n=3 Ta	2	1	E7EUT5		0	3.17E-08	2	666.22852	1331.44975	0.4
TAAENDFVVLKK	cDNA FLJ55805, highly similar to Keratin, type II cyt	2	1	B4DRW1		0	1.01E-07	2	668.40863	1335.80998	0.54
STEGGQSLDSLIK	Uncharacterized protein n=5 Tax=Catarrhini RepID=	2	1	F5H651		0	2.08E-10	2	668.38196	1335.75664	0.54
SKAEAEALYQTK	cDNA FLJ55805, highly similar to Keratin, type II cyt	3	1	B4DRW1		0	1.20E-07	2	670.14014	1339.273	0.29
PPYTVVYFPVR	Glutathione S-transferase P n=4 Tax=Simiiformes R	1	1	P09211		0	2.52E-05	2	669.87915	1338.75102	0.51
SKAEAESLYQSK	Keratin, type II cytoskeletal 1 n=7 Tax=Eutheria Rep	1	1	P04264		0	6.47E-09	2	671.25769	1341.5081	0.42
LPGDNALDMFQK	Arachidonate 12-lipoxygenase, 12S-type n=5 Tax=E	1	1	P18054		0	2.73E-05	2	675.51257	1350.01787	0.68
SLFEADKVELAK	phosphoglycerate kinase [Lactobacillus jensenii JV-V	2	1	297206263		0	4.18E-05	2	675.7948	1350.58232	0.43
TAAENEFVTLKK	Keratin, type II cytoskeletal 6A n=25 Tax=Euarchont	2	2	P02538;F6RMN8		0	5.66E-07	2	676.03467	1351.06206	0.17
NLDLDSIIAEVR	cDNA FLJ55805, highly similar to Keratin, type II cyt	2	1	B4DRW1		0	4.78E-10	2	679.59106	1358.17485	0.22
NKLEGLEDALQK	Keratin, type II cytoskeletal 6A n=25 Tax=Euarchont	2	2	P02538;F6RMN8		0	3.22E-05	2	679.66077	1358.31426	0.29
GLFIIDDKGILR	Peroxiredoxin-1 n=31 Tax=Mammalia RepID=PRDX	3	1	Q06830		0	3.73E-05	2	680.59033	1360.17339	0.19
SAYQEAMDISKK	14-3-3 protein sigma n=20 Tax=Eutheria RepID=143	1	1	P31947		0	2.36E-05	2	686.09045	1371.17363	0.26
ATLENDFVVLKK	Keratin, type II cytoskeletal 78 n=7 Tax=Hominoide	1	1	Q8N1N4		0	0.000344	2	688.95044	1376.8936	0.06
TRLEQEIATYR	Keratin, type I cytoskeletal 13 n=13 Tax=Simiiforme	1	1	P13646		0	0.000396	2	690.62354	1380.23979	0.26
VSFELFADKVPK	Peptidyl-prolyl cis-trans isomerase A n=98 Tax=The	1	1	P62937		0	3.96E-07	2	690.89929	1380.79131	0.52
DKILTATIENNR	Keratin, type I cytoskeletal 13 n=13 Tax=Simiiforme	1	1	P13646		0	5.12E-06	2	694.95245	1388.89763	0.57
LALDVEIATYRK	Keratin, type II cytoskeletal 6A n=25 Tax=Euarchont	4	4	P02538;P13647;F6RMN8;UPI00020CE287		0	0.000184	2	696.64734	1392.2874	0.25
MIGFPSSAGSVSPR	Keratin, type I cytoskeletal 13 n=13 Tax=Simiiforme	1	1	P13646		0	1.18E-14	2	697.31158	1393.61589	0.46
TNAENEFVTIKK	Keratin, type II cytoskeletal 1 n=7 Tax=Eutheria Rep	1	1	P04264		0	6.31E-07	2	697.60541	1394.20354	0.24
VDSLNDEINFLK	cDNA FLJ55805, highly similar to Keratin, type II cyt	2	1	B4DRW1		0	1.69E-06	2	703.98938	1406.97148	0.13
LALDIEIATYRK	cDNA FLJ55805, highly similar to Keratin, type II cyt	2	1	B4DRW1		0	5.50E-05	2	703.90839	1406.8095	0.5
ADTLTDEINFLR	Keratin, type II cytoskeletal 6A n=25 Tax=Euarchont	2	2	P02538;F6RMN8		0	5.85E-08	2	704.59204	1408.17681	0.23
mIGFPSSAGSVSPR	Keratin, type I cytoskeletal 13 n=13 Tax=Simiiforme	1	1	P13646	M1(Oxidation)	0	1.17E-13	2	705.27478	1409.54228	0.43
SFSTASAITPSVSR	Keratin, type II cytoskeletal 5 n=17 Tax=Eutheria Re	1	1	P13647		0	6.73E-09	1	1410.7207	1410.7207	0
GALQNIIPASTGAAK	Glyceraldehyde-3-phosphate dehydrogenase n=3 Ta	2	1	E7EUT5		0	5.58E-06	2	706.82373	1412.64018	0.43
ELDVEVSRLEAR	Periplakin n=3 Tax=Hominoidea RepID=PEPL_HUM	1	1	O60437		0	0.00104	2	708.82788	1416.64849	0.45
VHVGDEDFVHLR	Cystatin-B n=14 Tax=Simiiformes RepID=CYTB_HUM	1	1	P04080		0	3.88E-07	2	712.32471	1423.64214	0.47
YISPDQLADLYK	Alpha-enolase n=53 Tax=Euteleostomi RepID=ENO	1	1	P06733		0	1.01E-06	2	713.80237	1426.59746	0.44
SSKAEAEALYQTK	Keratin, type II cytoskeletal 78 n=7 Tax=Hominoide	1	1	Q8N1N4		0	3.56E-06	2	713.81158	1426.61589	0.45
RGAFSSVSMSGGAGR	cDNA FLJ55805, highly similar to Keratin, type II cyt	2	1	B4DRW1		0	2.03E-13	2	714.29907	1427.59087	0.45
TALQPmVSALNIR	Uncharacterized protein n=3 Tax=Simiiformes RepII	2	1	E9PFJ3	M6(Oxidation)	0	1.72E-07	2	715.57849	1430.14971	0.18
IRLENEIQTYR	Keratin, type I cytoskeletal 10 n=12 Tax=Eutheria R	1	1	P13645		0	3.17E-06	2	718.36572	1435.72417	0.48
AENPEQQLKQEK	Involucrin n=2 Tax=Homo sapiens RepID=INVO_HU	3	1	P07476		0	8.79E-06	2	721.87024	1442.7332	0.5
VVAGVANALAHKYH	Hemoglobin subunit beta n=66 Tax=Eutheria RepID	2	1	P60524		0	9.36E-05	2	725.63538	1450.26348	0.23
TSSANIIPHSTGAAK	glyceraldehyde-3-phosphate dehydrogenase [Lacto	1	1	297206264		0	0.00232	2	728.34009	1455.6729	0.46
LGHPDTLNQGEFK	Protein S100-A9 n=5 Tax=Hominoidea RepID=S10A	1	1	P06702		0	7.30E-09	2	728.80713	1456.60698	0.44
SRAEAESWYQTK	Keratin, type II cytoskeletal 6A n=25 Tax=Euarchont	2	2	P02538;F6RMN8		0	0.00754	2	728.60938	1456.21147	0.26
GQELSRLEAAILR	Envoplakin n=5 Tax=Simiiformes RepID=EVPL_HUM	2	1	Q92817		0	0.0068	2	728.74292	1456.47856	0.33
FLEQQNQVLQTK	Keratin, type II cytoskeletal 1 n=7 Tax=Eutheria Rep	1	1	P04264		0	5.62E-06	2	738.77338	1476.53948	0.38
WELLQQVDTSTR	Keratin, type II cytoskeletal 1 n=7 Tax=Eutheria Rep	1	1	P04264		0	2.19E-09	2	738.84143	1476.67558	0.46
FLEQQNKVLETK	Keratin, type II cytoskeletal 6A n=25 Tax=Euarchont	2	2	P02538;Q8N1N4		0	6.79E-06	2	739.38367	1477.76006	0.48
FQNSLKELQLQK	Desmoplakin n=28 Tax=Mammalia RepID=DESP_HU	1	1	P15924		0	1.76E-07	2	738.9469	1476.88652	0.53
SQYEQLAEQNRK	Keratin, type I cytoskeletal 10 n=12 Tax=Eutheria R	1	1	P13645		0.00258	0.0539	2	747.63123	1494.25517	0.26
SLLGEVEQNLQAAK	Periplakin n=3 Tax=Hominoidea RepID=PEPL_HUM	1	1	O60437		0	2.66E-06	2	750.86768	1500.72807	0.46
NLLSVAYKNVVGAR	14-3-3 protein zeta/delta n=26 Tax=Amniota RepID	1	1	P63104		0	1.45E-09	2	752.89734	1504.7874	0.46
VKEGMNIVEAMER	Peptidyl-prolyl cis-trans isomerase A n=98 Tax=The	1	1	P62937		0	6.59E-10	2	753.85498	1506.70268	0.48
VLKQVHPDTGISSK	Histone H2B type 1-J n=102 Tax=Euteleostomi RepI	4	1	P06899		0	1.67E-10	2	755.41364	1509.81999	0.49
LLRDYQELMNTK	Keratin, type II cytoskeletal 1 n=7 Tax=Eutheria Rep	1	1	P04264		0	0.000288	2	762.56897	1524.13066	0.17
MLREYQELMSVK	cDNA FLJ55805, highly similar to Keratin, type II cyt	2	1	B4DRW1		0	3.95E-07	2	764.3656	1527.72392	0.48
VGAHAGEYGAEALER	Hemoglobin subunit alpha n=29 Tax=Primates RepI	1	1	P69905		0	1.12E-08	2	765.81189	1530.6165	0.44
TFAPEEISAMVLTK	78 kDa glucose-regulated protein n=68 Tax=Coelom	1	1	P11021		0	1.04E-07	2	769.3985	1537.78972	0.5
LLREYQELMNTK	Keratin, type II cytoskeletal 5 n=17 Tax=Eutheria Re	2	2	P13647;UPI00020CE287		0	8.28E-07	2	769.78467	1538.56206	0.38
ISMPDIDLNLKGPK	Neuroblast differentiation-associated protein AHN	1	1	Q09666		0	1.65E-08	2	770.79932	1540.59136	-0.12
LLRDYQELmNTK	Keratin, type II cytoskeletal 1 n=7 Tax=Eutheria Rep	1	1	P04264	M9(Oxidation)	0	4.02E-05	2	770.84546	1540.68364	0.45
mLREYQELMSVK	cDNA FLJ55805, highly similar to Keratin, type II cyt	2	1	B4DRW1	M1(Oxidation)	0	0.000233	2	772.32953	1543.65178	0.44
GVDEATIIDILTKR	Annexin A1 n=14 Tax=Eutheria RepID=ANXA1_HUM	1	1	P04083		0	1.25E-11	2	772.65125	1544.29521	0.21
MQKEITALAPSTMK	Uncharacterized protein n=11 Tax=Simiiformes Rep	3	1	F5H0N0		0	1.07E-10	2	775.34875	1549.69023	0.44
GTDVNVFNTILTTR	Annexin A1 n=14 Tax=Eutheria RepID=ANXA1_HUM	1	1	P04083		0	4.57E-10	2	776.03552	1551.06377	0.12
NKLEGLEDALQKAK	Keratin, type II cytoskeletal 6A n=25 Tax=Euarchont	2	2	P02538;F6RMN8		0	0.00843	2	779.42871	1557.85015	0.49
mQKEITALAPSTMK	Uncharacterized protein n=11 Tax=Simiiformes Rep	3	1	F5H0N0	M1(Oxidation)	0	2.35E-05	2	783.43274	1565.8582	0.53
ALGVSNFSHFQIEK	Aldo-keto reductase family 1 member B10 n=4 Tax	1	1	O60218		0	0.000605	2	789.59863	1578.18999	0.69
ALAAAGYDVEKNNSR	Histone H1.2 n=12 Tax=Eutheria RepID=H12_HUM	4	1	P16403		0	2.74E-10	2	790.01123	1579.01518	0.11
LLQDFFNGRDLNK	Uncharacterized protein n=3 Tax=Simiiformes RepII	3	1	F6Y193		0	3.70E-05	2	790.96643	1580.92558	0.55
EEKFWPNKNTYK	Serpin B3 n=14 Tax=Hominoidea RepID=SPB3_HUM	2	2	P29508;C9JZ65		0	0.000173	2	792.75165	1584.49602	0.36
VLHFDQVTENTTGK	Serpin B3 n=14 Tax=Hominoidea RepID=SPB3_HUM	1	1	P29508		0	3.15E-08	2	794.97876	1588.95024	0.08
NKLNDLEDALQQAK	Keratin, type II cytoskeletal 1 n=7 Tax=Eutheria Rep	1	1	P04264		0	4.57E-08	2	800.84387	1600.68047	0.42
YEEIVKEVSTYIK	Eukaryotic translation elongation factor 1 alpha (Fr	3	1	A9X7H1		0	1.22E-08	2	801.43384	1601.8604	0.51
ALTGHLEEVVLALLK	Annexin A1 n=14 Tax=Eutheria RepID=ANXA1_HUM	1	1	P04083		0	4.35E-09	2	803.9386	1606.86992	0.46
NLLSVAYKNVVGGQR	14-3-3 protein sigma n=20 Tax=Eutheria RepID=143	1	1	P31947		0	3.89E-06	2	809.60181	1618.19634	0.14
WNLLQQQTTTTSSK	cDNA FLJ55805, highly similar to Keratin, type II cyt	2	1	B4DRW1		0	8.89E-14	2	818.79041	1636.57353	0.37
SLNNQFASFIDKVR	Keratin, type II cytoskeletal 1 n=7 Tax=Eutheria Rep	1	1	P04264		0	2.96E-07	2	820.07861	1639.14995	0.15
DLAKDITSDTSGDFR	Annexin A1 n=14 Tax=Eutheria RepID=ANXA1_HUM	1	1	P04083		0	3.98E-08	2	821.04559	1641.08391	0.15
TLDLGEAGDNVGVLLR	elongation factor EF1A [Lactobacillus jensenii JV-V1	3	1	297205850		0	3.03E-14	2	821.92004	1642.83281	0.48
AQLGGPEAAKSDETAAK	Heat shock protein beta-1 n=6 Tax=Simiiformes Rep	1	1	P04792		0	2.36E-10	2	822.76605	1644.52483	0.35
TNETYGKLEAVQYK	Cystatin-A n=6 Tax=Catarrhini RepID=CYTA_HUMA	1	1	P01040		0	3.24E-09	2	822.81018	1644.61308	0.39
TLNNKFASFIDKVR	Keratin, type II cytoskeletal 6A n=25 Tax=Euarchont	3	3	P02538;P13647;F6RMN8		0	4.91E-11	2	827.09399	1653.18071	0.13
TLNNQFASFIDKVR	Keratin, type II cytoskeletal 78 n=7 Tax=Hominoide	1	1	Q8N1N4		0	1.64E-11	2	827.09399	1653.18071	0.15
TPAQFDADELRAAMK	Annexin A1 n=14 Tax=Eutheria RepID=ANXA1_HUM	1	1	P04083		0	1.02E-10	2	832.86267	1664.71807	0.45
SNAQRQDIAFAYQR	Annexin A2 n=28 Tax=Eutheria RepID=ANXA2_HUM	1	1	P07355		0	9.81E-06	2	834.91504	1668.8228	0.5
DVDAAYLNKVELEAK	cDNA FLJ55805, highly similar to Keratin, type II cyt	2	1	B4DRW1		0	5.17E-06	2	839.58398	1678.16069	0.15
SLVNLGGSKSISISVAR	Keratin, type II cytoskeletal 1 n=7 Tax=Eutheria Rep	1	1	P04264		0	1.37E-13	2	844.74683	1688.48638	0.26
KKINSWVESQTNEK	Serpin B3 n=14 Tax=Hominoidea RepID=SPB3_HUM	2	2	P29508;C9JZ65		0	1.36E-09	2	846.08142	1691.15557	0.14
GSVYIGGAPDVATLTGGR	Basement membrane-specific heparan sulfate prote	1	1	P98160		0	2.30E-12	2	846.41492	1691.82256	0.47
NLEPLFETYLSVLR	cDNA FLJ55805, highly similar to Keratin, type II cyt	2	1	B4DRW1		0	1.68E-07	2	847.62927	1694.25127	0.17
TKDGVVEITGKHEER	Heat shock protein beta-1 n=6 Tax=Simiiformes Rep	1	1	P04792		0	9.01E-08	2	849.59979	1698.19231	0.16
SEIDMNDIKAFYQK	Annexin A1 n=14 Tax=Eutheria RepID=ANXA1_HUM	1	1	P04083		0	5.96E-09	2	851.78961	1702.57195	0.38
AVFVDLEPTVIDEVR	Tubulin alpha-1A chain n=597 RepID=TBA1A_HUM	2	1	Q71U36		0	5.00E-12	2	851.92346	1702.83965	0.47
GLGTDEDTLIEILASR	Annexin A1 n=14 Tax=Eutheria RepID=ANXA1_HUM	1	1	P04083		0	2.42E-09	2	852.3761	1703.74492	0.43

Sequence	Protein Descriptions	# Proteins	# Protein Groups	Protein Group Accessions	Modifications	q-Value	PEP	Charge	m/z [Da]	MH+ [Da]	Delta Mass [Da]
DSTIQVVENGESSQGR	Uncharacterized protein n=3 Tax=Simiiformes RepII	2	1	E9PFJ3		0	4.30E-12	2	853.76025	1706.51323	0.36
GSLGGGFSSGGFSGGSFSR	Keratin, type I cytoskeletal 10 n=12 Tax=Eutheria R	1	1	P13645		0	3.36E-15	2	854.79919	1708.59111	0.41
VPEPGSIKVPDQGFIK	Small proline-rich protein 3 n=4 Tax=Homininae Re	1	1	Q9UBC9		0	5.12E-06	2	856.34277	1711.67827	0.37
QISNLQQSISDAEQR	Keratin, type II cytoskeletal 1 n=7 Tax=Eutheria Rep	1	1	P04264		0	1.88E-11	2	859.15057	1717.29387	0.22
NLDIERPTYTNLNR	Tubulin alpha-1A chain n=597 RepID=TBA1A_HUM	2	1	Q71U36		0	0.00186	2	860.46729	1719.92729	0.52
PQLLQNINGIIEAFR	Cornulin n=6 Tax=Catarrhini RepID=CRNN_HUMAN	1	1	Q9UBG3		0	1.23E-11	2	863.55969	1726.11211	0.07
QLLQANPILEAFGNAK	Uncharacterized protein n=10 Tax=Catarrhini RepID	5	1	F2Z2S7		0	2.08E-07	2	864.39014	1727.773	0.41
LLEAQAATGGIVDLLSR	Envoplakin n=5 Tax=Simiiformes RepID=EVPL_HUM	2	1	Q92817		0	7.93E-16	2	864.44446	1727.88164	0.46
SEDFGVNEDLADSDAR	Annexin A1 n=14 Tax=Eutheria RepID=ANXA1_HUM	1	1	P04083		0	8.03E-10	2	870.47852	1739.94975	0.11
VLDTKWTLLQEQGTK	Keratin, type II cytoskeletal 5 n=17 Tax=Eutheria Re	2	2	P13647;F6RMN8		0	2.66E-09	2	880.8645	1760.72173	0.38
QTFTPPPQLQQQQVK	Small proline-rich protein 3 n=4 Tax=Homininae Re	1	1	Q9UBC9		0	3.33E-09	2	884.58728	1768.16728	0.11
AmGImNSFVNDIFER	Histone H2B type 1-J n=102 Tax=Euteleostomi RepI	4	1	P06899	M2(Oxidation); M5(Oxidation)	0	1.29E-05	2	888.71948	1776.43169	0.31
GADFLVTEVENGGSLGSK	Pyruvate kinase n=4 Tax=Simiiformes RepID=E9PF7	5	1	E9PF79		0	1.80E-12	2	890.73718	1780.46709	0.3
EYGGLDVLVNNAGIAFK	Carbonyl reductase 1, isoform CRA_c n=2 Tax=Simii	4	1	B4DFK7		0	9.08E-10	2	890.948	1780.88872	0.48
SLGFDKVFmYLSDSR	Uncharacterized protein n=3 Tax=Simiiformes RepII	2	1	E9PFJ3	M9(Oxidation)	0	2.57E-05	2	891.40405	1781.80083	0.47
VSLDVNHFAPDELTVK	Heat shock protein beta-1 n=6 Tax=Simiiformes Rep	1	1	P04792		0	4.62E-07	2	892.90686	1784.80644	0.44
ISPQIQLSGQTEQTQK	Cornulin n=6 Tax=Catarrhini RepID=CRNN_HUMAN	1	1	Q9UBG3		0	7.75E-13	2	893.83752	1786.66777	0.37
IINEPTAAAIAYGLDKK	Uncharacterized protein n=1 Tax=Homo sapiens Re	4	1	E9PI65		0	8.22E-11	2	894.84497	1788.68266	0.35
QSPASPERDYSPYYK	Keratin, type I cytoskeletal 13 n=13 Tax=Simiiforme	1	1	P13646		0	4.95E-09	2	894.95874	1788.9102	0.54
SYELPDGQVITIGNER	Uncharacterized protein n=11 Tax=Simiiformes Rep	3	1	F5H0N0		0	1.12E-10	2	896.30579	1791.6043	0.36
MAPYQGPDAVPGALDYK	Actinin alpha4 isoform n=3 Tax=Simiiformes RepID=	3	1	A4K467		0	4.63E-15	2	897.22668	1793.44609	0.29
QPSQPPPQEIFVPTTK	Small proline-rich protein 3 n=4 Tax=Homininae Re	1	1	Q9UBC9		0	3.17E-05	2	897.84119	1794.6751	0.37
GFDEYMKELGVGIALR	Fatty acid-binding protein, epidermal n=10 Tax=Sim	1	1	Q01469		0	4.12E-10	2	899.87622	1798.74516	0.41
VELEAALQQAKEELAR	cDNA FLJ55805, highly similar to Keratin, type II cyt	2	1	B4DRW1		0	3.24E-10	2	899.92383	1798.84038	0.44
NIETIINTFHQYSVK	Protein S100-A9 n=5 Tax=Hominoidea RepID=S10A	1	1	P06702		0	0.000443	2	904.47351	1807.93974	0.5
TLQGLEIELQSQLSMK	Keratin, type I cytoskeletal 19 n=9 Tax=Eutheria Re	3	2	A4FUA1;P13646		0	1.57E-11	2	909.55493	1818.10259	0.07
RFDEILEASDGIMVAR	Pyruvate kinase n=4 Tax=Simiiformes RepID=E9PF7	5	1	E9PF79		0	7.91E-09	2	911.49487	1821.98247	0.03
VQQLQISVDQHGDNLK	cDNA FLJ55805, highly similar to Keratin, type II cyt	2	1	B4DRW1		0	7.80E-15	2	911.81213	1822.61699	0.34
NLEPLFETYLSVLRK	cDNA FLJ55805, highly similar to Keratin, type II cyt	2	1	B4DRW1		0	4.35E-13	2	911.75208	1822.49687	0.24
QEAQLELPEQQVGQPK	Involucrin n=2 Tax=Homo sapiens RepID=INVO_HU	3	1	P07476		0	1.70E-05	2	912.16052	1823.31377	0.69
VLAEMREQYEAMAER	Keratin, type I cytoskeletal 13 n=13 Tax=Simiiforme	1	1	P13646		0	6.41E-06	2	913.88464	1826.76201	0.45
SIYGEKFEDENFILK	Peptidyl-prolyl cis-trans isomerase A n=98 Tax=The	1	1	P62937		0	4.31E-06	2	916.49976	1831.99224	0.04
RKGTDVNVFNTILTTR	Annexin A1 n=14 Tax=Eutheria RepID=ANXA1_HUM	1	1	P04083		0	1.27E-13	2	918.44348	1835.87969	0.43
VLAEMREQYEAmAER	Keratin, type I cytoskeletal 13 n=13 Tax=Simiiforme	1	1	P13646	M12(Oxidation)	0	4.40E-06	2	921.96033	1842.91338	0.53
LQLEAVNITDLSENRK	Interleukin-1 receptor antagonist protein n=4 Tax=	1	1	P18510		0	8.11E-10	2	922.35889	1843.7105	0.36
VSLKTALQPmVSALNIR	Uncharacterized protein n=3 Tax=Simiiformes RepII	2	1	E9PFJ3	M10(Oxidation)	0	3.46E-07	2	929.42432	1857.84136	0.39
SISNKELSELIEQLQK	Periplakin n=3 Tax=Hominoidea RepID=PEPL_HUM	1	1	O60437		0	3.67E-09	2	930.35925	1859.71123	0.35
DQVVGVVGTGHIGQVFMK	D-lactate dehydrogenase [Lactobacillus jensenii JV-	1	1	297205326		0	2.57E-10	2	935.92163	1870.83599	-0.07
QYNSFNFALLEDVQAK	Uncharacterized protein n=1 Tax=Homo sapiens Re	1	1	C9JZ65		0	1.27E-11	2	944.4408	1887.87432	0.47
LIDQTMIDLDGTPNKGR	enolase [Lactobacillus jensenii JV-V16]	2	1	297206261		0	3.75E-07	2	944.45984	1887.9124	0.47
LAEQAERYEDMAAFMK	14-3-3 protein sigma n=20 Tax=Eutheria RepID=143	1	1	P31947		0	0.000213	2	952.23328	1903.45928	0.29
AAYLQETGKPLDETLKK	Annexin A1 n=14 Tax=Eutheria RepID=ANXA1_HUM	1	1	P04083		0	1.60E-07	2	953.40637	1905.80547	0.39
LATQSNEITIPVTFESR	Heat shock protein beta-1 n=6 Tax=Simiiformes Rep	1	1	P04792		0	0.000263	2	953.86658	1906.72588	0.37
ITMQNLNDRLASYLEK	Keratin, type I cytoskeletal 13 n=13 Tax=Simiiforme	1	1	P13646		0	8.58E-06	2	955.4425	1909.87773	0.45
KVPDPVLEESFQQLQR	Periplakin n=3 Tax=Hominoidea RepID=PEPL_HUM	1	1	O60437		0	2.14E-10	2	957.44836	1913.88945	0.44
ITmQNLNDRLASYLEK	Keratin, type I cytoskeletal 13 n=13 Tax=Simiiforme	1	1	P13646	M3(Oxidation)	0	8.30E-09	2	963.39563	1925.78398	0.4
TLVTQNSGVEALIHAILR	Junction plakoglobin n=31 Tax=Theria RepID=PLAK_	1	1	P14923		0	9.67E-12	2	968.48132	1935.95537	0.43
TDLEKDIISDTSGDFRK	Annexin A2 n=28 Tax=Eutheria RepID=ANXA2_HUM	1	1	P07355		0	3.83E-10	2	970.88806	1940.76885	0.4
FLAGSTLNPSETIEFEGK	50S ribosomal protein L31 [Lactobacillus jensenii JV	1	1	297205124		0	1.06E-09	2	971.00562	1941.00395	0.52
LNDLEDALQQAKEDLAR	Keratin, type II cytoskeletal 1 n=7 Tax=Eutheria Rep	1	1	P04264		0	3.05E-09	2	971.82642	1942.64556	0.33
FASFIDKVQFLEQQNK	cDNA FLJ55805, highly similar to Keratin, type II cyt	2	1	B4DRW1		0	1.87E-10	2	971.89624	1942.7852	0.39
TVAAPSVFIFPPSDEQLK	Ig kappa chain C region n=2 Tax=Homo sapiens Rep	21	1	P01834		0	7.98E-05	2	973.92761	1946.84795	0.41
LGHPDTLNQGEFKELVR	Protein S100-A9 n=5 Tax=Hominoidea RepID=S10A	1	1	P06702		0	1.23E-09	2	977.5022	1953.99712	0.49
VAPEEHPVLLTEAPLNPK	Uncharacterized protein n=11 Tax=Simiiformes Rep	3	1	F5H0N0		0	4.93E-10	2	977.91284	1954.81841	0.38
LDSELKNMQDMVEDYR	Keratin, type II cytoskeletal 1 n=7 Tax=Eutheria Rep	1	1	P04264		0	1.03E-10	2	993.81934	1986.6314	0.37
THNLEPYFESFINNLR	Keratin, type II cytoskeletal 1 n=7 Tax=Eutheria Rep	1	1	P04264		0	1.28E-07	2	998.00989	1995.0125	0.52
VSLDVNHFAPDELTVKTK	Heat shock protein beta-1 n=6 Tax=Simiiformes Rep	1	1	P04792		0	1.08E-11	2	1007.38831	2013.76933	0.35
SLYYIQQDTKGDYQK	Annexin A2 n=28 Tax=Eutheria RepID=ANXA2_HUM	1	1	P07355		0	1.05E-10	2	1007.35913	2013.71099	0.38
GIVDQSQQAYQEAFEISK	14-3-3 protein zeta/delta n=26 Tax=Amniota RepID	1	1	P63104		0	1.76E-09	2	1021.32983	2041.65239	0.33
LSLQDAVSQGVIDQDMATR	Desmoplakin n=28 Tax=Mammalia RepID=DESP_HU	1	1	P15924		0	9.94E-06	2	1023.96521	2046.92314	-0.04
SAELNKEVSTNTAMIQTSK	GUCA1B protein (Fragment) n=1 Tax=Homo sapiens	2	2	A4FUA1;P13646		0	3.05E-16	2	1026.95081	2052.89433	0.43
VLDSEKLLYVLEADAAIAK	Periplakin n=3 Tax=Hominoidea RepID=PEPL_HUM	1	1	O60437		0	2.31E-11	2	1031.6355	2062.26372	0.56
GDRSEDFGVNEDLADSDAR	Annexin A1 n=14 Tax=Eutheria RepID=ANXA1_HUM	1	1	P04083		0	3.60E-15	2	1034.86316	2068.71904	0.42
SAELNKEVSTNTAmIQTSK	GUCA1B protein (Fragment) n=1 Tax=Homo sapiens	2	2	A4FUA1;P13646	M14(Oxidation)	0	2.67E-12	2	1034.91077	2068.81426	0.4
LGHPDTLNQGEFKELVRK	Protein S100-A9 n=5 Tax=Hominoidea RepID=S10A	1	1	P06702		0	2.56E-05	2	1041.57532	2082.14336	0.51
YGINTTDIFQTVDLWEGK	Transgelin-2 n=14 Tax=Theria RepID=TAGL2_HUM	1	1	P37802		0	2.42E-13	2	1050.74829	2100.48931	0.23
ALPGQLKPFETLLSQNQGGK	Glutathione S-transferase P n=4 Tax=Simiiformes R	1	1	P09211		0	1.58E-08	2	1064.02771	2127.04814	0.44
TIEELRDKILTATIENNR	Keratin, type I cytoskeletal 13 n=13 Tax=Simiiforme	1	1	P13646		0	1.36E-08	2	1065.5813	2130.15532	0.5
QAWFIENEEQEYVQTVK	Annexin A1 n=14 Tax=Eutheria RepID=ANXA1_HUM	1	1	P04083		0	8.91E-11	2	1071.42188	2141.83647	0.41
ITMQNLNDRLASYLEKVR	Keratin, type I cytoskeletal 13 n=13 Tax=Simiiforme	1	1	P13646		0	8.42E-08	3	722.24316	2164.71494	0.19
VQQLQISVDQHGDNLKNTK	cDNA FLJ55805, highly similar to Keratin, type II cyt	2	1	B4DRW1		0	1.68E-11	2	1083.55542	2166.10356	0.49
ILTATIENNRVILEIDNAR	Keratin, type I cytoskeletal 13 n=13 Tax=Simiiforme	1	1	P13646		0	5.51E-05	2	1084.9231	2168.83891	0.32
NKLNDLEDALQQAKEDLAR	Keratin, type II cytoskeletal 1 n=7 Tax=Eutheria Rep	1	1	P04264		0	2.65E-14	2	1092.95728	2184.90727	0.39
IGPILDNSTLQSEVKPILEK	Uncharacterized protein n=2 Tax=Theria RepID=B4E	5	1	B4E1Q0		0	3.55E-05	3	732.51599	2195.53342	0.43
VLETKWNLLQQQTTTTSSK	cDNA FLJ55805, highly similar to Keratin, type II cyt	2	1	B4DRW1		0	7.20E-12	2	1103.9646	2206.92192	0.38
ALEEANADLEVKIRDWHLK	Keratin, type I cytoskeletal 13 n=13 Tax=Simiiforme	1	1	P13646		0	9.30E-07	3	751.15662	2251.4553	0.42
TDLEMQIESLNEELAYMKK	Keratin, type I cytoskeletal 13 n=13 Tax=Simiiforme	1	1	P13646		0	1.91E-16	2	1143.35852	2285.70976	0.3
AEAESLYQSKYEELQITAGR	Keratin, type II cytoskeletal 1 n=7 Tax=Eutheria Rep	1	1	P04264		0	4.45E-10	2	1143.98352	2286.95976	0.42
SLDLDSIIAEVKAQYEDIAQK	Keratin, type II cytoskeletal 1 n=7 Tax=Eutheria Rep	1	1	P04264		0	8.55E-10	2	1175.53699	2350.0667	0.42
GGPGSAVSPYPTFNPSSDVAALHK	Annexin A1 n=14 Tax=Eutheria RepID=ANXA1_HUM	1	1	P04083		0	8.15E-09	2	1178.89185	2356.77641	0.31
GGGGGGYGSGGSSYGSGGGSYGSGGGGGGGR	Keratin, type II cytoskeletal 1 n=7 Tax=Eutheria Rep	1	1	P04264		0	4.13E-20	2	1192.92615	2384.84502	0.45
NLDLDSIIAEVKAQYEEIAQR	Keratin, type II cytoskeletal 6A n=25 Tax=Euarchont	2	2	P02538;F6RMN8		0	2.28E-14	2	1209.9259	2418.84453	0.3
TEAESWYQTKYEELQQTAGR	Keratin, type II cytoskeletal 5 n=17 Tax=Eutheria Re	2	2	P13647;UPI00020CE287		0	1.06E-07	2	1209.98828	2418.96929	0.42
DLAKDITSDTSGDFRNALLSLAK	Annexin A1 n=14 Tax=Eutheria RepID=ANXA1_HUM	1	1	P04083		0	7.60E-07	2	1226.56567	2452.12407	0.43
VFQSLPHENKPLTLSNYQTNK	Cystatin-B n=14 Tax=Simiiformes RepID=CYTB_HUM	1	1	P04080		0	4.21E-09	2	1230.04883	2459.09038	0.41
LDQGNLHTSVSSAQGQDAAQSEEK	Cornulin n=6 Tax=Catarrhini RepID=CRNN_HUMAN	1	1	Q9UBG3		0	5.43E-13	3	834.51855	2501.54111	0.46
FASFIDKVQFLEQQNKVLETK	cDNA FLJ55805, highly similar to Keratin, type II cyt	2	1	B4DRW1		0	2.93E-08	3	838.09662	2512.2753	-0.02
LLNNKFASFIDKVQFLEQQNK	cDNA FLJ55805, highly similar to Keratin, type II cyt	2	1	B4DRW1		0	1.23E-08	3	842.41736	2525.23752	0.29
SRAEAESWYQTKYEELQVTAGR	Keratin, type II cytoskeletal 6A n=25 Tax=Euarchont	2	2	P02538;F6RMN8		0	2.06E-05	2	1302.04749	2603.08769	0.42
DNTAQQIKKVLHFDQVTENTTGK	Serpin B3 n=14 Tax=Hominoidea RepID=SPB3_HUM	1	1	P29508		0	1.68E-09	3	872.56848	2615.69089	0.12
YSPTVQTRSISNKELSELIEQLQK	Periplakin n=3 Tax=Hominoidea RepID=PEPL_HUM	1	1	O60437		0	5.60E-05	3	930.91321	2790.72507	-0.25
SQTSQAVTGGHTQIQAGSHTETVEQDR	Cornulin n=6 Tax=Catarrhini RepID=CRNN_HUMAN	1	1	Q9UBG3		0	6.77E-14	3	952.22546	2854.66184	0.44
SALSGHLETVILGLLKTPAQYDASELK	Annexin A2 n=28 Tax=Eutheria RepID=ANXA2_HUM	1	1	P07355		0	6.27E-08	3	952.6261	2855.86374	0.44
NALLSLAKGDRSEDFGVNEDLADSDAR	Annexin A1 n=14 Tax=Eutheria RepID=ANXA1_HUM	1	1	P04083		0	4.84E-12	3	960.30969	2878.91452	0.18
SAELNKEVSTNTAMIQTSKTEITELR	GUCA1B protein (Fragment) n=1 Tax=Homo sapiens	2	2	A4FUA1;P13646		0	9.06E-13	3	965.8092	2895.41306	0.31
FLEQQNQVLQTKWELLQQVDTSTR	Keratin, type II cytoskeletal 1 n=7 Tax=Eutheria Rep	1	1	P04264		0	5.83E-08	2	1467.42261	2933.83794	0.66
ALYEAELSQmQTHVSDTSVVLSMDNNR	Uncharacterized protein n=4 Tax=Simiiformes RepII	1	1	F6RMN8	M10(Oxidation)	0	0.0101	2	1527.9895	3054.97173	0.28
DVDAAYLNKVELEAKVDSLNDEINFLK	cDNA FLJ55805, highly similar to Keratin, type II cyt	2	1	B4DRW1		0	1.33E-12	3	1022.63501	3065.89048	0.11
YGKDATNVGDEGGFAPNILENKEGLELLK	Alpha-enolase n=53 Tax=Euteleostomi RepID=ENO	1	1	P06733		0	2.87E-09	3	1031.42151	3092.24997	0.23
YTLPPGVDPTQVSSSLSPEGTLTVEAPMPK	Heat shock protein beta-1 n=6 Tax=Simiiformes Rep	1	1	P04792		0	2.57E-09	2	1550.41614	3099.825	0.63
KYTLPPGVDPTQVSSSLSPEGTLTVEAPMPK	Heat shock protein beta-1 n=6 Tax=Simiiformes Rep	1	1	P04792		0	3.61E-10	2	1614.12732	3227.24736	0.3
AHQTGETVTGSGTQTQAGATQTVEQDSSHQTGR	Cornulin n=6 Tax=Catarrhini RepID=CRNN_HUMAN	1	1	Q9UBG3		0	6.16E-09	2	1679.11462	3357.22197	0.34
EQDRAHQTGETVTGSGTQTQAGATQTVEQDSSHQTGR	Cornulin n=6 Tax=Catarrhini RepID=CRNN_HUMAN	1	1	Q9UBG3		0	4.16E-14	3	1296.04309	3886.11472	0.45
GSYGSGGSSYGSGGGSYGSGGGGGGHGSYGSGSSSGGYRGGSGGGGGGSSGGR	Keratin, type II cytoskeletal 1 n=7 Tax=Eutheria Rep	1	1	P04264		0	6.46E-28	3	1434.36426	4301.07822	0.45
xDALMDEINFMKmFFDAELSQMQTHVSDTSVVLSMDNNR	UPI00020CE287 related cluster n=1 Tax=Homo sapi	1	1	UPI00020CE287	N-Term(Acetyl); X1(D); M13(Oxidation	0	0.0354	3	1527.9895	4581.95395	-0.35
xDALmDEINFMKmFFDAELSQMQTHVSDTSVVLSMDNNR	UPI00020CE287 related cluster n=1 Tax=Homo sapi	1	1	UPI00020CE287	X1(V); N-Term(Acetyl); M5(Oxidation);	0	0.000307	3	1527.91724	4581.73716	-0.44
xDALMDEINFMKMFFDAELSQMQTHVSDTSVVLSMDNNR	UPI00020CE287 related cluster n=1 Tax=Homo sapi	1	1	UPI00020CE287	N-Term(Acetyl); X1(M)	0	0.00353	3	1527.91724	4581.73716	-0.43
xDALMDEINFMKmFFDAELSQMQTHVSDTSVVLSMDNNR	UPI00020CE287 related cluster n=1 Tax=Homo sapi	1	1	UPI00020CE287	N-Term(Acetyl); X1(N); M13(Oxidation	0	0.0283	3	1527.91724	4581.73716	-0.1
xDALMDEINFMKmFFDAELSQMQTHVSDTSVVLSMDNNR	UPI00020CE287 related cluster n=1 Tax=Homo sapi	1	1	UPI00020CE287	X1(R); M13(Oxidation)	0.00258	0.0537	3	1527.91724	4581.73716	-0.11
xDALMDEINFMKMFFDAELSQmQTHVSDTSVVLSMDNNR	UPI00020CE287 related cluster n=1 Tax=Homo sapi	1	1	UPI00020CE287	N-Term(Acetyl); X1(I); M22(Oxidation)	0	0.0329	3	1527.9895	4581.95395	0.29
xDALmDEINFMKMFFDAELSQmQTHVSDTSVVLSMDNNR	UPI00020CE287 related cluster n=1 Tax=Homo sapi	1	1	UPI00020CE287	X1(P); N-Term(Acetyl); M5(Oxidation);	0	0.0294	3	1527.9895	4581.95395	0.31
